# Effect of Explicit Hydration on the Cisplatin Reaction Mechanism with Adenine and Guanine

**DOI:** 10.3390/molecules30030510

**Published:** 2025-01-23

**Authors:** Jesús Iván Salazar-Barrientos, José Manuel Guevara-Vela, Marco A. García-Revilla, Evelio Francisco, Miguel Gallegos, Tomás Rocha-Rinza, Ángel Martín Pendás

**Affiliations:** 1 Instituto de Química, Universidad Nacional Autónoma de México, Circuito Exterior s/n, Ciudad Universitaria, Alcaldía Coyoacán, Cuidad de Mexico C.P. 04510, Mexico; 2 Departamento de Química Física Aplicada, Universidad Autónoma de Madrid, C.P. 28049 Madrid, Spain; jose.guevara@uam.es; 3 División de Ciencias Naturales y Exactas, Departamento de Química, Universidad de Guanajuato, Noria Alta, Guanajuato C.P. 36050, Mexico; magarcia@ugto.mx; 4 Departamento de Química Física y Analítica, Universidad de Oviedo, Av. Julián Clavería 8, 33006 Oviedo, Asturias, Spain; evelio@uniovi.es (E.F.); miguelgallegosgonzalez@gmail.com (M.G.)

**Keywords:** cisplatin action pathway, implicit solvation model, surrounding water molecules, surrounding water molecules, electronic and Gibbs free activation energies, quantum chemical topology, quantum theory of atoms in molecules, interacting quantum atoms, delocalisation indices

## Abstract

Cisplatin is still a first-line agent in cancer treatment due to its effectiveness. Despite the large body of research concerning this drug, the role of explicit water molecules in its mechanism remains uncertain. We addressed the addition of cisplatin with the nitrogenous DNA bases adenine and guanine, with an emphasis on the impact of explicit microsolvation on every step of the action pathway of this pharmaceutical. We used electronic structure calculations to explore the energetics of the key reactions of this mechanism. We also exploited state-of-the-art methods of wave function analyses, namely, the Quantum Theory of Atoms in Molecules and the Interacting Quantum Atoms partition, to explore the chemical bonding throughout such chemical reactions. Our results reveal that microsolvation significantly differently affects electronic and Gibbs free activation energies, as previously reported (F.P. Cossio et al. ChemPhysChem, 17, 3932, 2016). The changes in activation energies are consistent with Hammond’s postulate in terms of the changes in the chemical bonding scenario between reactants and transition states. Overall, we provide an in-depth description of the importance of the surrounding water molecules of cisplatin, which aids in understanding the mechanism of pharmaceuticals in the pursuit of more effective cancer treatments.

## 1. Introduction

**Figure 1:**
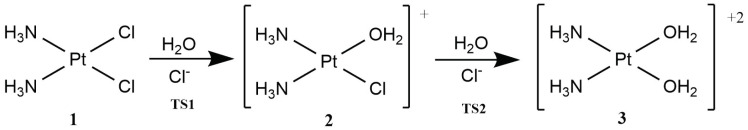
Hydration of cisplatin.

**Figure 2:**
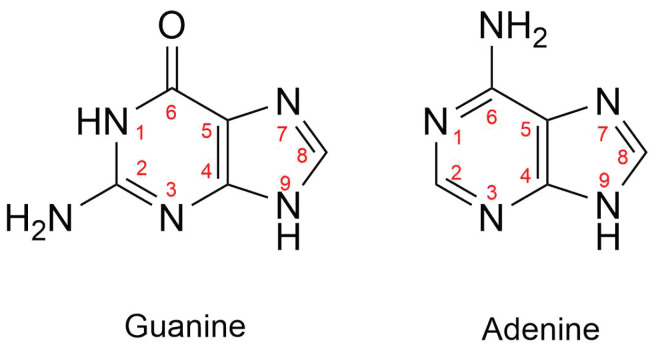
Numbering of atoms in guanine and adenine. We emphasize that cisplatin binds N7 when it interacts with either of these nitrogenous bases.

Cisplatin, *cis*-Pt(NH_3_)_2_Cl_2_ (**1**), is one of the quintessential drugs in cancer treatment [1]. Since its discovery in 1965 [2], it has been widely used in the fight against various types of cancer, such as testicular [3], ovarian [4], lung [5], and prostate cancer [6], among many others [7]. For almost six decades, the elucidation of its mechanism of action has been a topic of interest from experimental [1] and theoretical [8] perspectives. Cisplatin is a $d^{8}$ platinum (II) metal complex with a square planar geometry. This complex, however, does not react directly with DNA [9]. It must first undergo hydration reactions, in which it loses one or both chloride anions (Figure 1). These transformations occur via a characteristic substitution reaction mechanism of square planar complexes [10], wherein the transition state has a pentacoordinate trigonal bipyramid geometry [11]. It is still not clear which of the activated complexes, *cis*-diamminechloroaquaPt(II) (**2**) or *cis*-diamminediaquaPt(II) (**3**), binds directly to the DNA bases [12]. This addition is the rate-limiting step of the whole process. Previous in vitro [13] and computational [14] studies indicated the propensity of cisplatin to bind to the N7 of adenine and guanine nitrogenous bases (Figure 2).

Despite the large body of research on cisplatin, there are still unsolved issues concerning its reaction mechanism with DNA. For example, the role of the solvation water molecules in this mechanism is still unclear. Given the biological relevance of the action pathway of drugs, the availability of a hydration model capable of reliably reproducing experimental values is often pivotal. Due to this circumstance, there are studies that compare distinct solvation methods for biologically relevant reactions. For example, Lakbaibi et al. evaluated the role of explicit solvation in the Darzens reaction, one of the most useful synthetic procedures for forming epoxides, as well as natural and pharmacological products [15]. These authors found that explicit solvation significantly reduces the values of Gibbs free activation energies. This result was attributed to the capacity of the explicit solvation model to account for crucial hydrogen-bonding interactions, which are not properly described by implicit models. In another study, Wang and Cao [16] investigated the hydration of CO_2_ with both explicit and implicit methods of microhydration. Their results show that explicit solvation is critical for a reasonable description of the proton relay mechanism that occurs in this system. Alberto et al. [17] addressed the hydrolysis mechanism of a platinum-derivative anticancer drug [18], Nedaplatin, with an implicit solvation model and the influence of an extra explicit water molecule. These authors concluded that the explicit water molecule incorporates the effects of hydrogen bonding and provides a more accurate description of the stabilization of the leaving group. More specifically for the investigation reported herein, there are still unsolved issues regarding the role of microsolvation in the mechanism of cisplatin in spite of the considerable interest in this pharmaceutical. A related study on this matter [19] highlighted the significant influence of explicit water molecules for the underlying interactions governing the addition reaction mechanisms of cisplatin with DNA bases. In this investigation, Cossio et al. concluded that the incorporation of explicit water molecules reduces the Gibbs free activation energy in every step of the action mechanism of cisplatin. The utilization of explicit microhydration led to computational results that agree closely with experimental observations [19]. Unfortunately, the consideration of electronic energies without explicit H_2_O solvation molecules in this same study yields questionable negative activation energies for the second hydration of cisplatin [19]. Besides, the examination of interatomic distances of some reactants and the corresponding transition states indicates that the water molecules surrounding the reactive system interact more strongly with the reactants than with the transition state. Hence, one would expect that the activation energy of such steps of the action mechanism of cisplatin increases as a result of the consideration of explicit microhydration, as opposed to the conclusions reported by Cossio et al. [19]. Therefore, we decided to revisit the effect of explicit microhydration on the biological mechanism of cisplatin. For this purpose, we also exploited state-of-the-art methods of wave function analyses to gain further insights into the changes in the chemical bonding scenario in these reactive systems due to the incorporation of explicit H_2_O solvation molecules. It is well known, indeed, that non-covalent interactions play a fundamental role in a drug’s mechanism of action [20]. Since the hydration of cisplatin is the key step for the activation of this drug, it is vital to understand the role of hydrogen bonding in this reaction. Indeed, we have previously investigated the role of H-bonds in the catalysis of chemical reactions in detail [21,22]. More specifically, for the matter at hand, there are previous reports about non-covalent interactions occurring between Pt and water molecules. Kozelka et al. [23] investigated the interaction of a water molecule and two platinum (II) complexes at the MP2 level of theory. Their results indicate that the O−H⋯Pt interaction has a strong dispersion component that highly contributes to the energetics of the contact between water molecules and platinum(II) in force-field studies of solvated platinum complexes. Veljović et al. [24] evaluated X–H/Pt interactions in cisplatin and trasplatin. These workers concluded that both platinum complexes are able to form strong interactions with water molecules and that these interactions can be recognized in crystal structures. Bergés and coworkers [25] established energetic and topological descriptors of O−H⋯Pt bonds of different Pt(II) complexes that are axially approached by a water molecule using the MP2 method and the Quantum Theory of Atoms in Molecules (QTAIM) method of wavefunction analyses. Robertazzi et al. [26] considered thermodynamical properties and effects of explicit solvation in the first hydration shell of cisplatin using electronic structure calculations and the QTAIM. As opposed to the conclusions of Cossio et al. [19], these authors found that explicit solvation substantially distorts the cisplatin geometry and leads to higher activation energies while forming a solvation sphere with ten explicit water molecules.

In view of this background, we aim to elucidate the role of non-covalent interactions in the reactivity of cisplatin across its biological mechanism with the aid of wave function analyses. We also focused on the chemical bonds that are formed and destroyed throughout the whole reaction mechanism of cisplatin based on electronic structure theory and Quantum Chemical Topology (QCT) methods, namely, QTAIM and the Interacting Quantum Atoms (IQA) energy partition. In short, we address the following in this study: (i) the steps of the biological mechanism of cisplatin and their corresponding activation energies and (ii) the influence of explicit water molecules on the chemical bonding of the reactants and transition states for a better understanding of their effect on the activation energies along the biological mechanism of cisplatin. Electronic structure and QCT calculations indicate that the interaction of the explicit solvation water molecules stabilizes (i) reactants or (ii) transition states to a greater extent, leading to an increase or decrease in the corresponding activation energy, respectively. Finally, we observe the changes in the chemical bonding scenario of leaving and entering groups throughout the biological mechanism of cisplatin via delocalization indices as defined by the QTAIM. Overall, this study illustrates how the combination of suitable electronic structure calculations and QCT analyses provides (i) a well-suited assessment of the effects of explicit solvation water molecules on activation energies and (ii) valuable insights into such effects in terms of changes in the chemical bonding scenario within the actin pathway of cisplatin.

## 2. Results and Discussion

**Figure 3:**
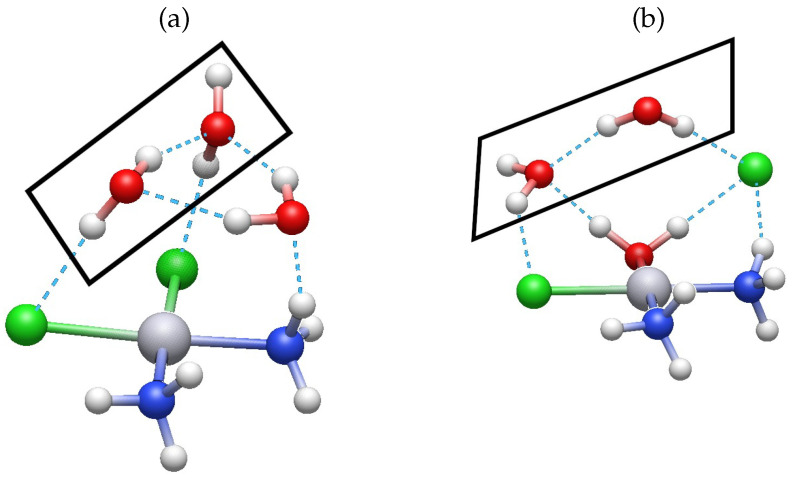
Structures of (**a**) the reactants and (**b**) the products of the first hydration of cisplatin (Figure 1) with the two explicit solvation water molecules enclosed within a rectangle.

We first discuss the hydration of cisplatin, as schematized in Figure 1. It is well known that the first hydration of cisplatin is the rate-limiting step for its activation in a cell [7]. Therefore, we set out to investigate the effect of microhydration on the activation energy for this first hydration step with different approximations. We considered two explicit water molecules along the entire reaction mechanism to have comparable results with previously reported activation energies. As Cossio et al. [19] wrote, *“going from zero to two surrounding water molecules, the computed activation and reaction energies can vary substantially, particularly the Gibbs activation and reaction energies. However, our results also show that for a reliable representation of the system, only two molecules of water need to be considered”* [19]. Figure 3 illustrates the effect of the solvation water molecules in the first hydration of cisplatin. The encompassing H_2_O monomers interact with the nucleophilic water molecule to form a water trimer, as shown in Figure 3a. Likewise, one of the surrounding water molecules (Figure 3b) forms a hydrogen bond with the Cl^-^ leaving group. Certainly, the encircling water molecules in all of the addressed reactions in this investigation interact with either the entering or the leaving groups, thereby altering the overall energetics and activation energies of such reactions.

The corresponding electronic and Gibss free activation energies are reported in Table 1. The consideration of microhydration in the system increases the electronic and the Gibbs free activation energies in contrast with the results of reference [de2016new]. We also observed that there are no large differences between $\Delta E^{‡}$ and $\Delta G^{‡}$ (>1.7 kcal/mol). The electronic activation energy computed with the M06-2X approximation along with Basis Set 1 with implicit solvation effects (20.57 kcal/mol) agrees with that experimentally determined by Repta and Long [27] (19–20 kcal/mol). However, by using the same approximate functional and basis set but considering explicit microsolvation (22.04 kcal/mol), our results agree with the experimental findings of Brancroft et al. [28] (22.25 kcal/mol). The difference in the activation barriers computed with the PBE0 and M06-2X functionals is significant (≈4kcal/mol), and, therefore, we conducted DLPNO- CCSD(T)/def2-TZVP single-point calculations to provide a better assessment of the correctness of these values. Table S1 reports the results of these calculations. We note overall that the DLPNO-CCSD(T)/def2-TZVP values of activation energies have a better agreement with those computed with the PBE0 functional. Regardless of the consideration of microsolvation, the values of $\Delta G_{\mathrm{rxn}}$, i.e., the change in Gibbs free energy for the overall reaction, indicate that the reaction is endergonic. Nevertheless, microsolvation results in a higher value of $\Delta G_{\mathrm{rxn}}$, as represented in Table 1. In summary, implicit and explicit solvation represents the experimental results adequately. Therefore, in the following section, we will consider both sets of results with and without the effect of explicit water molecules to understand the role of microsolvation in the reaction mechanism of cisplatin with the nitrogenous bases adenine and guanine.

### 2.1. Monofunctional and Bifunctional Addition of DNA Bases to Cisplatin

**Figure 4:**
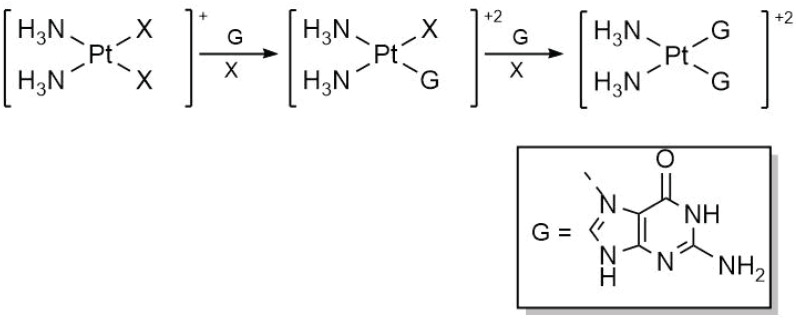
Mono- and bifunctionalization schemes of cisplatin with guanine.

Another significant step in the action pathway of cisplatin is the addition of nitrogenous bases. Table 2 presents the results concerning activation enthalpies for the first and second functionalizations of cisplatin with guanine, as schematized in Figure 4. Relevant effects occur with the inclusion of explicit water molecules for each of the additions of the nitrogenous bases. We found for the monofunctionalization of cisplatin that explicit microsolvation decreases the activation energy, while for the second functionalization, it presents the opposite effect. The M06-2X functional closely aligns with the experimental activation enthalpy for the first functionalization of cisplatin (18 ± 1 kcal/mol) [28] for both implicit and explicit solvation models. The experimental activation enthalpy for the subsequent addition of guanine is 21 ± 2 kcal/mol, a value that is accurately reproduced by the PBE0 functional with Grimme’s correction for dispersion and explicit solvation water molecules. Still, the M06-2X functional with explicit solvation remains competitive in modeling the bifunctionalization reaction of guanine, failing just by 1 kcal/mol in the superior limit of the experimental data, and it yields significantly better results regarding the first functionalization of cisplatin. Thus, the remainder of the discussion will focus exclusively on the results obtained using the M06-2X functional.

### 2.2. Energetics for the Reaction of Cisplatin with the Bases Adenine and Guanine

**Figure 5:**
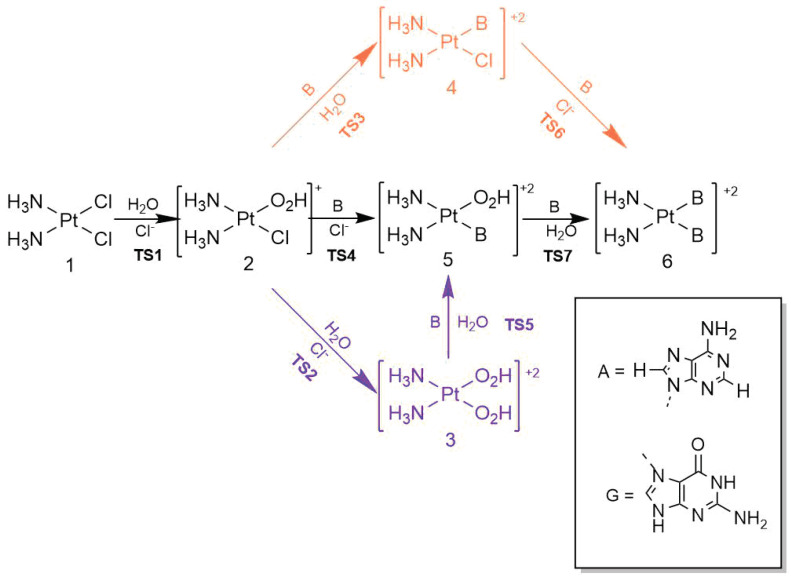
Reaction mechanism of cisplatin hydration (with transition states TS1, TS2), as well as the addition of adenine (TS3A–TS7A) and (TS3G–TS7G), for which B=A and B=G, respectively.

In this section, we will examine the biological addition of adenine and guanine to cisplatin (Figure 5) with implicit and explicit solvation effects. More specifically, we will analyze in detail each of the reaction steps for the addition of these nitrogenous bases to cisplatin, along with the corresponding changes in their electronic activation energies due to explicit microhydration. As shown in Figure 5, the mechanisms with guanine and adenine share certain steps, viz., the hydration of cisplatin (TS1, TS2). The mechanism involving the addition of adenine consists of transition states TS3A–TS7A. The same goes for the addition of guanine and transition states TS3G–TS7G. The profiles corresponding to the Gibbs free activation energies exhibit the same trends as the activation electronic energies (see Figures S1–S24 in the Supporting Information). These figures also reveal that the effects of solvation are rather mild in the general landscape of these profiles, with the exception of the electronic energy graph for transformation 2 → 4G → 6G computed with the M06-2X functional along with Basis Set 1, as shown in Figure S4. We note that the inclusion of explicit water molecules (i) dramatically increases the corresponding activation energies and (ii) changes the process from endothermic to exothermic. The examination of the involved structures revealed that the solvation water molecules interact with the entering and leaving groups of both substitution reactions. We also noted that the corresponding Gibbs free energy profiles with and without surrounding water molecules are more similar to one another (Figure S16). Still, the incorporation of water molecules considerably increases the Gibbs activation free energies of reactions 2 → 4G → 6G and tends to make the process of 2 → 4G more exergonic with approximation M06-2X/Basis Set 1.

Given the lack of a direct relationship between (i) the respective quality and nucleophilicity of the leaving and entering groups and (ii) changes in the activation energies, we ruled out that the entering or leaving group determines the variations in activation energies. As mentioned above, a preliminary examination of interatomic distances led us to realize that in cases where the activation energy increases due to the contemplation of explicit water molecules (see, e.g., TS4A and TS6G in Table 3), the apparent role of the surrounding water molecules is a greater stabilization in the reactants than in the corresponding transition states. The consideration of the differences in electronic activation energies,

(1) $$\Delta \Delta E^{‡}=\Delta E^{‡}\left(explicit\right)-\Delta E^{‡}\left(implicit\right),$$

in Table 3 shows that the explicit consideration of solvation water molecules increases the activation energy of the reaction steps overall, as considered in Figure 5, with a few exceptions. These results are in direct opposition to the conclusions offered by Cossio et al. [19]. The lower and upper limits of $\Delta \Delta E^{‡}$ are −4.58 and 15.07 kcal/mol. For further wave function analyses, we considered the reactants and transition states of the steps for which $|\Delta \Delta E^{‡}|>$ 3 kcal/mol, i.e., the elementary reactions for which the incorporation of explicit water solvation molecules is most conspicuous.

### 2.3. QTAIM and IQA Wave Function Analyses

In order to better understand the effect of the surrounding water molecules on the activation energies of the above-mentioned reaction steps, we determined the IQA interaction energy of the solvation water molecules with the rest of the electronic system $E_{\mathrm{int}}^{H_{2}O\cdots \mathrm{cisPt}}$ for both reactants and transition states, as defined in Equations (4)–(6) of Section 3. Table 4 reports these differences for these IQA interaction energies for the transition state and the corresponding reactants:

(2) $$\Delta E_{\mathrm{int}}^{H_{2}O\cdots \mathrm{cisPt}}=E_{\mathrm{int}}^{H_{2}O\cdots \mathrm{cisPt}}\left(\mathrm{TS}\right)-E_{\mathrm{int}}^{H_{2}O\cdots \mathrm{cisPt}}\left(reactants\right).$$

When $\Delta E_{\mathrm{int}}^{H_{2}O\cdots \mathrm{cisPt}}>0$, then the surrounding water molecules stabilize the reactants more strongly than the transition state; therefore, we would expect that the encompassing H_2_O monomers increase the activation energy of the reaction under consideration, i.e., $\Delta \Delta E^{‡}>0$. Correspondingly, when $\Delta E_{\mathrm{int}}^{H_{2}O\cdots \mathrm{cisPt}}<0$, then the encircling water molecules stabilize the transition state more substantially than the reactants, and hence, the solvation H_2_O monomers conduce to a reduction in the activation energy, viz., $\Delta \Delta E^{‡}<0$. There is indeed a correspondence between the signs of $\Delta \Delta E^{‡}$ and $\Delta E_{\mathrm{int}}^{H_{2}O\cdots \mathrm{cisPt}}$ for all of the systems shown in Table 4, apart from TS3. We mention that IQA interaction energies should not be confused with formation energies, $\Delta E_{\mathrm{form}}$ of a molecular cluster, G⋯H⋯I in the process

(3) $$G+H+I+\cdots ⇌G\cdots H\cdots I\cdots \;\Delta E_{\mathrm{form}}.$$

Indeed, one must take into account the IQA deformation energies of the interacting monomers $E_{\mathrm{def}}^{G}$ for the computation of $\Delta E_{\mathrm{form}}$ [30], i.e.,

(4) $$\Delta E_{\mathrm{form}}=\sum_{G}E_{\mathrm{def}}^{G}+\frac{1}{2}\sum_{G}\sum_{G\neq H}E_{\mathrm{int}}^{\mathrm{GH}}$$

Still, the IQA interaction energy is a good indicator of the formation energy, viz., large values of $|\Delta E_{\mathrm{form}}|$ are usually related with substantial magnitudes of $E_{\mathrm{int}}^{\mathrm{GH}}$.

**Figure 6:**
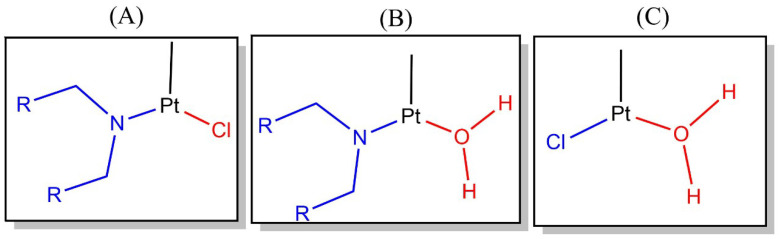
Representation of three different skeletons (**A**–**C**) displaying the entering and leaving groups of the investigated reaction in the ligands at equatorial positions in the geometric arrangement at every transition state. The entering and leaving groups are drawn in blue and red colors, respectively. The labels (**A**–**C**) are used in Table 5.

Finally, to gain further insights into the addition of nitrogenous bases to cisplatin, we used QTAIM delocalization indices as chemical bond descriptors. We proceeded in this way to describe the changes in the bond formation from reactants to transition states. There is a displacement of bonded species with the arrival of a nucleophilic ligand in substitution reactions of square planar complexes. The substitution takes place via an associative mechanism in which the nucleophilic ligand first binds to the metal center, forming a trigonal bipyramidal structure in the transition state. The leaving group is subsequently replaced by the entering nucleophile. More concretely, the entering and leaving species are in equatorial positions throughout the geometric rearrangement of the transition state, and the spectator ligands do not affect the direct substitution of the reaction. In the case of the reaction of cisplatin with nitrogenous bases of DNA, the entering group will always be the nitrogenous base, and the leaving group will be chloride, water, or a second nitrogenous base depending on the reaction step being analyzed. Table 5 shows the variations in the values of DIs for the leaving and entering groups involved in the formation and breaking of chemical bonds of each transition state with explicit and implicit solvation models for the structures shown in Figure 6. The corresponding changes in delocalization indices are

(5) $$\Delta {\mathrm{DI}}^{X}=\mathrm{DI}{\left(\mathrm{Pt},X\right)}^{\mathrm{TS}}-\mathrm{DI}{\left(\mathrm{Pt},X\right)}^{\mathrm{reactant}},$$

wherein X is an entering or leaving group, as shown in Figure 6.

The greater the absolute value of $\Delta {\mathrm{DI}}^{X}$, the greater the change in the chemical bonding of the Pt–X interaction. As expected, $\Delta {\mathrm{DI}}^{X}$ for the entering and leaving groups is positive and negative, respectively (Table 5). The addition of ΔDI for the leaving and the entering groups,

(6) $$\sum_{X}{\mathrm{DI}}^{X}=\Delta {\mathrm{DI}}^{\mathrm{entering}}+\Delta {\mathrm{DI}}^{\mathrm{leaving}},$$

represents a measure of the effects of the rupture and the formation of chemical bonds from the reactants to the transition state. Table 6 reports the values of $\sum_{X}\Delta {\mathrm{DI}}^{X}$ for the implicit and explicit solvation models addressed herein. Because

- the breaking of chemical bonds involves an energy cost, while the formation of these interactions releases energy to the surroundings, and
- the energy of the transition states is higher than that of the reactants in every case (all values of $\Delta E^{‡}>0$ in Table 3),

we expect the effects of the rupture of chemical bonds to be more important than those of the formation of these contacts. In other words, we anticipate that $\sum_{X}\Delta {\mathrm{DI}}^{X}<0$ since $\Delta {\mathrm{DI}}^{X}$ is positive for the entering group and negative for the leaving group, where the latter influence is more relevant, as mentioned above. Table 6 shows that this is indeed the case. The difference

(7) $$\Delta \Delta \mathrm{DI}\left(\mathrm{Pt}\right)=\sum_{X}\Delta {\mathrm{DI}}^{X}\left(\mathrm{expl}\right)-\sum_{X}\Delta {\mathrm{DI}}^{X}\left(\mathrm{impl}\right),$$

indicates whether the effects of the rupture of chemical bonds are more important for the explicit (ΔΔDI(Pt)<0) or the implicit (ΔΔDI(Pt)>0) solvation model. Apart from TS1, which is the transition state with the smallest value of $|\Delta \Delta E^{‡}|$ for which we carried out QCT analyses, we note that for transition states wherein ΔΔDI(Pt)<0, it holds that $\Delta \Delta E^{‡}>0$. Indeed, the inclusion of explicit solvation water molecules makes the effect of rupture of chemical bonding from reactants to the transition state more stringent, and, hence, a higher cost in activation energy is observed. We note the opposite behavior when ΔΔDI(Pt)>0. These observations are consistent with Hammond’s postulate: *“structures close in energy that transform directly into each other are also similar in structure”* [31]. In other words, when the inclusion of explicit solvation water molecules results in a more/less drastic change in the chemical bonding scenario, we expect a rise/diminution in activation energy with respect to implicit solvation models.

## 3. Theoretical Framework

In this section, we briefly discuss the QCT methods used in this work, namely, the QTAIM and IQA methods of wave function analyses. The QTAIM approach leads to a partition of the 3D space into disjointed regions $\Omega_{A}$, $\Omega_{B}$, … identified with the atoms of chemistry [32]. The QTAIM defines expectation values of quantum-mechanical observables within these atomic basins. These observables include energy and multipole moments. For example, the QTAIM atomic charge is defined as

(8) $$Q^{\Omega_{A}}=Z^{\Omega_{A}}-\int_{\Omega_{A}}\rho \left(r\right)dr,$$

wherein $Z^{\Omega_{A}}$ is the atomic number of the nucleus inside $\Omega_{A}$, ρ(r) is the electronic charge distribution, and $\int_{\Omega_{A}}\rho \left(r\right)dr$ is the expectation value of the number of electrons within $\Omega_{A}$. Topological analyses of the electronic density can be performed in terms of critical points of ρ(r), i.e., points where ∇ρ vanishes. The QTAIM allows the computation of the number of electrons shared between two atoms, denoted as the delocalization index (DI):

(9) $$\mathrm{DI}\left(\Omega_{A},\Omega_{B}\right)=-2\mathrm{cov}\left(N\left(\Omega_{A}\right),N\left(\Omega_{B}\right)\right),$$

in which $\mathrm{cov}\left(N\left(\Omega_{A}\right),N\left(\Omega_{B}\right)\right)$ is the covariance between the expectation value of the number of electrons in $\Omega_{A}$ and $\Omega_{B}$. The value of $\mathrm{DI}\left(\Omega_{A},\Omega_{B}\right)$ yields a measure of the exchange of electrons between $\Omega_{A}$ and $\Omega_{B}$, the distinctive feature of covalency.

On the other hand, the IQA energy partition can be used to divide the electronic energy of a system into net ($E_{\mathrm{net}\;}^{\Omega_{A}}$) and interatomic ($E_{\mathrm{int}}^{\Omega_{A}\Omega_{B}}$) contributions:

(10) $$E=\sum_{A}E_{\mathrm{net}\;}^{\Omega_{A}}+\sum_{A}\sum_{B>A}E_{\mathrm{int}\;}^{\Omega_{A}\Omega_{B}}.$$

The quantity $E_{\mathrm{net}\;}^{\Omega_{A}}$ is the net energy of atom A, while the value of $E_{\mathrm{int}\;}^{\Omega_{A}\Omega_{B}}$ is the interaction energy between atoms $\Omega_{A}$ and $\Omega_{B}$. In turn, we can split the IQA interaction energy into a classical ($E_{\mathrm{cl}}^{\Omega_{A}\Omega_{B}}$) and an exchange-correlation ($E_{\mathrm{xc}}^{\Omega_{A}\Omega_{B}}$) component, i.e.,

(11) $$E_{\mathrm{int}}^{\Omega_{A}\Omega_{B}}=E_{\mathrm{cl}}^{\Omega_{A}\Omega_{B}}+E_{\mathrm{xc}}^{\Omega_{A}\Omega_{B}},$$

wherein $E_{\mathrm{cl}}^{\Omega_{A}\Omega_{B}}$ and $E_{\mathrm{xc}}^{\Omega_{A}\Omega_{B}}$ are, respectively, related to ionic and covalent contributions to the chemical bond between basins $\Omega_{A}$ and $\Omega_{B}$. These quantities can be approximated using QTAIM charges and DIs [33] as follows:

(12) $$\begin{array}{cc} E_{\mathrm{cl}}^{\Omega_{A}\Omega_{B}} & \approx \frac{Q^{\Omega_{A}}Q^{\Omega_{B}}}{R}, \end{array}$$

(13) $$\begin{array}{cc} E_{\mathrm{xc}}^{\Omega_{A}\Omega_{B}} & \approx -\frac{\mathrm{DI}\left(\Omega_{A},\Omega_{B}\right)}{2R}, \end{array}$$

in which *R* is the distance between the nuclei within $\Omega_{A}$ and $\Omega_{B}$.

## 4. Computational Details

We performed geometric optimizations of all Pt complexes involved in the cisplatin reaction mechanism using both the M06-2X [34] and PBE0 [35] exchange-correlation functionals as implemented in the Orca 5.0.3 program package [36,37,38]. For these electronic structure calculations, we used the def2-SVP basis set for all the main-group elements and the def2-TZVP basis set for platinum (hereafter referred to as Basis Set 1), along with a Stuttgart–Dresden relativistic effective core potential for the Pt center [39]. To identify the most suitable exchange-correlation functional to carry out the QCT computations, we benchmarked the first hydration step of cisplatin using the more extensive def2-QZVP basis set (Basis Set 2) developed by Ahlrichs [29]. The PBE0 functional was used together with Grimme’s D3 dispersion correction [40,41], as this combination accurately accounts for non-covalent interactions such as hydrogen bonding between different molecular strands, which are critical factors in the second addition of nitrogenous bases to cisplatin. Moreover, PBE0+D3 reliably captures the structure and energetics of heavy metal complexes [42], while M06-2X agrees very well with experimental results for the hydration steps of cisplatin [27,28]. In addition, the last mentioned exchange-correlation functional is particularly accurate in predicting energy barriers across reaction mechanisms [43]. Notably, both functionals yielded similar trends throughout the reaction pathway involving (i) cisplatin and (ii) adenine or guanine. We emphasize that all calculations accounted for solvent effects via the conductor-like polarizable continuum model (C- PCM) [44]. We proceeded in this way so that we could better assess the effect of the surrounding water molecules on the reactive system. We computed numerical vibrational frequencies for all of the reactants, transition states, and products addressed in this investigation. Both reactants and products have no imaginary frequencies. On the other hand, transition states have only one imaginary frequency whose associated normal mode connects the corresponding reactants and products.

In order to generate suitable electron densities for QTAIM analyses, we performed single-point calculations for every minimum and transition state using the zero-order regular approximation (ZORA) [45]. These electronic structure computations were carried out with relativistically contracted orbital and auxiliary basis sets [46]. Finally, QTAIM analyses [32] were performed using the Aimall program [47] and by considering electron densities computed with the PBE0 approximation. We did not consider electronic charge distributions calculated with the M06-2X functional due to the well-known shortcomings and limitations of this electronic structure method for the computation of these scalar fields [48].

## 5. Conclusions

We examined the reaction mechanism of cisplatin with guanine and adenine, emphasizing the impact of microsolvation on activation energies, which are shown to closely match reported experimental values. Our findings highlight that the presence of explicit water molecules can significantly influence activation energies by preferentially stabilizing either the reactants or the transition state. State-of-the-art methods of wave function analyses, namely, the QTAIM and IQA approaches, have been instrumental in elucidating the role of microsolvation by providing detailed insights into the interaction energies and changes in the chemical bonding landscape of reactants and transition states. Overall, this research enhances our understanding of how explicit solvation shapes the biological mechanism of cisplatin, demonstrating that microsolvation can modulate activation barriers through a complex interplay of structural and energetic factors, consistently with Hammond’s postulate.

## Figures and Tables

**Table 1:** Electronic ($\Delta E^{‡}$) and Gibbs free ($\Delta G^{‡}$) activation energies for the first hydration of cisplatin using different exchange-correlation functionals along with Basis Set 1 defined in the Section 4. The quantity $\Delta G_{\mathrm{rxn}}$ denotes the computed value for the change in Gibbs free energy for the overall reaction. The asterisk indicates that we performed these calculations using Basis Set 2 [29].

Level of Theory	$\Delta G^{‡}$ (kcal/mol)	$\Delta E^{‡}$ (kcal/mol)	$\Delta G_{\mathrm{rxn}}$ (kcal/mol)
PBE0-D3BJ implicit solv.	24.20	24.25	6.95
PBE0-D3BJ explicit solv.	26.02	26.77	5.12
M06-2X implicit solv.	20.57	19.81	5.98
M06-2X explicit solv.	22.04	23.28	3.51
M06-2X implicit solv. *	21.56	19.85	5.24

**Table 2:** Activation enthalpies ($\Delta H^{‡}$) for the mono- and bifunctionalization of cisplatin with guanine as schematized in Figure 4 and computed with different approximations.

**Monofunctionalization of cisplatin with guanine**			
**Level of Theory**	**Solvation Type**	**$\Delta H^{‡}$ (kcal/mol)**	**$\Delta H_{\exp}^{‡}$ (kcal/mol)**
PBE0-D3BJ	Implicit Solv.	24.17	18 ± 1 ^1^
PBE0-D3BJ	Explicit Solv.	23.91	18 ± 1 ^1^
M06-2X	Implicit Solv.	19.17	18 ± 1 ^1^
M06-2X	Explicit Solv.	18.07	18 ± 1 ^1^
**Bifunctionalization of cisplatin with guanine**			
**Level of Theory**	**Solvation Type**	**$\Delta H^{‡}$ (kcal/mol)**	**$\Delta H_{\exp}^{‡}$ (kcal/mol)**
PBE0-D3BJ	Implicit Solv.	16.97	21 ± 2 ^1^
PBE0-D3BJ	Explicit Solv.	21.01	21 ± 2 ^1^
M06-2X	Implicit Solv.	10.20	21 ± 2 ^1^
M06-2X	Explicit Solv.	24.30	21 ± 2 ^1^

^1^ Experimental results from Branfort et al. [28].

**Table 3:** Electronic activation energies along with the values of $\Delta \Delta E^{‡}$, as defined in Equation (7), for each step of the reaction of cisplatin with adenine and guanine, with implicit and explicit hydration effects. The identity of each transition state is indicated in Figure 5.

Transition State	$\Delta E^{‡}$ Implicit (kcal/mol)	$\Delta E^{‡}$ Explicit (kcal/mol)	$\Delta \Delta E^{‡}$ (kcal/mol)
TS1	19.81	23.28	**3.46**
TS2	18.89	18.79	−0.10
TS3A	16.70	12.12	**−4.58**
TS4A	20.66	32.24	**11.58**
TS5A	17.28	16.51	−0.77
TS6A	16.11	17.86	1.75
TS7A	16.21	16.40	0.20
TS3G	14.95	21.08	**6.13**
TS4G	20.00	18.40	−1.60
TS5G	14.55	12.17	−2.38
TS6G	10.10	25.17	**15.07**
TS7G	16.71	18.89	2.18

**Table 4:** IQA interaction energies $E_{\mathrm{int}}^{H_{2}O\cdots \mathrm{cisPt}}$ for reactants and transition states along with their corresponding differences defined in Equation (2). Atomic units are used throughout.

Reaction Step	$E_{\mathrm{int}}^{H_{2}O\cdots \mathrm{cisPt}}$ (Reactant)	$E_{\mathrm{int}}^{H_{2}O\cdots \mathrm{cisPt}}$ (Transition State)	$\Delta E_{\mathrm{int}}^{H_{2}O\cdots \mathrm{cisPt}}$
TS1	−0.1145	−0.0924	0.0221
TS3A	−0.1375	−0.1094	0.0280
TS3G	−0.1119	−0.0689	0.0430
TS4A	−0.2907	−0.2363	0.0544
TS6G	−0.1341	−0.0903	0.0438

**Table 5:** Changes in the QTAIM delocalization indices ($\Delta {\mathrm{DI}}^{X}$ as defined in Equation (9) for the entering (${\mathrm{TS}}^{\mathrm{ent}}$) and leaving (${\mathrm{TS}}^{\mathrm{leav}}$) groups of the atoms involved in the formation and rupture of chemical bonds under explicit ($\Delta {\mathrm{DI}}^{X}$ (expl)) and implicit ($\Delta {\mathrm{DI}}^{X}$ (impl)) solvation models. The structures referred to by labels (A), (B), and (C) and the entering and leaving groups are shown in Figure 6. Atomic units are used throughout.

(A)			(B)			(C)		
**TS**	**$\Delta {\mathrm{DI}}^{X}$ (expl)**	**$\Delta {\mathrm{DI}}^{X}$ (impl)**	**TS**	**$\Delta {\mathrm{DI}}^{X}$ (expl)**	**$\Delta {\mathrm{DI}}^{X}$ (impl)**	**TS**	**$\Delta {\mathrm{DI}}^{X}$ (expl)**	**$\Delta {\mathrm{DI}}^{X}$ (impl)**
${\mathrm{TS}6G}^{\mathrm{ent}}$	0.27	0.31	${\mathrm{TS}3G}^{\mathrm{ent}}$	0.27	0.27	${\mathrm{TS}1}^{\mathrm{ent}}$	0.26	0.24
${\mathrm{TS}6G}^{\mathrm{leav}}$	−0.31	−0.31	${\mathrm{TS}3G}^{\mathrm{leav}}$	−0.32	−0.30	${\mathrm{TS}1}^{\mathrm{leav}}$	−0.45	−0.46
${\mathrm{TS}4A}^{\mathrm{ent}}$	0.33	0.29	${\mathrm{TS}3A}^{\mathrm{ent}}$	0.29	0.27			
${\mathrm{TS}4A}^{\mathrm{leav}}$	−0.45	−0.38	${\mathrm{TS}3A}^{\mathrm{leav}}$	−0.33	−0.32			

**Table 6:** Values of $\sum_{X}\Delta {\mathrm{DI}}^{X}$ as defined in Equations (9) and (10) for the explicit and implicit solvation models considered herein. We report the quantity ΔΔDI(Pt) defined in Formula (7) as well. Atomic units are used throughout.

Reaction Step	$\sum_{X}\Delta {\mathrm{DI}}_{X}\;\mathrm{Explicit}$	$\sum_{X}\Delta {\mathrm{DI}}_{X}\;\mathrm{Implicit}$	ΔΔDI(Pt)
TS1	−0.19	−0.22	0.03
TS3A	−0.04	−0.05	0.01
TS3G	−0.05	−0.03	−0.02
TS4A	−0.12	−0.09	−0.03
TS6G	−0.04	0.00	−0.04

## Data Availability

Data are contained within the article and Supplementary Materials.

## Supplementary Materials

The following supporting information can be downloaded at: https://www.mdpi.com/article/10.3390/molecules30030510/s1, Figures S1–S24: Relative Electronic and Gibbs free energy profiles; Tables S1–S7: IQA interaction, classical and exchange-correlation energies.
