# Supplementary material for: Effect of Explicit Hydration on the Cisplatin Reaction Mechanism with Adenine and Guanine
Source: Molecules. 2025 Jan 23;30(3):510. doi: 10.3390/molecules30030510 (PMC11820302; doi:10.3390/molecules30030510)
Supplement: Supplementary file 1 [file molecules-30-00510-s001.zip › molecules-3414851-supplementary.pdf]

Effect of Explicit Hydration on the Cisplatin Reaction Mechanism with Adenine and Guanine

J. Iván Salazar-Barrientos[1], José Manuel Guevara-Vela[2], Marco A. García-Revilla[3], Miguel Gallegos[4], Evelio Francisco[4], Tomás Rocha-Rinza*[1], and Ángel Martín Pendás*[4]

[1]Instituto de Química, Universidad Nacional Autónoma de México, Circuito Exterior s/n, Ciudad Universitaria, Alcaldía Coyoacán, C.P. 04510, Cuidad de Mexico, Mexico
[2]Universidad Autónoma de Madrid, Departamento de Química Física Aplicada, C.P 28049, Madrid, Spain
[3]Universidad de Guanajuato, Departamento de Química, División de Ciencias Naturales y Exactas, Noria Alta, C.P 36050, Guanajuato, Mexico
[4]Departamento de Química Física y Analítica, Universidad de Oviedo, Av. Julián Clavería, 8, Oviedo 33006, Asturias, Spain

Supporting Information

*To whom correspondence should be addressed: trocha@iquimica.unam.mx, ampendas@uniovi.es

DLPNO- CCSD(T) single point calculations

Table S1: Electronic ($\Delta E^{\ddagger}$) and Gibbs free ($\Delta G^{\ddagger}$) activation energies for the first hydration of cisplatin computed with single point DLPNO/CCSD(T)/def2-TZVP calculations. The quantity ΔG_{rxn} denotes the computed value for the change of Gibbs free energy for the overall reaction.

**Structure**	$\Delta G^{\ddagger}$ (kcal/mol)	$\Delta E^{\ddagger}$ (kcal/mol)	ΔG_{rxn} (kcal/mol)
PBE0-D3BJ implicit solv.	25.45	25.51	5.74
PBE0-D3BJ explicit solv.	24.69	25.44	3.91
M06-2X implicit solv.	25.97	25.21	6.14
M06-2X explicit solv.	24.20	25.44	2.74

QTAIM and IQA wave function analyses

Table S2: IQA classical energy of the solvation water molecules with those species with which they share at least a bond path. Atomic units are used throughout.

**Reaction Step**	**Reactants**	**Transition State**
TS1	-2.6058	-2.6390
TS3A	-2.2991	-2.3782
TS3G	-2.3816	-2.3143
TS4A	-2.8722	-2.2507
TS6G	-2.3587	-2.1544

Table S3: IQA exchange-correlation energy of the solvation water molecules with those species with which they share at least a bond path. Atomic units are used throughout.

**Reaction Step**	**Reactants**	**Transition State**
TS1	-0.6517	-0.6468
TS3A	-0.6491	-0.6511
TS3G	-0.6647	-0.6580
TS4A	-0.6679	-0.6772
TS6G	-0.6508	-0.6238

Table S4: IQA interaction energy of the solvation water molecules with those species with which they share at least a bond path. Atomic units are used throughout.

**Reaction Step**	**Reactants**	**Transition State**
TS1	-3.2575	-3.2858
TS3A	-2.9482	-3.0293
TS3G	-3.0463	-2.9723
TS4A	-3.5401	-2.9279
TS6G	-3.0095	-2.7781

Table S5: IQA classical energy of the surrounding water molecules and the reactive system. Atomic units are used throughout.

**Reaction Step**	**Reactants**	**Transition State**
TS1	-0.0823	-0.0517
TS3A	-0.0712	-0.0557
TS3G	-0.0577	-0.0286
TS4A	-0.1467	-0.1240
TS6G	-0.0622	-0.0323

Table S6: IQA exchange-correlation energy of the surrounding water molecules and the reactive system. Atomic units are used throughout.

**Reaction Step**	**Reactants**	**Transition State**
TS1	−0.0710	−0.0639
TS3A	−0.0662	−0.0537
TS3G	−0.0541	−0.0403
TS4A	−0.1440	−0.1124
TS6G	−0.0719	−0.0580

Table S7: IQA interaction energy of the surrounding water molecules and the reactive system. Atomic units are used throughout.

**Reaction Step**	**Reactants**	**Transition State**
TS1	−0.1533	−0.1156
TS3A	−0.1375	−0.1094
TS3G	−0.1119	−0.0689
TS4A	−0.2907	−0.2363
TS6G	−0.1341	−0.0903

Relative Energy Diagrams

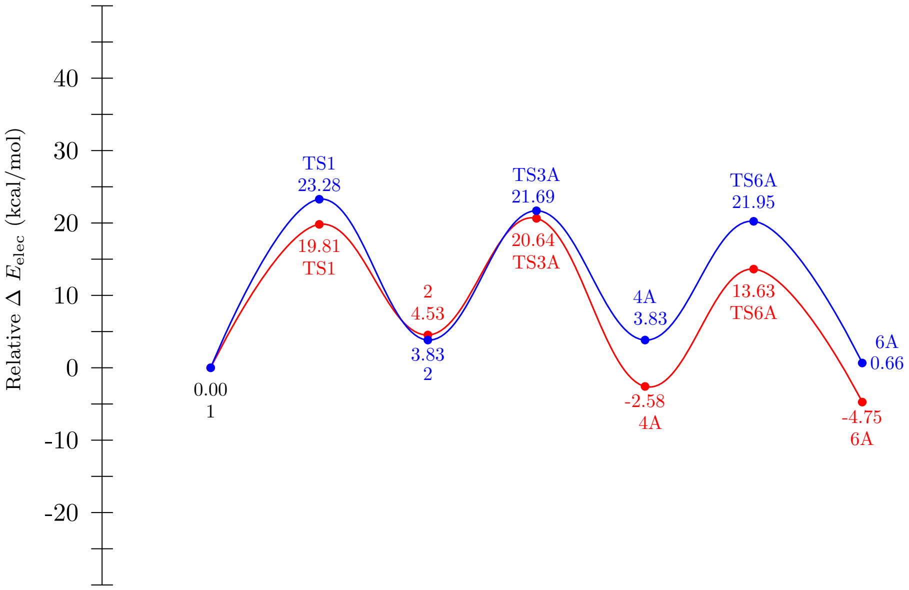

Figure S1: Relative electronic energy diagram of the reaction of 1 → 2 → 4A → 6A from **Figure 4** with (blue path) and without (red path) microsolvation computed with the approximation M06-2X along with Basis Set 1.

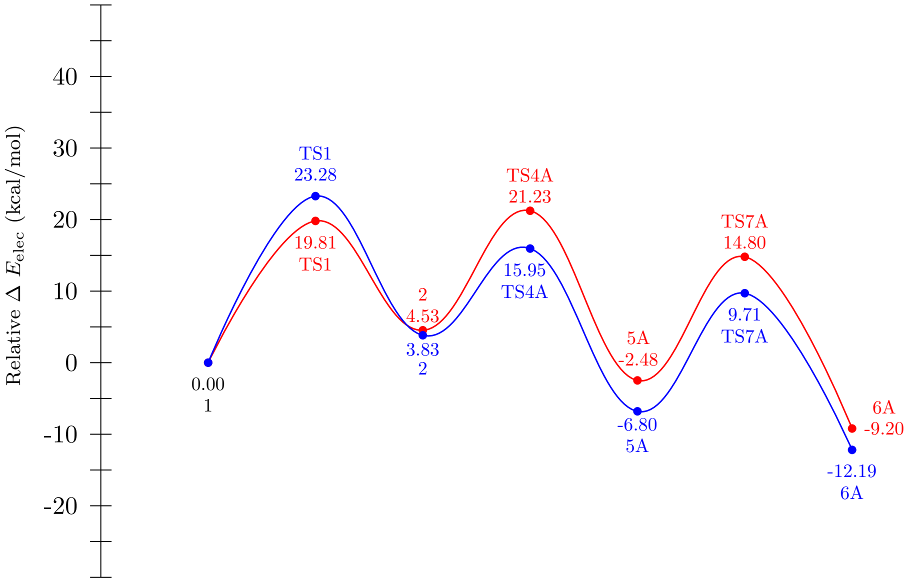

Figure S2: Relative electronic energy diagram of the reaction of 1 → 2 → 5A → 6A from **Figure 4** with (blue path) and without (red path) microsolvation computed with the approximation M06-2X along with Basis Set 1.

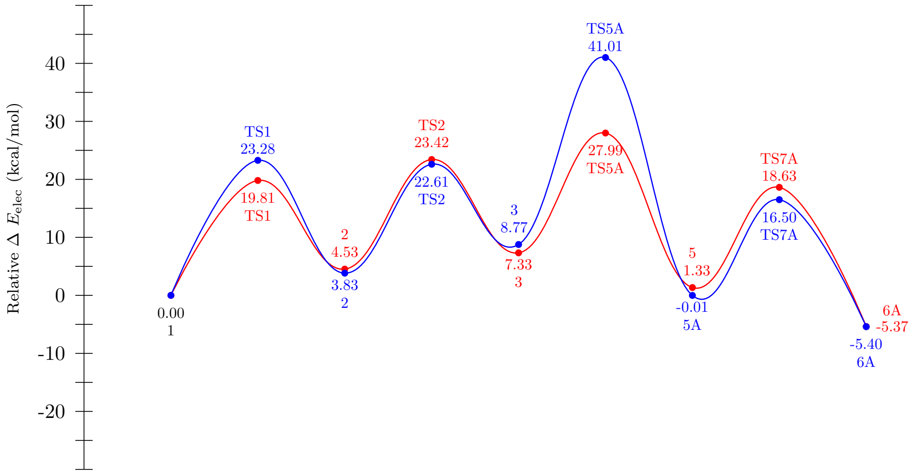

Figure S3: Relative electronic energy diagram of the reaction of 1 → 2 → 3 → 5A → 6A from **Figure 4** with (blue path) and without (red path) microsolvation computed with the approximation M06-2X along with Basis Set 1.

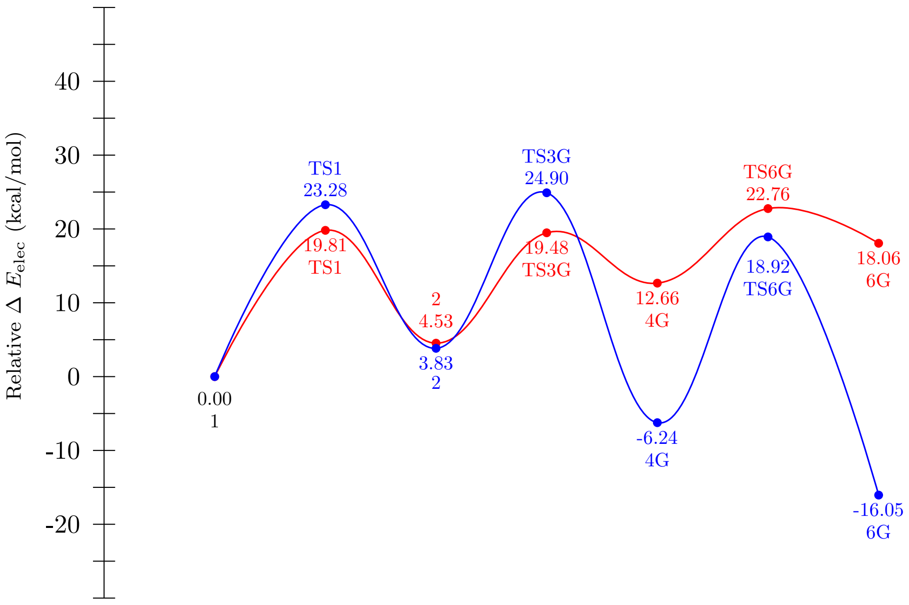

Figure S4: Relative electronic energy diagram of the reaction of 1 → 2 → 4G → 6G from **Figure 4** with (blue path) and without (red path) microsolvation computed with the approximation M06-2X along with Basis Set 1.

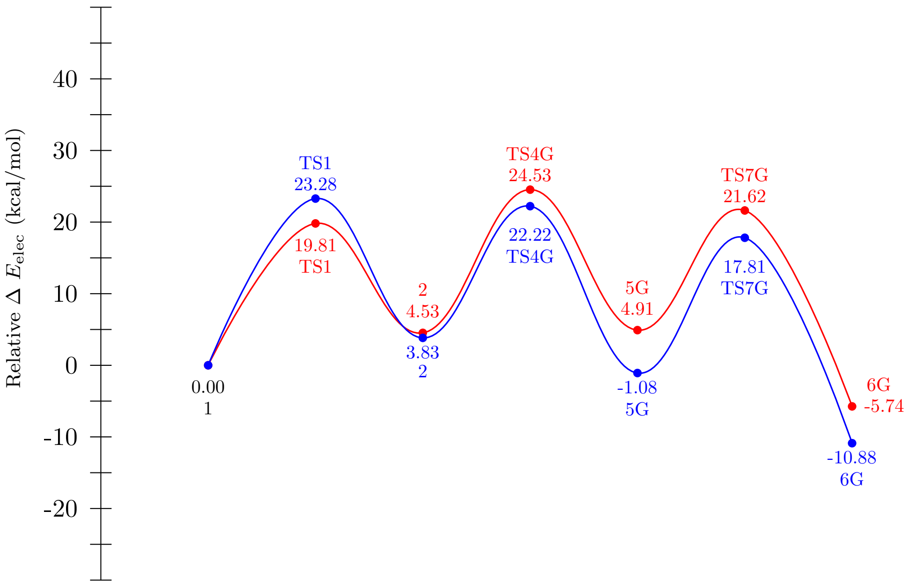

Figure S5: Relative electronic energy diagram of the reaction of 1 → 2 → 5G → 6G from **Figure 4** with (blue path) and without (red path) microsolvation computed with the approximation M06-2X along with Basis Set 1.

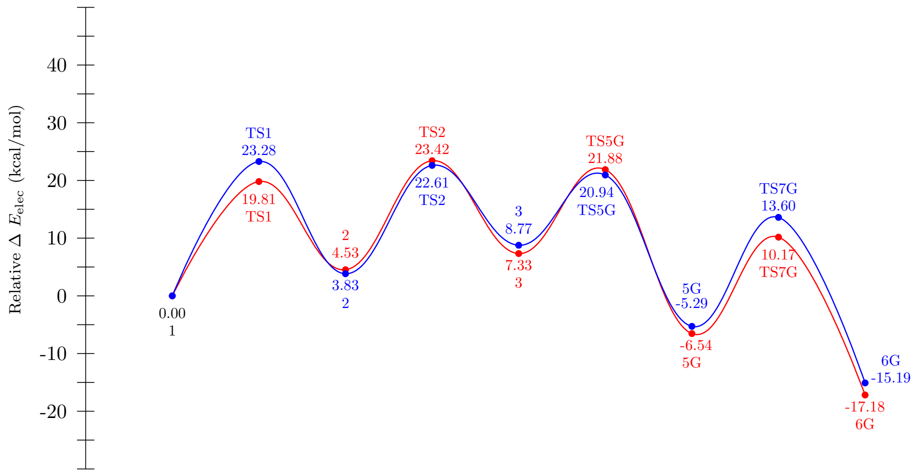

Figure S6: Relative electronic energy diagram of the reaction of 1 → 2 → 3 → 5G → 6G from **Figure 4** with (blue path) and without (red path) microsolvation computed with the approximation M06-2X along with Basis Set 1.

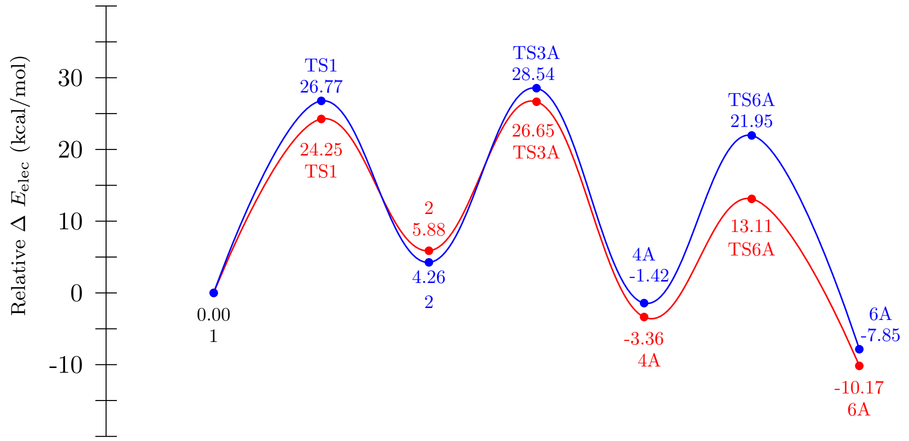

Figure S7: Relative electronic energy diagram of the reaction of 1 → 2 → 4A → 6A from **Figure 4** with (blue path) and without (red path) microsolvation computed with the approximation PBE0-D3BJ along with Basis Set 1.

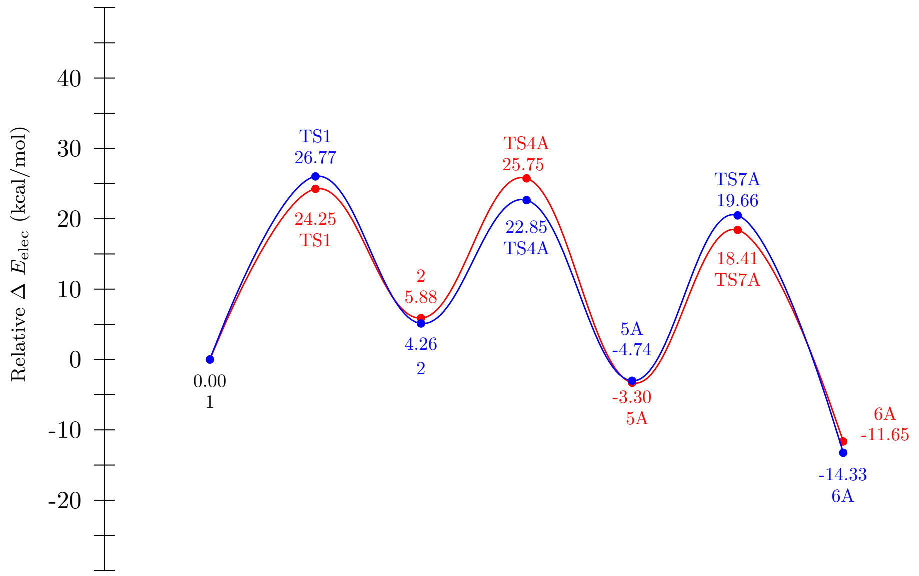

Figure S8: Relative electronic energy diagram of the reaction of 1 → 2 → 5A → 6A from **Figure 4** with (blue path) and without (red path) microsolvation computed with the approximation PBE0-D3BJ along with Basis Set 1.

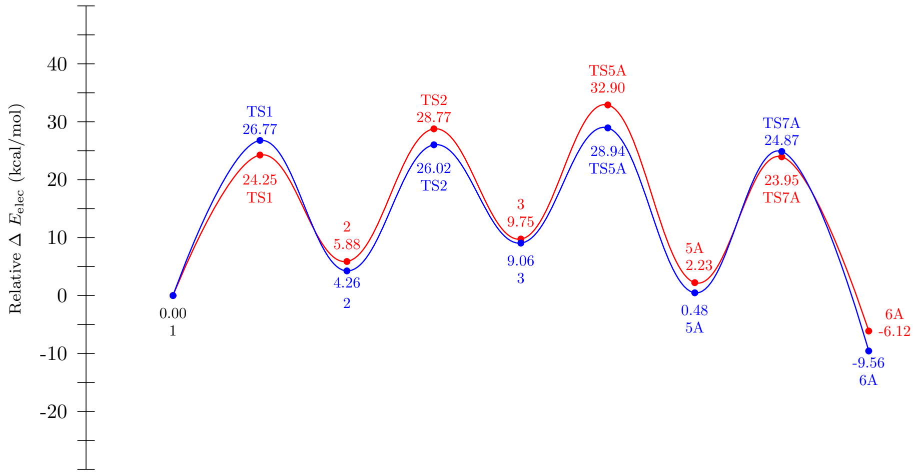

Figure S9: Relative electronic energy diagram of the reaction of 1 → 2 → 3 → 5A → 6A from **Figure 4** with (blue path) and without (red path) microsolvation computed with the approximation PBE0-D3BJ along with Basis Set 1.

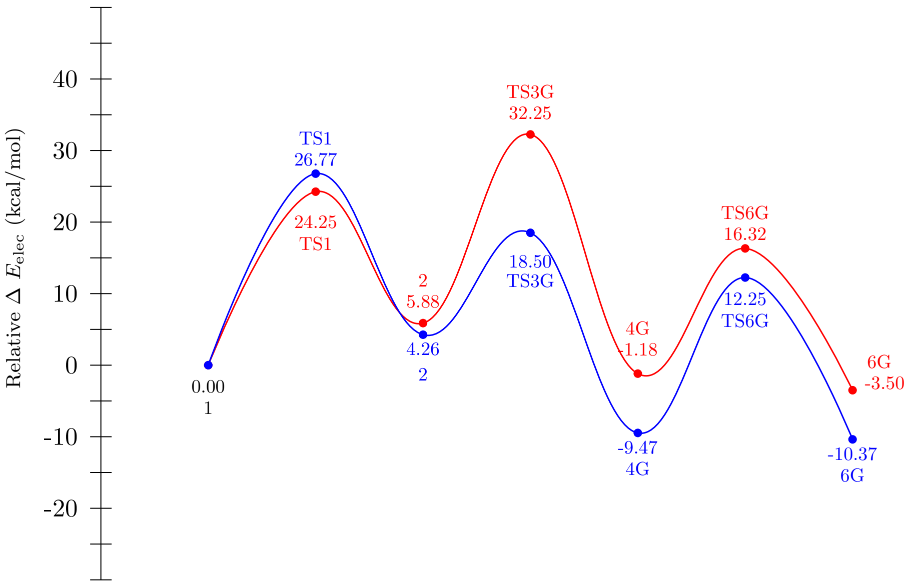

Figure S10: Relative electronic energy diagram of the reaction of 1 → 2 → 5G → 6G from **Figure 4** with (blue path) and without (red path) microsolvation computed with the approximation PBE0-D3BJ along with Basis Set 1.

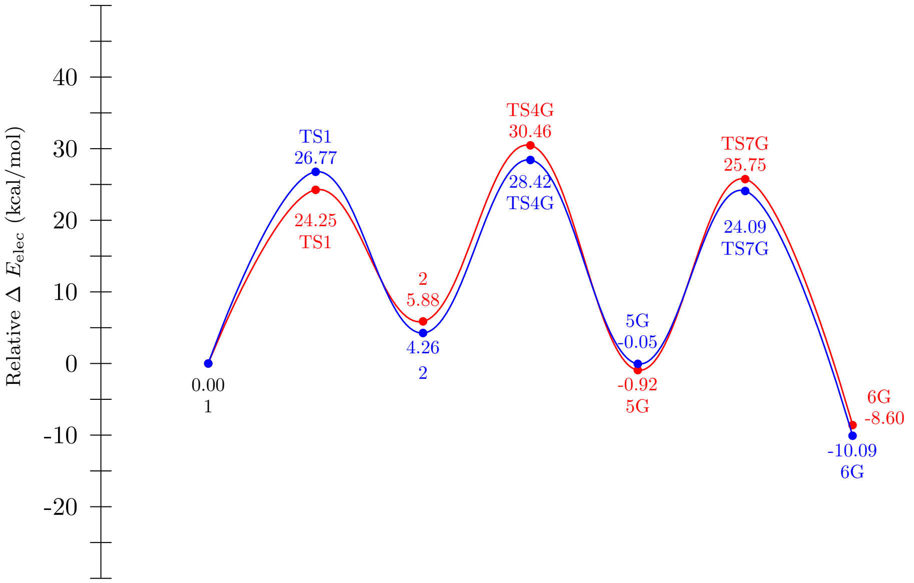

Figure S11: Relative electronic energy diagram of the reaction of 1 → 2 → 5G → 6G from **Scheme 2** with (blue path) and without (red path) microsolvation computed with the approximation PBE0-D3BJ along with Basis Set 1.

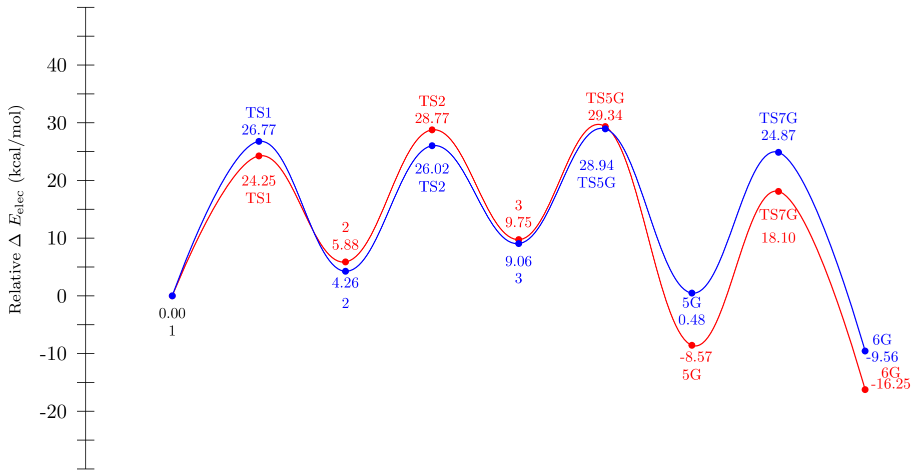

Figure S12: Relative electronic energy diagram of the reaction of 1 → 2 → 3 → 5G → 6G from **Figure 4** with (blue path) and without (red path) microsolvation computed with the approximation PBE0-D3BJ along with Basis Set 1.

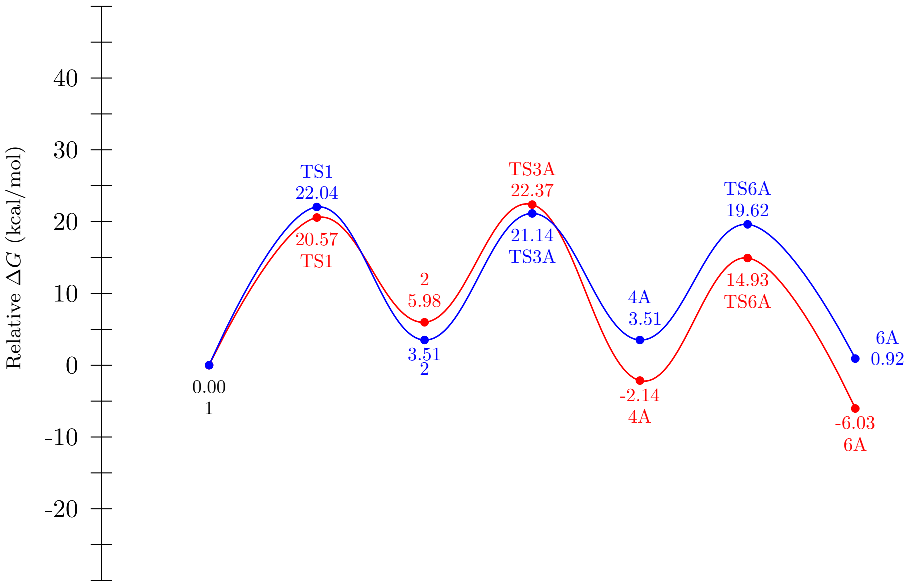

Figure S13: Relative Gibbs free energy diagram of the reaction of 1 → 2 → 4A → 6A from **Figure 4** with (blue path) and without (red path) microsolvation computed with the approximation M06-2X along with Basis Set 1.

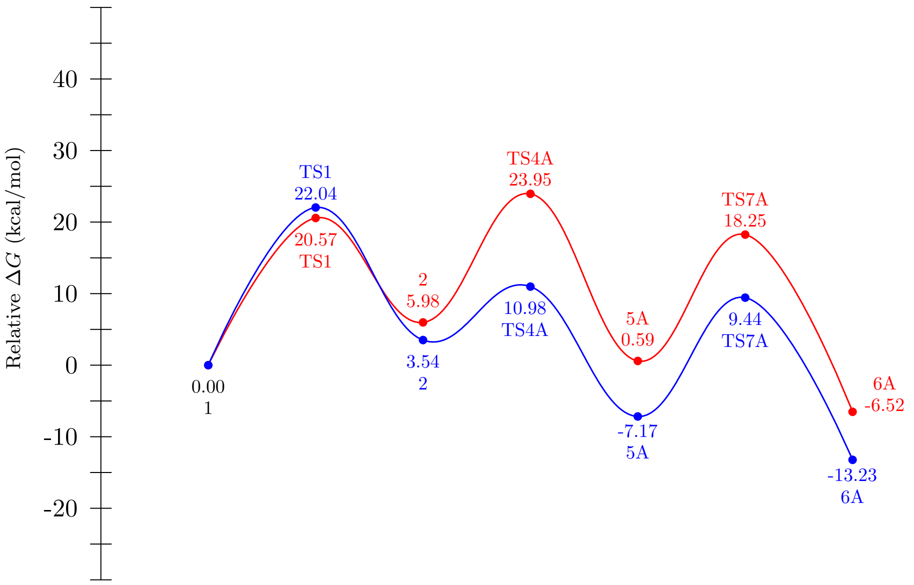

Figure S14: Relative Gibbs free energy diagram of the reaction of 1 → 2 → 5A → 6A from **Figure 4** with (blue path) and without (red path) microsolvation computed with the approximation M06-2X along with Basis Set 1.

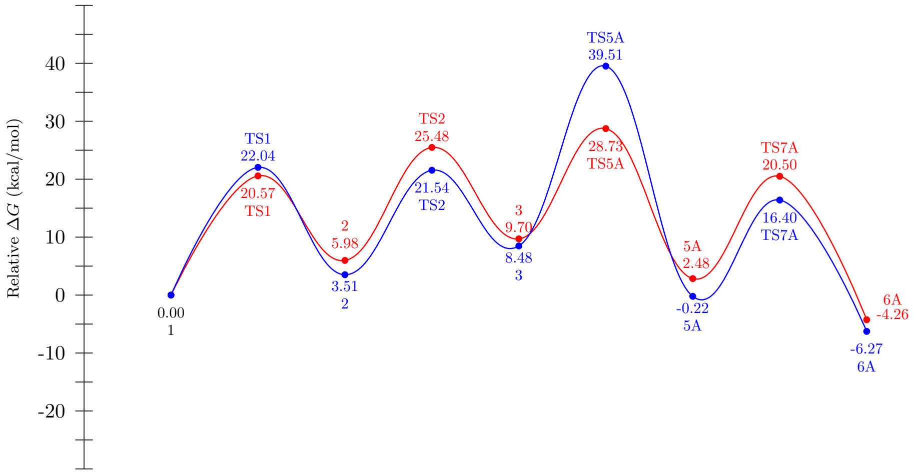

Figure S15: Relative Gibbs free energy diagram of the reaction of 1 → 2 → 3 → 5A → 6A from **Figure 4** with (blue path) and without (red path) microsolvation computed with the approximation M06-2X along with Basis Set 1.

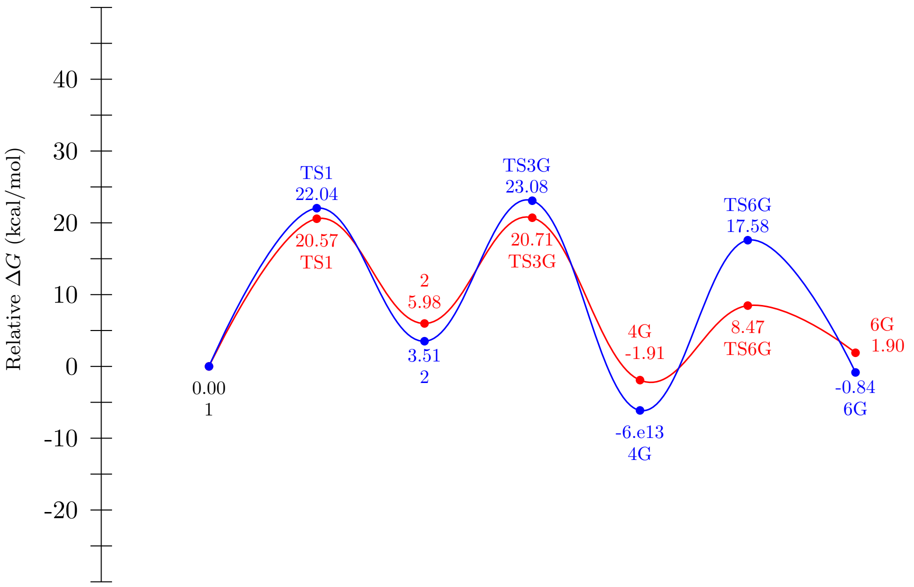

Figure S16: Relative Gibbs free energy diagram of the reaction of 1 → 2 → 4G → 6G from **Figure 4** with (blue path) and without (red path) microsolvation computed with the approximation M06-2X along with Basis Set 1.

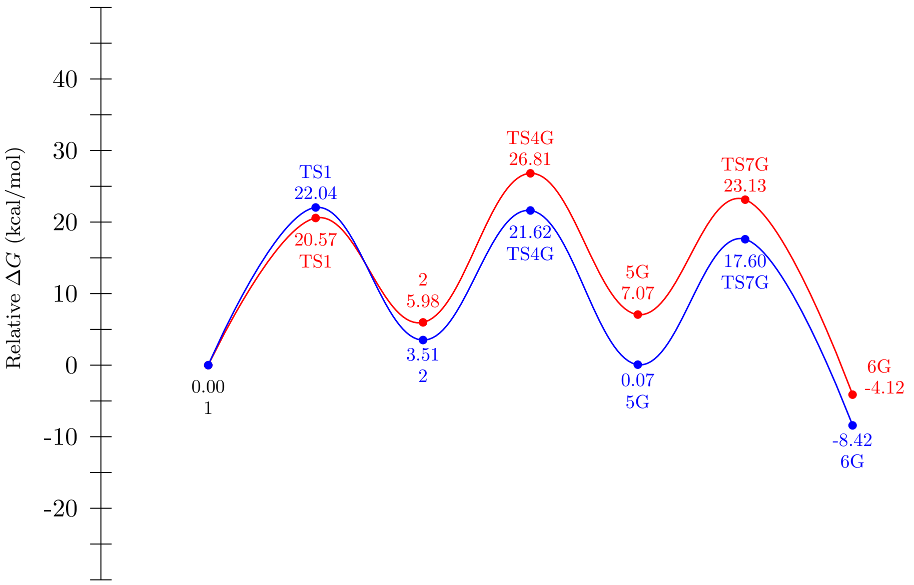

Figure S17: Relative Gibbs free energy diagram of the reaction of 1 → 2 → 5G → 6G from **Figure 4** with (blue path) and without (red path) microsolvation computed with the approximation M06-2X along with Basis Set 1.

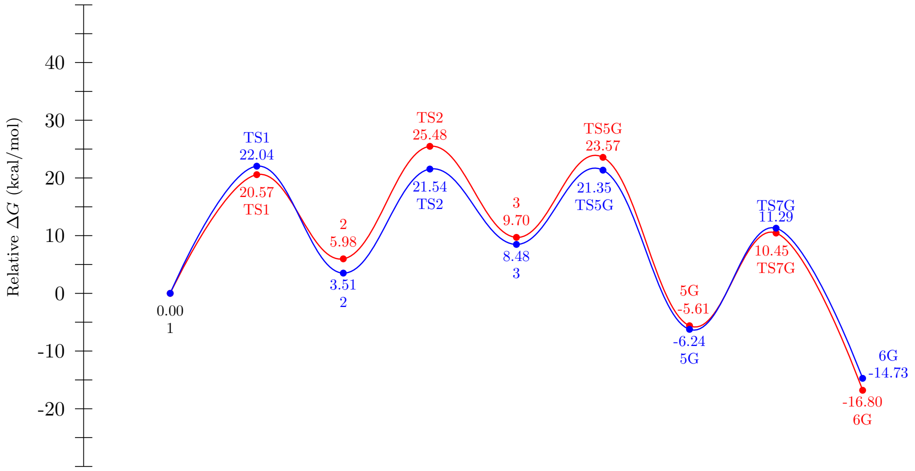

Figure S18: Relative Gibbs free energy diagram of the reaction of 1 → 2 → 3 → 5G → 6G from **Figure 4** with (blue path) and without (red path) microsolvation computed with the approximation M06-2X along with Basis Set 1.

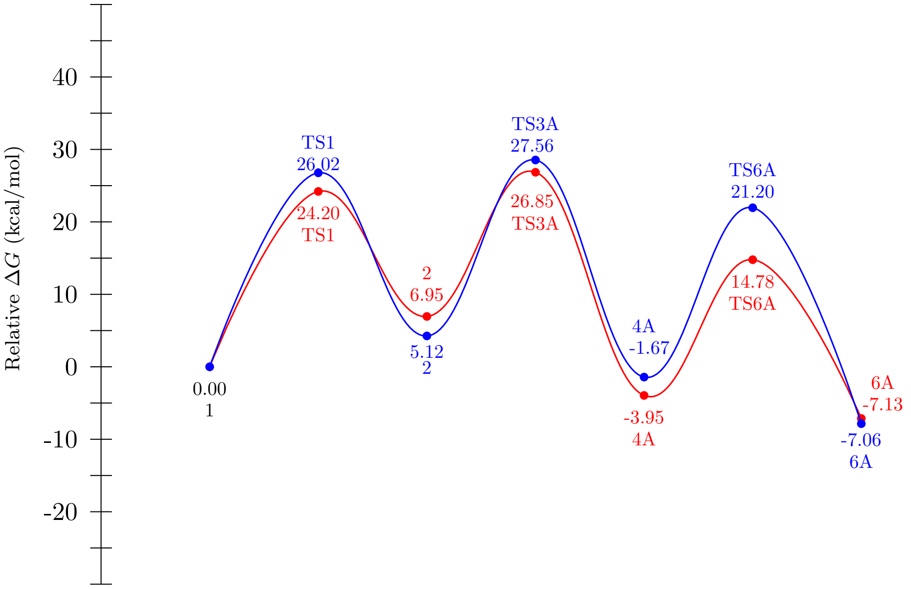

Figure S19: Relative Gibbs free energy diagram of the reaction of 1 → 2 → 4A → 6A from **Figure 4** with (blue path) and without (red path) microsolvation computed with the approximation PBE0-D3BJ along with Basis Set 1.

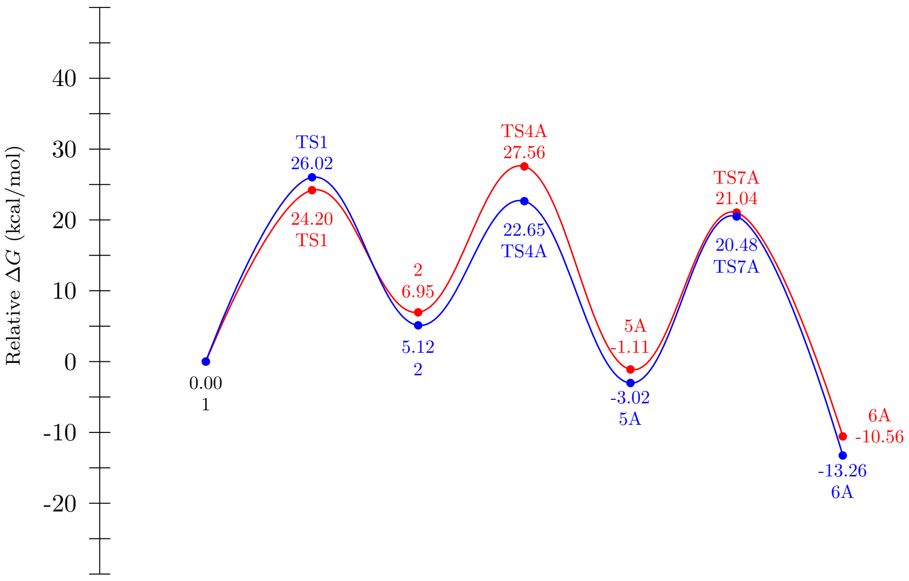

Figure S20: Relative Gibbs free energy diagram of the reaction of 1 → 2 → 5A → 6A from **Figure 4** with (blue path) and without (red path) microsolvation computed with the approximation PBE0-D3BJ along with Basis Set 1.

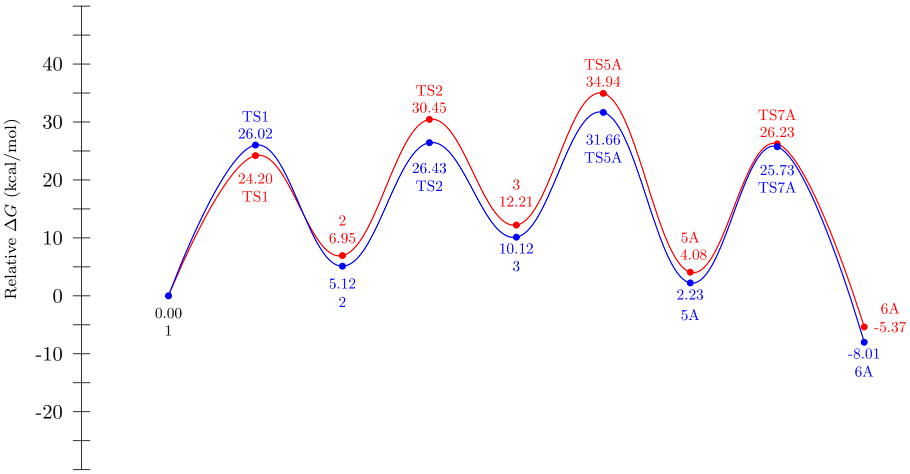

Figure S21: Relative Gibbs free energy diagram of the reaction of 1 → 2 → 3 → 5A → 6A from **Figure 4** with (blue path) and without (red path) microsolvation computed with the approximation PBE0-D3BJ along with Basis Set 1.

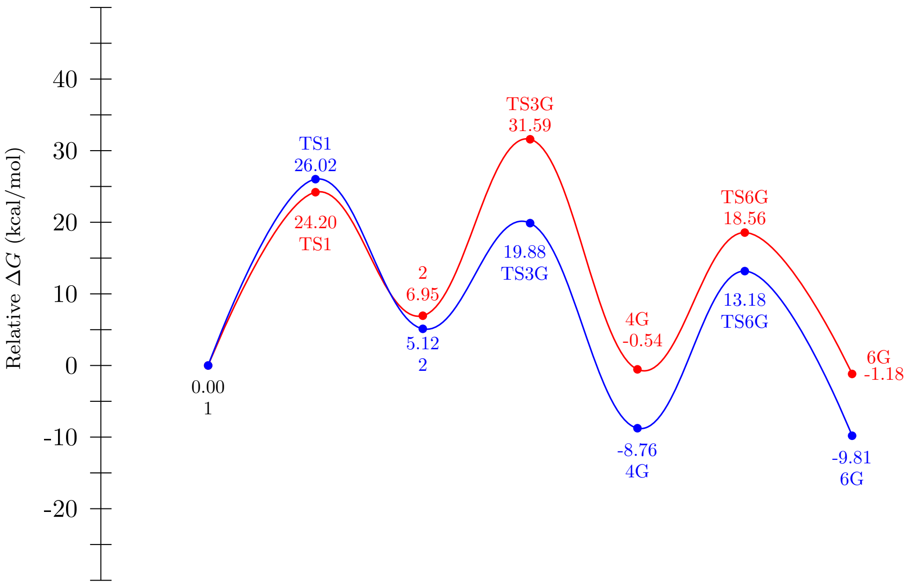

Figure S22: Relative Gibbs free energy diagram of the reaction of 1 → 2 → 4G → 6G from **Scheme 2** with (blue path) and without (red path) microsolvation computed with the approximation PBE0-D3BJ along with Basis Set 1.

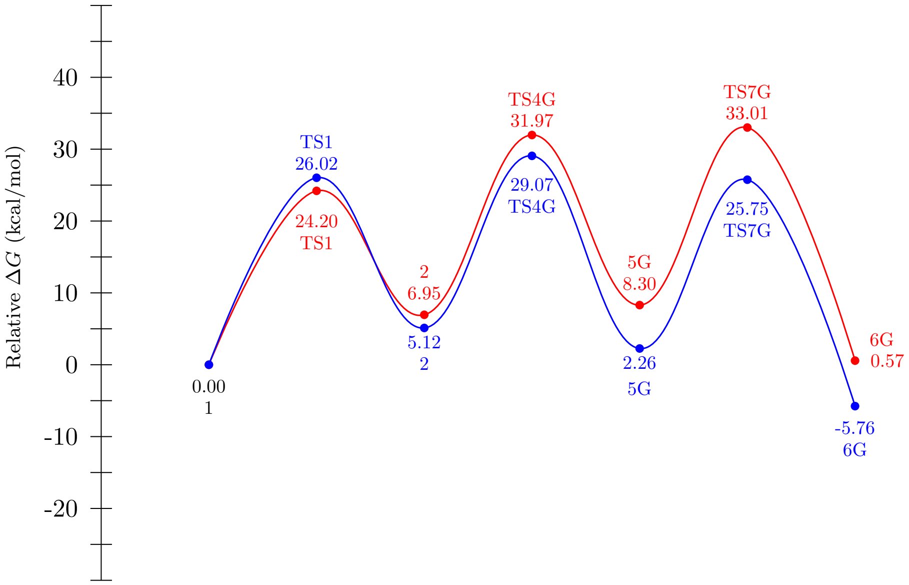

Figure S23: Relative Gibbs free energy diagram of the reaction of 1 → 2 → 5G → 6G from **Figure 4** with (blue path) and without (red path) microsolvation computed with the approximation PBE0-D3BJ along with Basis Set 1.

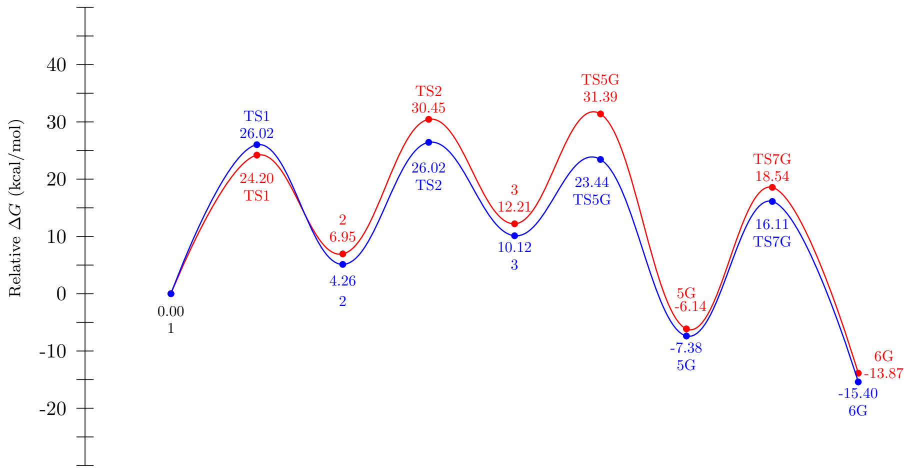

Figure S24: Relative Gibbs free energy diagram of the reaction of 1 → 2 → 3 → 5G → 6G from **Figure 4** with (blue path) and without (red path) microsolvation computed with the approximation PBE0-D3BJ along with Basis Set 1.

Cartesian coordinates of optimised geometries

Mechanism path without explicit solvation computed with the approximation M06-2X along with Basis Set 1.

Reactants for TS1

Pt	0.16030315824019	0.46730947836327	-0.85637906040144
Cl	1.25868761636759	2.27409902490784	-1.87130279481804
N	-0.77277164037539	-1.12331246504199	0.05350240851758
N	2.00471856383345	-0.39574653990534	-0.63567231537728
H	2.22330258370381	-0.63195593611567	0.33579743065220
H	2.72645965307193	0.25610264822824	-0.95719898184547
H	2.10999698074906	-1.25356129062774	-1.18395050838982
H	-1.20781605122987	-0.77679379701888	0.92128987831931
H	-0.15885809865247	-1.90501249906247	0.29203466365284
H	-1.52025072515765	-1.49660827300381	-0.53623127579290
Cl	-1.98473275752238	1.44746903166953	-1.09025220906197
O	-1.80576039162601	0.75158027161459	2.03675084826750
H	-0.98828645679084	1.20066429536627	2.29215615138697
H	-2.04499243461142	1.18578605062616	1.19945576489053

TS1

Pt	0.16806315965513	0.37809686741363	-0.16117225076812
Cl	1.11098519091531	2.51212819387074	-0.41908665598158
N	-0.69038433171756	-1.46366007728931	0.10061259883886
N	1.95679001472825	-0.43924094784767	-0.62516312450424
H	2.32687992252895	-1.02599069872605	0.12746394705233
H	2.64181443272399	0.30179690284796	-0.79996695556417
H	1.91153225152358	-1.01821723237855	-1.46795482376145
H	-0.68260029190177	-1.77095410361020	1.07661711278164
H	-0.27728686354720	-2.21633905470081	-0.45546934472152
H	-1.66852666916746	-1.36832911421269	-0.19479735846545
Cl	-2.43301362125540	0.87367523917701	-1.14784777752553
O	-1.34853645731554	1.43039362657517	1.54243757706853
H	-0.96824911487008	2.32116747370889	1.56584041051175
H	-2.04746762230019	1.48547292517187	0.85848664503894

Products for TS1

Pt	0.37031027584901	0.51968265174124	-0.12215698446892
Cl	1.60442972984789	2.44975150242634	0.31858664308890
N	-0.79705385662183	-1.13348787582490	-0.49550474609220
N	2.07164922462785	-0.56606317071013	-0.19348027810839
H	2.06945938106672	-1.33946183579292	0.47772978860086
H	2.87706951402643	0.02400618240142	0.03573651103776
H	2.24999606443685	-0.97086122321146	-1.11699635931062
H	-0.32388460504902	-2.03044418532343	-0.36435940165509
H	-1.16729769876423	-1.12911381446683	-1.44922166840373
H	-1.59917462507288	-1.10240416730932	0.15722948154326
Cl	-2.78702699578746	-0.08958826357900	1.82168432320114
O	-1.38629670884519	1.65327603994815	-0.03505463296740
H	-1.18643069851704	2.52860575874244	0.33559999845485
H	-1.99574800119710	1.18610240095841	0.63020832507959

Reactants for TS2

Pt	0.53494450580570	-0.13480591124346	-0.18900250376494
N	0.74576693219549	1.81255114773455	-0.64223883986793
N	2.34121760500935	-0.55522596005099	-1.07426098269214
H	2.30621030947254	-0.45832637419737	-2.09335574082370
H	2.64531992297970	-1.51434868157077	-0.88398184086870
H	3.09313271294325	0.05708768337907	-0.74477053965110
H	-0.16961861223578	2.22279139313354	-0.91184809601902
H	1.39748985923420	1.97945120959583	-1.41375952773016
H	1.09795298595047	2.34296837367038	0.16000316173483
Cl	-1.52547875993257	0.24865453679887	0.87719201540875
O	-1.94066241344738	2.64431899798663	-1.18245213254291
H	-2.21258879128956	1.98085193914967	-0.52612656192523
H	-2.10207719422614	3.48812418141498	-0.73808074500170
O	0.32566339850202	-2.18136626197303	0.30601071797130
H	0.19240172111696	-2.75496353020289	-0.46751061973655
H	-0.45660418207826	-2.32125274362502	0.86735223550921

TS2

Pt	0.08131695344010	-0.14305457685543	0.11571267609647
N	0.78870732875904	1.73148534038800	-0.00773733800749
N	1.80613930357729	-0.90280392793914	-0.61258101386380
H	1.74504527274328	-1.92198076878072	-0.69564416580666
H	2.60479241368876	-0.70187266661014	-0.00357331662758
H	2.03564704613835	-0.54008279490049	-1.54253239630361
H	-0.00470070579073	2.35525683301322	-0.19107810624816
H	1.47407997784052	1.88365671069888	-0.75293591483218
H	1.22435694386503	2.03707819371118	0.86787970337720
Cl	-1.51004462180348	-0.10199313498676	2.38812766245634
O	-2.00688212820286	1.11746058875820	-0.25224103852017
H	-2.59925001479986	0.75629486739258	-0.92724923270162
H	-2.41289750883580	0.89221946648565	0.60631932670395
O	-0.69030674128071	-2.09631529473298	0.30627650740704
H	-1.24522233993782	-2.00324253694154	1.11146595649100
H	-1.29078117940111	-2.36210629870050	-0.41020930962073

Products for TS2

Pt	-0.89875815438064	-0.05290690562164	-0.00443273761470
N	-2.34348509568255	1.35567110380251	-0.03690928756806
N	-2.25120519307882	-1.54532963408774	0.10322893675484
H	-2.74260070347919	-1.56531047422291	1.00194738478180
H	-1.78781908191525	-2.45321649188663	-0.00309609670733
H	-2.96703666996122	-1.49539748774336	-0.62828910067927
H	-1.93749764053844	2.28563477864849	-0.18021344854879
H	-2.87778808504869	1.39423278201363	0.83656631591908
H	-3.01757909161680	1.20742807270929	-0.79413464248895
Cl	2.35315903066022	-0.01941803599447	-1.85102923279540
O	0.50768218932698	1.48670780548187	-0.15412376580151
H	0.91688852610619	1.71008045157808	0.69808750060133
H	1.25375393115501	1.15352306231511	-0.74836551434023
O	0.59831616524777	-1.51127195876271	-0.03867330748000
H	1.32006934369242	-1.17881725558463	-0.66233601789559
H	1.02696852951306	-1.64737181264489	0.82260601386278

Reactants for TS3A

C	4.12547237455681	-1.13174281842321	0.06227414413461
N	3.77542203258949	-2.27802131884430	0.72890672240517
C	2.47064846380690	-2.16096575099562	1.08113547831876
N	1.94717742017352	-1.02228648847824	0.68893805580694
C	2.96655226735551	-0.35345993587870	0.04522082827385
Pt	-1.84607837966298	-0.21788083773736	-0.10523387860663
Cl	-1.29654350116689	2.03232500785021	-0.44227266735256
N	-2.30432772526830	-2.18997726187080	0.24352123459049
N	-3.22848632522343	-0.09529179923177	-1.57802514426221
H	4.38081101386898	-3.07038339828731	0.92694022198189
H	1.94342293131078	-2.94091240955925	1.62835566456652
H	-3.09504461616728	-0.79772736762703	-2.31120107660219
H	-3.18826906266822	0.82256094344424	-2.03089778330682
H	-4.18302185983575	-0.21266675787588	-1.22533524104667
H	-2.04294546527150	-2.80108300903234	-0.53518268350652
H	-3.30095533283755	-2.34393274692352	0.41931932914549
H	-1.80221405710473	-2.52939116742917	1.06904456173923
O	-0.45099696226646	-0.36388464266014	1.43004386688578
H	-0.36765246115666	0.47561272554820	1.90899720558094
H	0.50384840647652	-0.59967963935778	1.10376916512029
C	3.06354488323740	0.90813718311766	-0.59939646479962
C	5.28096563671527	0.39368145349075	-1.03454052489781
H	6.21726260756025	0.73799988232655	-1.48688527483555
H	1.11281237826723	1.56204350879330	-0.34235552453441
H	2.18614796925230	2.63458138437124	-1.19679079118138
N	2.04148774323573	1.76376354139805	-0.69940560527319

N	5.30931770092500	-0.80397877554434	-0.46459184779725
N	4.25188191929799	1.24197049541654	-1.12721197054715

TS3A

C	2.62682278819565	-0.64063032746830	0.16455321732467
N	2.26688327790374	-1.47632955274806	1.18834679276828
C	1.01882997792117	-1.11240832563573	1.58690733565530
N	0.54198071442751	-0.09950256216716	0.90031030945015
C	1.53751801375128	0.21910070425038	-0.00031144094412
Pt	-1.89971638731613	0.27608945134906	0.29752484931246
Cl	-1.87852805977194	2.55025562381450	0.89999304066207
N	-1.90521798259176	-1.71315903299733	-0.19550447280050
N	-3.07761639372302	0.73449424250914	-1.26884018842239
H	2.82659560615751	-2.23002296923462	1.57792201854532
H	0.48754367388112	-1.63132476502778	2.38259607443361
H	-2.63967107874474	0.49476160368617	-2.16276082152550
H	-3.25665430613435	1.74305761727241	-1.28028169504496
H	-3.98691825612804	0.26494354466432	-1.23695258883795
H	-1.07426217898457	-1.96342308865459	-0.73969969057437
H	-2.71675122786480	-2.02302034247488	-0.73646344502035
H	-1.88291429782010	-2.28115214533448	0.65710581104169
O	-1.62513706993235	-0.47127770769096	2.61361726022783
H	-2.48392356314742	-0.44883930135061	3.06015688244413
H	-1.08453386776659	0.17350750352032	3.09429080513109
C	1.66633057850608	1.20071602688151	-1.01792159988042
C	3.75155704638987	0.30437079406163	-1.48207625194526
H	4.64232999296872	0.36175690954493	-2.11685542678540
H	-0.04363756975571	2.27576068207212	-0.62939307357715
H	0.92491027052061	2.82231158257621	-1.97423512088529
N	0.71334478855838	2.10707119006510	-1.28792848224187
N	3.75848701555698	-0.63572020008960	-0.54711232643814
N	2.79234849494288	1.19861284460632	-1.74698777207292

Products for TS3A

C	-2.53210206503456	1.70364248245318	-0.16039196509253
N	-1.46142316925071	2.43097598754175	-0.61686218043617
C	-0.37358096461235	1.63239565454824	-0.60372707021352
N	-0.67367417241641	0.42291923680904	-0.16904749336510
C	-2.02561303979514	0.43626389859905	0.12402717302247
Pt	0.60861375117382	-1.14880177065570	0.05775009060096
Cl	1.23857060598360	-0.38588713584861	2.18587938284536
N	0.11601734125332	-1.83019021901946	-1.82062045551352
N	1.88121832922724	-2.74049070688563	0.31155373493610

H	-1.47730949962780	3.40604397224931	-0.90646930010843
H	0.63307143493787	1.94915254893025	-0.87713795050481
H	1.41300398567286	-3.64741975368400	0.23387239913775
H	2.31272135060055	-2.70218738261860	1.23929558928546
H	2.64355706770490	-2.74088454858585	-0.37197179251012
H	-0.66586847257847	-1.31008899272217	-2.22794383192933
H	-0.15232656265615	-2.81815585906949	-1.82435591913877
H	0.90177616164081	-1.73435903775128	-2.47000266800837
O	2.64676614188495	1.51890406275746	-0.15311115194714
H	2.37983599540515	1.12472994953328	0.69311000303056
H	3.24808674921925	0.85793252136056	-0.52085376042744
C	-2.94308961078231	-0.51251125392661	0.64996369127592
C	-4.56514179320513	1.13383350700737	0.47710671442488
H	-5.62006505988223	1.38225261548247	0.63288642245732
H	-1.64504186078597	-2.08411378638279	0.92740599394102
H	-3.31146317877407	-2.37089237513169	1.38233884761075
N	-2.60387508234902	-1.76031265039498	0.99083479108775
N	-3.79638176137866	2.10153315621345	-0.00501467983944
N	-4.21325762157529	-0.11293012080850	0.80569938537842

Reactants for TS4A

Pt	2.83209494679977	0.27129266898850	-0.21696126496877
N	1.76273117235022	-0.60893123724747	-1.66470502223244
N	2.87087142675083	-1.46545231994226	0.88419282824480
H	1.93601670693640	-1.85197067222615	1.04297332884938
H	3.28214710434646	-1.30314860915225	1.80772772606830
H	3.42564070863991	-2.20281001974589	0.44023403478705
H	0.76327483934376	-0.41461377147615	-1.45186893693830
H	1.89547172193473	-1.62005819146328	-1.74313403639006
H	1.97131239088981	-0.19874499044506	-2.57890993529389
Cl	2.78886805723298	2.25882181271421	-1.45068376101799
O	3.90930047552732	1.19107163079777	1.36518726354371
H	3.58707652599342	2.08423379689185	1.57206717892608
H	4.85324224045860	1.30043587855065	1.16106434905291
N	-0.73671317873894	0.46414843780524	-0.60612574531376
C	-1.98240661657605	0.10357323253781	-0.14804405671012
C	-0.63272572949977	1.74078064928940	-0.32175993700565
C	-2.63159607624187	1.20121081170716	0.42133006933508
H	0.23578658054160	2.36042049634260	-0.54899187738054
H	-1.88310228179769	3.19376342067300	0.60463218202752
N	-1.74325080099855	2.23607525819427	0.29540136100505
C	-2.69503137676345	-1.12214441764136	-0.14033800914618
C	-4.42089993339392	0.00413770248224	0.90980856874591
H	-5.42626680369584	-0.07668543443858	1.33714802942158

H	-2.73573312090783	-3.10169609897230	-0.62433257821641
H	-1.26339359122113	-2.26500705684510	-1.06224122424505
N	-2.18600795484635	-2.25195197173214	-0.64817076431589
N	-3.92153049736160	-1.12895880004463	0.40080631546259
N	-3.85515693570280	1.20222779439792	0.96367391370509

TS4A

Pt	1.95768272539408	-0.15446576813304	-0.21375221789489
N	1.40813267389084	-0.80286942186163	-2.03637186012636
N	2.82109615018061	-1.96173818573289	0.22783466463900
H	2.17517582917724	-2.75554677326243	0.21265622875907
H	3.23506831669901	-1.93439167462226	1.16389127526283
H	3.58114777235468	-2.18483733109859	-0.42066030482190
H	0.42349839445848	-1.08695665123272	-2.05193771741072
H	1.95011463290636	-1.59649624382761	-2.38971406987456
H	1.51071488470356	-0.04700710598981	-2.72076198957122
Cl	2.68505491714626	2.30064888825425	-0.93597217011835
O	2.58623005799405	0.60628520506611	1.64784250256808
H	1.88407327772971	0.64235543214693	2.31885689261338
H	2.81000748403495	1.53517314062045	1.43769698722563
N	-0.23712359616672	1.04833771465639	-0.08257729897689
C	-1.47133464192259	0.48570386918040	0.16957039275422
C	-0.47325048360468	2.32899602795649	-0.24901363717829
C	-2.46073079006834	1.47182914886281	0.14409291441956
H	0.29618392260815	3.06725396324676	-0.46707263563887
H	-2.20122673520276	3.56227739532793	-0.21116554195436
N	-1.79099560483965	2.63665539293614	-0.12238041886416
C	-1.91415810026736	-0.83329269571234	0.44625408167490
C	-4.06262059231193	0.01581326601922	0.57471175403333
H	-5.11944366769896	-0.21622756014711	0.74428031738252
H	-1.44204800992666	-2.78035860314246	0.80078947866615
H	-0.07743804397303	-1.72572781715167	0.52924758415093
N	-1.07804256394318	-1.88222090879176	0.50539284293039
N	-3.22571443651888	-1.02650282831842	0.63824626966906
N	-3.77005377283322	1.28731012475084	0.34001567568151

Products for TS4A

Pt	1.77019340154770	0.09902958305173	-0.52065155028428
N	0.83352249804035	-1.18943171757246	-1.83075620290651
N	3.55092111974611	-0.86861581477092	-0.81214043219729
H	3.88446458602829	-0.80453560991730	-1.77802285078640
H	3.49959873776802	-1.86427872026992	-0.57902545419755
H	4.27777339357686	-0.46017726978012	-0.21778764161345

H	0.22550580774946	-1.84135208391225	-1.32074164158564
H	1.47940066659206	-1.75521805308255	-2.38686148403758
H	0.23880570360043	-0.68851487611965	-2.49745134263011
Cl	2.87190965380631	1.56194167621731	0.95699305173674
O	1.03115988469222	-0.54805758688496	2.63352413512121
H	0.32879743861469	0.04640320357397	2.93347529285076
H	1.72765557833480	0.06370806517481	2.34339602996939
N	-0.01338815329700	1.06211021898367	-0.23705372784120
C	-1.23930072960441	0.51247295551462	0.10070430443696
C	-0.24463880057706	2.32890604501314	-0.52355460228092
C	-2.22034553678274	1.49840644100312	-0.02602673072295
H	0.51135272580944	3.04663036506348	-0.83528815665142
H	-1.96777152321452	3.54741446075650	-0.58349685002504
N	-1.55216411133701	2.63606303382102	-0.40243306106020
C	-1.68194448920412	-0.76493452924208	0.53108253156855
C	-3.83614353740437	0.08425610005628	0.47898537951274
H	-4.89929533969054	-0.13723508356726	0.61610810087912
H	-1.27703330296470	-2.61724841189114	1.17000917561814
H	0.06313145437519	-1.54250231081177	1.19135080425898
N	-0.83461407875767	-1.79732347106237	0.76377851070400
N	-2.99848084406625	-0.94126615909893	0.67355098576328
N	-3.52907120338151	1.33335054975401	0.15833242640066

Reactants for TS5A

C	1.96047078567258	-0.18759071459906	0.35140762825260
N	1.06475977281590	-0.37851535361414	1.38031107262611
C	1.33687354231994	-1.57344691488335	1.85385907253948
Pt	-2.24534364230393	0.37874022044173	-0.05920408702376
N	-3.33671656412990	-0.82809659449444	1.12737965070042
N	-3.27210891730907	-0.17910842377268	-1.70641151531614
H	-3.37539344761850	-1.19481453888479	-1.79042530481380
H	-2.79134378857918	0.14040079915100	-2.55324706996519
H	-4.21607796668981	0.21892464889283	-1.73218024151586
H	-3.18572672008341	-1.82139891792739	0.92514715340981
H	-4.34357233171169	-0.65657462139939	1.04529353270935
H	-3.08579559913657	-0.68131081877896	2.11027805187267
H	0.81366250542622	-2.05854899283963	2.67646914104277
H	2.73983898598928	-3.09846007620541	1.39592757712748
O	-1.18759141315225	0.89903971439445	1.66046332616425
H	-1.08482408433170	1.85215726272608	1.80803853473843
H	-0.25196790735489	0.47016070972474	1.64078898966465
O	-1.13566692494163	1.62561741228250	-1.32684912945455
H	-1.50474189087118	2.52281931034121	-1.37542890554756
H	-0.16693347923116	1.74019333875643	-1.08743491650617

N	2.36127867308895	-2.17503361980794	1.19821464154134
C	2.78106239206603	-1.31391283619158	0.21943002904670
C	2.20641707761788	0.86190599496002	-0.55287858041953
C	3.85844998279482	-0.43605416080685	-1.49942585429090
H	4.62184108624934	-0.50768492340809	-2.27968332446789
H	1.22459857340222	2.36555652213772	0.39076016120584
H	1.86770790324231	2.76192239499543	-1.11627036662323
N	3.74087356017929	-1.47739311162443	-0.68851908259116
N	3.15087967784395	0.70513108953552	-1.47195189262449
N	1.45510015873616	2.02534520089847	-0.54380829148166

TS5A

C	1.64785772103876	-0.17915294503612	0.02669752984951
N	0.55500859866420	-0.22341424287376	0.86950478517857
C	0.70015151799645	-1.34897217700243	1.53747493933900
Pt	-1.76652389261439	0.24228182874696	-0.03262195425183
N	-2.51265894100705	-1.32160958279343	1.00052549786763
N	-3.08997992509200	-0.03729580868478	-1.51794327059256
H	-3.01192162064981	-0.96501395362954	-1.94480787968896
H	-2.92231254488355	0.65270850681714	-2.25724919317992
H	-4.05992857929128	0.07950616349864	-1.20898623614990
H	-2.03055599828259	-2.20437671850627	0.80446819333560
H	-3.50633175036924	-1.48885738266430	0.81615987122326
H	-2.43271401447105	-1.13670122529022	2.00660615268020
H	0.02080675138540	-1.71846282517947	2.30528947291614
H	2.09441201272497	-2.94609480776380	1.55229886317367
O	-1.40537631877684	1.10234714776316	2.28117745065009
H	-1.84423921265684	1.93318495935407	2.51377306066681
H	-0.45609859122614	1.28359007878811	2.34295092854951
O	-1.12738996806654	1.88286121365150	-1.15755293651536
H	-1.41514546380495	2.73017403187706	-0.77934864072433
H	-0.12940979343817	1.94937592727091	-1.27070559446712
N	1.80110456755208	-2.04923881485808	1.17123119278083
C	2.42516425558899	-1.33404873079667	0.18410646382813
C	2.15284725148916	0.77323155546432	-0.87713077595237
C	3.83292457156150	-0.71262169557905	-1.40266471237603
H	4.69987111153168	-0.91580086693087	-2.03816914075903
H	1.40315640722133	2.48457230158315	-0.13037206096011
H	2.08442513526379	2.62351567339637	-1.65538765061436
N	3.51827454145116	-1.64000129553105	-0.51017027001459
N	3.22047263109224	0.46593336013978	-1.60078438379875
N	1.55410954006873	2.01838032476866	-1.02836970199372

Products for TS5A

C	1.63234679750711	-0.20729193509603	-0.26268981663501
N	0.43291834896320	-0.21610674248928	0.42755582803859
C	0.72151321837536	-0.57114421539962	1.66841525855874
Pt	-1.38605196009306	0.40337878514070	-0.27223223982983
N	-2.22693543319276	-1.31189745300416	0.35654789129155
N	-3.18140180844497	1.08032730457182	-0.99206759966481
H	-3.58472774698954	0.46186664334085	-1.70234815701795
H	-3.07255802690006	2.00004171595113	-1.42954282002454
H	-3.88627813059785	1.18386952353013	-0.25616000115867
H	-1.64397297967877	-2.12273391123120	0.12842891447943
H	-3.14880304312395	-1.49120925296656	-0.04996483465882
H	-2.33267140209140	-1.28088567714967	1.38719921481723
H	-0.01922419206375	-0.67182383898568	2.46577595159907
H	2.49701045155167	-1.07128321706187	2.68942525482010
O	-2.00088500907598	-0.85795832253595	3.15186656464220
H	-2.04129861603800	-1.53728967913733	3.83971373633571
H	-2.40267377787087	-0.07657229476766	3.55722275121918
O	-0.54058113060895	2.20924472544446	-0.97844366618975
H	-0.13265800685092	2.77200672727831	-0.29918096198636
H	0.15154757087407	2.02831913919529	-1.65208059705371
N	2.04049109747927	-0.79400325625707	1.82247203100075
C	2.65640524817883	-0.56114921723513	0.61828893967194
C	2.01884172755192	0.09415617879127	-1.58719802726113
C	4.20213990940963	-0.29197324472115	-0.93448225695788
H	5.25055382341805	-0.30610519369103	-1.24684318765097
H	1.52832833190961	0.57369493621512	-3.47626690636136
H	0.22986413624131	-0.00958657352336	-2.55337854466810
N	3.95219667587856	-0.62399356881642	0.32405131473728
N	3.31603450605129	0.05611866128401	-1.87834330006770
N	1.12288942023093	0.48425325332606	-2.54702073402520

Reactants for TS6A

N	-1.73640408369256	-2.75974600010723	-1.35789119032287
C	-0.76618791583962	-2.24903430286015	-0.57335120803416
N	-1.16312446298345	-1.14242558936675	0.02884255696246
C	-2.46299123873797	-0.91576437361302	-0.39224961736602
C	-2.83536232130245	-1.94434167088139	-1.25925959326575
Pt	-0.01740260284719	-0.00564428711957	1.29019587176217
Cl	-0.05343703954777	1.77581519423804	-0.23145170340999
N	1.15302457292774	1.16313987980677	2.51809554363461
N	-0.05277798246832	-1.51763617828005	2.69486911497173
N	2.41689043531315	-2.64644738790431	0.00078095887684
C	2.83059892283343	-2.69953195253517	1.24196346241178
N	3.34662870787227	-1.51315882408417	1.68700611851857

C	3.26049818619949	-0.62762325019998	0.64805120974690
C	2.68302623330891	-1.35458860756029	-0.39407989374845
H	3.73413877913492	-1.33065860459673	2.60867570676382
H	2.79109815253989	-3.57752216216851	1.88581151698479
H	0.82460490652953	-1.62163035180658	3.21265490087841
H	-0.25730680351847	-2.43459259582964	2.28709408677599
H	-0.78333544107270	-1.34328722224757	3.39123637771840
H	2.08533610429074	1.29259039865844	2.10275179357580
H	1.29343024278369	0.80070485720912	3.46441017576839
H	0.74633163060752	2.09680249307241	2.61895308412514
H	0.22002075295827	-2.70414966368155	-0.45849447486259
H	-1.66376566857267	-3.60913107348986	-1.91328517758572
C	3.36747584490918	1.21322619460213	-0.57325077967892
H	3.63154591835086	2.27086274577271	-0.67784314901391
C	2.45424360948544	-0.64341844827415	-1.59857196464082
N	3.60615097201801	0.66556242861940	0.61455065210115
N	2.84308253211285	0.63758585691166	-1.65785936126272
N	1.85140584317469	-1.20103563347262	-2.65856763675162
H	1.76011009083094	-0.66542087841660	-3.51347592997239
H	1.65472468178403	-2.19339501435729	-2.67572175148732
C	-3.44927066851479	0.07392146343731	-0.12979239068425
C	-4.87550527813489	-1.14284703583820	-1.49442546911857
H	-5.88287356521047	-1.21297855401952	-1.91738345801580
N	-3.24448300242851	1.13347448674238	0.66722102630334
N	-4.02527120972837	-2.10026792372513	-1.83976508045939
N	-4.65211101121549	-0.09459278504257	-0.69628994106745
H	-2.29463065998820	1.44401770990059	0.84952205338581
H	-3.98327616416168	1.82691666250793	0.71279355948271

TS6A

N	-0.85156815225998	-2.12279365360727	-1.48984507010482
C	-0.24907070908467	-1.65336947511331	-0.38012836586056
N	-0.91788493090129	-0.64398271556329	0.14470503925008
C	-2.01869136415506	-0.44044727387841	-0.66952257857327
C	-1.98876931596384	-1.38407252237946	-1.69741502039043
Pt	-0.32973240691523	0.38003644560476	1.80980488759897
Cl	-0.32444969687726	2.83110798186714	0.64098403916462
N	0.27361251820632	1.42684714335347	3.46925685269744
N	-1.51512141059634	-0.82969313509918	2.95998708817891
N	1.95847406210613	0.41341377472578	0.75294659166397
C	2.97024206274383	-0.01829541055134	1.46826474197264
N	4.01621166056466	-0.43280386568555	0.70524150279857
C	3.65203859641143	-0.27067694474730	-0.60437953972688
C	2.36306704711592	0.27189326946282	-0.56123348716203

H	4.89840679092290	-0.80689973901001	1.04389481734284
H	3.00081641845183	-0.06032441097750	2.55617267645104
H	-1.67247389041859	-1.73977061105512	2.51872840031931
H	-2.43681579624513	-0.40977325613555	3.10878694271892
H	-1.11986057612366	-1.01824994138092	3.88505579958990
H	1.08294633655038	1.01741080528677	3.94364584919904
H	-0.45753725379266	1.53002093707044	4.17761116697454
H	0.53213880454114	2.37347439275891	3.17468842118702
H	0.67333289173885	-2.06785746417653	0.02331512407172
H	-0.52595223687704	-2.90203390421587	-2.05764501504394
C	3.67838393580220	-0.30692162731933	-2.80987654694811
H	4.18332711795479	-0.54108432604152	-3.75323628330326
C	1.76129267642138	0.54228350661942	-1.82158830928888
N	4.35369633741037	-0.57362552043992	-1.70061808307485
N	2.45619120574445	0.22164184345076	-2.92461380142396
N	0.53690030020790	1.07519926034862	-1.95174871388488
H	0.24019709809301	1.32989426859114	-2.88736306608126
H	0.12034635600622	1.56776003357000	-1.15747619084696
C	-3.10924528549626	0.47018115741715	-0.68031658930168
C	-3.89935751614142	-0.67395790516442	-2.53634945149103
H	-4.70248644447242	-0.75517140841776	-3.27600612282610
N	-3.25750135669817	1.46199484452586	0.21686452116881
N	-2.90039899075094	-1.53760816964256	-2.65729381317164
N	-4.04224157255624	0.29381312896770	-1.62579799011232
H	-2.41044844130457	1.87892377180292	0.61270045201278
H	-4.02201486936299	2.10351671517848	0.02979912425575

Products for TS6A

N	-3.74517355045721	0.84851969968210	-1.46849000816969
C	-2.44574623383276	0.75980133051822	-1.80889398447882
N	-1.77022184363626	0.01236176320074	-0.95600876146021
C	-2.68328104936977	-0.41461080706641	-0.00273761708853
C	-3.93541159099194	0.11476450670648	-0.32540333139163
Pt	0.24170566421457	-0.34768653283788	-1.11638393619844
Cl	1.59636739110650	-2.60374107195403	1.21187418070257
N	2.25377670437086	-0.71772299418934	-1.28163732782751
N	-0.10051983477145	-2.33630879857967	-1.52508047398217
N	0.62173785158695	1.61717398798773	-0.71430399987156
C	0.62401087192433	2.58647203846690	-1.61074643197787
N	0.79948233145896	3.79087303584727	-1.03391927947748
C	0.91334998345927	3.60068269143534	0.32024897651053
C	0.81420483632407	2.22212058851194	0.51649553096663
H	0.83471337929172	4.68351503560183	-1.52162695606031
H	0.49492645567649	2.44868881395776	-2.68270764327030

H	0.39813334134550	-2.88718035899869	-0.80883441870639
H	0.25768540051743	-2.61277209529012	-2.44337398143122
H	-1.08585492663135	-2.61158630886306	-1.50073140523586
H	2.83163750099243	0.12172259269262	-1.19446043141530
H	2.50943148697638	-1.16608330439063	-2.16551977408089
H	2.50040505679207	-1.36251466911984	-0.51346134534215
H	-2.02287957323664	1.24621787300754	-2.68594965601488
H	-4.45907532569456	1.36801300467867	-1.97448295667724
C	1.12255704694509	3.99285925771206	2.47768645234239
H	1.23224901617527	4.69319966093116	3.31217630285775
C	0.93912017236903	1.76235977176271	1.85732239889118
N	1.07066820474975	4.52802282648228	1.26465779833161
N	1.06910569930733	2.70094903005102	2.80896260627308
N	0.92410483110499	0.47607425237490	2.22275480639862
H	1.07909015062731	0.27860891224841	3.20576506075560
H	1.06335056330483	-0.30257178789186	1.57636979522345
C	-2.62899661520229	-1.23849950053815	1.15891737057001
C	-4.90354863539507	-0.83088287580170	1.41136313301161
H	-5.79304487642593	-1.02585957933157	2.01941594799524
N	-1.53717962244631	-1.85404652778993	1.61607631471783
N	-5.07423357324512	-0.06183870067105	0.34417563602213
N	-3.77793837302853	-1.40670606795634	1.83572457337010
H	-0.61078312331896	-1.83520545362765	1.18938369823115
H	-1.64307819293688	-2.42158023895982	2.45003113698697

Reactants for TS7A

N	-3.62318359583671	-2.67763826097638	0.28994591953601
C	-2.51274725646409	-2.21251449792555	0.89153307452384
N	-2.19263423208188	-1.00877812309669	0.44897879427943
C	-3.15106437924090	-0.67302943989518	-0.49328869929800
C	-4.05737808197011	-1.72650895190470	-0.60074188266521
Pt	-0.55088288233080	0.06883760639301	0.99985453983826
O	0.41004979635675	-0.46539381736079	-0.77147014865464
N	1.12513160054597	1.14342828162263	1.53066179917947
N	-1.45927719741359	0.61963484152498	2.72099139918325
N	2.69254231620781	-1.50475985353483	-0.29532557556978
C	3.20120787956900	-2.62397883665890	0.16683827683834
N	4.54603668077079	-2.55014445971340	0.32485932427263
C	4.93881124868630	-1.29244363319415	-0.05689191419984
C	3.76273788033723	-0.65064474819846	-0.44558441733900
H	5.14848010906089	-3.29382027423928	0.66929400748457
H	2.63372622860695	-3.52097534043050	0.40836289641581
H	-0.91417585630962	0.35052922370045	3.54608144259140
H	-2.38455335742083	0.19441870338384	2.83030757308810

H	-1.59741191672456	1.63376015925102	2.77443408485923
H	1.79279603593208	0.55989964008917	2.04502735605983
H	0.92516090151041	1.95231936481146	2.12604233105519
H	1.62670911924301	1.50558557695156	0.70963021165065
H	-0.13667001575208	-0.94663691585113	-1.41240446824156
H	1.28107570674021	-1.02196342322451	-0.59854616823502
H	-1.95966177692671	-2.76790861379786	1.64670655017526
H	-4.06052189050235	-3.57929756055636	0.46952388512644
C	6.15509984563056	0.49960963514209	-0.48160160680661
H	7.12431864542579	1.00759275381575	-0.51241312730397
C	3.88550132760510	0.69098312134298	-0.87888727919453
N	6.16157480409288	-0.75772370038115	-0.06031402216064
N	5.10622576718101	1.23321690351550	-0.87961731297504
N	2.81523425833969	1.43572906520012	-1.23824909117542
H	1.94791991791943	0.96881070290947	-1.49267527490418
H	2.99606681082438	2.34104045177689	-1.65997602450222
C	-3.38525370279701	0.45562585173972	-1.32470081069516
C	-5.23524488974140	-0.67763294598741	-2.14004314946395
H	-6.08475037060726	-0.64384722176204	-2.82981424016372
N	-5.11689664098568	-1.77751392002950	-1.40785666473010
N	-4.44716988122895	0.40145349805006	-2.14019519733007
N	-2.60692290161835	1.54269320603016	-1.33787519912131
H	-1.83573578400759	1.65386638710926	-0.68989808406045
H	-2.86240027062577	2.31820956435867	-1.93845310736729

TS7A

N	-3.57156831543182	0.40262667469838	1.50298962662567
C	-2.68201302344703	-0.59955270499259	1.37874673594858
N	-1.82205008552090	-0.36542068816248	0.40294044884909
C	-2.17444456292364	0.85947674914580	-0.14122169033314
C	-3.28200812298864	1.34916573725821	0.55135993858203
Pt	-0.22993617928758	-1.57348623167614	-0.01562920728642
O	0.56033388932647	-1.70607437373610	2.24387113179306
N	1.36999137126005	-2.83518868129721	-0.28894839001603
N	-1.24463982199367	-2.41849138297790	-1.54875972654834
N	1.13422152457275	0.51962485398523	0.54742476183275
C	0.78507161553978	1.39055151490703	1.46731938680760
N	1.69491741032728	2.38777678977596	1.61885251767388
C	2.71294286415547	2.14619200784373	0.73601912603082
C	2.33748228567643	0.98070499196106	0.05789570400394
H	1.63893891022621	3.15687078712011	2.28184486265856
H	-0.12146275225254	1.34170361300071	2.07191570933679
H	-0.77991454429387	-2.25648442014848	-2.44711669771862
H	-2.19189776835150	-2.03842365973006	-1.63268846870995

H	-1.34220889547815	-3.43148144199284	-1.43551826331001
H	2.21871310509626	-2.35031004543495	-0.60344812387304
H	1.21613833592075	-3.61077752802835	-0.93915136488289
H	1.58955909678572	-3.25000165397791	0.62222539028382
H	-0.09107913273984	-1.92771611144265	2.92590594648628
H	1.08060900274336	-0.97523759093907	2.60746696109636
H	-2.68181390979493	-1.48613149185724	2.01078030340462
H	-4.32727297014123	0.44237293502280	2.18386841122848
C	4.61355525778260	2.29696586266701	-0.37688836737129
H	5.57063315536679	2.79359595589587	-0.56564480514501
C	3.22010603050286	0.54560963805093	-0.95909309527813
N	3.83915174423743	2.83778418828734	0.55282068819523
N	4.36107370732726	1.21675394221365	-1.12755727421779
N	2.97108901576198	-0.53476735789883	-1.74559607605884
H	2.00199557269009	-0.75697732719710	-1.95147771848793
H	3.60695762064423	-0.65745678845203	-2.52842356602461
C	-1.68760644808478	1.66835456358293	-1.20486468429776
C	-3.37708725205657	3.16916448798510	-0.68705484978352
H	-3.84443518096002	4.12662045069392	-0.93894970093913
N	-3.91909918089913	2.49664040913462	0.31956315825952
N	-2.32836551612720	2.82281184580939	-1.43766967110107
N	-0.65193621625890	1.33263748968446	-1.97842559738387
H	-0.11278178888052	0.49637081951710	-1.79644105627191
H	-0.35464984803136	1.96945317170059	-2.70857241405777

Products for TS7A

N	4.01644079623484	-0.20928949287437	1.07199751341775
C	3.15298309024512	0.78537417033295	0.79802822492346
N	2.15506802345959	0.36997950388408	0.03656329597706
C	2.38495973673413	-0.97648369536311	-0.19738851382460
C	3.55543585448138	-1.35052336637882	0.46492117148303
Pt	0.50907068720178	1.54536327465722	-0.33813228873989
O	-3.09387722617413	1.92980877703858	1.37638510944931
N	-1.10735056855445	2.79992455426148	-0.54377426234032
N	1.65882600560907	2.80646421142037	-1.48896365123203
N	-0.64320955324756	0.29637285244897	0.78645662370190
C	-0.50946521749975	0.12185776820836	2.08718462697558
N	-1.44924329281457	-0.70939951776201	2.57304374611208
C	-2.25465614610401	-1.09326919312720	1.53117753163203
C	-1.73828671223221	-0.45770848585337	0.40150535816993
H	-1.54545606330207	-0.99336322682198	3.54546291341742
H	0.26254071107658	0.58142603686725	2.70196654398078
H	1.15909000754388	3.14242273038938	-2.31730151839528
H	2.51254592572941	2.35526275968781	-1.82892741199443

H	1.95363923736320	3.63942239923201	-0.96989693531486
H	-1.45785744975921	2.90068474631050	-1.50078337141435
H	-0.85101610656810	3.74443570905910	-0.24076942663341
H	-1.89950783345873	2.50713550243098	0.05833670567727
H	-3.43984449524715	2.64790514615943	1.92458054205273
H	-3.88726586574470	1.51774029678123	1.00595096378816
H	3.27074525573783	1.79967206054213	1.17626286656478
H	4.85512204843857	-0.12542896855231	1.64280211223708
C	-3.87169134513595	-2.04739886472349	0.37702400632725
H	-4.75007005039667	-2.69879959585552	0.32781121917728
C	-2.43477027784687	-0.67459154252444	-0.81640176601671
N	-3.31458196138692	-1.89874139807747	1.57117522745091
N	-3.49561531549689	-1.49115429656825	-0.77829202267463
N	-2.09458904352929	-0.12657644534442	-1.99438761076660
H	-1.38289675702954	0.58765008260338	-2.07543185286904
H	-2.69070108437332	-0.30155022631477	-2.79558183952301
C	1.73468271561317	-1.99562696454071	-0.94362054180829
C	3.37396248485184	-3.44827449839432	-0.18779711776203
H	3.73865981708738	-4.48028200961732	-0.19218122797938
N	4.09198557371764	-2.57011014920577	0.49891463172684
N	2.25842578900528	-3.22602885408195	-0.88868982882267
N	0.63221752717516	-1.79103727255794	-1.67839131104634
H	0.34661810137570	-0.85482038240952	-1.93470619192533
H	0.28893297722063	-2.55844513536615	-2.24612824315944

Reactants for TS3G

C	-2.63305438413691	-1.08503894669290	-0.45935999609466
C	-2.50171950718302	0.32086553169263	-0.21188973387862
C	-3.57371245508914	1.00185693786821	0.35970328451472
N	-4.77390212717113	0.51188649378737	0.71368920368120
C	-4.90387996450110	-0.77732063910711	0.47693233831830
N	-3.89759519753936	-1.53877994158069	-0.07201020115133
N	-1.45246111431049	1.18785243875398	-0.42020051815097
C	-1.86782379023401	2.35234829923194	0.01064695032870
N	-3.14234064035274	2.29115700924073	0.48956630542707
Pt	1.66830228137602	-0.76950968067067	-0.05170630993813
O	0.91351655507859	0.66271976593773	-1.36794479919123
N	-6.05155070903168	-1.40440663853327	0.76452904119894
O	-1.81583942878026	-1.86512577659383	-0.92986832582455
Cl	2.36994309898365	0.91418105171332	1.41454955950827
N	1.01859762857953	-2.18871633289473	-1.38684176368971
N	2.39258451629924	-2.16784930691590	1.21907607164009
H	-3.67654310375834	3.06492854352571	0.87643228428895
H	-1.28942402485766	3.27422159094616	0.00398702893758

H	1.66549240813788	-2.80381516927747	1.55910883131980
H	2.80563042303564	-1.71864418129205	2.04158646698472
H	3.12175663930599	-2.74875349120943	0.79556540779910
H	-0.01166900455162	-2.17774007231074	-1.38792955096982
H	1.31958059595925	-3.14485134091276	-1.18570115217971
H	1.33410516047448	-1.97371659778771	-2.33594665719708
H	1.41514427902162	1.48769132043794	-1.27091978370975
H	-6.16931592730527	-2.40164359793017	0.63991225261945
H	-6.79295909460219	-0.87164454082861	1.20208300543374
H	-4.06248389494594	-2.53264149163941	-0.22488032198703
H	-0.04372921790100	0.88786876304174	-1.04928891803802

TS3G

C	-1.43241713405704	-0.58797783272827	0.74610296373337
C	-0.95948111599742	0.52857697551683	-0.00114470480058
C	-1.88617016908615	1.33395402293595	-0.66109261388669
N	-3.21804240182577	1.16957455737748	-0.72046223969160
C	-3.65218989018620	0.11932734211482	-0.05358272317444
N	-2.81251138095273	-0.71873288447577	0.65089808330288
N	0.31302726974178	1.01534081638073	-0.17738231120109
C	0.17104480136562	2.08387419859661	-0.91606820439951
N	-1.14011684055797	2.32095597558964	-1.23465831601045
Pt	2.62291063277259	-0.00482672341551	0.12170187404275
O	1.44140357529159	0.12496597053298	2.24057161506869
N	-4.95876375152020	-0.17105293107285	-0.03099772928794
O	-0.76584624442012	-1.37415427739138	1.42851357853431
Cl	3.44449012061826	2.19169762102813	0.20074277538032
N	1.87736873380698	-1.92000482999504	-0.00212317024557
N	4.22852161584762	-0.51436930921813	-0.98802716848416
H	-1.49243443243980	3.09202780696837	-1.79555631468325
H	0.98452270368344	2.73016850194898	-1.24204758405861
H	4.86277596390489	-1.14971610601982	-0.49679756841445
H	4.76316241402873	0.32865396663889	-1.21719127834945
H	3.97592667727675	-0.96215233340022	-1.87289029192251
H	0.97585753564166	-1.98580750731435	0.48990140634168
H	2.50058032446795	-2.61927575184706	0.40972011778430
H	1.70964004519615	-2.20775442575769	-0.97005708651277
H	1.94989937060446	-0.35209819518062	2.91134269541630
H	-5.32650981902967	-0.98911634494712	0.43762112707166
H	-5.59063365487460	0.42464970054843	-0.55159582759178
H	-3.21397009481655	-1.49850051068557	1.17078444750826
H	0.62795514551576	-0.40822749272842	2.12377444853035

Products for TS3G

C	1.15805220005990	0.65681537194305	0.76780831178093
C	0.02669212541201	0.82632019666179	1.63367263419705
C	0.10111906620606	1.76617555744318	2.65485143733065
N	1.13751952740615	2.55006114152856	2.98245630138391
C	2.18906417631685	2.38750925847579	2.20371064586349
N	2.20239952614699	1.49959372441875	1.15288400205186
N	-1.23879842310039	0.27413771526576	1.62484743648290
C	-1.90133520836142	0.84673566411634	2.60611156084408
N	-1.12439994964921	1.74188539511548	3.25965216530631
Pt	-2.04037734955036	-1.01937654751825	0.26625137610220
O	-1.89297946026507	3.03259051028052	0.44824003338485
N	3.30060606176011	3.09896211624842	2.41620644947860
O	1.28590107170663	-0.09446378768204	-0.19040925694159
Cl	-3.85121725018891	0.45256950131300	-0.07094929114443
N	-0.45231970739423	-2.28927324745991	0.55933138720731
N	-2.87294848910755	-2.30862138192006	-1.10350225332302
H	-1.41168821173159	2.30753880679079	4.05508625728744
H	-2.93838955420147	0.64580962442413	2.86462097763543
H	-2.27081788934705	-2.45876773017035	-1.91793521866885
H	-3.76033603100177	-1.93842089812345	-1.45471811197790
H	-3.07164389285740	-3.23237685129002	-0.70880665980304
H	0.39911869237275	-1.81678605616970	0.22684679560921
H	-0.52720405172767	-3.18216408749357	0.06617135674810
H	-0.31613948713664	-2.51183685391312	1.54908808996002
H	-2.18510612259767	3.38250231509666	1.29903020232109
H	4.12990347034969	2.99512920968314	1.84555931006439
H	3.31065919725483	3.75874730609366	3.18425659601924
H	3.04133290036054	1.42811403466241	0.57809534538245
H	-2.54248238713402	2.33503639217900	0.26201133941723

Reactants for TS4G

Pt	2.45584194559777	-0.76610436767002	-0.03855069384169
N	2.60691046110890	-2.16510551550375	-1.49077267291965
N	1.71923196606886	-2.12191370563153	1.31701468455688
H	1.39462312414388	-2.99961498629268	0.90453505078294
H	0.91235116797941	-1.69332946226113	1.79680951615710
H	2.41473319485158	-2.36640895148843	2.02620667558232
H	1.71174174942228	-2.61455798527270	-1.70392257615592
H	3.26857309717970	-2.91080203574637	-1.25680937555145
H	2.93716063209529	-1.73034391938250	-2.35764099816855
Cl	3.26083201118523	0.83472869606051	-1.53505479011802
O	2.29220298495512	0.66986797744379	1.46643300379458
H	1.37778519225689	1.13542448431426	1.34873223782538
H	2.96165077597485	1.36310286824895	1.35639529949821

N	-0.04221297906317	1.72331230772938	1.04175771920212
C	-1.23595753969222	1.10791168263487	1.34071547323962
C	-0.36359206798682	2.85452622879318	0.46621670627521
C	-2.29906162535432	1.90691838146091	0.92968570929281
H	0.33681093280459	3.59908295482015	0.09320513621234
H	-2.20277984270527	3.80748224252574	-0.02545656588442
N	-1.71526273651119	3.01108345481535	0.37683620198095
C	-3.88007136468765	0.50282830166396	1.62238269216002
N	-2.89726095083517	-0.35618488180908	2.06007018087639
N	-3.61433223056908	1.65674576332628	1.04472260474977
C	-1.51805933639741	-0.15125675742375	1.96361045351280
O	-0.73338080898359	-0.99291137941891	2.38319558053860
H	-3.17089025705582	-1.23687015838859	2.49378165270417
N	-5.14889453652006	0.11512705239215	1.80238951634115
H	-5.88908752100729	0.73805892687755	1.50431359908886
H	-5.38841543825526	-0.75117721681758	2.26697797826740

TS4G

Pt	2.31857107620769	-0.57226406908416	-0.27311594098623
N	2.39989164001181	-1.01545287065377	-2.23822470634663
N	2.82047890752344	-2.47817546771511	0.24973622884871
H	2.12577100453561	-3.16681583766080	-0.05128613752317
H	2.89203848527252	-2.55377064004513	1.26850285854147
H	3.72269845328751	-2.77230671451676	-0.13323099904114
H	1.47114956265272	-1.17283895364600	-2.64002383153597
H	2.97081742344647	-1.83054636262263	-2.47665603172611
H	2.81037316767635	-0.19816041483560	-2.70534741887799
Cl	3.37532253476522	1.75426536285766	-1.21255978305165
O	2.18923730627159	-0.16791223713351	1.77671401260814
H	1.20714042493330	-0.26508842061099	2.01166081694189
H	2.45363904131989	0.73342789043808	2.01640531866986
N	0.37459214051003	0.91758785312863	-0.48082011473871
C	-0.81684108158883	0.69250023990061	0.16850750563204
C	0.07122015108750	1.65465437758027	-1.51665596616601
C	-1.86338028068941	1.31263661296512	-0.51371800808868
H	0.79497253849294	2.02926688038858	-2.23845859740608
H	-1.73846394842292	2.47368574670456	-2.29306153185749
N	-1.26962998769626	1.91825518920164	-1.58219460884906
C	-3.43852376603688	0.65661890335713	0.91638009928360
N	-2.46536002307025	0.03912985818916	1.67394485659767
N	-3.16556897138948	1.32002472930209	-0.18874591398873
C	-1.10722681416901	0.02074857455357	1.39060915464976
O	-0.32053110227157	-0.52680568588407	2.17406396948975
H	-2.73735217667240	-0.43206476712765	2.53618170369541

N	-4.69930127843080	0.56176056789054	1.35325286104855
H	-5.42927564500592	1.00815766663758	0.81197689673379
H	-4.94645878255088	0.05948198844097	2.19616330744299

Products for TS4G

Pt	0.69839075334776	-0.90181181957841	-0.47913100335236
N	0.10991551335344	-2.69520958442320	-1.21063428101078
N	2.48622288734270	-1.69290175398566	0.15783174568860
H	2.49403568530810	-2.71178013656549	0.24806496901857
H	2.69492219323329	-1.29527511058498	1.09136661683818
H	3.25965253869565	-1.44644200629328	-0.46579618476300
H	-0.65860344762167	-2.61148981067630	-1.88223648248609
H	-0.22578654981101	-3.30655166421274	-0.45975591456709
H	0.86300938905729	-3.19956517898457	-1.68737468324663
Cl	2.27587149386693	0.02023471985183	2.85773070561965
O	1.25658282390053	0.95691763637235	0.30828338259292
H	1.95395592718294	1.39203719796144	-0.20739889391539
H	1.64700366968110	0.78754780567779	1.23427807575568
N	-1.06007348452335	-0.05724713249541	-1.07443190228014
C	-2.11842644640508	0.23759846467243	-0.24132834671730
C	-1.39711689103697	0.36993059497888	-2.27359656878998
C	-3.11748473816127	0.85822126719919	-0.97824796257220
H	-0.78053787644444	0.30406552270112	-3.16735596871451
H	-3.11293489303724	1.32741068514525	-3.05853314265840
N	-2.63229022198390	0.92639716420412	-2.25635726672677
C	-4.50159453796720	1.10506989669505	0.74579139107142
N	-3.56865598616881	0.50249781382852	1.55842815341220
N	-4.30623476563994	1.30245572874531	-0.54389035901589
C	-2.31192992855188	0.02044762655291	1.16629354119729
O	-1.55230425242737	-0.49075882101656	1.96865470316368
H	-3.78389570599565	0.38249376416532	2.54732350597237
N	-5.64805197993527	1.49108823888345	1.31577057348623
H	-6.33499507953139	1.96281122950138	0.74031125319443
H	-5.81877351972726	1.39416261168025	2.30858183380530

Reactants for TS5G

C	-2.59226593719291	-0.36565900166519	-0.67660281029213
N	-1.73818459994141	-1.18462606499159	-1.38768664214103
C	-2.47387633526965	-2.19966150600293	-1.76318222887533
Pt	1.87271914415545	-0.51450414372492	0.11475419564365
N	0.39679687610431	-1.48279028329892	1.08732040328701
N	3.26119444295371	-1.20756170119765	1.41455284368848
H	3.06114217372959	-0.94468601569003	2.38454070448209

H	4.18646129266288	-0.82751274865117	1.19114009709334
H	3.34786168693196	-2.22830889498794	1.39319643123551
H	-0.07085824257119	-0.86464948153115	1.75921040935921
H	0.71461101383859	-2.30253517133047	1.61308530090960
H	-0.32347989958177	-1.81512856280679	0.43490696116090
H	-2.13473024520954	-3.05208560895716	-2.34889304997173
H	-4.51475235100187	-2.75389445867109	-1.50164888136406
O	0.52009302977055	0.25238031269019	-1.25867590805583
H	-0.14118847228122	-0.43643001706092	-1.54925224670947
H	-0.08628944515437	0.90245056827339	-0.78969690650500
O	3.43392130428964	0.48301927668509	-0.88999488108098
H	3.59753485267406	0.13982821374845	-1.78490015675283
H	3.29190856280274	1.43909658710030	-0.99552897477230
N	-3.76587172512322	-2.08630005647719	-1.33518393844182
C	-3.86981988001536	-0.92055125302690	-0.63389610823781
C	-4.75718782395422	0.72483746225607	0.57488696401409
N	-4.96331693373612	-0.42445245912229	-0.03615402113179
N	-3.52827555513497	1.35004778697213	0.58969522149186
C	-2.37045795952381	0.88254116568556	-0.01706023660337
O	-1.32530510788755	1.53831806973158	0.05556354372486
H	-3.43261320184224	2.24229266432890	1.07407433843288
N	-5.76006156344437	1.33584265774870	1.21395231099577
H	-6.66932037060728	0.88995419999071	1.21562297047073
H	-5.64150873044035	2.21550846398331	1.70012429494551

TS5G

C	-1.16692005300637	0.18826097552639	-0.29898743859382
N	0.06238137319635	0.12918206828182	-0.91773028786534
C	-0.08947275878715	0.79191354717460	-2.03582660346883
Pt	2.33794710188965	-0.48179596121668	-0.02482233838681
N	1.79022811056382	0.21314324948917	1.78130107102228
N	4.18700183258986	0.30393311242382	0.19754330969093
H	4.18606863863760	1.32628646444205	0.14161187782505
H	4.80657530914502	-0.03571454451639	-0.54458896899747
H	4.62468624710065	0.04887419362330	1.08742840457307
H	0.83123411167115	-0.09006858429551	2.00679695086248
H	1.80561528072828	1.23653589077307	1.82572862689556
H	2.39793116961307	-0.12875623739838	2.53180702904127
H	0.67998476586210	0.95759526718412	-2.78862450970742
H	-1.70607684773775	1.82230572865665	-2.96783386624743
O	0.84618154865290	-2.37149693806387	0.20880818669350
H	1.30312705268233	-3.06199198413236	0.71004350583284
H	0.13574665203472	-2.06127166353047	0.81044426952991
O	2.97434782026862	-1.23612926886232	-1.88931546806762

H	2.75459358534573	-2.17267585106740	-2.03025337428241
H	2.64069641124136	-0.76470276883927	-2.67065050489639
N	-1.35812943787635	1.27895528222106	-2.18172933126116
C	-2.06934581017581	0.91166599956187	-1.07785975537553
C	-3.76287852005770	0.71649796065220	0.35642977238452
N	-3.35077233025787	1.19517681967965	-0.80040492043751
N	-2.94511050231526	-0.01672438891061	1.19135422429269
C	-1.61778794346267	-0.33864974648699	0.94550856608071
O	-0.96945335066763	-1.00985188342370	1.75791732459350
H	-3.32324485031252	-0.37093137036355	2.06980040440174
N	-5.01933809360512	0.92570707879495	0.76460517058336
H	-5.62804244901660	1.48060134617112	0.17550893976574
H	-5.35777406394442	0.61412620645164	1.66598973351860

Products for TS5G

C	-1.66037901067468	-0.21009346626631	-0.17419863855747
N	-0.53632907595271	-0.83852739491030	-0.68552670169162
C	-0.95598532153914	-1.96864742638712	-1.21504656890094
Pt	1.40134244275099	-0.18212952102818	-0.59840226255108
N	1.70045076237190	-0.67731251902116	-2.54943783295199
N	3.32781432880507	0.49529590940727	-0.43046929992929
H	4.02484425105244	-0.24285790450489	-0.56462978190847
H	3.47849389358183	0.87818642352876	0.50747937917590
H	3.55478403712911	1.24189436704315	-1.09381316200969
H	2.02330642517568	-1.64479140718562	-2.65339990157213
H	2.40102281910748	-0.08781609537621	-3.00874024775913
H	0.84752531961858	-0.58970002714556	-3.11001607776103
H	-0.32768801206867	-2.71144952056529	-1.70318659226494
H	-2.84857457035708	-2.88852808686872	-1.42165821777546
O	1.14688607364594	0.36556071245541	1.38547610727169
H	0.43988364087770	1.05940524142662	1.37705073962861
H	0.75451649476636	-0.41627247408939	1.88455542819695
O	0.07155382906522	-1.75075531027418	2.40306515962643
H	-0.88452891691465	-1.65836655016233	2.53081852948898
H	0.40222353000320	-2.05839083918306	3.25968411102493
N	-2.29554873624318	-2.10255947331456	-1.08554541703273
C	-2.77722689393119	-1.00018468563259	-0.43596653776264
C	-4.23860660897494	0.40105367393316	0.48680252933140
N	-4.05568055174660	-0.74774686212492	-0.13642405753269
N	-3.19487340039224	1.23934016640392	0.80750921268606
C	-1.84976197374823	1.01992643076919	0.53631687747520
O	-1.00427849697934	1.82950285807115	0.91441637200406
H	-3.39280759034745	2.10088510415178	1.31651844693747
N	-5.46538149012169	0.79145219622777	0.83583920523651

H	-6.24572470495319	0.18503328163490	0.61372389559871
H	-5.64204549300650	1.66798719898736	1.31067030427839

Reactants for TS6G

N	-3.438419000	-1.411436000	1.771376000
C	-2.262832000	-1.820931000	1.269151000
N	-1.823259000	-0.975695000	0.310203000
C	-2.746652000	-0.001779000	0.207991000
C	-3.809050000	-0.245985000	1.132446000
Pt	-0.074189000	-1.153130000	-0.781771000
Cl	0.473407000	-3.377263000	1.813882000
N	1.659365000	-1.260334000	-1.869255000
N	-1.040209000	-2.462518000	-2.060627000
N	0.865815000	0.148258000	0.489913000
C	0.972843000	0.000484000	1.795070000
N	1.639333000	1.044804000	2.344492000
C	1.990204000	1.909191000	1.342337000
C	1.501587000	1.334676000	0.172660000
H	1.850087000	1.158066000	3.334721000
H	0.623898000	-0.876250000	2.343945000
H	-0.495860000	-2.741336000	-2.883403000
H	-1.917226000	-2.064246000	-2.417688000
H	-1.292275000	-3.330025000	-1.576789000
H	1.797783000	-0.352404000	-2.333531000
H	1.684174000	-1.988055000	-2.591601000
H	2.473778000	-1.422043000	-1.265393000
H	-1.668724000	-2.689361000	1.598780000
H	-3.966066000	-1.879739000	2.510381000
C	2.873139000	3.686791000	0.333909000
N	2.665076000	3.059352000	1.475874000
N	2.444904000	3.188326000	-0.876512000
C	-4.970961000	1.582981000	0.520673000
N	-4.889688000	0.478654000	1.318668000
N	-3.997719000	1.904412000	-0.392385000
C	1.754019000	1.991695000	-1.080280000
O	1.462779000	1.623208000	-2.210773000
H	2.660575000	3.706268000	-1.728420000
N	3.529104000	4.851253000	0.316582000
H	3.844058000	5.239970000	1.197416000
H	3.689319000	5.370352000	-0.538969000
C	-2.806204000	1.190081000	-0.641717000
O	-1.966969000	1.561390000	-1.441527000
H	-4.105139000	2.763272000	-0.936872000
N	-6.038626000	2.361726000	0.671710000

H	-6.752909000	2.091602000	1.348533000
H	-6.202732000	3.169494000	0.073115000

TS6G

N	-3.44042593011623	-0.71574844130701	1.91024577820127
C	-2.33760514273660	-1.37185266462951	1.47397405118674
N	-1.85067389497174	-0.80638380782594	0.39028740940931
C	-2.67085484702937	0.27072086921340	0.10866804580615
C	-3.67656726547712	0.33541926245167	1.06857534439372
Pt	-0.15089929821672	-1.39184573414270	-0.59827506314326
Cl	0.57160875910583	-2.93069497215670	1.52700609637347
N	1.58535889380856	-1.90468908568775	-1.54998186536157
N	-1.20171626577006	-1.42102240377627	-2.36386237882134
N	1.22291435870014	0.12994287891469	0.74921550461647
C	1.17219826051680	0.29979345427364	2.04418364213440
N	1.72990899686463	1.48925361811114	2.42441041910571
C	2.16851240117036	2.11966391451088	1.29544154046758
C	1.84185504303950	1.25352777858332	0.25418676125369
H	1.81326883999690	1.83768011119276	3.37543109369710
H	0.75631916717949	-0.42075415530335	2.74612162947470
H	-1.48143239330356	-0.45686641393356	-2.58328273164689
H	-2.05136158694328	-1.98622352132239	-2.29664901140842
H	-0.67388251310357	-1.78023212892120	-3.16211560755066
H	2.02374529936971	-1.03660726694292	-1.88812873412483
H	1.48869060147570	-2.54142333091041	-2.34405947878374
H	2.22391290868591	-2.35095563499671	-0.88684619273957
H	-1.89193238678075	-2.23524792777942	1.96422286137177
H	-3.99061104877767	-0.96076587772935	2.72998249406961
C	3.10928474670516	3.65617670527432	-0.01175262844345
N	2.78769169265549	3.31166960708487	1.21745042856116
N	2.83819172041602	2.86383597498121	-1.10419993027587
C	-4.64228694550699	2.15418613335917	0.22624976268003
N	-4.66801432222198	1.23291377369083	1.16863963704682
N	-3.68339448109050	2.17927148839189	-0.75970919669016
C	2.21697759372598	1.61053123142331	-1.08177621487600
O	2.06959133745324	0.97183320302503	-2.11730725119955
H	3.12801683848507	3.18443750059878	-2.02662958638471
N	3.73535760500433	4.81729866020004	-0.24688550331123
H	3.92568047140129	5.42854828173468	0.53704760472697
H	3.97811007577992	5.12156087371205	-1.18066100479324
C	-2.63759976417530	1.26772729881553	-0.92444869966657
O	-1.85551307695592	1.38118927489593	-1.85877357781808
H	-3.72223162992614	2.91596167396779	-1.46321145404247
N	-5.57060774289257	3.11562242579333	0.19523877077230

H	-6.28198458663215	3.12454022492690	0.91577337701616
H	-5.57176048891175	3.84291714823793	-0.50874614128352

Products for TS6G

N	1.520409000	-2.523051000	0.879617000
C	0.798792000	-1.399924000	0.717233000
N	1.553354000	-0.402838000	0.198372000
C	2.790302000	-0.921810000	0.020062000
C	2.821204000	-2.275840000	0.475658000
Pt	0.875360000	1.377345000	-0.595034000
Cl	1.564812000	2.585285000	1.308350000
N	0.256839000	3.163465000	-1.405611000
N	0.227985000	0.354864000	-2.277576000
N	-2.252129000	-1.691384000	1.179918000
C	-2.752964000	-2.249681000	2.252131000
N	-4.089542000	-2.531641000	2.117534000
C	-4.469892000	-2.125364000	0.868832000
C	-3.308136000	-1.601878000	0.295965000
H	-4.687975000	-2.964038000	2.817864000
H	-2.202369000	-2.484079000	3.165285000
H	-0.704510000	-0.058931000	-2.099693000
H	0.863355000	-0.412590000	-2.534848000
H	0.141446000	0.952070000	-3.111771000
H	-0.751023000	3.182274000	-1.622182000
H	0.755794000	3.401281000	-2.273441000
H	0.433544000	3.929983000	-0.737706000
H	-0.289657000	-1.337265000	0.917482000
H	1.173465000	-3.405076000	1.264357000
C	-5.771079000	-1.734854000	-0.903673000
N	-5.700937000	-2.213687000	0.324099000
N	-4.682593000	-1.193464000	-1.554222000
C	5.015675000	-2.535187000	0.062180000
N	3.837758000	-3.112287000	0.454145000
N	5.112085000	-1.222118000	-0.306430000
C	-3.382637000	-1.070651000	-1.037296000
O	-2.498591000	-0.554432000	-1.714909000
H	-4.805140000	-0.825775000	-2.499706000
N	-6.934550000	-1.772776000	-1.578965000
H	-7.756615000	-2.098827000	-1.082180000
H	-7.056304000	-1.318260000	-2.479772000
C	4.049818000	-0.289492000	-0.388567000
O	4.220900000	0.856486000	-0.737614000
H	6.030291000	-0.853491000	-0.572964000
N	6.078582000	-3.334544000	-0.002520000

H	6.002129000	-4.274737000	0.385582000
H	7.008969000	-2.960547000	-0.190282000

Reactants for TS7G

N	-4.78293939495827	-1.31413292310416	0.23412180083401
C	-3.64817033391771	-1.78850015493841	0.79708697905887
N	-2.60314059057031	-1.06964995983582	0.44418750337550
C	-3.08212019899485	-0.08242325010930	-0.40046298716586
C	-4.46055951810403	-0.22697331968879	-0.52970288712794
Pt	-0.66665226145712	-1.36208553001092	1.05727754880475
O	-0.05102545873546	-1.09288204032167	-0.90072767076190
N	1.28865679592048	-1.62626414299811	1.62474015075808
N	-1.29973674616537	-1.62598911119339	2.97352197049773
N	2.55005739647958	-1.18701333328702	-1.32694312054885
C	3.20039636246718	-1.86585907668765	-2.23908141549954
N	4.47872844814447	-1.41741544507095	-2.39397428230221
C	4.66196858416065	-0.37754419858661	-1.52808324829720
C	3.44484816837567	-0.24736070920034	-0.86484823698964
H	5.16995097485840	-1.78789217869560	-3.04119527086726
H	2.79916762884896	-2.68957761570541	-2.82603342103243
H	-1.39275870273926	-2.61791577110355	3.21502037579047
H	-2.21162036343227	-1.19409988651584	3.14839472611335
H	-0.66095700362531	-1.21627335965496	3.66156432195981
H	1.78306150975721	-2.27348083433316	1.00312174033091
H	1.42150299826002	-1.97937416440984	2.57553623593481
H	1.76801311558498	-0.71533270319809	1.55851272341560
H	-0.31748168931514	-0.17002512866984	-1.13808357362711
H	0.95304265231395	-1.16651911460961	-1.06283805944213
H	-3.62797217188667	-2.65833620279807	1.45127987416786
H	-5.71533865870088	-1.70167558670003	0.36382899935365
C	5.65489912526469	1.29508702376058	-0.44679564214947
N	5.77693798191579	0.35173563592856	-1.35860582592840
N	4.49180230545417	1.50245452233482	0.26057522964824
C	-4.71897720869732	1.52344409492728	-1.87955566072302
N	-5.30448696733817	0.52847592349678	-1.24073600817646
N	-3.36097903612469	1.74400229163433	-1.81700733302012
C	3.31309175238949	0.76424132773151	0.13969660435615
O	2.34525877987328	1.00484444644341	0.85474797540472
H	4.46783106494316	2.24365644080613	0.95967184055264
N	6.68553362394976	2.10356217832897	-0.17210529763676
H	7.55319781066527	1.96357504170926	-0.67450057432585
H	6.63915910707429	2.81987036753567	0.54141805014553
C	-2.43767404862465	0.98122415745519	-1.11199753004837
O	-1.23902722942407	1.25211615386962	-1.16495307724944

H	-2.95920689325949	2.51920251157720	-2.34445137733580
N	-5.44423128512272	2.36098837149774	-2.62345021193542
H	-5.03202450752758	3.11932314364308	-3.15282049259261
H	-6.44247591798017	2.20202210874701	-2.68874144571851

TS7G

N	2.68413416600660	-0.61924689011086	2.35943889121796
C	1.63623489011865	0.11870347965330	1.92538195942260
N	1.64190295138206	0.22858627028014	0.61322764013026
C	2.75053710473370	-0.47234346108943	0.17149551817725
C	3.41106366889354	-1.01396710738680	1.27069810159617
Pt	0.17251800327758	1.17257147276983	-0.46904403949688
O	-1.39857255856523	1.04279207758002	1.33419217960729
N	-1.33357356973982	2.09716634278055	-1.49272751712618
N	1.57097491131716	2.13269686661682	-1.56418766848432
N	-1.10081489783523	-1.06840243279699	-0.37206059253563
C	-0.47588338115857	-2.16948481195570	-0.04134428023508
N	-1.33949440042777	-3.16326379454278	0.32375200498885
C	-2.60564457576839	-2.66432960182398	0.22547116033908
C	-2.44019671540544	-1.35273019928951	-0.21867480117059
H	-1.08706355679513	-4.10159261969867	0.62217349896256
H	0.60488121025158	-2.31074239116252	-0.03646313611878
H	2.20886316395965	2.67139634742176	-0.97182828249071
H	1.18018389417800	2.78588970717863	-2.24759125044122
H	2.12907428215017	1.43194896017281	-2.07349846658396
H	-2.23069259039585	1.62831194665132	-1.27231166035137
H	-1.21914724661969	2.06386168853570	-2.50889472168029
H	-1.42681614445734	3.08420525218428	-1.23888415082115
H	-1.53586015462740	0.19557626239773	1.78301225629460
H	-1.31091988575594	1.70352801492103	2.03690499088235
H	0.89221888615401	0.54924436550702	2.59123841494797
H	2.89149924570511	-0.84189732478985	3.33064051708065
C	-4.82691615811679	-2.57000758212534	0.32613812214047
N	-3.75110693441938	-3.30858748013784	0.50707057331417
N	-4.76880171307054	-1.26951457275218	-0.12005389031418
C	5.00155757724210	-2.01392485060766	0.07894332787978
N	4.51560376258800	-1.77076174439330	1.28142625310864
N	4.41847015724730	-1.52504671779862	-1.06749132824917
C	-3.61035412613630	-0.55336307682688	-0.43658117463174
O	-3.69570549955659	0.59799856516671	-0.84586789617169
H	-5.63801333918404	-0.75466357812770	-0.25399792407894
N	-6.04202884513497	-3.07518264709061	0.57533248102345
H	-6.10697423076042	-4.02801917033910	0.91075524868258
H	-6.88935560041729	-2.53440307903944	0.45915524645446

C	3.27281312689099	-0.73189632450697	-1.14106704393112
O	2.84884883948840	-0.35040724657251	-2.22396396066359
H	4.84519533465243	-1.74064599309304	-1.96796608113413
N	6.10112463732077	-2.75805992661541	-0.06418226483769
H	6.49482106207423	-2.97619469422549	-0.97107234102382
H	6.54095524871602	-3.13224830091841	0.76779608632109

Products for TS7G

N	3.21933350048669	-2.54444238187778	-0.79246889698094
C	2.31016597109021	-2.01315712035722	-1.64544454389439
N	1.56520141463685	-1.11325774820243	-1.03844665824894
C	2.00666288558616	-1.05810136887801	0.26841657448519
C	3.05011994535596	-1.96222617875852	0.43416628250854
Pt	-0.05666653669452	-0.12170115916656	-1.78567560212209
O	0.51894635346641	2.75609380178855	-0.45675661476048
N	-1.72215010577723	0.84970461885040	-2.51705801951042
N	1.17094507369629	1.12800322735307	-2.85634840376804
N	-1.24782219776790	-1.24398001867348	-0.55888631671584
C	-1.28772663802360	-2.55299443281659	-0.43331313157340
N	-2.05401777832991	-2.91465272090047	0.62328628644546
C	-2.52810093873684	-1.77776010093836	1.21951056726227
C	-2.02169017793742	-0.72753293473039	0.46229018767992
H	-2.23838826951888	-3.86932608467902	0.92349930815829
H	-0.77178816161087	-3.26482259991810	-1.07449222120736
H	0.84209152272329	1.37286910641837	-3.79320512920609
H	2.12616667591494	0.77774132653617	-2.96069853117678
H	1.21117938486307	1.98893513654616	-2.29162785128937
H	-2.44038267146896	0.19251921976678	-2.83276272614234
H	-1.54534681432737	1.48411883272894	-3.29930338281354
H	-2.13061029126394	1.40527761217509	-1.75545227267772
H	0.71848364475335	2.03176597090901	0.16412124877393
H	-0.44976420712630	2.71868814288571	-0.45185583186683
H	2.22243874713617	-2.30931340866294	-2.68855863930419
H	3.90608672271206	-3.25727828926213	-1.02905794120474
C	-3.58923831599762	-0.47556652839617	2.67757473099274
N	-3.30567951264002	-1.70937867357946	2.30913614524240
N	-3.13490390895786	0.62735522142484	1.98972943482277
C	3.36026227327433	-1.50430352699727	2.58941421518672
N	3.74754496211522	-2.21792325084114	1.54891174633118
N	2.34125578786964	-0.58210612669218	2.51928352134598
C	-2.35109598271137	0.61899614447109	0.83626305206301
O	-2.05857929863371	1.66849307546418	0.27656306363604
H	-3.40183355735364	1.55458614076207	2.31804177580552
N	-4.34210076562444	-0.24942517614131	3.75832567974226

H	-4.71074891426666	-1.04597483296074	4.26353307784243
H	-4.61556148786544	0.68319255339060	4.04127713076774
C	1.59550912605126	-0.25780868994742	1.38495047351139
O	0.73405780568598	0.60990392700484	1.42874218906653
H	2.09708303554090	-0.05422941810233	3.35629441471559
N	3.96274357917583	-1.65524839903418	3.77160825058128
H	3.68866772883311	-1.12916486792515	4.59215994002180
H	4.71194139166676	-2.33221402003645	3.84704741747461

Mechanism path with explicit solvation computed with the approximation M06-2X along with Basis Set 1.

Reactants for TS1

Pt	-0.45974771234604	0.15986635593298	-0.51717188868104
Cl	0.53302015781108	-0.88278612993480	-2.40019592439571
N	-1.24493410015794	0.96241669769030	1.18560561452195
N	-1.08394066850264	-1.70005131301407	0.08703653484821
H	-0.84364761578304	-1.89625682837088	1.06291164536593
H	-0.64412481826368	-2.42627601376356	-0.48564351316432
H	-2.09643503873488	-1.82003029753877	-0.00232363292867
H	-0.74297357629379	0.53810903647721	1.97888777989724
H	-2.24671067625618	0.79487595065561	1.30564353940312
H	-1.09357665043868	1.97418572717313	1.21638946408809
Cl	0.23567366354870	2.33261506042083	-1.13353280619479
O	0.92609853492770	-0.49057481638351	2.55906912957175
H	1.20877002088359	-1.17526148449131	1.92959719617037
H	1.43538377058523	0.26845138214185	2.21064599220515
O	2.23196675331201	-1.60459721440131	0.24613540470485
H	1.88156777820584	-1.56967897130507	-0.66266525498103
H	2.95091965923452	-2.25047822169753	0.22019465376488
O	2.51073864774717	1.11524369061946	0.93625342511867
H	1.96244993546825	1.61594353265113	0.31004061826614
H	2.55747193505275	0.22198385713836	0.54502202241913

TS1

Pt	-0.94801269989367	-0.11285094104876	-0.31646665746795
Cl	0.39761748059175	-1.15346824875400	-1.97109619113243
N	-2.10255830703611	0.80379051826378	1.09205029563321
N	-2.45073816960044	-1.34789314911705	-0.87423535808158
H	-2.79581079549938	-1.92209626556239	-0.10032985347470
H	-2.12777261904335	-1.98713893508727	-1.60619084635475
H	-3.25206268929616	-0.83506082282854	-1.25211031734765
H	-1.83238215504975	0.51267215267656	2.03547946182882

H	-3.11204285633863	0.66261979003823	1.00770175239992
H	-1.92216975372919	1.81095276423473	1.01890468107512
Cl	0.02030532469231	2.48969411062267	-0.66957429666935
O	0.85049930445395	0.37079069955845	1.31463313188761
H	1.57176483813821	-0.24766072803443	1.07621053795463
H	1.13512017034471	1.22990813125063	0.95923683436966
O	2.73381522576147	-1.14451029416218	0.07470471419318
H	2.19017903648558	-1.43968872743813	-0.67957959740498
H	3.29957181551719	-1.88991782677753	0.31630376248548
O	3.09290331558208	1.64123796046016	-0.44164352426676
H	2.19085223975713	1.87054003714281	-0.72231052560454
H	3.06092129416230	0.68807977456225	-0.26168800402294

Products for TS1

Pt	-0.58215935032425	-0.17433332805694	-0.09077071004133
Cl	-1.32803727014650	2.06941690440837	-0.09444092883839
N	0.04691599734559	-2.12493927111611	-0.08190129435606
N	-2.11702387167620	-0.63975189059485	1.13987549312041
H	-2.76362259863425	-1.31475463540416	0.72116965766075
H	-2.66305084172580	0.19708963078062	1.36468427613413
H	-1.80429470932975	-1.03932162938993	2.02974116352518
H	-0.31364013561803	-2.64567122910951	-0.88610039562142
H	-0.23997207737374	-2.64214871327923	0.75297788684323
H	1.08123140080795	-2.16399759689761	-0.12510598743716
Cl	3.17215408174177	-1.44392763560741	-0.34965998503934
O	0.98099216574825	0.28690296934468	-1.40299045513257
H	1.28507073993567	1.21621189092815	-1.22967676143429
H	1.77548483827055	-0.28171976803081	-1.19080205919537
O	1.66331842324695	2.68836097168252	-0.52463822325113
H	0.75412434554049	3.01504575203866	-0.39938260178462
H	2.13762570188127	3.37491546744360	-1.01417589808600
O	2.16067002460318	0.85349421566183	1.65455237813072
H	2.48752447607657	0.11599237864256	1.10654685261130
H	2.04470665963027	1.56585151655556	1.00582359219197

Reactants for TS2

Pt	0.48752441363045	-0.23232919977025	-0.17662075353075
N	0.97759831762504	1.60381518875016	-0.85627028635860
N	0.99273299288413	-1.07411752247694	-1.98066402698446
H	0.71696686054839	-0.48979661530684	-2.77522171554163
H	0.55066924542459	-1.98408313603617	-2.13810168147041
H	2.00293497315187	-1.22322449900273	-2.06038653651183
H	0.10089055330368	2.15167397021724	-0.88443449020554

H	1.40618604348090	1.63247403972278	-1.78453412814638
H	1.62032688457458	2.07613536121794	-0.21467433320440
Cl	-0.11193784731130	0.75636051929387	1.88557656890004
O	-1.77208133753119	2.34578297941967	-0.41637357899548
H	-2.10843955612860	1.50075905504469	-0.77065091062762
H	-1.55278039651092	2.11469032277298	0.50008632649755
O	-2.83858614490891	-0.15064242743782	-1.20325859345831
H	-2.30581772599805	-0.62660644765413	-1.85853452635774
H	-3.72312192836821	-0.08878115731889	-1.59301711010120
O	-2.43711008100060	-1.39993337216064	1.23047039417210
H	-2.17701739082241	-0.62420516316144	1.75507916082454
H	-2.71383434040021	-1.01459652606088	0.37067537599563
O	-0.07048200530040	-2.09215926946528	0.62210935473437
H	-1.04395932201715	-1.96748851344270	0.90353047569988
H	-0.04097220832565	-2.85657758714457	0.02765501467033

TS2

Pt	1.02579115669445	0.02798589465141	-0.22678293085618
N	2.01692755518043	1.76872539205709	-0.44509320670762
N	2.41374242294911	-0.97493728465283	-1.32162271549612
H	2.57126216137909	-0.53924788278904	-2.23490566305054
H	2.13267780054444	-1.94207332915060	-1.50695943495082
H	3.32123190851786	-1.01793612032816	-0.84919000698432
H	1.54660553841422	2.38518993539848	-1.11496421042941
H	2.99013632674235	1.68370663384640	-0.74973574104490
H	2.02355735686352	2.25439653004755	0.45836019927225
Cl	0.42945395793820	0.94755985913577	2.33742508391579
O	-0.99165866789344	1.43564020244501	-0.22948280474879
H	-1.84086663698131	0.95701443436729	-0.31768108558772
H	-0.93493950658493	1.65690527370717	0.71778798330282
O	-3.33207386258747	-0.04316516981807	-0.09863074860024
H	-3.56398127839887	-0.65119259695761	-0.81660812254603
H	-4.14412677725156	0.45544194492860	0.07588432320329
O	-1.72995675857807	-1.24193874693170	1.78113813133358
H	-1.22342044498589	-0.50665478533977	2.18548407347680
H	-2.44421600529299	-0.81087567259913	1.26671365765709
O	-0.02740961265163	-1.75405169232940	0.01357302902329
H	-0.77339215770825	-1.62317984727008	0.72079461562981
H	0.53465552369074	-2.46731297241838	0.35449557418797

Products for TS2

Pt	-0.85752734767624	0.03134606334552	0.02188626693070
N	-1.90656817408115	1.22789496228717	-1.22035154216812

N	-2.50596410285669	-1.06771286388695	0.45880036266593
H	-3.30404738017695	-0.48064627556309	0.71913317483220
H	-2.35093315034890	-1.70848094486015	1.24276099630426
H	-2.80920320165628	-1.64096392860922	-0.33455558027358
H	-1.27966328625092	1.92450761236175	-1.63497085510110
H	-2.65497469744887	1.74265353822897	-0.74698388152260
H	-2.33797508893268	0.71533935409091	-1.99516498677545
Cl	2.62128315560512	-0.71245713183708	-1.68868170362953
O	0.77763902909357	1.20693290640265	-0.47699443232559
H	1.23763099514405	1.62185359723683	0.29499160131348
H	1.46031792950655	0.64088467317715	-0.96086008293267
O	2.09534625937326	2.08274504448693	1.68147433896953
H	1.57184103063542	2.27147308000712	2.47520638769884
H	2.71493919635755	2.82386983494317	1.60378563616782
O	2.64848793431440	-0.61874458743600	1.36927526451396
H	2.83862891400820	-0.70645702624266	0.40464054171729
H	2.69519775261134	0.33982242945882	1.55282241450653
O	0.21035580473395	-1.17514037496997	1.33882295777244
H	1.22700116346005	-0.96322069977892	1.40229014555288
H	0.15032926458524	-2.11489926284298	1.10620997578274

Reactants for TS3A

C	3.323020000	-1.475279000	-0.547816000
N	2.970379000	-2.121251000	0.606528000
C	1.868260000	-1.481783000	1.100674000
N	1.494099000	-0.469867000	0.361963000
C	2.383655000	-0.448776000	-0.679751000
Pt	-1.266943000	0.415028000	0.197989000
Cl	-0.537490000	2.641007000	0.589394000
N	-1.648641000	-1.547233000	-0.238726000
N	-2.192228000	1.046143000	-1.478465000
H	3.436163000	-2.921860000	1.022779000
H	1.366726000	-1.808921000	2.010584000
H	-1.959174000	0.537856000	-2.335124000
H	-2.002217000	2.038850000	-1.644994000
H	-3.203831000	0.958692000	-1.341136000
H	-0.964594000	-1.875231000	-0.927783000
H	-2.576004000	-1.743185000	-0.622069000
H	-1.527845000	-2.135575000	0.590749000
O	-0.672319000	-0.261054000	2.127599000
H	-1.550789000	-0.181159000	2.619650000
H	-0.031237000	0.319204000	2.565199000
O	-3.463252000	2.457122000	1.756420000
H	-2.592583000	2.708160000	1.397652000

H	-4.042801000	2.461421000	0.982019000
O	-3.101590000	-0.005067000	2.825659000
H	-3.297448000	0.894960000	2.466563000
H	-3.467037000	-0.017890000	3.719391000
C	2.491602000	0.368248000	-1.830765000
C	4.333645000	-0.899011000	-2.423668000
H	5.130584000	-1.047036000	-3.159769000
H	0.990439000	1.692617000	-1.371917000
H	1.807059000	1.961020000	-2.890910000
N	1.622261000	1.360696000	-2.095516000
N	4.326215000	-1.735607000	-1.394616000
N	3.489748000	0.108784000	-2.686328000

TS3A

C	3.23542500052666	-1.43197541223195	-0.51108624086210
N	2.87507403850802	-2.06110951242776	0.65088452853783
C	1.74033870308855	-1.46061459754518	1.10231270305322
N	1.34325532681862	-0.48141922928762	0.32370326222333
C	2.26858995908906	-0.43924222394620	-0.69677381575959
Pt	-1.01053815694051	0.38106983585393	0.05884070941453
Cl	-0.38180303766908	2.61893073305613	0.50968543977964
N	-1.53967524133080	-1.55778715375470	-0.30160892450045
N	-2.31204465727974	1.05237578289168	-1.33482653344914
H	3.35993518908189	-2.83854452265544	1.09046231629034
H	1.22518259017871	-1.77493599077619	2.00737448377940
H	-2.17783095828431	0.60206853499060	-2.24444286942276
H	-2.18694918950543	2.05931130073800	-1.47552904674620
H	-3.28804113525293	0.90450921842757	-1.06288043198279
H	-0.92018089412248	-1.98952482265188	-0.99362304781810
H	-2.49750343114331	-1.68532132531614	-0.63888465465043
H	-1.45135870864997	-2.10664640741398	0.55921298286325
O	-0.60795038744692	-0.35071794288596	2.34658165771159
H	-1.54664929637072	-0.23842207050462	2.65900958961691
H	-0.09521996532091	0.32999296433464	2.80404197214381
O	-3.37703950976772	2.46919072905718	1.73579560914198
H	-2.52584424166512	2.65856455727091	1.30374062621976
H	-4.01877668622198	2.50587694925811	1.01241898888689
O	-3.16789099060844	-0.02010540995293	2.78594703319595
H	-3.30231194930009	0.89261512718120	2.43778991673154
H	-3.52083076143056	-0.00535123418107	3.68504342465996
C	2.42404602209231	0.37872272815188	-1.84517736968134
C	4.29251160422493	-0.88547764363696	-2.36846419252141
H	5.10354819682897	-1.04494402446004	-3.08721916459773
H	0.94236834367346	1.74254167299798	-1.44517559020380

H	1.81223900521742	1.97386317933529	-2.93890221128046
N	1.58727661305673	1.38627952786942	-2.14447331398422
N	4.26939564681122	-1.69373980905894	-1.31753008396011
N	3.44925295911451	0.10996649127304	-2.66624775282930

Products for TS3A

C	-3.064119000	1.698852000	-0.109074000
N	-2.180834000	2.666199000	-0.517866000
C	-0.945081000	2.127095000	-0.547412000
N	-0.973579000	0.857233000	-0.178400000
C	-2.290160000	0.557794000	0.107482000
Pt	0.622919000	-0.368204000	-0.094926000
Cl	1.245485000	0.582758000	1.937157000
N	0.109661000	-1.161133000	-1.903223000
N	2.277609000	-1.556994000	-0.065769000
H	-2.409849000	3.626494000	-0.759946000
H	-0.031171000	2.659460000	-0.821107000
H	2.178239000	-2.445499000	-0.559717000
H	2.576978000	-1.774887000	0.887043000
H	3.040384000	-1.015031000	-0.512220000
H	-1.016298000	-0.966294000	-2.123069000
H	0.318128000	-2.319828000	-1.916148000
H	0.773907000	-0.639568000	-2.708022000
O	2.169488000	2.611576000	-0.588104000
H	2.806132000	1.931404000	-0.889331000
H	1.953200000	2.293265000	0.301531000
O	4.448544000	0.658596000	1.656259000
H	3.503615000	0.654401000	1.900979000
H	4.772258000	-0.192892000	1.981043000
O	3.978379000	0.527130000	-0.964066000
H	4.309259000	0.612192000	-0.031986000
H	4.767024000	0.525403000	-1.523058000
C	-2.967846000	-0.603029000	0.557177000
C	-4.914193000	0.647259000	0.467882000
H	-6.000045000	0.646560000	0.622525000
H	-1.337846000	-1.827631000	0.737785000
H	-2.884116000	-2.546819000	1.155255000
N	-2.348924000	-1.757470000	0.814561000
N	-4.382498000	1.794247000	0.056243000
N	-4.296615000	-0.507965000	0.721007000

Reactants for TS4A

Pt	2.452383000	-0.054121000	-0.140791000
N	3.886396000	1.082426000	-1.033078000
N	3.566628000	-1.750249000	-0.379976000
H	3.618934000	-2.053518000	-1.365632000
H	3.134958000	-2.516623000	0.152570000
H	4.537845000	-1.657670000	-0.040144000
H	4.192106000	0.710392000	-1.971049000
H	4.752659000	1.161393000	-0.437831000
H	3.526949000	2.041659000	-1.180972000
Cl	1.198007000	1.886283000	0.155054000
O	-0.881128000	-1.754595000	-0.779998000
H	-1.352388000	-0.869329000	-0.682028000
H	-0.710533000	-1.865768000	-1.728467000
O	1.082263000	-1.268211000	0.792832000
H	0.380245000	-1.557906000	0.121262000
H	0.536446000	-0.748686000	1.464814000
N	-2.429318000	0.355447000	-0.269144000
C	-3.738427000	-0.027392000	-0.070171000
C	-2.452766000	1.674072000	-0.279091000
C	-4.556052000	1.103122000	0.048048000
H	-1.579100000	2.314589000	-0.415564000
H	-3.958886000	3.157243000	-0.052589000
N	-3.704553000	2.172052000	-0.089785000
O	-0.453479000	0.267904000	2.295490000
H	-1.351222000	0.117599000	1.796116000
H	-0.063738000	1.070264000	1.849668000
C	-4.378714000	-1.292201000	0.014638000
C	-6.364369000	-0.134431000	0.315241000
H	-7.447574000	-0.217647000	0.474742000
H	-4.238306000	-3.318092000	-0.056683000
H	-2.715320000	-2.459833000	-0.286983000
N	-3.718981000	-2.450182000	-0.095660000
N	-5.709967000	-1.296027000	0.211332000
N	-5.877600000	1.103652000	0.245866000

TS4A

Pt	1.60794770713145	-0.03997002473471	-0.18170434976306
N	2.07903352971838	1.26371598217480	-1.65440006753383
N	2.61941665230795	-1.56339228031687	-1.09156130106768
H	2.26520635326834	-1.75883545573879	-2.03162581492345
H	2.51764676486194	-2.42745741990199	-0.55131847435120
H	3.62281739751824	-1.38189462587837	-1.17731902408582
H	1.34851739083393	1.31066886408774	-2.37100641432959
H	2.95659244760294	1.05714295174599	-2.13902675149131

H	2.17407471072085	2.20353155682779	-1.25616419682140
Cl	1.77458280202271	2.01270946862218	1.59752188393848
O	-0.43909404055672	0.13054315263324	2.77297733729238
H	0.08208709257960	0.91492801253456	2.50021068583910
H	-1.31201921537863	0.28055806005915	2.38215318935108
O	1.12886001644492	-1.35602159664386	1.36514701235684
H	0.40105000870323	-0.90659898889747	1.90378634609303
H	1.91868204500741	-1.28106687794857	1.98352162524587
N	-0.70172340741554	0.88313250478643	-0.14388176801814
C	-1.80887651164421	0.20619642680601	-0.60847387496753
C	-1.03517658718047	2.15182841070365	-0.17525921902522
C	-2.82292171113694	1.11353393701443	-0.92709021201496
H	-0.38566813491819	2.96888805756302	0.13579337055288
H	-2.76623472080064	3.24192233093958	-0.75142139970988
N	-2.29939386554780	2.34619372394297	-0.63736224073841
O	3.12641158526667	-0.58897596351361	2.80783933745553
H	3.10944240758808	-0.71645558894231	3.76648933378984
H	2.87576282675981	0.34624734076784	2.66662928315687
C	-2.11083022165349	-1.16675276780749	-0.80120472389274
C	-4.18993849150777	-0.49456959090655	-1.56938070665930
H	-5.15543110203770	-0.82358335234524	-1.96829217448134
H	-1.57624926704310	-3.11311656386664	-0.62235506255511
H	-0.40689051059576	-1.99340559162210	0.01325246312766
N	-1.24704098091448	-2.15910198221073	-0.52983754160468
N	-3.31855247551741	-1.47268733503559	-1.29620868110745
N	-4.03209049448762	0.81214522510151	-1.41042786905747

Products for TS4A

Pt	0.58338639936222	-0.99553848713756	0.16136355773850
N	-0.17079334193220	-2.46929145002263	-1.01170391278258
N	2.05323553054401	-2.25836883435713	0.86008764774034
H	1.82246328496323	-2.63339526790280	1.78415839708060
H	2.93760157027642	-1.73908112695752	0.94857035354740
H	2.23622173694503	-3.06320507179142	0.25505593472485
H	-1.01742083535612	-2.17658618406179	-1.50787452836844
H	-0.42021506981285	-3.30459674973974	-0.47402916481878
H	0.49783650458307	-2.76720428454246	-1.72847567460812
Cl	2.63484816295197	1.06213428381265	-1.88027521251601
O	1.14879770092582	2.75275888068964	0.21246637141834
H	1.59134616909866	2.38301244836451	-0.58736773281770
H	0.32538992660383	3.15331167733029	-0.09813033553461
O	1.33687249568159	0.47849750855013	1.43023063647789
H	1.14230246968550	1.39460404758960	1.06320388569431
H	2.32998271990854	0.40978599057675	1.38286549293040

N	-0.85492564946197	0.28126953017626	-0.52284835119636
C	-2.09931322157520	0.52988355093503	0.02680314007706
C	-0.75929092717077	1.04700442107910	-1.59497896692363
C	-2.75064600381563	1.48287891128828	-0.75498603245255
H	0.12574853377622	1.09788485175114	-2.23134874486603
H	-2.03117928488889	2.44837690320366	-2.52427089561951
N	-1.87298267249739	1.78647185136972	-1.76760258433773
O	3.82612208238750	0.05820816325006	0.75693767211148
H	4.62225664223837	0.49285207721849	1.09142457295832
H	3.64803360791677	0.44451477462834	-0.13501201550114
C	-2.81020586167633	0.03501365963429	1.15290765787457
C	-4.54138222199319	1.44416293220162	0.52940477867291
H	-5.55045870707121	1.79544918138140	0.76833950830278
H	-2.88855268612701	-1.19196687843100	2.77047928445751
H	-1.39490426287947	-1.27956403444902	1.86061561619889
N	-2.31903860490555	-0.88293216273921	1.99136660891121
N	-4.03909323964439	0.52800171607247	1.36282851649108
N	-3.97227594704055	1.97533817102884	-0.54476648106526

Reactants for TS5A

C	2.93994897684871	-0.22043951341216	-0.03507520274201
N	1.89885549689317	-1.02642438310784	0.37572207643197
C	2.40490231014074	-2.23776204969200	0.45204025348257
Pt	-1.89768969531278	-0.38865024877354	-0.05099239673568
N	-2.87083722767174	-1.91458925905245	0.86027469510910
N	-3.33405767321988	-0.29115754190169	-1.47033913448818
H	-3.36250566842650	-1.13172784463919	-2.05630019111380
H	-3.19561925295022	0.50183106314744	-2.10383258838800
H	-4.26908549912453	-0.17658394990140	-1.06619676657714
H	-3.53889010587079	-2.39868804114898	0.25286338054794
H	-3.40311444706757	-1.58578926300413	1.67227441332864
H	-2.22538860269800	-2.63349816773301	1.20295467820528
H	1.85536811850765	-3.12750912023828	0.75504834445587
H	4.31223406410585	-3.09541485261081	0.10309509169548
O	-0.38015669662617	-0.39881490270927	1.38421579248470
H	-0.51437004829456	-0.96260966600196	2.16292086239105
H	0.55172568622451	-0.66181278447146	0.96263896064939
O	-0.84116532781221	1.18015909743151	-0.91841770979553
H	-1.20101474469759	2.09886680361476	-0.55757475717972
H	-0.80276845371816	1.20352479574455	-1.88765902807440
O	0.09262390107025	2.36297902827333	2.09594590954266
H	-0.08245004712852	1.40519622403422	2.05416867326445
H	0.96785119369509	2.44752218036763	1.68304605507939
O	-1.55716573348659	3.24228950475806	0.22645178577171

H	-1.01442600124752	3.04109586498888	1.03698323758138
H	-2.48228136817993	3.23307307898186	0.51097774163318
N	3.71598274903839	-2.27151751869658	0.11481807404003
C	4.09384617292957	-0.99062029763596	-0.19718741263265
C	3.06264966037269	1.15856165591725	-0.32822354503600
C	5.29776340478547	0.76657646267709	-0.77390348197966
H	6.25337633154384	1.21095003799079	-1.07019935915665
H	1.08201803427609	1.63272429141658	-0.37244247309120
H	2.17157042513593	2.93825078152166	-0.63811315241886
N	5.29503031794923	-0.54311867022559	-0.56665365283941
N	4.26715800445268	1.61690546084438	-0.67955628772408
N	2.01764174556335	2.01606174324628	-0.23974288572184

TS5A

C	1.91197902526398	-0.45471252435284	-0.35087610280017
N	0.74766424935484	-1.20372041324503	-0.38112362739197
C	1.14931609933198	-2.45281396802613	-0.31093455068381
Pt	-1.81476019428073	-0.90438626496019	-0.46585954573024
N	-2.22027485965132	-2.56156892012224	0.59576613332603
N	-3.40778484035792	-1.26974953246316	-1.65203473878681
H	-3.12371839617077	-1.70904591013592	-2.53345856741714
H	-3.89949079137856	-0.40319734523159	-1.89239503925609
H	-4.10651197466776	-1.88630812393838	-1.22669744790040
H	-2.15869030220893	-3.41179584260119	0.02525390052554
H	-3.16221167799761	-2.54039609455095	1.00056538526496
H	-1.57082779442128	-2.68536437390521	1.37970750885563
H	0.50005029549056	-3.32785688480679	-0.31221666798736
H	3.02396934869572	-3.43929290793561	-0.16611113690190
O	-0.84459863077206	-0.03557265029802	1.58921213720495
H	-1.41187876142292	-0.21044612759022	2.35788382407994
H	-0.01158824431200	-0.49706941317050	1.78084715138310
O	-1.38023366850580	0.85390115123744	-1.56253047960548
H	-2.07050064304560	1.54073631524847	-1.54721798155525
H	-1.17132533293106	0.70396298776539	-2.50198410123651
O	0.12171795105474	2.64720515609215	2.11930933048289
H	-0.33563523876452	1.79350492713485	2.04741535591646
H	0.69647596447555	2.62666331650339	1.33728409255068
O	-1.69861030419643	4.62641867822699	1.36939461735912
H	-1.08739333853675	3.92117190855384	1.65756981888957
H	-1.31408580075561	5.41792348150046	1.76439748746664
N	2.49852620054402	-2.57128884349491	-0.23231746374495
C	3.01960028551736	-1.30635089831989	-0.25120943437491
C	2.21067960277644	0.92964885412430	-0.42838541572449
C	4.44980800877428	0.37321617766611	-0.24601414777771

H	5.47547094057014	0.75237212014842	-0.19470546469114
H	0.33188326349214	1.62605433007612	-0.84767816196380
H	1.59358015223047	2.80942415908809	-0.82407296552179
N	4.30153390487202	-0.94267481279704	-0.19174573467603
N	3.49341507633803	1.30054852850441	-0.36421332784058
N	1.26445042559534	1.89085976007534	-0.54082463973697

Products for TS5A

C	1.82834170101334	-0.18545431689837	-0.16296024184717
N	0.71497056679955	-0.69902106092481	0.47818764718717
C	1.15477603625467	-1.25816037474616	1.59131158435109
Pt	-1.25344938474998	-0.44539452673102	-0.01365832327795
N	-1.52227512750298	0.64089522346311	1.65155221842335
N	-3.23239976932685	-0.15289510489549	-0.48409200869548
H	-3.85574752731938	-0.65243357854031	0.15635340139158
H	-3.49095667996511	-0.44575656325703	-1.42953177839536
H	-3.44161447002819	0.85568976340333	-0.40497324761965
H	-1.95767594608051	0.09750737149945	2.40234563959643
H	-2.12556841447597	1.45394684921022	1.44135613906242
H	-0.62154003343039	1.00223889484669	1.99356331308614
H	0.52765816642162	-1.74797003056207	2.33457596633889
H	3.04863197701632	-1.49145207241441	2.48753766242448
O	1.07174688434235	2.04168831828389	1.87695414543333
H	1.02909787515124	2.69179149315557	2.59476117820426
H	2.02033150091420	1.95223887749328	1.70255627391114
O	-0.87169223013107	-1.54574059836628	-1.79164147564803
H	-1.60687366348450	-1.73214891564777	-2.39936434446680
H	-0.41105707751157	-2.39219063977679	-1.65611738825950
O	-0.28424814040363	2.90413581064590	-0.43113699835031
H	0.22849246899731	2.66525893651141	0.36655175391796
H	0.08490650460907	2.34706813508800	-1.13692814908489
O	-2.89249326709142	2.59408339934079	0.11778626258669
H	-1.97282759922760	2.81123742475506	-0.17245525381658
H	-3.32924036899457	3.43718616529856	0.29154081713606
N	2.48953660879504	-1.14113143577011	1.71179987640394
C	2.95436876917041	-0.45247731329990	0.61974042773074
C	2.05471946047397	0.52964701177055	-1.36452042468341
C	4.28899910447113	0.62426698295407	-0.76729917651096
H	5.28389474355967	0.98649956893802	-1.04304602368600
H	0.27329007227320	0.21929511866794	-2.30771314897174
H	1.37841510536280	1.25206091540519	-3.11506678614018
N	4.20282675049448	-0.07362448039021	0.35617138881306
N	3.30049454959784	0.93267358722299	-1.61624390432433
N	1.06217285400548	0.85902916426666	-2.23217102222041

Reactants for TS6A

N	-1.88951952673746	-2.85987371289252	-1.67491140446990
C	-0.95300100980966	-1.89926130205362	-1.53467673880204
N	-1.28466665424211	-1.03615475592321	-0.59299701663988
C	-2.51403434548827	-1.44523120437814	-0.10655825558792
C	-2.89426402867215	-2.60958979678333	-0.77429686175123
Pt	-0.20591195736737	0.58750040319391	0.02407213954546
Cl	-0.24885298838010	1.38827598856778	-2.18713526592167
N	0.78366299812204	2.27258539407638	0.67564549886141
N	-0.10666625170986	-0.19995430968058	1.93624371619524
N	2.25643168287726	-1.93809607807470	-1.19783574993863
C	1.95463115102308	-2.93761176271271	-0.40652491168201
N	2.25110464722763	-2.69297235097737	0.90569118128027
C	2.78819219337165	-1.43814618629723	0.96320456824674
C	2.78280198486822	-0.98773115654204	-0.35822284333410
H	2.11192321748576	-3.32895529794435	1.68616008554324
H	1.50978924115075	-3.88128544741664	-0.72166903694440
H	0.80645476373128	-0.04580197217624	2.37752830011868
H	-0.25229497541938	-1.21431967320732	1.93808308343766
H	-0.81017417564350	0.19341581858436	2.56900369897856
H	1.76638083004322	2.08967214927459	0.90612988100317
H	0.35072637041773	2.66324236052049	1.51662405442208
H	0.80291005181166	3.02143357235243	-0.03377541465640
H	-0.03761555008665	-1.85681047692080	-2.12193582937689
H	-1.85029531497954	-3.63389374364700	-2.33444759048945
O	3.93444078187025	3.60404142733765	-0.90888464067136
H	3.89642025237871	2.96058883856053	-0.17898821766775
H	3.01055695488445	3.87706527651424	-1.05480109077541
O	1.23126059790842	4.11278775482914	-1.52413229004766
H	0.72395666270667	4.92776528946643	-1.64216069027300
H	0.78532142062669	3.44867616863722	-2.07925933998633
C	3.64699774558017	0.45615396618517	1.71447198573545
H	4.01039784224152	1.08214598211399	2.53572283971473
C	3.24302196978096	0.33569776069413	-0.57521363592212
N	3.20779442731641	-0.75066812575877	2.03733437394859
N	3.67840907025598	1.02528273588367	0.49907628488087
N	3.25509269431595	0.91187405860765	-1.77894139265138
H	3.51200512530117	1.89891656569301	-1.83683721328586
H	2.79303995139884	0.45489825690702	-2.55487108575367
C	-3.43578790074687	-0.93141657487760	0.84223001052467
C	-4.75819603739531	-2.77905146831208	0.39410232430453
H	-5.67755534880210	-3.31663608683900	0.64681016944396
N	-3.24379583492035	0.21796897288826	1.51728151232074
N	-4.00510057862786	-3.31352471258711	-0.55790975786396
N	-4.53799215913712	-1.65113521023561	1.07841341004631
H	-2.60084746798299	0.91301029058955	1.14912011312734

H	-4.01587252254799	0.55634237476038	2.08211704281330

TS6A

N	-1.01533095336322	-2.99164434578107	-0.80979401017214
C	-0.53066351299775	-2.13269892613880	0.10768618245343
N	-1.31037506867203	-1.07692054873328	0.24362227362747
C	-2.36559743586070	-1.25661100224020	-0.63358405749910
C	-2.18704432616706	-2.47040956821449	-1.29865206890911
Pt	-0.88361623148116	0.52203963383037	1.43826591945264
Cl	-0.99929599996985	2.27230549698904	-0.65857868483964
N	-0.42255279532424	2.13359957513834	2.62368725668852
N	-1.97439194543138	-0.29242194809773	2.96067667055383
N	1.45342823048321	0.40938131437316	0.54299746154050
C	2.38240610291116	0.37451597875452	1.46898988329026
N	3.52084751910430	-0.23765082097511	1.05039928416094
C	3.32130925185914	-0.61492593017357	-0.24984746918466
C	2.02081261212696	-0.20186657137087	-0.55589738689121
H	4.36503290956236	-0.38057637176384	1.59791668571703
H	2.27759227245623	0.77049989141860	2.47850988920890
H	-1.41715693627998	-0.41893303639783	3.80977133139635
H	-2.34360399525245	-1.21358868458573	2.70997856804168
H	-2.77793070325900	0.29023208941433	3.21045551803556
H	-0.08732887881453	1.87770333116061	3.55600905083493
H	-1.23046998748147	2.74699912566405	2.75976343345132
H	0.31444270341754	2.69993031090054	2.17415653085328
H	0.39048195864736	-2.30451789421106	0.66165030411301
H	-0.58937367026349	-3.87660973447885	-1.07657347990038
O	3.92343417178798	2.48256934760274	-0.31927370891786
H	4.42709050590429	2.11450508637875	0.41606998859550
H	3.11023659534570	2.81220701465703	0.10675387147019
O	1.51512342109073	3.40106088644567	0.80182533362780
H	1.38690891807911	4.35713801483570	0.71917089042760
H	0.86454978483107	3.01632375912522	0.17591332222338
C	3.63291887712412	-1.47624442953782	-2.25520500788858
H	4.26541977913574	-2.00001086231035	-2.97996213547287
C	1.56944616675208	-0.49264949552581	-1.87075214202410
N	4.16900238127195	-1.24923773942435	-1.06373674111947
N	2.41307801002326	-1.14244375912600	-2.68834285343823
N	0.34305393906547	-0.17241251702940	-2.31478188991004
H	0.15142139540098	-0.33074394265507	-3.29780591122891
H	-0.20041198570355	0.54055745536636	-1.82239257755551
C	-3.54114363328256	-0.52585348894847	-0.95547550112921
C	-4.08382657076312	-2.25400434735995	-2.40326869568937
H	-4.81586788316989	-2.63707195235560	-3.12154793673076

N	-3.86336824084404	0.64999974921671	-0.39120665347254
N	-3.01211384641710	-3.00762924853082	-2.19718287424540
N	-4.38010847271360	-1.07755163766494	-1.84250198846388
H	-3.11354928976328	1.23791262053711	-0.03162942721694
H	-4.67290514310540	1.12574812182218	-0.77625644786419

Products for TS6A

N	-3.159920000	0.786927000	-1.212105000
C	-1.843603000	0.849135000	-1.491545000
N	-1.147116000	0.022770000	-0.732330000
C	-2.060993000	-0.612971000	0.095870000
C	-3.338625000	-0.137366000	-0.211383000
Pt	0.885022000	-0.229528000	-0.836084000
Cl	2.270778000	-1.293789000	2.203015000
N	2.917418000	-0.482456000	-0.886589000
N	0.646919000	-2.216848000	-1.321612000
N	1.103442000	1.785527000	-0.495260000
C	1.504876000	2.637867000	-1.422512000
N	1.283965000	3.914218000	-1.053455000
C	0.686984000	3.899577000	0.182934000
C	0.592329000	2.552977000	0.537759000
H	1.506738000	4.739777000	-1.605778000
H	1.943662000	2.358272000	-2.379113000
H	1.283736000	-2.860610000	-0.719431000
H	0.959278000	-2.342583000	-2.335570000
H	-0.360206000	-2.563320000	-1.242409000
H	3.196934000	-0.653803000	0.091366000
H	3.436220000	0.333501000	-1.226702000
H	3.207610000	-1.303923000	-1.445249000
H	-1.425062000	1.507662000	-2.252372000
H	-3.890903000	1.330233000	-1.667034000
O	3.421433000	-3.065964000	-2.059129000
H	3.134170000	-3.621803000	-2.798533000
H	3.206151000	-3.576516000	-1.244451000
O	2.371254000	-3.803042000	0.407598000
H	1.805463000	-4.527304000	0.791302000
H	2.345007000	-3.085335000	1.104581000
C	-0.308580000	4.555580000	2.036926000
H	-0.715253000	5.350559000	2.670324000
C	0.039362000	2.272763000	1.816447000
N	0.257799000	4.938371000	0.899217000
N	-0.431761000	3.314358000	2.516462000
N	-0.044834000	1.038055000	2.336922000
H	-0.372023000	0.983126000	3.296715000

H	0.619211000	0.312578000	2.050206000
C	-1.982827000	-1.586524000	1.134251000
C	-4.296871000	-1.438394000	1.290235000
H	-5.194901000	-1.807081000	1.799488000
N	-0.848265000	-2.154896000	1.568703000
N	-4.486022000	-0.513247000	0.356065000
N	-3.141672000	-1.973016000	1.691883000
H	0.084894000	-1.767489000	1.394730000
H	-0.934331000	-2.775575000	2.368813000

Reactants for TS7A

N	-3.273422000	-2.825826000	0.209162000
C	-2.193452000	-2.195782000	0.707742000
N	-2.159391000	-0.927822000	0.332945000
C	-3.283839000	-0.720344000	-0.450839000
C	-3.988481000	-1.922451000	-0.539314000
Pt	-0.602709000	0.321838000	0.737890000
O	0.294550000	-0.203410000	-1.076762000
N	1.049307000	1.496512000	1.139640000
N	-1.472495000	0.880748000	2.477740000
N	2.649909000	-0.989949000	-0.419622000
C	2.739633000	-2.180335000	0.148112000
N	4.013863000	-2.496827000	0.479349000
C	4.811410000	-1.436713000	0.105201000
C	3.940715000	-0.495315000	-0.457840000
H	4.318572000	-3.363941000	0.924992000
H	1.904455000	-2.858780000	0.341501000
H	-0.823026000	0.877510000	3.269906000
H	-2.251780000	0.268244000	2.735463000
H	-1.855107000	1.829366000	2.409979000
H	1.851689000	0.920092000	1.416569000
H	0.907347000	2.175562000	1.893527000
H	1.332461000	2.033555000	0.309428000
H	-0.161269000	-0.909567000	-1.564482000
H	1.284075000	-0.516867000	-0.895476000
H	-1.447221000	-2.683145000	1.333621000
H	-3.511183000	-3.804840000	0.357933000
O	-1.317602000	3.409118000	-0.234553000
H	-0.493265000	3.163432000	-0.697268000
H	-1.032993000	3.657662000	0.655907000
O	0.889666000	2.394344000	-1.696670000
H	0.986576000	2.890753000	-2.522233000
H	0.540012000	1.518881000	-1.944376000
C	6.571047000	-0.140945000	-0.226667000

H	7.649396000	0.040021000	-0.151802000
C	4.530102000	0.722068000	-0.915620000
N	6.133966000	-1.309250000	0.244385000
N	5.860304000	0.850686000	-0.780312000
N	3.839212000	1.729854000	-1.466229000
H	2.826750000	1.712672000	-1.571381000
H	4.346467000	2.563425000	-1.748684000
C	-3.836218000	0.393396000	-1.144988000
C	-5.530037000	-1.039721000	-1.841500000
H	-6.449051000	-1.130024000	-2.431263000
N	-5.115206000	-2.137429000	-1.219642000
N	-4.968197000	0.170912000	-1.836400000
N	-3.311530000	1.619073000	-1.154180000
H	-2.494157000	1.925023000	-0.621156000
H	-3.811232000	2.329733000	-1.678717000

TS7A

N	3.25753104741639	0.03425253611538	1.53588522629187
C	2.33679079001562	0.94798403897851	1.17486800453025
N	1.45770608320191	0.43556368523643	0.33182541159475
C	1.83755572959508	-0.87751867842987	0.11670453134342
C	2.97131627812228	-1.14108678917595	0.88477545020826
Pt	-0.22596271824561	1.40684000715163	-0.27266440175517
O	-0.98442494966161	1.82692762127691	1.98357878939641
N	-1.89279407387933	2.50036560531122	-0.79855612668132
N	0.77052497106206	2.03223207692397	-1.90274472367084
N	-1.43080663091010	-0.74864727045486	0.76507435445641
C	-0.94269193330444	-1.34265929014311	1.82995941581604
N	-1.40151524267376	-2.61391423291212	1.98444262155542
C	-2.24945408860370	-2.86764348924595	0.93841326330059
C	-2.25125973447216	-1.69184679863430	0.18613970606229
H	-1.15827816087161	-3.25248070940555	2.73709885371853
H	-0.23562272165889	-0.90304280002233	2.53371520045905
H	0.17777596895531	2.39890828946771	-2.65088758736290
H	1.35016673852139	1.29350321168952	-2.30975821789129
H	1.38660446024556	2.79022974651320	-1.56305824572771
H	-2.76352693286528	2.12995072267789	-0.40622127805025
H	-2.05172874013613	2.61755827629038	-1.80294684747791
H	-1.75706121124052	3.43884693545945	-0.39495706590147
H	-0.38593209292605	1.60318699711229	2.71369645467724
H	-1.82373714217572	1.38972270877448	2.20044917543637
H	2.33407572325662	1.98675442264325	1.50217954087346
H	4.03792277196718	0.19520155517770	2.16892094734511
O	1.91406673747185	3.77227575451157	0.01930170842031

H	1.07225304945586	4.12195209035663	0.37910809555069
H	2.53144333768500	4.51374426396953	0.05111475066380
O	-0.62009307345696	4.41696453257680	0.94684943984956
H	-0.82239465436281	5.24975723864386	1.39416481888226
H	-0.80955404760320	3.71649156245284	1.59704347410473
C	-3.62709964113717	-3.87716933516593	-0.46076181422471
H	-4.22165174958002	-4.75071476666534	-0.74848231343408
C	-2.99860018290215	-1.72342354373428	-1.01757332237799
N	-2.92981024836658	-3.98473719613943	0.66209803772282
N	-3.68877974906635	-2.83566549633787	-1.29984764928949
N	-3.01718060362398	-0.69502898874527	-1.87770176694639
H	-2.45969123420734	0.13021798451583	-1.70501907184176
H	-3.52731802094202	-0.77784929805396	-2.74868248134784
C	1.38111639165916	-1.93094287839213	-0.72099886419080
C	3.08371650484135	-3.22472607885510	0.17531672314703
H	3.55714360850302	-4.21171351830309	0.19138840070376
N	3.62822234474864	-2.29895648094287	0.95427781202940
N	2.03191749464737	-3.10064747401084	-0.63909891785576
N	0.37408398116830	-1.80788520874133	-1.59205028182202
H	-0.15506960551302	-0.94848554702061	-1.67840154396432
H	0.10712517184661	-2.61087599429486	-2.15014768632582

Products for TS7G

N	3.05202939733652	0.87950656788745	2.12780892504860
C	2.77332681363107	-0.14344756515707	1.29902158792124
N	1.90855657691840	0.21186552151329	0.36534607911240
C	1.61058886900026	1.54545226830814	0.59849526313333
C	2.32245132755917	1.96890734004794	1.72000881942580
Pt	1.11610366134845	-1.10777464874680	-0.98452354078150
O	-5.31929615342022	1.57600842571257	0.53581200717667
N	0.35260406208442	-2.49055976105294	-2.30572226471690
N	2.32285831765679	-0.36817064316596	-2.47636211626228
N	-0.05800024100825	-1.85419831194574	0.51809097632713
C	0.34567208967214	-2.72441418497104	1.42666916550086
N	-0.56690188591706	-2.87190493326772	2.40603146593771
C	-1.62560881902793	-2.04566412497031	2.12850674034322
C	-1.30634054944233	-1.41756196343851	0.92678264558738
H	-0.47784465128640	-3.48706355372208	3.21240832342849
H	1.29490167045677	-3.25711330468493	1.40803753240513
H	3.03217497903769	-1.05332640141609	-2.75463562257829
H	2.82903720941182	0.47737236406578	-2.19844344956513
H	1.79956833008007	-0.12954131346821	-3.32423444308034
H	-0.23537642840974	-3.18548470624699	-1.83662739898120
H	1.08888508227592	-3.01114874404062	-2.79161190777600

H	-0.22974318746918	-2.06028809965546	-3.03096815551306
H	-6.12466348696191	1.08587723873790	0.32089547122196
H	-4.66184869121592	0.87508389847578	0.77565740535861
H	3.20984932165625	-1.13524563798623	1.40411262523676
H	3.69105583914264	0.84358239159858	2.91920276634151
O	-2.00215608724558	4.00861785878149	-1.97044446792666
H	-2.75514001786165	3.38350571065973	-1.90211056467106
H	-1.96162099304768	4.22842704327812	-2.91028975513767
O	-3.98950335143016	2.13129169245355	-1.67092023317542
H	-4.52645696305976	1.84026049441623	-2.42056364056007
H	-4.57827534298156	2.04142279298697	-0.87290141625435
C	-3.53327750922142	-0.95707054438544	2.30880368419203
H	-4.44804055969097	-0.71068201831692	2.85720990449010
C	-2.28103908998950	-0.53085163325544	0.38650481733478
N	-2.72924029503534	-1.85217593333957	2.85569935642769
N	-3.36922741340622	-0.31080573910512	1.14682986005299
N	-2.17074179084882	0.05481008496202	-0.80254438178063
H	-1.39585617484509	-0.17946879392085	-1.41015537247844
H	-2.86691692695116	0.73462713340070	-1.13532684880316
C	0.78184636448097	2.51581387882394	-0.03359124715790
C	1.43782100677764	3.98589547925896	1.63791711805304
H	1.32706035096407	4.99394041365500	2.05104522761128
N	2.27614179195785	3.17960353967516	2.27750860776633
N	0.71349533724796	3.72514427520812	0.54780181664630
N	0.09637523285105	2.29722791884805	-1.15470387699113
H	0.15102295445168	1.40351266865168	-1.62388767466203
H	-0.59201797622570	2.99526455885284	-1.47923581322811

Reactants for TS3G

C	-2.60665853028141	-1.07257580940915	-0.42469317899653
C	-2.46927406738610	0.33649323731678	-0.20549020872919
C	-3.53758344740028	1.03402300996950	0.35283000082443
N	-4.74165038441362	0.55631024101618	0.71331623875056
C	-4.87715659823894	-0.73644710707976	0.50177988146153
N	-3.87346365293789	-1.51355358363459	-0.03026414185859
N	-1.41374190055047	1.19158144317536	-0.42585601680868
C	-1.81826919599080	2.36796935004828	-0.01450144628305
N	-3.09657246192333	2.32150967168188	0.46263839892500
Pt	1.71133768324966	-0.78700449461454	-0.08470931557128
O	0.95915395217092	0.64500620392792	-1.39132532918091
N	-6.02629195519599	-1.35386653975460	0.80596765664423
O	-1.79182631860500	-1.86642100765813	-0.87846751930790
Cl	2.44554038098095	0.82545572658780	1.47129592253623
N	1.03247550284475	-2.16625687750968	-1.43289325940873

N	2.43031069173200	-2.21900979380950	1.15859543331861
O	2.16778643703542	2.97074722293813	-0.76460379224113
O	0.20359856479251	4.91951065993138	-0.16090514214213
H	-3.62644429826894	3.10505517995287	0.83488150745937
H	-1.22444291061575	3.28541410794524	-0.03193908085148
H	1.69090674442416	-2.82512570446599	1.52559782894033
H	2.89508530955527	-1.79603744585217	1.96749609019241
H	3.11646524154889	-2.83030840367877	0.70700484604911
H	0.00240917947190	-2.16549046357298	-1.39045870448445
H	1.35364937382731	-3.12379603267591	-1.27440748136465
H	1.30723246687242	-1.91217309445628	-2.38495047060221
H	0.90320919375825	4.28588733918692	-0.40503884987850
H	-0.14565970494396	5.20499686100999	-1.01488514342994
H	2.46387687544858	2.57931844676302	0.08030497943098
H	2.97723613271355	3.26876910988518	-1.20542581452487
H	1.44519793538917	1.50183037400126	-1.31819614661853
H	-6.16344195265593	-2.34638757832950	0.66535803145831
H	-6.77743481565892	-0.80328842174826	1.20263905532201
H	-4.04085670927807	-2.51013608769607	-0.16065096595213
H	0.01010723852980	0.86172026060817	-1.07192386307825

TS3G

C	2.69162028014253	-0.56373697103871	0.82024535433740
C	2.15217898676311	0.09035188177993	-0.33502286005846
C	2.99874324656291	0.89508278499757	-1.09690279794079
N	4.30152694660809	1.15691138919423	-0.89337114205064
C	4.79794624410838	0.56064368139966	0.17094952122883
N	4.04425705471082	-0.25342007030997	0.98523157419795
N	0.88213731490032	0.09881637670671	-0.87031824617123
C	0.95501657382755	0.87613187096997	-1.92031470640973
N	2.21309930815553	1.37715883234215	-2.10252727004112
Pt	-1.37986789837650	-0.83057740346848	-0.16308784135229
O	-0.51539445244132	0.78832894220511	1.44597334216445
N	6.08672063951672	0.72492060237678	0.49780376538863
O	2.13376395893903	-1.31274221791656	1.61494061491579
Cl	-2.43803222865983	0.83368027483373	-1.49013607364320
N	-0.49374279795317	-2.26948314064302	0.97375208094398
N	-2.82893243335295	-2.13023699551570	-0.69934098513091
O	-2.92676729150822	2.07700403244556	1.37272978816014
O	-2.54729773892587	4.83027102849423	1.96380211339634
H	2.50922057048143	2.00546501542374	-2.84467480724714
H	0.11930881139743	1.12772298397904	-2.57129007732718
H	-2.46751583069113	-2.93505897557225	-1.21834619126041
H	-3.50689070183145	-1.66052189155819	-1.30721990827271

H	-3.34882428348866	-2.49412440372493	0.10388477984340
H	0.45124053573462	-1.95740956492291	1.26128081698531
H	-0.38601947178003	-3.16002929804440	0.48206334303733
H	-1.02868754965738	-2.46400422152116	1.82414695524433
H	-2.73276892507592	3.89764392165970	1.76277110514898
H	-1.60397440487075	4.90729628311149	1.77674846844778
H	-3.02467013189120	1.86423343583940	0.42435823366646
H	-3.65189934596004	1.60590498948357	1.80851819251260
H	-1.32082984928268	1.33316627640354	1.58141775411065
H	6.48587333364723	0.33271502581165	1.34082664630918
H	6.64844588727693	1.34794072618978	-0.06892446433769
H	4.48987751092021	-0.69713462687588	1.78684785766783
H	0.10455813205425	1.37027942546461	0.98500506353613

Products for TS3G

C	-2.44714083282013	-0.60619104338817	-0.89054512524300
C	-1.80672814570064	-0.04595535878665	0.26717383853195
C	-2.60106164326653	0.53982156431774	1.24966750022147
N	-3.93222482916001	0.69512686226241	1.25440476199411
C	-4.52565882136402	0.20129660073897	0.18537739742905
N	-3.83057026154585	-0.41779598662346	-0.82771648289159
N	-0.48699349307506	-0.01610501842338	0.68049892893631
C	-0.48228048643421	0.56882021843511	1.85917071765569
N	-1.73393947989226	0.92326257493201	2.23449445786957
Pt	1.24550717886383	-0.60575367371585	-0.25071212115592
O	-0.30490115027758	2.72816392110482	-0.87298603391710
N	-5.85258029431792	0.29003855532460	0.05329778148355
O	-1.94104825684652	-1.18203763125739	-1.84304295486370
Cl	2.04562580195818	-1.49280419391546	1.79427327431236
N	0.57547437508555	0.20112772586471	-2.00946881110257
N	3.05088443532046	-1.11130115129491	-1.08597636287206
O	1.87350417342432	2.58701697677402	0.87959363152901
O	4.06641599551686	0.89667784870038	0.75351450044828
H	-1.98391607282020	1.38061329671325	3.10833964170457
H	0.40209049195205	0.73976528343271	2.46728906661667
H	3.35646172379511	-2.04125819883152	-0.79006462314228
H	3.10284419139254	-1.08773531212167	-2.10629293334458
H	3.72706687422951	-0.43043013298449	-0.70747773207206
H	-0.22451191410767	-0.34495985260072	-2.35214443598957
H	1.27149132763767	0.25401220819484	-2.75577466128843
H	0.23755443966046	1.15801013606152	-1.82680372213405
H	3.76115345700402	0.21850336843965	1.37601494307848
H	3.31019831804659	1.51916742136879	0.74390937522675
H	1.55362231315721	2.87439990884388	1.74420301075914

H	1.09143857711783	2.62590160168641	0.29486875163040
H	-1.20642879068179	2.70140820231062	-0.51892923132758
H	-6.34753578507217	-0.08012855039771	-0.74816694550527
H	-6.37980692744629	0.73520415396019	0.79444725291679
H	-4.34734894276820	-0.78928384573029	-1.62409717347676
H	-0.26925993256511	3.55365302860491	-1.37843548201760

Reactants for TS4G

Pt	0.56536053726707	-0.56322941601875	0.02087553217015
N	-0.52463727863727	-1.77575510614490	-1.20027115926739
N	0.41107815833092	-1.84841278099477	1.61037958909548
H	-0.38574588331795	-1.57375037859572	2.20066603718798
H	1.24987765639883	-1.78253625859810	2.19336618958284
H	0.29310273749797	-2.83442259466225	1.36534377565904
H	-1.16832869270274	-2.41066713994458	-0.71827210165315
H	0.09712900529412	-2.37001427658664	-1.75694766255530
H	-1.07734262545480	-1.23098610155643	-1.87141821432395
Cl	0.70227454693086	0.98022753794931	-1.76355613180215
O	0.42352759364716	2.46740723551386	2.40091269305451
H	0.88437415829605	3.30736235789042	2.26564169025023
H	-0.40573681052721	2.54692721894022	1.85961501898106
O	1.75407060212729	0.58926059485099	1.26861626458870
H	1.23745817967179	1.33398835982114	1.72888352328550
H	2.47309318011066	1.02585645184928	0.73147667185834
N	-1.68233395491316	2.41099334033225	0.66614696960149
C	-2.27204438939270	1.17995404300049	0.50667733034758
C	-1.84796877141918	3.01811219557666	-0.48018569050740
C	-2.79359495638394	1.05534381791071	-0.77767675900410
H	-1.50427146495610	4.02055244561543	-0.72820726238906
H	-2.74927876189681	2.49586766608214	-2.34041332931675
N	-2.52542001015648	2.24595881696881	-1.38132692985408
O	3.48890359600714	1.62997335623305	-0.40499930410295
H	3.73723951117830	2.54865574967213	-0.23045232282138
H	2.82650497660995	1.68357504647817	-1.11656552461108
C	-3.49811471029432	-1.04040145606805	-0.50687588061363
N	-3.00918206065474	-1.01619416430629	0.77893283960423
N	-3.40028805754377	-0.01621841881729	-1.32689289255958
C	-2.33446208435312	0.05279594882031	1.38935659407747
O	-1.86469278147301	-0.07313943458190	2.51219581759983
H	-3.09997280906386	-1.85213664020339	1.35499498408017
N	-4.03814238022928	-2.19604170873153	-0.92972186519637
H	-4.47632985577291	-2.20411257430476	-1.84302912905079
H	-4.24541610022459	-2.95402373339005	-0.29083936139550

TS4G

Pt	1.81324101334964	-0.42929696205542	-0.09176861933615
N	1.71901372917340	-1.45133644298870	-1.83781604503166
N	2.36263829406271	-2.08514007210810	0.96844550113454
H	1.49539412098860	-2.57127362531297	1.21953477541655
H	2.83030437198538	-1.82902592777213	1.84183783956320
H	2.97372129426734	-2.74888230401485	0.48726719264627
H	0.77227724199721	-1.79582846399750	-2.02313791736529
H	2.34592651879594	-2.25733247009228	-1.90030689497085
H	1.96491457451809	-0.82358837445597	-2.60952028673238
Cl	2.59232889109695	1.87588105382711	-1.47478506773196
O	0.95119364125011	2.92828398093064	0.98666253687951
H	1.39077028164859	2.81677805361989	0.11690479767344
H	0.00505011143459	2.90440618286812	0.78640874733662
O	1.97173058410901	0.64017351024759	1.69231122890081
H	1.46294123462969	1.49574296479926	1.57507192116658
H	2.92566008794434	0.94514525378715	1.67365616179583
N	-0.29470236968199	0.57993473846843	-0.72117505145611
C	-1.43014786705382	-0.05618184382117	-0.27327806165586
C	-0.71094054283787	1.53549592991404	-1.51196517763671
C	-2.55948005842107	0.55394676670509	-0.81539360550502
H	-0.06293635212374	2.22993346953620	-2.04403806792533
H	-2.62822715467852	2.21569348586669	-2.14429557046138
N	-2.07375513014314	1.56337589828300	-1.59628658669820
O	4.38812623021242	1.37586838280636	1.06755127806285
H	4.83013406686601	2.13046451776120	1.47985026689024
H	4.05414605994398	1.70706967621270	0.20863298033631
C	-4.01933587714021	-0.80228323757734	0.17408851169011
N	-2.96906854420620	-1.47524668925753	0.75449748075596
N	-3.85147699491897	0.23015694554727	-0.62625369655080
C	-1.60497535325103	-1.18658385861539	0.60012503209773
O	-0.76352780367276	-1.86562358675079	1.16934091527499
H	-3.17078494053754	-2.26863802968863	1.36082352601327
N	-5.25333203481969	-1.23747782522453	0.46363965425613
H	-6.03931909800328	-0.78246669510656	0.01676415436792
H	-5.41750222678416	-2.06214440234089	1.02660614679882

Products for TS4G

Pt	0.85100565899097	-1.10958551421576	0.13731364558524
N	0.44771443145943	-2.65498445052073	-1.11479410211137
N	2.40957948684752	-2.15990177889977	0.98762256605085
H	2.15851536196093	-2.54857867172100	1.90059395559413
H	3.20648440460177	-1.52438876398400	1.12980584455705
H	2.74605916469357	-2.94199653598733	0.42090918323996
H	-0.29765831455723	-2.42463859972650	-1.77783487085394

H	0.14238587037882	-3.49189581199972	-0.60943821630520
H	1.26209214868284	-2.92622220823429	-1.67384046405508
Cl	2.84044925554429	1.28091053825608	-1.74855179336083
O	0.90284567149651	2.67372198237685	0.18161865688406
H	1.49085320956932	2.37104681355345	-0.55153297455930
H	0.08973992176747	2.98679415828572	-0.23698622936190
O	1.24464672141637	0.45439255692971	1.46130672062853
H	0.96389888455351	1.32822348093977	1.05099012928774
H	2.23703896795172	0.52861097529242	1.50398299587460
N	-0.64861973010635	-0.00643756037186	-0.68848115831842
C	-1.88664986995094	0.23578046552354	-0.13218893542665
C	-0.56792185065921	0.74024753753621	-1.77035946408212
C	-2.56287803417255	1.16142312897944	-0.91586847936252
H	0.31268033166706	0.82087843292877	-2.40708797777982
H	-1.88552159631501	2.10397953443022	-2.70706722411093
N	-1.70500529556977	1.45690241010198	-1.94275548511848
O	3.82319542177144	0.38161534601939	1.01346819982706
H	4.50074129234680	0.94132724712480	1.41617388525787
H	3.68504956893759	0.72635171365029	0.09677306140575
C	-4.37875720378336	1.20624101432672	0.36792179946473
N	-3.78989593419694	0.28435377485322	1.20271138490277
N	-3.78884002276762	1.66972827230861	-0.71715786508764
C	-2.51851974259317	-0.28423861552919	1.04947434146888
O	-2.08817941436816	-1.09371676682809	1.85052497638507
H	-4.30325138371608	-0.03026890183934	2.02466945500600
N	-5.60323427839485	1.62818368114764	0.70131529881790
H	-6.05301827037605	2.31873530115121	0.11320173227095
H	-6.07657583311061	1.30493281414149	1.53581440738505

Reactants for TS5G

C	-2.56137699244137	-0.21680180176500	-0.53806327012649
N	-1.68372594792678	-0.99395926610023	-1.26636453211809
C	-2.42120007365579	-1.93800549950055	-1.79359662142224
Pt	1.90618063434359	-0.47447593169299	0.44777802173695
N	0.71344188575176	-2.10457937444202	0.63061909180827
N	3.26582162800775	-1.27574658709232	1.70724929779848
H	2.88478993685958	-1.45033246230235	2.64218174104209
H	4.06057650441245	-0.63899645830862	1.82149813864640
H	3.64469218388487	-2.16542965507738	1.36884499670530
H	0.34691465551029	-2.20646123940970	1.58208471450554
H	1.20570302292591	-2.97506792864412	0.40811379911957
H	-0.10121556646497	-2.05566827123660	0.00727704000205
H	-2.06508235748148	-2.74052125809389	-2.43686515073357
H	-4.49199505824507	-2.43274870848496	-1.74190199457775

O	0.56057801046787	0.42430181719456	-0.87370967470314
H	-0.07582151821342	-0.22965833314675	-1.27665560261442
H	-0.08493263311145	1.00383557994869	-0.34132409128278
O	3.15895399181640	1.17597435392469	0.28025690972780
H	3.20650479059940	1.45167151401503	-0.66370287022830
H	2.70217707435038	1.98540948913012	0.67347014216409
O	2.24911286051321	2.40951836980642	-1.89488432049734
H	1.52157790148517	1.77575762448254	-2.01958227484155
H	2.51578848823016	2.69282346749680	-2.77982235396299
O	1.77679122161574	3.29189494091181	0.70364705938814
H	1.78641694527946	3.40268411319265	-0.26576302146556
H	2.18628132988299	4.09443463434334	1.05628929329094
N	-3.73670960426299	-1.81785868973843	-1.44880542456382
C	-3.85653483366224	-0.72216269293108	-0.64431604649340
C	-4.78516415976720	0.84214864626753	0.63900781846713
N	-4.97698807052753	-0.24216993555695	-0.08513617013934
N	-3.54355624515450	1.42115394419046	0.79481155310086
C	-2.35954400114210	0.96521070764336	0.23671835051852
O	-1.30706643687192	1.58462989244205	0.43868871813098
H	-3.45765810507458	2.26765206843006	1.35692186875690
N	-5.81219229715559	1.43041274782314	1.25977951819622
H	-6.73464042041811	1.02789004895060	1.14792649793415
H	-5.70375874435979	2.26542013333006	1.82146884873040

TS5G

C	1.93209890020836	0.41558735904875	-0.13062349019503
N	0.68061686891852	0.85229634861825	-0.51446771478964
C	0.90403550883618	1.76080301660420	-1.42974244771103
Pt	-1.55084062677672	-0.42161288300586	-0.29081365621564
N	-0.38872144548077	-2.07237871259874	-0.51927794957858
N	-3.00677538999343	-1.23690485492613	-1.41653439481807
H	-2.70736109561422	-1.42075162754952	-2.37830553276779
H	-3.79757733611757	-0.58686702563007	-1.46491609235410
H	-3.36158120982174	-2.11724268505785	-1.03239868750018
H	0.29386474214186	-2.14215305846697	0.24737553142956
H	0.14780848294306	-2.03320076664114	-1.39162201360780
H	-0.91659232961872	-2.94904609115605	-0.53678091487937
H	0.13876118942303	2.33457025000331	-1.94830678508947
H	2.64735630293855	2.59999025083907	-2.32362646663154
O	-0.64184901256246	0.18259520226222	1.89009426864987
H	0.14196241522164	-0.40566468264487	1.98842494876692
H	-0.25835445993961	1.06868346452902	1.79190587423113
O	-2.79479505771501	1.21467716616816	-0.04504973751107
H	-3.13174082627985	1.13523371964826	0.89503418722587

H	-2.29502011308840	2.08169072676024	-0.11176001078595
O	-3.33971356176964	0.65658619047776	2.44593375723392
H	-2.42926019670667	0.34946840386902	2.61218748067277
H	-3.88458938378731	-0.13940823251560	2.52292702245152
O	-1.37382181723918	3.38355239812633	-0.21080306627386
H	-0.67500467808444	3.36894191123312	0.46021543380041
H	-1.84370983629646	4.21627553437995	-0.05624248551037
N	2.23807608899909	1.94500019550394	-1.66230715731727
C	2.92124662542451	1.09409917578125	-0.84470260123206
C	4.62605697580094	0.03593362923439	0.12183529009995
N	4.25093028223717	0.94459085304529	-0.75611610226672
N	3.72360523427281	-0.68543239620272	0.87286532679320
C	2.34191931346451	-0.56547992352738	0.82020538494885
O	1.62827009813556	-1.27380817939528	1.54274402576216
H	4.07393243732561	-1.37694190814175	1.53507274992855
N	5.92361149791795	-0.22314107854263	0.31650294688126
H	6.60397933620051	0.29364830171889	-0.22680211619337
H	6.24708607648225	-0.91499999184891	0.98088519435297

Products for TS5G

C	-1.57176687782774	-0.31731492293360	-0.24329846781174
N	-0.43738393351144	-1.03285429595928	-0.56464572777892
C	-0.81913241454393	-2.27983476954889	-0.73602689044462
Pt	1.44480124203074	-0.26542138145025	-0.46582568355843
N	1.42609591078390	0.12807223739736	-2.45882169627315
N	3.31013922128209	0.58598278441795	-0.29398998728387
H	3.91422142184641	0.47039733936275	-1.11132679829490
H	3.83391153184394	0.22672079174922	0.50880267313869
H	3.16918922692010	1.59392638867572	-0.14747996068346
H	2.27442870087090	-0.18468589861929	-2.93964690264436
H	1.33529776445664	1.13198771087951	-2.64299811629760
H	0.63976372533445	-0.32719535348175	-2.93060551272672
H	-0.16787162613386	-3.11287700314776	-0.99039622071736
H	-2.68439736084391	-3.27141831556159	-0.62773897834441
O	1.64012481202844	2.90906271434917	0.28853374490170
H	1.58962421494810	3.87326255592306	0.23970250166196
H	0.73471633498741	2.58872524458224	0.09785777579728
O	1.47126563423345	-0.67196093422980	1.57021931854071
H	1.61403606308362	0.18679830476786	2.08513740577467
H	0.62056113549970	-1.07665794841623	1.89264035603310
O	1.91622915184969	1.61570147448252	2.66099510846718
H	1.82577801263090	2.22391884203535	1.89134345376543
H	2.84550786169076	1.68532134992058	2.92172058058187
O	-0.83025037972914	-1.75601180122635	2.32388005096448

H	-1.46608790565503	-1.10321488988412	2.65145650395387
H	-0.79464324590665	-2.42825999247388	3.01940986338200
N	-2.15429728190171	-2.40666943335105	-0.54786792530224
C	-2.66049750012540	-1.18020081247834	-0.21772872206161
C	-4.12794305070257	0.38701588704661	0.36700728814587
N	-3.93452877881706	-0.88501797152377	0.07321666407603
N	-3.11042041683797	1.31474288691117	0.36089766161626
C	-1.76965990721768	1.06689168465438	0.06516817486100
O	-0.94939157253382	1.97464618854993	0.09161628870439
H	-3.32694337876593	2.28452859064944	0.58931451397157
N	-5.34840565104394	0.82631241165247	0.68737921107874
H	-6.11011945522493	0.15928561192505	0.70766105123698
H	-5.52828622899850	1.78913172435361	0.94298639956962

Reactants for TS6G

N	-0.07317777586125	-1.99413038101532	1.78044127641634
C	-0.08583554577704	-0.65993712701696	2.00983003098650
N	0.58478027299620	-0.01310530870642	1.07910575914385
C	1.05074057332247	-0.96941169356655	0.19943131002465
C	0.63110802029283	-2.22258518469856	0.63194303169284
Pt	0.74912793342040	2.02739118844536	1.02765635202021
Cl	1.07560652624263	2.06223373045662	3.37683644273063
N	0.87227656986915	4.08089067084719	1.05529466449439
N	0.34458954722083	2.11579568141958	-0.97525596936296
N	-2.50520224715620	0.47391044235547	0.07056686731965
C	-3.21177674720587	0.13575120368101	1.11683208960927
N	-3.37263074738686	-1.22056125163792	1.22075296204275
C	-2.72471078831058	-1.78916492545159	0.16438040693576
C	-2.18316065378626	-0.71563371655828	-0.54118475593944
H	-3.88346986245391	-1.71419821049301	1.94771635496706
H	-3.63681455447263	0.82627917184560	1.84389732227078
H	0.01918286148906	1.23510786024109	-1.39997271206994
H	1.15373540171531	2.41919393453347	-1.52160248393473
H	-0.41993823605998	2.79554312457624	-1.08604629992189
H	0.84921228944263	4.53348726342576	0.13829914115468
H	1.72076774058238	4.40256002003206	1.52718517464326
H	0.07092508256154	4.42974521054047	1.59760269079199
H	-0.57295045729611	-0.19446365403007	2.86340072814318
H	-0.53276181346047	-2.70097402234675	2.35016754943452
O	-2.17413012709079	3.31264617774297	-0.12593216474371
H	-2.30261763700801	2.34153651603117	-0.12822694201943
H	-2.00289716789087	3.50585499205960	0.81253424033599
O	-1.57508551120264	3.75980404589343	2.63111216083746
H	-2.06276375822934	4.27640692304340	3.28608667355770

H	-0.97281997461606	3.19277895945327	3.14370338093679
C	-1.92466919453266	-3.35886790525834	-1.19942865444065
N	-2.61578804157692	-3.10327459665473	-0.10961329163981
N	-1.35518675527111	-2.36884887807540	-1.96796934283516
C	1.52713313629766	-3.33769519494678	-1.06916010084493
N	0.85006982093547	-3.41587631385755	0.05751086967289
N	2.00404219748362	-2.14890423706629	-1.56920207030127
C	-1.39472153897072	-0.99554119980062	-1.70494550601000
O	-0.77614188693827	-0.21397334446614	-2.41887648134487
H	-0.83182867820568	-2.63302004415268	-2.80202490384501
N	-1.76741315551648	-4.62235484781446	-1.62663323872839
H	-2.07597519092748	-5.36235746025360	-1.00735870812631
H	-1.07944075091748	-4.85169502108661	-2.33423214598605
C	1.84853234237437	-0.87798400425762	-0.99582526680269
O	2.35471394983168	0.10328821109321	-1.50725976460727
H	2.54722967695410	-2.16198998742582	-2.43156194114401
N	1.75021481974704	-4.44102285876271	-1.79717903122882
H	1.47933179978703	-5.33254594176502	-1.39924405325976
H	2.37056823555645	-4.43491801655130	-2.59764165102560

TS6G

N	-3.46493027534401	-0.84640638972065	1.82816655189537
C	-2.42000687458786	-1.57664363691760	1.36641744307978
N	-1.84643209284766	-0.98353944506495	0.34244435299716
C	-2.54798908141278	0.18758450401387	0.12683422002396
C	-3.57018949968861	0.28395726757801	1.06577132975527
Pt	-0.16520927965397	-1.59670924594796	-0.66403977424272
Cl	0.46911901020819	-3.13794774556069	1.49761326627736
N	1.54824660728358	-2.08822287657854	-1.66978537654680
N	-1.19400701804141	-1.48201774842337	-2.43290682341021
N	1.26441194016658	-0.10733563377495	0.71428835277234
C	1.21995471292997	0.06609789804104	2.00905924570882
N	1.71998371671724	1.28218613501804	2.38072061181630
C	2.13730868123563	1.91705487205317	1.25004106890849
C	1.83764791748329	1.03615321794297	0.21209140687241
H	1.81658524715916	1.62837689832952	3.33079726093499
H	0.82421424673688	-0.65990713678599	2.71691675559826
H	-1.38522834002751	-0.49032800721045	-2.62206889783772
H	-2.09055406296051	-1.97295578472512	-2.39571320395385
H	-0.68402321005502	-1.85933362143321	-3.23455840668816
H	1.86624442536893	-1.23556300832013	-2.14930156430215
H	1.45988085782954	-2.83894171503569	-2.35709386513461
H	2.29161552145003	-2.34434148200616	-1.00310892694296
H	-2.08139743328236	-2.51281564717125	1.80452334466084

H	-4.06006819490833	-1.09550540471194	2.61465503328052
O	4.47202387366714	-0.04156916458917	1.61397350604708
H	4.48218166724550	0.54823999181068	0.85098225616625
H	4.05482012373469	-0.85192321655481	1.26325345035056
O	3.33385364323881	-2.38592032058368	0.63006986479314
H	3.84406156177538	-3.19827591291314	0.75530960242431
H	2.47441321590900	-2.56211047394047	1.07529664462248
C	3.03485558777057	3.47505310646756	-0.06158350706728
N	2.72874367266621	3.12300696208207	1.16918545367973
N	2.76940111986603	2.67965555412733	-1.15236213649946
C	-4.32621566355317	2.24603800729760	0.33696424209346
N	-4.46930069749128	1.26869685707863	1.21038946318651
N	-3.34413542306363	2.24145592834377	-0.62644839901755
C	2.17170523177916	1.41305443457397	-1.12921925980077
O	2.00880785531227	0.78963226788271	-2.17162079785234
H	3.03099050490352	3.01516899593421	-2.07777217675464
N	3.62398925696747	4.65514535235956	-0.30237078145294
H	3.86657605127915	5.24033408885431	0.48701314672961
H	3.93346679226977	4.92273886389928	-1.22767281435654
C	-2.38788710643678	1.24354742104510	-0.83301051729701
O	-1.57389457882426	1.34121935342613	-1.74099824666987
H	-3.28993130071390	3.02336162755827	-1.27809061884817
N	-5.15119856662092	3.29819964981114	0.36101962767275
H	-5.88625048507823	3.31739058132802	1.05710835906951
H	-5.08658385836155	4.05518378111280	-0.30828976674159

Products for TS6G

N	-3.14969114502784	-1.34471603581450	1.85790221534905
C	-1.96710517531998	-1.73710398820097	1.32258466854434
N	-1.61882390221002	-0.94321941784862	0.33094795872286
C	-2.61233637906424	0.00842804995310	0.22058754704909
C	-3.58488623652641	-0.23826393309392	1.18126821259493
Pt	0.05550170744863	-1.11076951261151	-0.84459803591547
Cl	0.90260700739582	-3.48282188454253	1.85907635289192
N	1.75313726619436	-1.26004274387544	-1.98763348739561
N	-1.02731305921999	-2.34363906219325	-2.09887950426254
N	1.09628898928321	0.12145434213384	0.40695143938752
C	1.30864540991373	-0.07854397315848	1.69071599754583
N	1.91862174114131	0.99330497709531	2.25015429525516
C	2.14011859107132	1.92137432582240	1.27354714466636
C	1.61535405900466	1.36661129215051	0.11246072095783
H	2.20761773154504	1.06850119454470	3.22197451106025
H	1.04746034707631	-0.99524973477952	2.22335445745505
H	-0.52131638723141	-2.67122449273530	-2.92474068138676

H	-1.86858888458788	-1.87247160503652	-2.44406103291008
H	-1.34856831012422	-3.18128040420363	-1.60515257676092
H	1.98954254307048	-0.33563180378235	-2.36457800849648
H	1.70734020579049	-1.91221320801958	-2.77268808726381
H	2.50562526811842	-1.58211635632967	-1.35841755288781
H	-1.36725048653463	-2.57835907904720	1.67665692783840
H	-3.62590413696888	-1.79422321450399	2.63602557318056
O	4.41358923722256	0.07461546311648	0.64270222512741
H	4.24240747484144	0.50490712833016	-0.20366942480863
H	4.00219767571800	-0.80455756162341	0.53546688867107
O	3.23520098860017	-2.38897542358937	0.20652605422826
H	3.78307932210212	-3.16776561200159	0.03892507666161
H	2.48492851069570	-2.72009080555437	0.76359783199047
C	2.86205716306658	3.78297851026768	0.29101905853063
N	2.75364220796849	3.10466668179123	1.41648828915290
N	2.40604957226825	3.30032609945364	-0.91419796598198
C	-4.87024273488178	1.48436702225053	0.60591704284763
N	-4.71253689640243	0.45250927696920	1.41195359375903
N	-3.95803206604152	1.81364028843222	-0.36996457976058
C	1.77305438036723	2.07132969615210	-1.12653218276706
O	1.46378668393170	1.71481888049692	-2.25471043437396
H	2.55172453918098	3.85730760171129	-1.75528960671132
N	3.43491721945920	4.99160472749456	0.28675628065781
H	3.79097844255927	5.35745669373968	1.16095740646358
H	3.54505225779031	5.53771589330483	-0.55808508492508
C	-2.76470470578618	1.13611105769008	-0.65716500625572
O	-2.02140820084065	1.52245367211810	-1.54086329647616
H	-4.13800776030188	2.62672359752891	-0.95697813741505
N	-5.95298760864624	2.26247483548603	0.71473659396496
H	-6.63204329950346	2.04399159852337	1.43317386042507
H	-6.09979016760616	3.07275694598871	0.12639246177545

Reactants for TS7G

N	-3.90660571338115	-1.90405363978650	-1.54945484879929
C	-2.77391041439642	-2.07487315135470	-0.82713571693016
N	-2.45511830473274	-0.97340177064450	-0.18088255271300
C	-3.42274364975382	-0.04074661770322	-0.49830501799424
C	-4.34486420936591	-0.62199351391800	-1.36335780161766
Pt	-0.80026379344612	-0.73412854587854	1.00038452641036
O	0.15051635677075	0.20571843090501	-0.58177414822664
N	0.89602896015768	-0.52417264164286	2.13783380664814
N	-1.75876225770755	-1.66838592652594	2.53319487868666
N	2.72603154159389	-0.54426515772504	-0.56940162510799
C	2.89320935173277	-1.74391918509288	-1.06773190971331

N	4.19067117431710	-1.98161353380053	-1.41420774021175
C	4.90698102336604	-0.85593125536875	-1.12693050841089
C	3.97595236880082	0.03535107373160	-0.59336260029160
H	4.55719840018747	-2.83882337438624	-1.81980048221016
H	2.11017716315580	-2.48731879529580	-1.21149909050068
H	-1.29057660714347	-2.53753121602010	2.80744107598627
H	-2.72405122096873	-1.91714116408027	2.29944833940190
H	-1.80413251988562	-1.08114830166717	3.37153043639192
H	1.67700804157152	-1.03582072242477	1.71542126306770
H	0.80930291120886	-0.85587430731751	3.10113710289061
H	1.17768603343785	0.47220749129954	2.17190664226489
H	-0.00569483067721	1.20744965204999	-0.49207714588531
H	1.14055133306146	0.03888429509304	-0.56745210605404
H	-2.21332810932907	-3.00679266237807	-0.79940249090705
H	-4.35160603049138	-2.61026853273609	-2.13170685695903
O	-0.33257434126810	2.65792520457946	-0.07822375228378
H	0.27145240786596	2.69206879389263	0.69606633306538
H	-1.23120525985869	2.55899199918671	0.28959835440490
O	1.55486315529637	2.24591520046145	1.80711536548581
H	1.74796912341564	2.80971443354045	2.56886210274939
H	2.38113390142954	2.23750777680557	1.26691010123477
C	6.63821421169317	0.53319936006414	-0.97124903513934
N	6.21990017018734	-0.66288966758241	-1.33151538968378
N	5.79929300774359	1.47815366052476	-0.42644200626873
C	-5.61466559897083	1.19515419890945	-1.54599828369038
N	-5.43181566707264	-0.06045371849515	-1.90766368368554
N	-4.76087325186301	1.85182237830937	-0.68858533032410
C	4.43417307641776	1.32730611011368	-0.18083963052791
O	3.80477406367014	2.25070424144039	0.32881080860390
H	6.18363706151227	2.38265534456638	-0.15659622916673
N	7.92426580436461	0.87173267454945	-1.12340312600799
H	8.55055804089819	0.19675931576285	-1.54440666215637
H	8.27522452746218	1.79290602531582	-0.89469840676191
C	-3.61373520679140	1.32199560167514	-0.09835536230366
O	-2.92540644457201	2.00592751857181	0.64994928788885
N	-6.66161170410985	1.88305460751498	-2.00926188485509
H	-7.29399637631864	1.42170485933053	-2.65191085121566
H	-6.81468438193759	2.85709151253647	-1.78086771864420
H	-4.96013731727687	2.82169564109431	-0.44439042993351

TS12G

N	2.50853186098500	-0.55874338916563	2.38077486259469
C	1.44605857580843	0.15164524438011	1.93434596818374
N	1.48405657284067	0.29606113651881	0.62642477494939

C	2.62957817600327	-0.35092981988883	0.20052249957217
C	3.27862702483395	-0.89880227019395	1.30353288301361
Pt	-0.00342756670806	1.19938658966042	-0.47660558077250
O	-1.61443908765447	1.07324770672827	1.30683172761096
N	-1.51403002422534	2.10629843103039	-1.49532901357419
N	1.36994463704616	2.27962918386008	-1.50022715962478
N	-1.18053281095127	-1.05590941284990	-0.38970327483647
C	-0.52051469146172	-2.13101526446817	-0.04335490432416
N	-1.35123250402693	-3.14225872224408	0.34911454973423
C	-2.63190717159553	-2.68197603105040	0.25244263279887
C	-2.50991697161076	-1.37468111219564	-0.21813642960364
H	-1.06946926045373	-4.06674827638579	0.66345077403337
H	0.56395844994798	-2.24027086710989	-0.04467393979304
H	1.94015024698682	2.85499506559996	-0.87461396981227
H	0.95874580571462	2.91270069743391	-2.19068646502733
H	1.99886390399176	1.63300464578263	-1.99694255887552
H	-2.40700727452394	1.64101431009635	-1.26135912765925
H	-1.41040480568248	2.07588997299887	-2.51205034236783
H	-1.55722974667186	3.09335843562542	-1.20952468208308
H	-1.66846644280841	0.29497660369697	1.87738176437610
H	-1.31310860189628	1.83700781093891	1.85820686830536
H	0.67091374039335	0.54191476517979	2.58939394261072
H	2.69750929668922	-0.79799899984832	3.35160895336661
O	-0.57388059058411	3.31452136923403	2.27368875322295
H	-0.68720147277060	3.83789885348380	1.45189083030797
H	0.38440293057429	3.22313077949462	2.36473223297450
O	-0.86873958900428	4.63822803586256	-0.14243356007704
H	-0.02822315232198	4.97963199992905	-0.48192067425683
H	-1.45581782719096	5.40813085926565	-0.13119902407862
C	-4.85369879771946	-2.65709436221465	0.36983444555144
N	-3.75421844839165	-3.35729404704675	0.55611904950037
N	-4.84037008847169	-1.36497729816905	-0.10182780595467
C	4.93675809308965	-1.80751323721905	0.13150331226585
N	4.40945177512494	-1.61620682718522	1.32545166343029
N	4.37100512541069	-1.30400106000503	-1.01757621275619
C	-3.70739026280025	-0.61673604426960	-0.44311655921046
O	-3.83741516088061	0.52081776755586	-0.87693769373848
H	-5.72731296446403	-0.88278480774198	-0.24040260423814
N	-6.05007992173444	-3.19587565554154	0.64029425770796
H	-6.08172506397019	-4.14577760729017	0.98860307736859
H	-6.91526555568682	-2.68668381381516	0.51528384604320
C	3.20095687896990	-0.54736430732025	-1.10274937506864
O	2.79727356817026	-0.14245646348918	-2.18442632927535
N	6.06441491932580	-2.51110309172735	-0.00037325907541
H	6.49391229043046	-2.89270662976668	0.83359870300788

H	6.49095106459722	-2.68969332575583	-0.90096254410630
H	4.83283091932752	-1.47688752039841	-1.90986928234061

Products for TS7G

N	3.17225552082439	-1.87406842652606	-1.11769634507907
C	2.15529559577471	-1.24544580265153	-1.75450836676203
N	1.54207850318922	-0.40262574944738	-0.94916672143276
C	2.19613724879378	-0.47951173364187	0.26373817431664
C	3.22257343744139	-1.41487996273823	0.16957824072584
Pt	-0.14675737671255	0.68314322595752	-1.37661094683894
O	-0.61872954736855	3.13148917454065	1.01441862291378
N	-1.78213472396676	1.81389087298528	-1.88609787185945
N	0.99272878992849	1.82419339439275	-2.62909397039798
N	-1.23360981517484	-0.61415097715792	-0.14751906486423
C	-0.92216838746082	-1.89418561470235	-0.09773425992216
N	-1.71610112560494	-2.57197420646545	0.75981597407326
C	-2.60277110021372	-1.68989218003807	1.30529187286993
C	-2.29988020095735	-0.45532939992815	0.72873118898945
H	-1.65948316182902	-3.56802617513005	0.96151232778483
H	-0.13310530581144	-2.38126732425923	-0.66535881558935
H	0.59506794098311	2.75846992883198	-2.77166917220169
H	1.14174186918712	1.38789483906786	-3.54236376968389
H	1.90425403310894	1.98569390459964	-2.18332077414695
H	-2.21410533626778	1.50740121275627	-2.76111974175631
H	-1.49312067345950	2.79406613740056	-2.00029407270031
H	-2.50200311000653	1.81409681614565	-1.15301752698393
H	-0.03733903983905	2.38358745501106	1.24242651357178
H	-1.50380092829603	2.72946193527497	0.93500549969407
H	1.88718280326330	-1.43931416806591	-2.79140935115734
H	3.79093686043373	-2.56919329715021	-1.53055035179685
O	2.40520833571657	2.95674073139982	-0.40497391535122
H	1.70460973430332	3.56485952533301	-0.70157936867244
H	1.99848475004995	2.50745743278870	0.35102721881520
O	-0.02164872023619	4.16272067382900	-1.36204337069023
H	-0.18244191567696	5.11498021320111	-1.39052998604959
H	-0.28642369409316	3.87151714007426	-0.45079196554094
C	-4.27788878335894	-0.94162865712636	2.56347515332471
N	-3.54958441452718	-1.97994112812172	2.20338760222327
N	-4.07125411484166	0.31357993875783	2.03985223306796
C	3.90303170158168	-1.16333580615129	2.27241093201476
N	4.08224395127850	-1.78844845550463	1.12362245232890
N	2.93619959114866	-0.19652070976096	2.44771220466026
C	-3.10745751308900	0.66539475383117	1.10376887671991
O	-3.04635450658467	1.82750746600568	0.69893735131095

H	-4.67170481969290	1.08069945410704	2.34291377239397
N	-5.25559393840401	-1.08038941349073	3.46018323855446
H	-5.41860225976854	-1.99856556322297	3.85593150296952
H	-5.82517768283663	-0.30240556375912	3.76816143276223
C	2.03956277266318	0.25005597779283	1.48328531117936
O	1.27027759468629	1.17665191468212	1.72613565624606
N	4.67349827472986	-1.45171572557600	3.32265203173098
H	5.39776626871862	-2.15123769676036	3.21182884792601
H	4.56969284841977	-0.98641672126188	4.21569060697067
H	2.87465976985420	0.27992733987157	3.34759288933874

Mechanism path without explicit solvation computed with the approximation PBE0-D3BJ along with Basis Set 1.

Reactants for TS1

Pt	0.23454256412123	0.50389626633168	-0.85414122903359
Cl	1.34831067381194	2.22736715961823	-1.93498361781865
N	-0.70171440423759	-1.03052348697826	0.12594002390062
N	2.02981824337216	-0.45211574099294	-0.74650352076176
H	2.33000380143965	-0.63008463403476	0.21512708722349
H	2.74876119206316	0.13163749903839	-1.18333811059921
H	2.03295005189385	-1.35157406082871	-1.23387509661467
H	-1.21107316113918	-0.63732533617508	0.93876173195676
H	-0.06892574773205	-1.75482379733171	0.47007892125098
H	-1.38934572874656	-1.49523808226227	-0.47054339178708
Cl	-1.82886138878008	1.59176650608759	-0.97645836575906
O	-2.04253532720909	0.64339848913325	2.01988939712420
H	-1.30974993128827	1.11315250672859	2.44040572625972
H	-2.17218083756919	1.14048671166599	1.18964044465824

TS1

Pt	0.15887398344521	0.38786738470724	-0.15993451199012
Cl	1.08274510677959	2.49525615235016	-0.45795323632131
N	-0.68370483943900	-1.44508519055853	0.13339838719611
N	1.93929796424565	-0.41372349202453	-0.65760325917478
H	2.33252553586923	-0.99369227561637	0.08746481575864
H	2.60990227726790	0.33647851808384	-0.84697800531517
H	1.88521079490129	-0.99237704654199	-1.49918191254649
H	-0.78538486524672	-1.66688942260193	1.12650897803168
H	-0.20069437296513	-2.23338643674236	-0.30180929053998
H	-1.62453855005420	-1.38781615654659	-0.27260586193720
Cl	-2.39620451982362	0.85050081743423	-1.11566557463334
O	-1.33162485787224	1.36795160192086	1.53801050085056

H	-0.97402633953569	2.26576264612328	1.59685766837282
H	-2.01237731757227	1.42915290001270	0.82949130224858

Products for TS1

Pt	0.38094903892666	0.52803505812680	-0.10535431761896
Cl	1.62088875498702	2.43184391592035	0.30350107143887
N	-0.78257923836995	-1.12231622938193	-0.43702060155707
N	2.07254369066913	-0.55338430776296	-0.23314153279998
H	2.10636866779533	-1.31667185818640	0.44782915306850
H	2.88532379740042	0.04244080198083	-0.05228339340780
H	2.20757350850722	-0.97083386840510	-1.15781564960164
H	-0.32195467462754	-2.01173921242961	-0.23475707307861
H	-1.11213676893084	-1.17234561851024	-1.40373648347487
H	-1.61589242052871	-1.05059831108167	0.18039916236157
Cl	-2.95737212911137	-0.09540331170644	1.63915777484820
O	-1.34205470388263	1.64756891228290	0.00280022552033
H	-1.14172317488087	2.49134518295540	0.43904262038346
H	-1.99993334795384	1.15205884619808	0.61138004391799

Reactants for TS2

Pt	0.52592693126206	-0.14755909035347	-0.20019639143014
N	0.72624393826368	1.79238866797902	-0.66868177974824
N	2.33763498228240	-0.54906916725802	-1.05512718414651
H	2.31778246719707	-0.44799093617102	-2.07365869592891
H	2.64692522252310	-1.50559761180222	-0.86412474389515
H	3.07876022332905	0.06712100069435	-0.71058819350871
H	-0.19592937814073	2.20767375307348	-0.92314643387551
H	1.36175635318682	1.95448569679928	-1.45340978212613
H	1.09602955377382	2.32572382561335	0.12274303839701
Cl	-1.52708768849659	0.21600464127826	0.81725002693182
O	-1.91981927039788	2.65705283831317	-1.15410333714199
H	-2.16684718639089	1.95431817970224	-0.52645880166052
H	-2.05514102576315	3.46931197197935	-0.64741014513168
O	0.32708594786669	-2.15248054511709	0.31321136196923
H	0.20675419377734	-2.71853859780244	-0.46774273208034
H	-0.48700526427279	-2.26633462692824	0.83461379337581

TS2

Pt	0.06972094705658	-0.13797702753200	0.11050976796917
N	0.78898397000425	1.72907086175204	0.01407829735569
N	1.78954535187555	-0.90150748734377	-0.61825502722990
H	1.75507240946940	-1.92436206471578	-0.61203235221387

H	2.60477765127040	-0.63001478405298	-0.06209301155687
H	1.97228783147698	-0.61444909817170	-1.58357220175364
H	0.01178864175754	2.36014661762995	-0.20369567341351
H	1.50785387407894	1.87691002098527	-0.69863935440090
H	1.18760435940069	2.03027236320468	0.90782078314659
Cl	-1.50041621934916	-0.12726114720039	2.33552552621698
O	-1.99942697452090	1.09263575338795	-0.25457372416431
H	-2.56736597539476	0.66564721256660	-0.91154740744273
H	-2.36363786141379	0.82874163702809	0.61598450660027
O	-0.69525261759730	-2.05618180679790	0.28540754159961
H	-1.23880412667308	-1.92060400150167	1.09972657538297
H	-1.32273126144135	-2.27106704923841	-0.42464424609553

Products for TS2

Pt	-0.89739983465865	-0.05442000765970	-0.02208712013050
N	-2.33253192369142	1.35359045571830	-0.02870038440412
N	-2.24028542537392	-1.54516905677268	0.10009750930836
H	-2.71373834598657	-1.57266043567610	1.00782728420501
H	-1.77847215809711	-2.45125073429460	-0.02168458810576
H	-2.96974320341477	-1.49009142181360	-0.61596462250034
H	-1.92704281902953	2.27356265139494	-0.22442874398299
H	-2.81230928542050	1.42768904140731	0.87318518727198
H	-3.05240716002197	1.19051511247261	-0.73813332781248
Cl	2.41448650678517	-0.01663274387999	-1.76654981155452
O	0.48038905462148	1.46573002321193	-0.18944470278703
H	0.86390070576350	1.69119933816823	0.67359387342371
H	1.25178451705907	1.11273549892327	-0.75170045918432
O	0.57400481201204	-1.49378306096921	-0.07249985447271
H	1.32055826765948	-1.13820108879728	-0.66549784654207
H	0.97187429179376	-1.62857557143344	0.80282060726781

Reactants for TS3A

C	4.060941000	-1.168136000	0.006719000
N	3.683091000	-2.333133000	0.632003000
C	2.392604000	-2.192444000	1.021583000
N	1.902471000	-1.016001000	0.690782000
C	2.927943000	-0.347376000	0.049327000
Pt	-1.807109000	-0.191796000	-0.025194000
Cl	-1.286623000	2.043070000	-0.370949000
N	-2.234454000	-2.163552000	0.347274000
N	-3.187565000	-0.116581000	-1.499878000
H	4.264513000	-3.153112000	0.781766000
H	1.846765000	-2.978392000	1.550532000

H	-2.938986000	-0.711942000	-2.297852000
H	-3.272341000	0.838022000	-1.861591000
H	-4.123680000	-0.404776000	-1.191234000
H	-1.884665000	-2.787992000	-0.390750000
H	-3.236716000	-2.348269000	0.446493000
H	-1.794058000	-2.463786000	1.225044000
O	-0.425561000	-0.289351000	1.486170000
H	-0.298858000	0.591603000	1.874610000
H	0.519574000	-0.573003000	1.121644000
C	3.057209000	0.937502000	-0.548265000
C	5.249971000	0.368280000	-1.049074000
H	6.192988000	0.700298000	-1.508418000
H	1.122878000	1.654971000	-0.289002000
H	2.257078000	2.715558000	-1.085918000
N	2.076228000	1.841696000	-0.602594000
N	5.244942000	-0.859808000	-0.530988000
N	4.253631000	1.253331000	-1.084996000

TS3A

C	2.63534377897657	-0.64951996245821	0.13172509854920
N	2.25176170450478	-1.54057247481211	1.09721464380995
C	0.98603661464760	-1.21855756504455	1.46983485399979
N	0.51887376863311	-0.17516186596843	0.81880003115906
C	1.53797169633617	0.20631938866512	-0.02897578469651
Pt	-1.89831691966104	0.23117834845359	0.31216986144602
Cl	-1.80694298171520	2.49411876808048	0.82082162127211
N	-1.99871209743613	-1.76107342228628	-0.11621351300118
N	-3.07406868671372	0.68255078244790	-1.25231041841715
H	2.80768676920634	-2.30751103992680	1.46333867123764
H	0.43654977213938	-1.77941406721218	2.22450194851261
H	-2.64075532346992	0.43403517561625	-2.14528706093589
H	-3.24150479048497	1.69275554125130	-1.26926807878198
H	-3.98894664429692	0.22617526050671	-1.21643424620379
H	-1.20134067216335	-2.06064186868419	-0.68327040908428
H	-2.84155222629948	-2.04576474890622	-0.62020104084807
H	-1.97270609455990	-2.31233381963206	0.74582358289259
O	-1.58380295827530	-0.37115613906137	2.61387487462795
H	-2.44584155009479	-0.22592697370929	3.02831571826937
H	-1.01460380060903	0.31237295266298	2.99640256818671
C	1.69184555358944	1.24309260928837	-0.98526977211606
C	3.80638242734202	0.39721905145500	-1.41415297444811
H	4.72179437894369	0.50177348248305	-2.00997337674193
H	-0.05733408951951	2.27239115473956	-0.63805645119812
H	0.98036330359840	2.90094061581088	-1.89591882875535

N	0.75014476730198	2.15666633512994	-1.24898508960083
N	3.79197304731684	-0.59510226737111	-0.53114756214205
N	2.84970125276291	1.29114674848169	-1.66735886699170

Products for TS3A

C	-2.50264119172073	1.71008667078958	-0.12255695136291
N	-1.40963554438714	2.44984170420617	-0.49673679590993
C	-0.32517543486258	1.64853332422907	-0.47181696590098
N	-0.65261626433617	0.42038740963399	-0.10525749778214
C	-2.01427359606763	0.42696639371894	0.12837387022624
Pt	0.61046432017937	-1.13564232566683	0.09315001848108
Cl	1.36150853131978	-0.30284675334986	2.11890145955832
N	0.00421070086625	-1.86891937933809	-1.72121381000496
N	1.89799620950324	-2.70206460072708	0.31280490975498
H	-1.40739714508907	3.43528919427192	-0.74493683868275
H	0.69947574136598	1.96873809194447	-0.67921484637610
H	1.43079240168546	-3.61208967257311	0.30218512912560
H	2.39289893021637	-2.62788990566641	1.20558776141294
H	2.61108783547552	-2.72367835268425	-0.42079768620625
H	-0.71921258187040	-1.28953801123705	-2.15348133126028
H	-0.37803432948949	-2.81564431882858	-1.65297056243587
H	0.78046463979498	-1.90938627846485	-2.38654046370933
O	2.75935911174389	1.57893765545843	-0.19713487387599
H	2.50144479455850	1.11115676095254	0.61529992284545
H	3.04471879947603	0.85780829553632	-0.77315365595385
C	-2.95023627506875	-0.53933715107954	0.57362134720003
C	-4.56038118445425	1.11506002852394	0.39926125646502
H	-5.62259411030572	1.36212396407291	0.51533290626123
H	-1.64617903387883	-2.09136246147649	0.85809556667898
H	-3.32676746502387	-2.42719213186053	1.23086921099659
N	-2.61899411556484	-1.79792769861269	0.87282467106553
N	-3.76968764063799	2.10363140050495	-0.00590884589298
N	-4.22757110342785	-0.14368785227782	0.68962709528236

Reactants for TS4A

Pt	2.96731605710298	0.21365891832149	-0.06715254337174
N	1.60395118922209	-0.50065231605627	-1.34630660172643
N	3.24510826369343	-1.64915265143672	0.73118360040424
H	2.36536334825766	-2.07828283383961	1.03047176643204
H	3.85114440052711	-1.62982167526856	1.55519049292640
H	3.67737250658846	-2.29765751994291	0.06764456886392
H	0.65364169605753	-0.16833049089572	-1.04410239970467
H	1.58246298469173	-1.52035339111770	-1.41382724908158

H	1.76493060212701	-0.14428606920959	-2.29116531320677
Cl	2.67979598479347	2.34375904457371	-0.91556949249762
O	4.33537399199580	1.00198085780932	1.29434608315963
H	4.26713674961929	1.97242203081199	1.26123897617129
H	5.25213252372345	0.80848459862104	1.03602574693384
N	-0.85011258912601	0.64224313102115	-0.46352467800912
C	-2.10871184621183	0.19743049298630	-0.13484735649482
C	-0.89918474488758	1.94584801759120	-0.31166702982489
C	-2.92601607928877	1.27597569382591	0.22074654962642
H	-0.06073050993421	2.62276529612336	-0.48308549683719
H	-2.38972762613778	3.34141047023879	0.27764362500406
N	-2.12332450939663	2.37848431107540	0.09700178626509
C	-2.70390744856468	-1.08623216150627	-0.08473299317367
C	-4.65230031305492	-0.04325198065327	0.59960587467787
H	-5.69975088304719	-0.18099592661382	0.89588835754749
H	-2.51891485857798	-3.09938956020436	-0.33412344318726
H	-1.07258846740033	-2.18426457699315	-0.67112688689917
N	-2.04320041590752	-2.20826431287761	-0.39166183893258
N	-3.99146668045324	-1.16169014287450	0.29068128405938
N	-4.20577332641131	1.20818274649041	0.59520461087586

TS4A

Pt	1.94460510779380	-0.10328564544729	-0.20049117665082
N	1.39307978498420	-0.66543189642303	-2.04494415322756
N	2.75262962945102	-1.95458380074522	0.16872214476623
H	2.20593920109886	-2.73562932718034	-0.20221160598615
H	2.84759091592413	-2.11789978379101	1.17403164686897
H	3.69006678364272	-2.03395081162351	-0.23185085422353
H	0.43529151690081	-1.02656633758678	-2.05440024686776
H	1.98679573054359	-1.38535612066267	-2.46402854344659
H	1.41448298138389	0.13827947289298	-2.67804336878938
Cl	2.68660714951064	2.30640676573257	-0.77068045236796
O	2.56539212569202	0.56271889296442	1.66317215293806
H	1.82744041531290	0.61740572979850	2.29270857599890
H	2.79563955082753	1.49105054259504	1.44150984124956
N	-0.22521994056413	1.04565469270003	-0.06389698609950
C	-1.44253852739964	0.46090582726924	0.20248057675756
C	-0.49226166656275	2.30854967530535	-0.30913472983404
C	-2.46031291195508	1.41587278209763	0.10810877560249
H	0.26671184743455	3.04894359748496	-0.56158896476836
H	-2.25369953137494	3.48778791598604	-0.36675497038425
N	-1.81882825047395	2.58270801008252	-0.21503956971560
C	-1.84499293833684	-0.85463458746012	0.53917402121545
C	-4.01910766529086	-0.06708955074266	0.59352133916894

H	-5.07351455954431	-0.32285443851907	0.75634761788854
H	-1.30441096715716	-2.77923685719495	0.94641139335858
H	0.01929166888872	-1.68185726369931	0.58702471348529
N	-0.97613082451144	-1.86254623438888	0.67152710306754
N	-3.15610531809024	-1.07761044355996	0.72372257899591
N	-3.76444130812803	1.20224919411547	0.29460314099950

Products for TS4A

Pt	0.40193353765272	-0.78289655616132	-0.24116545151044
N	-0.46095232438054	-2.46603956772320	-0.93662366937289
N	2.09885152063688	-1.80352746746901	0.25423998374864
H	2.23449654689847	-2.68211402413007	-0.25086883691604
H	2.12879632095890	-2.02961772288583	1.25153121899922
H	2.91434110946028	-1.19315491111415	0.04206008364838
H	-1.41794887889448	-2.31072072023598	-1.26358342555893
H	-0.50701790163195	-3.19930422087902	-0.22339702411512
H	0.05841500601342	-2.85528640430755	-1.72871340504371
Cl	3.92567405861396	0.62352245747534	-0.62594872887155
O	1.27345476122334	0.95858616745960	0.41648583539222
H	2.25325715269961	0.96071715350869	0.09953705942935
H	1.30119671146394	0.99944516854940	1.38528706055558
N	-1.24466164971456	0.27986143115215	-0.71557876968942
C	-2.36334004169015	0.48746161879853	0.06993010517227
C	-1.44607448542653	0.94701959353328	-1.83955723566136
C	-3.25071624253830	1.30772587563124	-0.62850233545134
H	-0.75062651891597	0.99229870724287	-2.67573356703529
H	-3.00987169245838	2.15046684618260	-2.57692480026220
N	-2.63722054332501	1.57559590088864	-1.82569660461978
C	-2.76431682429019	0.06680730714610	1.36326589519680
C	-4.71273486923938	1.27099828074828	1.01953672933021
H	-5.67774962662169	1.57381006549194	1.44351997157661
H	-2.36472470824468	-0.96788387782833	3.06690807031433
H	-1.10303003331962	-1.03106001534687	1.85173356958251
N	-2.02028768514492	-0.71384663743285	2.14929073989897
N	-3.96233981950761	0.48935967465181	1.79675994973378
N	-4.43830288027745	1.72769587705372	-0.19827241847079

Reactants for TS5A

C	1.92913706277244	-0.18893960123399	0.36483887318472
N	1.04947498351945	-0.36198901023402	1.40887980024707
C	1.33402643333539	-1.54429347855582	1.91071717515433
Pt	-2.21826161745312	0.39927192170802	-0.07180681895130
N	-3.26408321614804	-0.85624203066189	1.09252154401983

N	-3.23852264517506	-0.14078001982400	-1.72048337057931
H	-3.13164225592108	-1.13662570812717	-1.93342703373805
H	-2.90288798886161	0.37396250076610	-2.53977526307308
H	-4.24307782191445	0.04206459593255	-1.64476996205179
H	-3.11600955848090	-1.83935920866559	0.84709337807867
H	-4.27298245685260	-0.68647275432679	1.05303247280238
H	-2.97888635485058	-0.74478604504284	2.06994515829448
H	0.81957684719057	-2.01026346218108	2.75118299758045
H	2.73869112993113	-3.06925267160471	1.47243234850886
O	-1.19255168300547	0.88800667897808	1.63603618721661
H	-1.06711633526978	1.84127546945300	1.75839473108986
H	-0.25108748864270	0.44141436765665	1.62351361300267
O	-1.16373005398020	1.67596697856499	-1.29901129549502
H	-1.52665350885753	2.57556549994509	-1.26054029381299
H	-0.18162523584020	1.76982195899135	-1.07570085276991
N	2.35306307785245	-2.15357308372270	1.25759835947763
C	2.75537180902560	-1.31744820637157	0.25104414633830
C	2.15856353215166	0.83705984330686	-0.56703439160520
C	3.79835455679112	-0.48176426147693	-1.50662060889699
H	4.54992490606183	-0.57010773935327	-2.29910593857752
H	1.21757267291492	2.38188162282680	0.34553673607583
H	1.83429289256367	2.71782925575643	-1.18204389052166
N	3.70077458764506	-1.50274875408140	-0.66385840487774
N	3.09001801997828	0.65436457186172	-1.49765172149090
N	1.42028570951975	2.00613076971613	-0.58088767463024

TS5A

C	1.65255311370597	-0.25114510513890	0.04525016834715
N	0.45359541496041	-0.58736440561113	0.64145751144195
C	0.60505812750010	-1.84197880644229	1.01503107053415
Pt	-1.80422020548701	0.18749805811151	-0.04937984608196
N	-2.70246628233732	-0.91959267751690	1.36647975673500
N	-3.27657191300634	-0.22783341667998	-1.35879550197139
H	-3.40439061527271	-1.23524872157583	-1.48784222184482
H	-3.06286967650652	0.16086263553067	-2.28160850545617
H	-4.18038584630012	0.16008579057867	-1.07479634828850
H	-2.64431943582599	-1.92795379802463	1.19887398738931
H	-3.69696209269226	-0.69469562857026	1.45660018155740
H	-2.28033771744027	-0.73509522970298	2.28119269975123
H	-0.14697644255366	-2.44589919613994	1.52212426579429
H	2.14623151471452	-3.29043455744922	0.88107282025533
O	-1.05852478436780	1.41873646383089	1.88789837563962
H	-1.07784121290301	2.38489523307227	1.84069266819711
H	-0.14616535907007	1.18071048450584	2.10819294696390

O	-0.97639860224905	1.34554366755221	-1.54828691785646
H	-1.50714050442521	2.14627483817611	-1.68856672788489
H	-0.04520076055703	1.68059239523161	-1.33574581001015
N	1.82203875868496	-2.34492814604041	0.69611888600829
C	2.51943826043273	-1.35657656094429	0.05852922904605
C	2.16309117076573	0.91252748126044	-0.55483815600686
C	4.08411691085090	-0.25995961129364	-1.04884097272611
H	5.06966958293620	-0.23005828680523	-1.52674844176165
H	1.00453638797360	2.37623542533104	0.23765747256207
H	1.95760140344521	2.84485921293719	-1.05933071932997
N	3.73679974549296	-1.40278085243087	-0.47065184862698
N	3.37035677671304	0.87109967976969	-1.10820989577889
N	1.42568428281803	2.08162363447834	-0.64353012659805

Products for TS5A

C	1.61505813659847	-0.18598416797469	-0.27444381556373
N	0.40737324354285	-0.18545313820160	0.40155273112324
C	0.68090868549846	-0.53281713267225	1.65153101238000
Pt	-1.37928939198265	0.44367825108274	-0.31109074493142
N	-2.28062721541318	-1.18771604508506	0.43619679650749
N	-3.14420210545843	1.13810481540012	-1.04733166923015
H	-3.54735858368976	0.52124811475981	-1.75813335009529
H	-3.01114368206165	2.05113046899744	-1.49080767287698
H	-3.85631802167083	1.26111977830455	-0.32219145597981
H	-1.77800949929503	-2.04072265087599	0.17635695013988
H	-3.24287614411763	-1.30955408578357	0.11284193057953
H	-2.30150240189002	-1.14435648545637	1.47620330935603
H	-0.06629553493079	-0.62812674547235	2.44661695484933
H	2.44038845037094	-1.02559038777625	2.69599578308806
O	-1.95893842597609	-0.87710292406025	3.21473481242100
H	-2.10744355415524	-1.63228072147905	3.80015256376382
H	-2.42946872274807	-0.14504575376702	3.63673235219851
O	-0.50374207893769	2.13286564292619	-1.13470257976521
H	-0.06432264717320	2.70323421554424	-0.48322832431962
H	0.19138525050620	1.84417237086183	-1.78635129626832
N	1.99684305449326	-0.75687743642846	1.82090964372781
C	2.62916179053629	-0.53333232975474	0.62580802347389
C	2.02228375064620	0.10663590621145	-1.59041016192905
C	4.19470360556838	-0.26493419244138	-0.90687011489298
H	5.24981602072487	-0.27484820559947	-1.20198723637493
H	1.57363972862777	0.60596845091027	-3.48315705426827
H	0.28189357747347	-0.05807157149186	-2.62226182257790
N	3.92602973721858	-0.59300729460440	0.35089714666950
N	3.32332921975920	0.07293842645914	-1.86404632444909

N	1.13872475793534	0.49472582746694	-2.56951438675534

Reactants for TS6A

N	-1.77478334603037	-3.04023803835819	-0.90085279081949
C	-0.91250191371895	-2.06070497404860	-0.55850991529645
N	-1.56220848363936	-0.98526797006735	-0.14513682933421
C	-2.91000444813638	-1.27684955387992	-0.22907447887451
C	-3.05254403572495	-2.58158960573225	-0.70434436035675
Pt	-0.71712002456044	0.74779352073960	0.42862476347208
Cl	-0.37817182481375	1.33147031458939	-1.78261466406677
N	0.14494993199177	2.51493913963056	0.98183854033690
N	-0.97520791142881	0.22020159897215	2.39141364255086
N	2.23611696288466	-2.11313627261024	-0.29878959866642
C	2.56823339111456	-3.32131988962716	0.08988428377722
N	3.85934527262569	-3.40719508174494	0.51696714275733
C	4.40617939578631	-2.15908922435467	0.40023215928177
C	3.37413121042064	-1.36220969255539	-0.11321770792662
H	4.32719606394327	-4.24137025876460	0.85737924844660
H	1.90775168510521	-4.18943144036277	0.08756805369426
H	-0.15281627850506	0.42287620326647	2.96484458441115
H	-1.16208734399641	-0.78031680902588	2.49321597588358
H	-1.76392432124064	0.71163980585222	2.82031467841079
H	1.04243074367321	2.37540217336084	1.45334671390493
H	-0.43965387661693	3.07254774663148	1.60908281458765
H	0.33236600254140	3.08828509044888	0.15501863987909
H	0.18125686615211	-2.13611829935972	-0.61211798585637
H	-1.51408021535678	-3.95985940095697	-1.24561406003567
C	5.81433992131868	-0.46428831941719	0.46494138322662
H	6.80781115606312	-0.05763920224838	0.69244101144335
C	3.68933153242879	0.00247726618397	-0.32795669028145
N	5.64317353768975	-1.75957538676304	0.70511206543081
N	4.93223461582786	0.41189357918573	-0.01974794357637
N	2.81815267980014	0.89612148406472	-0.81142246099517
H	3.12860556611090	1.84871251860493	-0.95565222453379
H	1.88084973944472	0.64490799010138	-1.11003830059370
C	-4.10182444445627	-0.55780223904249	0.03535181461298
C	-5.25180987631329	-2.45790624102684	-0.61923931343762
H	-6.23771679545117	-2.91652004853524	-0.76270393575556
N	-4.11628509627501	0.70288391784869	0.47649786744330
N	-4.20289892672585	-3.21893161537046	-0.91468156965741
N	-5.26146335062494	-1.20127012106371	-0.17218116568611
H	-3.24977229836034	1.22513366588345	0.56835162242908
H	-5.00273146294708	1.17509366955161	0.60393898977007

TS6A

N	-0.95531699766764	-2.39702157062072	-0.96138905129106
C	-0.37990612987345	-1.68568052270750	0.02661376183639
N	-0.95569006294364	-0.50344596328268	0.16732265933466
C	-1.96453003736982	-0.44323937206310	-0.77950675442668
C	-1.97548169178735	-1.64859860786021	-1.48835649532023
Pt	-0.42287608696910	0.85144826816188	1.56509008111490
Cl	-0.10984923606748	2.92793834538785	-0.04838774848993
N	0.09942550099387	2.21333674417388	2.99064469016075
N	-1.78552645631764	0.00726920938402	2.83549523347250
N	1.88327573081151	0.46304257165537	0.86843528802646
C	2.72846134992021	0.05938776179121	1.79168035326357
N	3.76594693414964	-0.64722903178530	1.27618442103075
C	3.57923478887955	-0.72193374261872	-0.07758697664955
C	2.39682850589028	-0.01086569668292	-0.32415992771554
H	4.53328830524948	-1.05421335278361	1.80241680496115
H	2.63234701677407	0.24521052886145	2.86083821540865
H	-1.40299500486776	-0.14839350615567	3.77094158481239
H	-2.10598604904476	-0.90246246611649	2.49598384226114
H	-2.61761998083638	0.59162366120648	2.94316170527981
H	0.87002607792659	1.90896156278380	3.59023092534526
H	-0.66924788255456	2.47320428343771	3.61277284578692
H	0.39656840884140	3.07024862560410	2.51496979007154
H	0.44734528857867	-2.05503609128396	0.62893695903723
H	-0.69000511591478	-3.33632115061521	-1.24510860667321
C	3.84295663222728	-1.22680787303943	-2.20653532538997
H	4.40687890853637	-1.72318301140963	-3.00630005944848
C	1.98908517140207	0.06334194691401	-1.68331209065541
N	4.34046951601853	-1.34290097693699	-0.97995541171459
N	2.74563536456670	-0.57715742156265	-2.59331374164265
N	0.89719481279758	0.71541279339631	-2.09321545356437
H	0.73579548770109	0.78276649080691	-3.09072737255368
H	0.43727400068452	1.38565368194573	-1.46932466995549
C	-2.93656914896008	0.52021715468381	-1.15578022366197
C	-3.71820674930733	-1.03582642134796	-2.68788705432107
H	-4.46881519934506	-1.25204707802991	-3.45785211754468
N	-3.03535952347965	1.73655458693905	-0.60378370336129
N	-2.82456517763503	-1.99119747595749	-2.45422891914927
N	-3.81242662522671	0.16049616272994	-2.10884684272273
H	-2.19705026523833	2.15845755200611	-0.19016960669427
H	-3.70001438054279	2.36898940099055	-1.03599100825796

Products for TS6A

N	-3.50940661827082	1.20467027421956	-0.96158151409303
C	-2.25103766576282	1.08216841374790	-1.42034879760509

N	-1.60104727302921	0.11562305019771	-0.79382656644368
C	-2.48590292622605	-0.42185256269197	0.12878801215001
C	-3.70215090559049	0.26345562317595	0.01566232844351
Pt	0.32043870296214	-0.39900803918844	-1.15644960882620
Cl	1.79577404476385	-2.08377200790631	1.44318646773638
N	2.27137309013532	-0.92100587691796	-1.42568732129573
N	-0.29934622324408	-2.28828783978955	-1.65753790863311
N	0.86717812187060	1.50077248775543	-0.74459687908512
C	1.18371370299351	2.38754759807616	-1.67593424757444
N	1.23720289569422	3.63231285890940	-1.16857557271656
C	0.92359311247814	3.56544282608069	0.16498809614032
C	0.71326465227865	2.20963163388275	0.43570679831191
H	1.45403083921068	4.47524151873930	-1.69402065756850
H	1.35948351724892	2.15744197629502	-2.72528424833113
H	0.44258898898324	-2.89433561414157	-2.01419502905243
H	-1.02079045174198	-2.25877338168822	-2.38252492223298
H	-0.70926566705356	-2.77900575968138	-0.85483889880117
H	2.90041931891972	-0.11982789923254	-1.50737497962147
H	2.46102572954007	-1.53067952977343	-2.22373486992627
H	2.50681276849714	-1.42676352453551	-0.55107384444511
H	-1.83773912413274	1.70574259454939	-2.21099163417319
H	-4.19843654596273	1.87234903857766	-1.29781370812849
C	0.45468200194904	4.14622120501507	2.23629985270323
H	0.30566174365234	4.92102840738866	2.99817867787343
C	0.42466726499568	1.87002810121490	1.78377695760484
N	0.81431103986808	4.57114338475115	1.02915831549896
N	0.26705859621481	2.89228421693629	2.64304021933817
N	0.29442570664192	0.61930121416323	2.23771808432861
H	0.17058341130733	0.52807686897888	3.24050496417550
H	0.75447012930434	-0.17833591695196	1.77377030303330
C	-2.44138748133701	-1.47319116159987	1.08291916803491
C	-4.66333259826998	-0.98584703138222	1.55200201604941
H	-5.54108762273911	-1.25182340097944	2.15320324650018
N	-1.38447698990607	-2.26784661193552	1.31653318541805
N	-4.81375805930527	0.02669392261485	0.70563171654091
N	-3.57237080464634	-1.71894851241363	1.76486606119370
H	-0.40399243670861	-1.99570096409392	1.15586712591047
H	-1.51238298558280	-2.93656858036653	2.06923761156787

Reactants for TS7A

N	4.05363841713122	0.13549519659829	1.57955342666102
C	2.81305269318599	0.64507863936500	1.45468297763774
N	2.30751083611195	0.38598527344037	0.25933421606774
C	3.27220494594844	-0.32964607983485	-0.42643675830129

C	4.38219059140538	-0.49057502543704	0.40369279069540
Pt	0.50234743034525	0.94198825140022	-0.43517996378191
O	-0.36730051493266	-0.63116437919077	0.46416220088920
N	-1.34910319723065	1.45905686716567	-1.13363267209543
N	1.34496427538296	2.55677084322131	-1.35319001013486
N	-2.46443349548844	-0.35390323196766	1.86475922061976
C	-2.93957775199917	0.13537718586814	2.99525728060321
N	-4.26299393737716	-0.07314113969330	3.06670897075715
C	-4.66986714574855	-0.72823994802994	1.92413182730699
C	-3.51566319715472	-0.90252267049128	1.16815528852916
H	-4.85835146398797	0.21278510702860	3.84107285189063
H	-2.35298444783913	0.63608564174336	3.76383071625659
H	0.83745382188128	3.42498675781203	-1.16375387165288
H	1.37772117261010	2.45331307759560	-2.37126168208434
H	2.30812753499073	2.71738420815747	-1.04851631173357
H	-1.85907275232545	0.63766112920984	-1.47291378614532
H	-1.34182377151338	2.13334023393900	-1.90229125620339
H	-1.92505755117087	1.87031186943687	-0.39375668151841
H	0.30373256813237	-1.22510529704864	0.82731349740742
H	-1.45091730108427	-0.36996132019297	1.45222682763444
H	2.30614880645433	1.19681826776981	2.24420584776748
H	4.64093697611381	0.20757694237980	2.40626434403402
C	-5.89740391219054	-1.72075039937493	0.38736532190655
H	-6.87260849371838	-2.07413189075537	0.03176465232165
C	-3.63614413013125	-1.55510942527667	-0.08354658538657
N	-5.89140733390117	-1.12295987534063	1.57654610565295
N	-4.86949167694488	-1.95018147035036	-0.43290353806910
N	-2.59223081703987	-1.76047302872421	-0.88875152191315
H	-1.64599669937419	-1.52963117791703	-0.55841722390968
H	-2.72920989753075	-2.27715174174192	-1.74937014810228
C	3.35170528263475	-0.90326744362137	-1.72094986150217
C	5.47773877468530	-1.61612436080503	-1.14188557467277
H	6.37547516914102	-2.15225675255768	-1.47253896570002
N	5.51017554316097	-1.12575921569422	0.09310612072348
N	4.49017853988922	-1.54157307886138	-2.03505210351760
N	2.36594259066621	-0.83600229985762	-2.61773330936530
H	1.49018934861245	-0.37321332446527	-2.39085294915706
H	2.48429417019970	-1.27597091490121	-3.52197971041551

TS7A

N	3.71234815982078	0.57349599405344	1.59644743931082
C	2.70506652882289	1.39921329561575	1.25944417450850
N	1.89408178186409	0.83494018222551	0.37776605057695
C	2.41047893387656	-0.42477384107630	0.12289056520756

C	3.56317309249125	-0.59468948877017	0.89368647001676
Pt	0.22512486599253	1.71206137242180	-0.34754508818070
O	-0.64106496008089	2.34865203899589	1.75331256802699
N	-1.45353419424591	2.68051404121602	-0.98833103777113
N	1.24830766999282	2.20883433508907	-2.01574865326216
N	-1.02287500759687	-0.24860520112917	0.61800225909386
C	-0.68658805587628	-0.81676039556490	1.75709023615440
N	-1.60280955976811	-1.72291395842761	2.17783439430438
C	-2.61116328417396	-1.74787977209017	1.25240364235556
C	-2.22272663321461	-0.82962645245327	0.26496057855696
H	-1.55390031989770	-2.27099892691099	3.03220083395581
H	0.21290343302958	-0.59348592661411	2.33141470350953
H	1.19337946050186	1.47294995349905	-2.72541349674802
H	2.24079909858684	2.34644847476740	-1.80772234959475
H	0.92166550109502	3.07296066700079	-2.45451539165747
H	-2.19358844785221	2.03293748301415	-1.29174912202110
H	-1.31184525496672	3.34102961210100	-1.75569455487840
H	-1.82923101786250	3.22049614091092	-0.20391866021313
H	0.02650581391180	2.49132318746362	2.44013465509244
H	-1.26629198862268	1.71993961762476	2.14247605762562
H	2.58679894530908	2.40169334887417	1.66757208199870
H	4.46001660338318	0.79042012680392	2.25051104598073
C	-4.49820538629928	-2.21303035703409	0.21141474628087
H	-5.45759921151172	-2.74194997023413	0.16788172368242
C	-3.08631728962843	-0.71933730839255	-0.84814393372589
N	-3.73550805983872	-2.45985214984643	1.26998205412273
N	-4.23128927000252	-1.40872711826807	-0.82142543388689
N	-2.82631346325087	0.06994379237460	-1.92403101436573
H	-1.85650719325245	0.17946348159841	-2.20377452657637
H	-3.46648306778702	-0.04638561233890	-2.70389526903394
C	2.05011118141733	-1.50567477682379	-0.72285152026384
C	3.91044122087263	-2.61807967493390	0.09372839175956
H	4.50413384271329	-3.53944431526816	0.05837992449285
N	4.34499772642733	-1.67130509315795	0.91881744281283
N	2.84030224179537	-2.59034987770110	-0.70030712626148
N	0.99382956083968	-1.48325095741350	-1.53749776134145
H	0.32018089646436	-0.73015131087411	-1.46756410696787
H	0.75947510652114	-2.31414466032691	-2.06665299267651

Products for TS7A

N	3.34990179208695	-1.04440034044078	1.51342016449986
C	2.51328327614281	0.00723126805619	1.45038585977564
N	1.91479320050278	0.07811479304677	0.27256041751310
C	2.39233038280473	-0.98719812845739	-0.47317396029028

C	3.29695921380390	-1.70665159334683	0.31402311982965
Pt	0.47023258603993	1.41272475477164	-0.20632332520271
O	-3.32980998347833	2.25918028861001	0.86324362619527
N	-1.00217090177514	2.74010059670139	-0.67729724955034
N	1.75493456004219	2.42239610685413	-1.43981647999977
N	-0.78982331521324	0.52158067389001	1.09654803839390
C	-0.86747375321939	0.86453651380267	2.37248194909080
N	-1.85969578346538	0.20637862480744	2.99686707984516
C	-2.48207213649125	-0.59856927434532	2.07986589521444
C	-1.79371920378361	-0.40472844797872	0.87963854853048
H	-2.11232992610269	0.30978113603904	3.97586986514170
H	-0.22201101477109	1.58807145576100	2.86748924377986
H	1.36881729149435	2.61092725270371	-2.36920013321429
H	2.64818290438642	1.94481480682530	-1.58581965389482
H	1.98270989953157	3.33345974897484	-1.03124253387704
H	-1.25692418635061	2.67807533770963	-1.66622399176481
H	-0.71702117035622	3.70830438791968	-0.51101756538480
H	-1.87154284308519	2.57669583718598	-0.12764447593989
H	-3.81468636195055	3.05509982901542	1.11897552006976
H	-3.97372622471072	1.74804447080608	0.35470828200000
H	2.35600554961008	0.69876996123002	2.27652907820600
H	3.91641481833736	-1.30017296358312	2.31826505378995
C	-3.88594455210089	-2.02120908102225	1.15376886264588
H	-4.74917894041437	-2.69349806493797	1.22818947408227
C	-2.26035989284302	-1.13747849482085	-0.24069643776164
N	-3.52964862464219	-1.39645958429589	2.27089370067194
N	-3.32352654539184	-1.93506289133136	-0.05123544574041
N	-1.71588787778233	-1.06749991656757	-1.45778997008971
H	-0.84876684156448	-0.57098719047205	-1.61667476365699
H	-2.07229990120079	-1.67119417811099	-2.18856019752178
C	2.18130091235497	-1.46898270665711	-1.78981131764751
C	3.64855704195914	-3.18686404772276	-1.27939474959309
H	4.13803295011683	-4.09820919769839	-1.64276279728829
N	3.95154220980964	-2.80807629829462	-0.04300173600481
N	2.82501399635046	-2.59012618706005	-2.14187508572465
N	1.37290324741680	-0.88349550956235	-2.68643089712790
H	1.04182419941188	0.06285679323843	-2.54908287237269
H	1.35487994849059	-1.25628154124304	-3.62864413962749

Reactants for TS3G

C	-2.58309816212998	-1.15549896688244	0.02525433308899
C	-2.53367326079156	0.26236283431656	-0.14235475114478
C	-3.72339962400462	0.98606663111369	-0.01622246508011
N	-4.94678928080384	0.52524483087905	0.26626952851303

C	-4.99107256685553	-0.78247481138243	0.44085543349636
N	-3.87907990688536	-1.57849485193519	0.32625502157584
N	-1.49634179440906	1.12317474667596	-0.42800982706684
C	-2.03272013544623	2.31870455717607	-0.47827329460433
N	-3.37157482506619	2.28613408176871	-0.23970594452413
Pt	1.88493658596931	-0.79087043355835	0.14106808225685
O	1.07975021881534	0.96365080543689	-0.54127928021862
N	-6.14830190611208	-1.38067741567090	0.73799860207685
O	-1.67977202505239	-1.98665333336730	-0.06077066406850
Cl	2.99914122762810	0.38583502211873	1.79034402992007
N	0.83629044003570	-1.77510773194820	-1.30829983227365
N	2.71093386998834	-2.50903353195250	0.79044475037646
H	-3.99718821162904	3.08628274725870	-0.22525310067606
H	-1.49601895581702	3.24540326685415	-0.67917920520497
H	2.01314999083267	-3.18934278192745	1.10292593284869
H	3.32581432988098	-2.32685498446580	1.58825300906810
H	3.28020722878395	-2.96960776728937	0.07543031063814
H	-0.14070588147259	-1.88201435655378	-0.97479564964216
H	1.19316705946208	-2.70471359200590	-1.53681457843782
H	0.81366514412462	-1.24865505947599	-2.18427269402988
H	1.38172607045242	1.67471620794596	0.04628552087661
H	-6.22089547001863	-2.37664492836609	0.89684131495543
H	-6.97588876406593	-0.80728719514006	0.83593775507052
H	-3.97613499636007	-2.58266203209951	0.46093571124727
H	0.03852360094676	0.97639804247684	-0.49698804903737

TS3G

C	-1.24302472174812	-1.03278298509766	0.73396337719840
C	-0.99282030404270	0.28655951740477	0.23197018020393
C	-2.08624957313468	1.08795375058013	-0.10771301105948
N	-3.39233237203154	0.79426460781215	-0.02375162258116
C	-3.62820510819059	-0.41730199390225	0.44084403768349
N	-2.62457193517652	-1.27963156247948	0.79936362082211
N	0.18073110081700	0.96623839536308	-0.00616637239734
C	-0.18245844747069	2.13567224539942	-0.47510844639968
N	-1.53981191062767	2.25620753436685	-0.55345537306199
Pt	2.44738082356879	-0.13496036922343	0.18574543660371
O	2.70144676591709	2.22137398137390	-0.16483140946108
N	-4.88842599843142	-0.85570900473211	0.56381963666690
O	-0.45269877901797	-1.89903969052038	1.08313107496856
Cl	2.77778573413062	0.31390697237228	2.43588893736656
N	2.18692266566297	-0.50393335930931	-1.80864871352516
N	3.17719073317892	-1.98414683057045	0.48792340370769
H	-2.05303334720410	3.06802545832513	-0.88301915589088

H	0.49913785295218	2.93040995905323	-0.76983446184768
H	2.46276623193748	-2.70621066098074	0.36467394254613
H	3.51509651217963	-2.05824321723901	1.45170077290332
H	3.96099936996164	-2.21733731191819	-0.12603689521439
H	2.00800806523637	-1.48546796882292	-2.03478192709809
H	2.99690801238243	-0.21736172643283	-2.36374045204073
H	1.38389635933021	0.02844711266071	-2.15242886281375
H	3.56795967479158	2.33959149801604	-0.57883977203044
H	-5.10845806066957	-1.73925861913798	1.00336000987002
H	-5.64168661403745	-0.20964298040972	0.36833967288641
H	-2.87186068348292	-2.20285966251023	1.14829262797823
H	2.83940795321899	2.51523691055898	0.74933974401640

Products for TS3G

C	1.17194351029141	0.66553956525702	0.79740326407655
C	0.03712909300766	0.83068379458970	1.65162081944114
C	0.11835935646955	1.75621634571939	2.69199738316148
N	1.15301643819861	2.52909022230226	3.02848545507706
C	2.20631784557952	2.37872956754882	2.24562631397595
N	2.21474019141225	1.50436542666428	1.18779997462851
N	-1.22952549964961	0.27270446057205	1.65092823829249
C	-1.88281747873974	0.83099814933576	2.65101205453519
N	-1.10270072002652	1.72161687215110	3.30408688245777
Pt	-2.05963815062412	-1.00483962713151	0.32248999365580
O	-1.99646895550233	2.93582718621129	0.10349491564243
N	3.31308546573938	3.09022679681708	2.46767228036388
O	1.31648517133760	-0.08580321313308	-0.16539839117680
Cl	-3.93682577254971	0.35258586984778	0.14989184248392
N	-0.41667177043262	-2.20911089961835	0.45163090371166
N	-2.92240165021046	-2.28097645370190	-1.01303871054483
H	-1.38446985953711	2.27400081371146	4.10943779400399
H	-2.91691781595018	0.62774383674658	2.91813226967727
H	-2.36187260164597	-2.40663111732939	-1.85978278155658
H	-3.83379986468892	-1.92324061799189	-1.31051169483102
H	-3.08287836552649	-3.21257604504002	-0.62100782576512
H	0.39741355184311	-1.64818597116592	0.14693660446450
H	-0.46090234338504	-3.04655643965207	-0.13163939236190
H	-0.24358226549259	-2.52424602562969	1.40883519425578
H	-2.06965701563054	3.32311095569160	0.98408288041015
H	4.14774894821626	2.98793897655847	1.90588612505514
H	3.32668340869150	3.73007343136809	3.25142571012830
H	3.05326306289956	1.42928534440323	0.61484623742221
H	-2.67087136409443	2.23557519489782	0.11812487931502

Reactants for TS4G

Pt	2.53192542003921	-0.77543440228823	-0.14775279534134
N	2.68901794582770	-2.47403880078517	-1.21949676180123
N	1.90474129264636	-1.78563150615977	1.51266583470522
H	0.89867945680977	-1.58990759815768	1.67420914037157
H	2.41784886201362	-1.47528828715738	2.34068567507167
H	2.01683939537021	-2.80003985197344	1.46556790126144
H	1.79008106222651	-2.95133374227340	-1.32755939160194
H	3.33876763323029	-3.14984198741768	-0.80881757953570
H	3.03074505869696	-2.26722841253015	-2.16206205552212
Cl	3.24023081895609	0.45107288597701	-1.97633106773824
O	2.37721673934457	0.94843963133640	0.95003508518326
H	1.37364001730845	1.22010797046095	1.03604170846100
H	2.79030041955046	1.66109581662771	0.43660658171904
N	-0.09588880499851	1.69001369856183	1.08509380758031
C	-1.29468399602723	1.04108906207131	1.28537773758651
C	-0.40641047182744	2.93974094794040	0.83784550908614
C	-2.34864504615394	1.95001167528473	1.15083528814280
H	0.30305776970933	3.73982105701029	0.62870912196250
H	-2.22558166370699	4.02778844499905	0.70498458036358
N	-1.75089168101888	3.14469500714229	0.86868972943474
C	-3.95463139534722	0.46251723243848	1.54113309425569
N	-2.98976257624002	-0.50136796736537	1.69413311046044
N	-3.66131850091987	1.71970245531497	1.26539183952491
C	-1.60899523292631	-0.32141202657246	1.58042399064219
O	-0.86142073820463	-1.28522952644322	1.73331159804189
H	-3.27431223008805	-1.45543843746696	1.90703597248012
N	-5.22970534731112	0.08651525493480	1.67958443971405
H	-5.95317688737453	0.78559461391774	1.57383221564356
H	-5.49647731958474	-0.86639320742707	1.88760568984783

TS4G

Pt	2.30906437783471	-0.52431001099955	-0.26365972595636
N	2.45481785945837	-0.95939050083384	-2.22044019998891
N	2.87286089563459	-2.40978007282982	0.29170120545370
H	2.12351998716631	-3.09659398618920	0.18020599624725
H	3.12887012404988	-2.41068107099878	1.28220315284553
H	3.68405044480009	-2.76536272714743	-0.21887458994554
H	1.54324799854539	-1.12173611864355	-2.65551779170232
H	3.04189243538929	-1.76443869820849	-2.44958169581378
H	2.87213240800789	-0.13189719945461	-2.66273875000674
Cl	3.30688743194850	1.77106504075192	-1.10068154188876
O	2.15048505416285	-0.11664176339162	1.75169956925601
H	1.16498354539167	-0.24574547831619	1.98378419876825
H	2.36507873925818	0.80786735245768	1.94587833911199

N	0.34382342931945	0.81169801468950	-0.54205937846279
C	-0.83600886352064	0.62356393678886	0.13936664119332
C	0.03085524884374	1.52253050284420	-1.59628329993820
C	-1.88898959113186	1.23507431158343	-0.54815255807921
H	0.74473571175811	1.86622015363138	-2.34352057439383
H	-1.77978311656355	2.33418681528041	-2.36656362262744
N	-1.30611147520997	1.79730191418977	-1.64594827320221
C	-3.44437803382377	0.64635063377054	0.93051805266682
N	-2.46628583084089	0.04987985489415	1.69206121948588
N	-3.18016225866066	1.26896420788484	-0.20345996286573
C	-1.11423883995602	0.00585460693230	1.38729288432928
O	-0.32393085400893	-0.52274460440324	2.18630124065568
H	-2.72508892735421	-0.38587298153913	2.57520341300504
N	-4.69895532404210	0.58093849179954	1.38384053217877
H	-5.43096499160932	1.01860096235756	0.83966688884386
H	-4.94240758484707	0.11509841309940	2.24775863083048

Products for TS4G

Pt	0.69331666947383	-0.88547789663974	-0.45115152623749
N	0.04425964716362	-2.67329587953758	-1.11862320345994
N	2.45943767279183	-1.70477610809245	0.16663101220592
H	2.40578382085928	-2.70304386762938	0.37985465702364
H	2.76205821639472	-1.21783327313623	1.03494675174645
H	3.20032061185125	-1.59221828596791	-0.52958005249552
H	-0.73041497803132	-2.58255930412153	-1.78109215280501
H	-0.29893964550228	-3.25293808196974	-0.34707071722656
H	0.77413721559676	-3.21271645052166	-1.59136650144684
Cl	2.67429549533723	0.21164021182036	2.67349568458031
O	1.30669384729689	0.94935560310114	0.25495843182507
H	1.93966200826292	1.36223520875132	-0.35288526453860
H	1.81342365585969	0.80858569638836	1.13828085635383
N	-1.03564056982727	-0.02626448471085	-1.02652704382904
C	-2.12177749265079	0.22971192721452	-0.21499921622321
C	-1.33248235438301	0.45456137202620	-2.21892290413877
C	-3.09493548371907	0.88875908324603	-0.96118177449546
H	-0.68799740070735	0.42040765519927	-3.09482384815973
H	-3.01702743774470	1.44836406132727	-3.01615824068897
N	-2.56569724764422	1.01183938322759	-2.21694340836386
C	-4.53817549203836	1.06159893460585	0.72173178725269
N	-3.64007167654094	0.41537885725924	1.53368112279584
N	-4.29264720714264	1.31654141880927	-0.55179708081435
C	-2.37355008712305	-0.05958792176112	1.16584007882809
O	-1.65809073755744	-0.62686234686083	1.97667653865574
H	-3.88928909295174	0.24672970998545	2.50614006746616

N	-5.70111268684145	1.43325511197173	1.26245980493162
H	-6.36716641225296	1.92676870138815	0.68228788973243
H	-5.91850028822940	1.28419591462729	2.23877974152555

Reactants for TS5G

C	-2.70322005950437	-0.57016119565190	-0.28798526371215
N	-1.90229220956319	-1.65427479675927	-0.57030101146586
C	-2.72441692482163	-2.66865753698338	-0.65737316509878
Pt	1.96833748850119	-0.49253433683982	0.03497335426280
N	0.86719590354155	-2.16410017514935	0.01841526905766
N	3.27660952679696	-1.28525525645288	1.34392155133109
H	2.83078751428914	-1.59311137432549	2.21278244877911
H	3.98943734475258	-0.59880452383821	1.60748691961418
H	3.77302676846273	-2.09494785179187	0.96119662787294
H	0.84685902190760	-2.64701304470976	0.92008910942473
H	1.23305867936949	-2.83448583749978	-0.66378363738759
H	-0.12800271648028	-1.96072495879808	-0.24405298457434
H	-2.44062105716311	-3.70039673308050	-0.86486763323483
H	-4.83132330416561	-2.92442339932373	-0.46496649337430
O	0.69441635710013	0.40954645183375	-1.28321649275769
H	0.38597853003498	-0.16737155635189	-1.99787786376310
H	-0.14560485998913	0.77315253527448	-0.80474148022004
O	3.12001271355934	1.23650602242061	0.06426314568605
H	3.55633203297233	1.40235827332174	-0.78807833427111
H	2.60096982492469	2.03626673030473	0.25165319038280
N	-4.02510393907308	-2.30680952059046	-0.44710531973125
C	-4.04412243121454	-0.96685602905976	-0.20049954992108
C	-4.82101975120741	1.06082769856923	0.28394476153382
N	-5.11081743035044	-0.20984929402972	0.07459858370712
N	-3.53716523241895	1.54557276953633	0.20322487966992
C	-2.40463256845560	0.80346727699937	-0.09015048162652
O	-1.30825340929407	1.38724564976853	-0.15314731673976
H	-3.37343400271166	2.54014989884458	0.34882923015980
N	-5.78761948575834	1.93416287541567	0.57829616587465
H	-6.73523168152326	1.59156619200878	0.66928143504569
H	-5.59526064251795	2.90373504693809	0.79346035547605

TS5G

C	2.01903685093272	0.19151058650480	0.00041983927890
N	0.71402016652442	0.62628049911549	-0.11404440999504
C	0.80080542388960	1.89294901547376	-0.44283144389661
Pt	-1.49378394928731	-0.59041983836050	-0.15649304512717
N	-0.48746540618055	-2.25364188638212	-0.63014629025964

N	-2.94937589306226	-1.12318096542426	-1.44233835421247
H	-2.62478261606617	-1.15415048345058	-2.41246766002568
H	-3.72408692433900	-0.45474346674684	-1.40908887032044
H	-3.34596471384916	-2.04170968692110	-1.22643342745364
H	0.42129414420918	-2.28841686263923	-0.11868284042580
H	-0.27262252046604	-2.30417745293281	-1.62922426546684
H	-1.01176339719969	-3.10053532805979	-0.39627334294836
H	-0.03799477078072	2.56521361310810	-0.61888013176763
H	2.40278824108928	3.24780407456611	-0.78759261061641
O	-0.73158737956712	-0.30803452167418	2.06079086817946
H	-0.75490108377401	-1.11688991930016	2.59201374878538
H	0.19314575562885	-0.02176198734087	2.06557285847177
O	-2.61337846937347	1.07249946991147	0.38838663509485
H	-2.41825988323321	1.29549994933132	1.31503621235740
H	-2.43200511601033	1.87799024105276	-0.12303107773236
N	2.09374198760183	2.31030822628588	-0.54729750287796
C	2.89855902115155	1.24567023849232	-0.26518627823992
C	4.74622187297903	0.05444702643520	0.07407691468012
N	4.23540482204681	1.22904504212057	-0.24644641349642
N	3.96360675019387	-1.03800149358479	0.35818246776463
C	2.57092108000574	-1.07654063221997	0.35150685626300
O	1.98345634489932	-2.12527568867855	0.62653602642506
H	4.41429344894815	-1.91967561536657	0.59495215247963
N	6.07088890522818	-0.10578985797411	0.13399460969213
H	6.66241900075300	0.68449580335921	-0.08749683518617
H	6.50276830710748	-0.99237809870055	0.35820561057621

Products for TS5G

C	-1.68544039399773	-0.19851094008660	-0.18865262199406
N	-0.55895852837640	-0.83552891051413	-0.68721132585424
C	-0.98815290037747	-1.93790282002845	-1.27269462416487
Pt	1.36103249677556	-0.20459768417167	-0.58145128635037
N	1.67173520165679	-0.71522077867040	-2.51840951752978
N	3.27760742661768	0.45171389577960	-0.39396460367840
H	3.96718215339259	-0.29963818559091	-0.48132188778072
H	3.40292844087861	0.86139416083954	0.53587149629879
H	3.53694126840255	1.17355382687647	-1.07167660813683
H	1.94035663320962	-1.69900261814196	-2.61641067740491
H	2.41492107485269	-0.16918610528319	-2.96195750012442
H	0.83927652674943	-0.57663927096850	-3.09770372108000
H	-0.36300573933381	-2.67735225432323	-1.76994963437137
H	-2.88922773910076	-2.80437083149131	-1.57168380051023
O	1.11411124098665	0.36786696703205	1.35962437035532
H	0.34269595011053	1.01015506984595	1.34389017607720

H	0.81484769536029	-0.42957978808042	1.90231698821482
O	0.34857878740275	-1.77621138292048	2.56033340264605
H	-0.60768739763347	-1.77134585335783	2.71036266571623
H	0.72871086968572	-1.91690546853701	3.43882735586449
N	-2.33264628116551	-2.04311635676217	-1.19112293186131
C	-2.81194011551656	-0.95540895906244	-0.51750923744766
C	-4.26727790664306	0.44248791909275	0.41859129338542
N	-4.08761546202958	-0.68670070209169	-0.24516277996433
N	-3.21717422554399	1.24023432462582	0.80424485227745
C	-1.87268685971679	0.99486687400921	0.57010600858343
O	-1.02087449688283	1.76921640288887	1.02187936387639
H	-3.40914931458511	2.08471342133858	1.34080190860694
N	-5.49609278406470	0.84233917020802	0.74075013922371
H	-6.27961707672472	0.26178522409839	0.46946669602623
H	-5.67415154438890	1.69628565344716	1.25328104110105

Reactants for TS6G

N	2.27822037694184	-2.84359239770156	0.89288647182873
C	1.25481329428252	-2.06336680162127	0.46983773115828
N	1.70910030843717	-0.93453044884568	-0.03954514171797
C	3.08527395123542	-0.98180743070875	0.05096275645124
C	3.45279767757410	-2.18834578809826	0.64455258378031
Pt	0.53121918630142	0.56457732142982	-0.68590867805456
Cl	0.91823848641192	1.77415948667731	1.25284147305332
N	-0.73294194675198	2.03394958269037	-1.32250721840385
N	0.08108843624165	-0.49939872572058	-2.37121157653893
N	-2.14439367167653	-1.88800894353020	0.34700341763189
C	-2.53617241613301	-2.54962647402343	1.40412910419917
N	-3.88181741611697	-2.43357928478664	1.63269029988138
C	-4.39249701377666	-1.63762548833301	0.65348403484337
C	-3.29005794814390	-1.30290515621509	-0.14568434367863
H	-4.40493623565377	-2.85759857039058	2.39250826647643
H	-1.89617920008780	-3.14184243122045	2.05891833106442
H	-0.94956121796483	-0.55898872840501	-2.42878680922039
H	0.45815419572667	-1.44881719165215	-2.35783497791365
H	0.42145270310945	-0.05564418060952	-3.22692453211658
H	-1.61428073824877	1.61447809966935	-1.64771067741673
H	-0.34798041245881	2.58717283475445	-2.09142688631345
H	-0.95059327177598	2.68588448065800	-0.56540455069945
H	0.19861039510831	-2.32953927694813	0.53350979670100
H	2.18291857632759	-3.76112184549969	1.31864200941380
C	-5.87338912737973	-0.47856669448497	-0.53473564279608
N	-5.67008865409150	-1.26400690551472	0.50487477718870
N	-4.85766311150561	-0.08889030353103	-1.37159729614287

C	5.63948603258049	-1.79445148266014	0.54716054626821
N	4.68569765168036	-2.63192589504198	0.91303291137766
N	5.37529562456043	-0.59398550004170	-0.06126221857435
C	-3.51471560100500	-0.44813382709239	-1.26399618100490
O	-2.70893029584494	-0.00966205331709	-2.09399750238803
H	-5.07089540534190	0.52503657429272	-2.15489230020688
N	-7.10153809155819	-0.01873609197419	-0.80188725235326
H	-7.86852650529080	-0.33675061135897	-0.22406283876247
H	-7.30840114481134	0.52035131391631	-1.63205718116025
C	4.11060688599749	-0.07820587104456	-0.38226898346716
O	4.00714568787786	0.99326235954837	-0.95846121588323
H	6.15494200742664	-0.00000203536398	-0.33584426057354
N	6.92052149704895	-2.10411364011269	0.76755773581422
H	7.13520608478839	-2.99295814967868	1.20031815123855
H	7.68146036595936	-1.50472382810945	0.47717786701663

TS6G

N	-3.30821063572764	-0.67728905870029	1.96405957652708
C	-2.24133075198054	-1.35937645441879	1.48514482398989
N	-1.79805804801214	-0.81939560038942	0.36723960344996
C	-2.61575942920426	0.26701757808846	0.10479095508257
C	-3.57022921194702	0.36377051728568	1.11933849024043
Pt	-0.12320831027977	-1.40539042343324	-0.61367545352785
Cl	0.58544249126278	-2.97482027488329	1.39912939612174
N	1.59856293737802	-1.91452909361337	-1.56252469149364
N	-1.17173013663052	-1.39179151302190	-2.37759138825067
N	1.16458453866363	0.09266791893853	0.69782435876194
C	0.94366827755751	0.33135323505044	1.96593005721290
N	1.41463554536206	1.55628787730119	2.34104010891025
C	1.97646566075125	2.14071147102222	1.24206293286013
C	1.81488851706768	1.20557503444358	0.21639029353863
H	1.35853798601683	1.96004354191440	3.27117539297161
H	0.45511891965130	-0.35987623990927	2.65103530668968
H	-1.49604571630550	-0.42372515148824	-2.52738187904396
H	-1.98986961378125	-2.00266056318083	-2.35271891095909
H	-0.62431104628973	-1.66821111102997	-3.19400598362918
H	2.06495511359201	-1.03500697344494	-1.84472693937605
H	1.49428348505340	-2.50337993048850	-2.39056174475630
H	2.20872509527538	-2.41668752325463	-0.91436726665915
H	-1.77748786927699	-2.21901129355735	1.96817641396717
H	-3.81778300777301	-0.89993512854447	2.81444606282864
C	3.02640616086925	3.63791253938753	-0.02449123903107
N	2.55761489286213	3.34551341253947	1.17405805575815
N	2.93735076398543	2.77219719716999	-1.08697146520157

C	-4.55132031375979	2.18462794746754	0.29967319815800
N	-4.53625864631921	1.27542667677736	1.25731228830251
N	-3.65564437342356	2.17013712775144	-0.74006327504263
C	2.35622470754762	1.50161736893210	-1.07136172726548
O	2.37388423055158	0.80485052977325	-2.08599067762371
H	3.34522977102418	3.04458547313736	-1.97884401434975
N	3.62053672845402	4.81584488391643	-0.24359461950340
H	3.70485330797400	5.46554148770551	0.52694612646329
H	3.99448499240025	5.07477585283662	-1.14631781415895
C	-2.64049439846148	1.23461662128361	-0.94889214100887
O	-1.92815927074017	1.31644669027497	-1.94716463721084
H	-3.72050498537692	2.88851104481534	-1.45911041843555
N	-5.46177526913642	3.16085666526266	0.31927458501323
H	-6.12569696038744	3.18657435598038	1.08236552487565
H	-5.50673612848691	3.87853328430243	-0.39200726519574

Products for TS6G

N	-3.00869823886891	-0.72578766712630	2.19384445734812
C	-1.85216579948331	-1.18923725686649	1.66302977047168
N	-1.70758196592423	-0.75861342728286	0.42295873010239
C	-2.82440582258626	0.00610827673740	0.13349317870887
C	-3.64855760931833	0.03694723756423	1.25672380222050
Pt	-0.10453851786049	-1.00670181500131	-0.78669078923819
Cl	0.46764665584851	-3.43350776911311	2.45374704138853
N	1.49746424479716	-1.15132262768392	-2.04221045445688
N	-1.35278115375975	-1.90754546617076	-2.12577022558666
N	1.09483744435186	-0.00616234963463	0.49841767161902
C	1.38637880024564	-0.32177741276844	1.74700597976407
N	2.12136548340567	0.65868652980324	2.32401811572548
C	2.32118295828405	1.65562827949006	1.40949862394855
C	1.67698707015438	1.22494168327258	0.25140537404345
H	2.46967486982490	0.64519010904479	3.27843279268173
H	1.10859530530249	-1.27299516390127	2.23057761622640
H	-0.89856547067682	-2.34802628001788	-2.92778774053911
H	-1.97251533216558	-1.16496270265730	-2.48140401275926
H	-1.94188933689904	-2.62261074545391	-1.69315113388682
H	1.66364985457049	-0.20233913564056	-2.40689894933935
H	1.38435136493948	-1.78072757831685	-2.83887935819436
H	2.34686528401526	-1.44547592307919	-1.55469033715560
H	-1.15193605162866	-1.87217010397305	2.17212356998901
H	-3.34549343887609	-0.92370010009925	3.13184486131173
C	3.04301051634208	3.56534138410863	0.52563566281571
N	2.99539527922161	2.79350888305361	1.59735131857278
N	2.47523404252290	3.20244125208954	-0.67040297102903

C	-5.20664971881392	1.31667026666892	0.31649749813265
N	-4.81793588883880	0.66670549074404	1.39927796049285
N	-4.47661473235949	1.31361178081195	-0.84636864843099
C	1.77898726759334	2.02043342324331	-0.93204880632877
O	1.37155248258551	1.77588638292090	-2.06442108595474
H	2.58255415012411	3.81905589262567	-1.47308781897269
N	3.66550097658263	4.74510815892911	0.57777052667551
H	4.09238228343012	5.02771161298630	1.45044099171588
H	3.71893878549989	5.36714869492914	-0.21790005430250
C	-3.26586438634871	0.65303080955111	-1.06269272208103
O	-2.74262755403405	0.66254218894591	-2.17390098583103
H	-4.84268596113377	1.80454184949662	-1.65955795972234
N	-6.35307423010084	2.00116171739928	0.32248356907435
H	-6.89738753024613	2.02319057165683	1.17512469200702
H	-6.67334437971886	2.53192604871385	-0.47680875122693

Reactants for TS7G

N	-4.76960612088859	-1.30469455122892	0.30036680590168
C	-3.64474146245548	-1.80422054334755	0.85843510494672
N	-2.57857113292736	-1.12615656116639	0.47664932223935
C	-3.03715015723741	-0.14233991464901	-0.38532898582054
C	-4.42541775364195	-0.24526332706326	-0.49043818404427
Pt	-0.67779874341321	-1.44328473479216	1.11651371641238
O	-0.02856043397883	-1.19704001175240	-0.79920822937583
N	1.24252674679327	-1.73974460819064	1.72387683368113
N	-1.34430628709635	-1.68572989364175	3.01445438059791
N	2.52965325426248	-1.10199814389183	-1.32439982671591
C	3.04443283184427	-1.57576989521524	-2.43309341756872
N	4.26493160934529	-1.03353037038873	-2.70129537382359
C	4.55430448934204	-0.14517765168096	-1.70506121772063
C	3.45555132768040	-0.20362275423741	-0.84455053882437
H	4.85029019484445	-1.24815418743047	-3.50341178832638
H	2.57447139563492	-2.30793767661115	-3.08889548654520
H	-1.50323721436340	-2.67133124906198	3.24464359795242
H	-2.22743038303210	-1.19805384834317	3.18625742537799
H	-0.69042305632696	-1.32600944836037	3.71508418340617
H	1.70477025206553	-2.46373182283982	1.16787626627920
H	1.34605733541535	-2.02312223751195	2.70015072587539
H	1.78323494564403	-0.86024336358305	1.59584248721539
H	-0.33738518065239	-0.28232279990513	-1.05977525494931
H	0.98631910259473	-1.20691913723273	-0.94383160062269
H	-3.64576453104697	-2.65652621064826	1.53548584500326
H	-5.71017850162924	-1.66078128451433	0.45017618602982
C	5.64677274838923	1.38780358335579	-0.51990429997528

N	5.64923405737564	0.61496698305239	-1.59076476673480
N	4.61906545439823	1.38484724447524	0.39184233678267
C	-4.64517701172606	1.47968847763850	-1.87912864986630
N	-5.25183634467882	0.51879880844854	-1.20329296041985
N	-3.28163574106328	1.64581475245931	-1.85131327426671
C	3.46486179989333	0.60555409805004	0.32959471396410
O	2.62641540440543	0.67009250087441	1.23070455163436
H	4.68483689244465	1.98502585480325	1.21128748418250
N	6.67140280736232	2.21288308529958	-0.28560495700634
H	7.43928355473723	2.22821432143509	-0.94402952739919
H	6.70103697196671	2.82937743190387	0.51591527399499
C	-2.37551524586878	0.86532896148554	-1.14730238741117
O	-1.16447336366727	1.08599554472517	-1.25070486909162
H	-2.85762327737083	2.38149714602185	-2.41426859131271
N	-5.35825146111721	2.32562314327057	-2.62182125449761
H	-4.93490379515258	3.05634357880380	-3.17907812488207
H	-6.36191597710440	2.20108071118573	-2.66204367427636

TS7G

N	-3.27935871622317	1.72596084947037	0.58949769556090
C	-2.33468227967589	1.13538254325691	1.35505073284411
N	-1.92856606026059	0.00001817830080	0.81981927400912
C	-2.65090170689402	-0.15639412159479	-0.35259095540415
C	-3.49973456121850	0.94126339690930	-0.50697462750110
Pt	-0.41736805781464	-1.14492167596871	1.54365703625058
O	0.52502778710377	0.60515456617237	2.84522751451646
N	1.10931010757586	-2.25998319056271	2.28231208658807
N	-1.56253730772604	-2.78683674829180	1.31505797524390
N	1.13855746635584	0.29329181762052	0.18072726514564
C	0.68542336459464	0.98890196902301	-0.83410957351831
N	1.68858978481053	1.59174437603783	-1.53278732528877
C	2.86872193549881	1.26573056551715	-0.93072895465890
C	2.50919374261666	0.44541965314279	0.14389711612165
H	1.57737940617740	2.17904745236663	-2.35387726543132
H	-0.36097693589487	1.09384702949505	-1.11804136840095
H	-2.44350778404205	-2.71186204722035	1.82855030052103
H	-1.11426133457232	-3.65226005663797	1.62184789624488
H	-1.78168059981253	-2.87177959035599	0.30709537465715
H	1.99808500021815	-1.71988014892667	2.22891187263296
H	1.26410010324358	-3.13471138309544	1.77659369146118
H	0.95562598356724	-2.51110576845337	3.26155487231261
H	0.72316967171953	1.38168025892757	2.30270208584923
H	-0.01353420273483	0.92787704776292	3.58109700469803
H	-1.96300426130558	1.56594002848231	2.28111334428470

H	-3.73668309167452	2.61036412797335	0.79439842711374
C	5.05423738437780	1.18798235609633	-0.52579912248630
N	4.09380718346799	1.65624077607406	-1.30093193680338
N	4.80146626101024	0.37572539841123	0.55130634499199
C	-4.33678299582579	0.27323845260979	-2.45591923044955
N	-4.34103964807177	1.19610955915208	-1.50989187687794
N	-3.54644553929908	-0.84680767374367	-2.38485291449141
C	3.54889574380382	-0.06697826508122	0.97871523958819
O	3.46925947354673	-0.80599973061371	1.95973238938505
H	5.58394656189695	0.03408156113621	1.10550701362133
N	6.32993023722723	1.49895282607378	-0.77536603021356
H	6.53566407348022	2.09409859414568	-1.56693327108108
H	7.09375501598561	1.16130277972516	-0.20511406700115
C	-2.66134362968743	-1.17383735504839	-1.35891072240463
O	-2.02943441878351	-2.22704944555223	-1.40219648900500
H	-3.59947705753167	-1.53513491209780	-3.13361752342429
N	-5.11967911855431	0.40888814128222	-3.52658625780441
H	-5.13125194575068	-0.26613672490268	-4.27996266993262
H	-5.70185503492477	1.23343453298208	-3.59918037146366

Products for TS7G

N	2.58698418992995	-3.15383324561121	-0.08886837969448
C	1.58839033323424	-2.64682774667088	-0.84568602731592
N	1.40538359352864	-1.36448810782558	-0.59574124385894
C	2.34558313608215	-1.01785278179921	0.35989573986566
C	3.08980769293105	-2.15114725129713	0.69172720442641
Pt	-0.01482442224157	-0.15240296984646	-1.38687883343109
O	0.10474211916232	2.99560702658423	0.11743939904003
N	-1.43796076672397	1.09075239920329	-2.15222134653802
N	1.50212908493955	0.96391711299415	-2.16571244128623
N	-1.51441079345827	-1.23823719031471	-0.55558498440154
C	-1.75550655176943	-2.52207904969254	-0.73965553953972
N	-2.76549150840449	-2.94852704328764	0.05045461851863
C	-3.21529549205903	-1.88879572636712	0.78636349943795
C	-2.42758342390398	-0.80536331188858	0.39229626784133
H	-3.12862053156893	-3.89770937948772	0.08237322963028
H	-1.23905470722657	-3.17578121112369	-1.43765816913775
H	1.22041679139474	1.65125527282127	-2.86743976913656
H	2.22707611734585	0.38631803786565	-2.59792769668615
H	1.94088527631353	1.47579256310881	-1.39003611712001
H	-2.16480234362155	0.58204633507259	-2.66087525362897
H	-1.08592542038333	1.81011386511682	-2.78676002369842
H	-1.89071495788383	1.57012807712203	-1.36209912044160
H	0.81560864882230	2.40249521836382	0.41012822649822

H	-0.69673982901267	2.51274837291290	0.37558215021624
H	1.03575267841993	-3.24160484202666	-1.56907368871067
H	2.90985472003316	-4.11768141854793	-0.10902147662834
C	-4.44664887238500	-0.72537640249409	2.22748239538723
N	-4.20263762238323	-1.90444405149194	1.68152470761626
N	-3.75679355106514	0.40549618681566	1.86507837690917
C	4.38275474974234	-1.11006762962720	2.17145944630151
N	4.08444059495507	-2.25082547015818	1.57379049061264
N	3.74134490948371	0.06550750280943	1.86861608021385
C	-2.73857240776025	0.48412738264256	0.92053521394547
O	-2.26026733838981	1.57950604372441	0.62571448566239
H	-4.01615987513948	1.29808580090751	2.28133787099529
N	-5.39286535000126	-0.60058937590993	3.15787215996138
H	-5.91447919482541	-1.42408382855829	3.43004102940654
H	-5.60829564827883	0.28385390912313	3.60018150013241
C	2.71616987645098	0.23257212306949	0.94192911018261
O	2.28061540709993	1.35788791206640	0.70500795588089
H	4.04381861518925	0.92471346014723	2.32373880323652
N	5.33936160339313	-1.06857082012058	3.09856621841503
H	5.58940888997410	-0.21696794533473	3.58449057705365
H	5.82581258006010	-1.92531380298931	3.33034735386680

Mechanism path with explicit solvation computed with the approximation PBE0-D3BJ along with Basis Set 1.

Reactants for TS1

Pt	-0.52344306850895	0.16934485874686	-0.50644769208337
Cl	0.48005843548850	-1.13451189852723	-2.15627022167416
N	-1.41488884606626	1.26445611060644	0.96406982309884
N	-1.11506937458548	-1.55610808801761	0.39698997844271
H	-0.49183863423551	-1.65986645105103	1.22260091655952
H	-0.97029974581224	-2.36165249606681	-0.21544169786235
H	-2.08837204401178	-1.56999390485046	0.70628482001506
H	-1.11147054801443	0.98725193955633	1.90076092688138
H	-2.43515532522147	1.19650315893535	0.94526963757976
H	-1.17623032432496	2.25334871551958	0.84798816775086
Cl	0.26859939974417	2.18641287988923	-1.36097827946055
O	1.01699838576631	-1.38089830747620	2.28083229093843
H	1.63082888318562	-1.73267081701312	1.60740821883700
H	1.19777731098987	-0.42573411603278	2.17317891634939
O	2.67860639346280	-1.46202776367406	0.06996562133478
H	2.10984895449041	-1.45621092680019	-0.72691277666907
H	3.51936449480079	-1.84321330741978	-0.21319362582237
O	2.20280644123055	0.98692183210836	1.27593559044948

H	1.67351133039447	1.48014553449258	0.62903383918006
H	2.52633788122804	0.22620304707454	0.75082554615544

TS1

Pt	-0.97426599674939	-0.14503641646747	-0.23645031440226
Cl	0.32534418452078	-1.28026707916524	-1.81147720913068
N	-2.11615052301685	0.84186804204183	1.12362931750043
N	-2.53546598157901	-1.27414707245581	-0.83878534220171
H	-2.91331286922227	-1.85265511131875	-0.08460729423590
H	-2.24007750239966	-1.90735662070521	-1.58696080290049
H	-3.30486623933190	-0.70976406881438	-1.20712960805787
H	-1.92611968337439	0.51468648738734	2.07396182131097
H	-3.12647833490800	0.78291421591696	0.98130377978137
H	-1.85438482821587	1.83204056214270	1.08123085891807
Cl	0.17267898725453	2.33394790654620	-0.50284901908517
O	0.81004433848028	0.20466212170113	1.42614043791572
H	1.58095904104515	-0.30140334234024	1.08152282923840
H	0.94971538477361	1.10471045980667	1.07058717780804
O	2.76408562765886	-1.03798524121971	0.06307324914626
H	2.15632791219957	-1.35928630704357	-0.63391097225911
H	3.31594621544178	-1.79333439920809	0.30522105357817
O	3.29331244371400	1.56528935960066	-0.88870698097793
H	2.36188004611241	1.83957187958531	-0.82848540955985
H	3.26082777759637	0.64154462400968	-0.58730757238646

Products for TS1

Pt	-0.60647567567553	-0.16606683801196	-0.10857189357324
Cl	-1.37600571146286	2.03157006635166	-0.16549124280107
N	0.06517714246917	-2.09235091673657	-0.03459046692677
N	-2.17086610070374	-0.62828713471627	1.07426817592836
H	-2.79639015718561	-1.31665935778299	0.64700396971054
H	-2.73153754501709	0.20741536005442	1.26295660487169
H	-1.88722043391612	-1.00526131568167	1.98265389691676
H	-0.31451503767569	-2.65808787114945	-0.79764986133909
H	-0.17358890637081	-2.57508016039702	0.83436518057612
H	1.10119289932754	-2.11078509660662	-0.11898440542971
Cl	3.14456702810302	-1.45802832502450	-0.44066952849200
O	0.95584132535051	0.29772769985345	-1.36746418422608
H	1.26907819525239	1.22299796596725	-1.16267353953737
H	1.75074242052643	-0.28081564837685	-1.15545688407402
O	1.61820021910656	2.66709584221366	-0.45307762956185
H	0.68419547364814	2.91463035478808	-0.30770999357986
H	1.99683655431475	3.36172569040450	-1.00895486553836

O	2.38743998306106	0.84509285212460	1.64088999752745
H	2.64757493693556	0.10105770463575	1.06376553920508
H	2.21377138991229	1.55482512809054	1.00111713034341

Reactants for TS2

Pt	0.50216605207267	-0.27350268029765	-0.16305150510027
N	0.96101354526715	1.55900758523315	-0.85762042866555
N	1.09358702462051	-1.10633602591511	-1.93142336647566
H	0.52323259701125	-0.76694211211220	-2.71098218513418
H	1.03245180839596	-2.12717805315028	-1.95605150724108
H	2.06583778563757	-0.87561117816891	-2.15429404605198
H	0.06544235125022	2.08790789280614	-0.88225873470544
H	1.37198698582075	1.58039929270031	-1.79307384014537
H	1.60394255125509	2.05467373846343	-0.23571403014100
Cl	-0.17831990439766	0.70949315949272	1.83904305636568
O	-1.72293866668464	2.39154773339571	-0.46176069618526
H	-2.14496223538946	1.56612329621868	-0.77601862550125
H	-1.50883707035459	2.16447961226744	0.45814122183609
O	-2.92130572775735	-0.05705329270704	-1.13826262628965
H	-2.42104187790609	-0.47749653486638	-1.85322772695206
H	-3.82103340149063	0.03203595648791	-1.48391921197988
O	-2.45924100507185	-1.45819636558048	1.16612980241946
H	-2.18652931488935	-0.70718240853588	1.72030877934260
H	-2.73399006417490	-1.01836830659661	0.32809093782363
O	-0.06891154042232	-2.10130540051008	0.61510757761954
H	-1.05799806669959	-1.97706424427999	0.86040232366113
H	-0.03486182609267	-2.84228166434487	-0.00712516849944

TS2

Pt	0.98690706810052	0.02994024602664	-0.22818721854098
N	1.96956322511860	1.76566132749152	-0.46847654573436
N	2.38687532757010	-0.98624323295515	-1.28593352598071
H	2.62938953196977	-0.51392277582257	-2.16073928501996
H	2.06658365087527	-1.92304621372673	-1.54489330212995
H	3.25562893945466	-1.10781254830539	-0.75895545296664
H	1.48675746966272	2.37317030856769	-1.13624404219751
H	2.93770529386032	1.68216521298178	-0.78653727287647
H	1.99116149007103	2.26047990054439	0.42854517604889
Cl	0.37008170067687	0.90247057618010	2.29327003606856
O	-0.95313025762834	1.48007352184834	-0.25557435851114
H	-1.79070558622036	0.97079150111248	-0.31861772486094
H	-0.85941242779852	1.65311209411719	0.70293376348722
O	-3.25209218409272	-0.01148005875847	-0.09139362689304

H	-3.49273357065054	-0.59488125392623	-0.82585327595225
H	-4.05579384458018	0.49393885037239	0.09778833553304
O	-1.74705207461606	-1.29102517470948	1.78693918149490
H	-1.22981357127707	-0.55615200072895	2.18502827362012
H	-2.43460779181499	-0.83634187339419	1.25193159913049
O	-0.03695803222485	-1.73998833763320	0.01307509416186
H	-0.77794606952101	-1.62544909183689	0.73409498826653
H	0.54959171306479	-2.42546097744526	0.36779918385233

Products for TS2

Pt	-0.84828840465752	0.02333144421284	-0.00703091491953
N	-1.90821829358917	1.25782857803871	-1.18843966152187
N	-2.48711067962729	-1.04605754326075	0.49098253183972
H	-3.26429921283011	-0.45230758093336	0.79284164016098
H	-2.29654968081315	-1.68929432524767	1.26429904697436
H	-2.83417352746272	-1.61361653022347	-0.28736303515720
H	-1.27938651389542	1.94973486317086	-1.60678073600928
H	-2.63238301016813	1.77686361022907	-0.68420713627714
H	-2.37121126061474	0.76919426062264	-1.95954584904948
Cl	2.81561195828543	-0.63911082921139	-1.53701378635485
O	0.75757949060421	1.16607931577333	-0.54985403122410
H	1.18889631752913	1.59639329183887	0.23568483355428
H	1.47939057991231	0.58595417446159	-0.97084269258129
O	2.02939229371096	2.04956088633603	1.60802859335433
H	1.48361717849794	2.32602531195926	2.35902454878780
H	2.67691125026496	2.76003692427672	1.49044025718314
O	2.61539746409567	-0.64111292848049	1.49594587461699
H	2.86856709660942	-0.70952610470985	0.53984283446194
H	2.60764403872513	0.32386356334476	1.66363245804787
O	0.20596569786114	-1.23479012240595	1.22698538527311
H	1.21199487554757	-0.98959718888630	1.38544559169239
H	0.22279434201438	-2.12885307090546	0.85146124714782

Reactants for TS3A

C	4.30215035277583	-1.02084880006945	0.01918759002280
N	3.94194496761888	-2.34143719858087	-0.00881946861225
C	2.58573282666506	-2.39262666126376	0.01491265044478
N	2.03556850253912	-1.19994485617381	0.04377795685097
C	3.09109381676658	-0.31520463737238	0.04349295994968
Pt	-1.89644819629728	-0.38524011368081	-0.13278764992507
Cl	-0.71945645617910	1.10642215434802	-1.48003042725932
N	-2.90668241490156	-1.68438188745355	1.05967580805724
N	-3.50774383110887	-0.12571747206617	-1.32804105312971

H	4.57197450267103	-3.13801547832378	-0.02875335676925
H	2.04096934011426	-3.33674088407792	0.01921308636761
H	-3.74726585180811	-0.97456466048491	-1.84741234518536
H	-3.33636529440959	0.60686179108903	-2.02224504225459
H	-4.34484892696700	0.14843904461417	-0.80718351691068
H	-3.26841375139432	-2.49665851808052	0.55363471282771
H	-3.70030543246726	-1.25159382891833	1.53908719493444
H	-2.27348636464833	-2.03686962869875	1.78275368596960
O	-0.32461916918128	-0.69018017108502	1.12330028369004
H	-0.13422918972473	0.16618562656979	1.64089864082805
H	0.53731841351099	-0.89514656053172	0.62871427256039
O	-1.68792506394061	2.97516791614259	0.94301001402072
H	-1.47411560243334	2.58697978608585	0.07476819907705
H	-2.58099750602719	2.64846499683812	1.11859671374358
O	0.17882019111209	1.54854391353061	2.28140718136998
H	-0.53176550962081	2.11988547850025	1.89695241108843
H	0.97012670909548	1.80601859382922	1.77156120689149
C	3.19240136780906	1.09379056777524	0.05941434470657
C	5.49006124305336	0.83579023579157	0.08883102676361
H	6.46013772512871	1.34604652892493	0.12144949301334
H	1.25646941912717	1.57811250493666	-0.37472554650031
H	2.31722855062438	2.89507911802015	-0.09658198808969
N	2.11096666616092	1.91528841068711	0.07612944982382
N	5.52385925318175	-0.49088337627900	0.03745787726661
N	4.41684471315472	1.63177806545745	0.09630363436770

TS3A

C	3.27954459740647	-1.33301783463777	-0.34117475493495
N	2.87484110879153	-1.94656825753408	0.81388818322653
C	1.68105547505380	-1.40307680742397	1.16775006328644
N	1.28315495918771	-0.47380753210501	0.32741322340004
C	2.27115165459914	-0.40508466778127	-0.63054957398734
Pt	-1.05313572020608	0.24283749860100	-0.02821273025952
Cl	-0.53207658632200	2.50527993347322	0.19779600475751
N	-1.50649458537183	-1.73048362875178	-0.21016436559175
N	-2.31863441219701	0.73086909716338	-1.52028363896820
H	3.36985253299113	-2.68010934633392	1.31195341536258
H	1.12469407644871	-1.71714410901191	2.04877194395130
H	-2.09961299983725	0.25640053218623	-2.39965495646700
H	-2.26252843969551	1.73703966604099	-1.70165741041155
H	-3.29397062162286	0.52129432984508	-1.29400321710133
H	-2.32459929970522	-1.93511870379824	-0.78792479469871
H	-1.68671739134037	-2.13615407669856	0.71212883130642
H	-0.72210243983379	-2.24909410790568	-0.61358640370277

O	-0.71614499462830	-0.20504419602766	2.28022809653510
H	-1.63993035321417	-0.01701268560071	2.61975949286747
H	-0.16383451336350	0.50386818013897	2.63554861980040
O	-3.43293394480289	2.57741264835071	1.51671994175059
H	-2.57169031695524	2.64180730602254	1.06225349306039
H	-4.06273860672293	2.47160094603708	0.79065864731893
O	-3.20700906891155	0.27337477652591	2.87277659443736
H	-3.35058848529521	1.13617998897808	2.40817742851586
H	-3.41750178441143	0.45146215148652	3.79785269750808
C	2.46155514837738	0.38358254907167	-1.79272955460527
C	4.43586913638380	-0.77759860142368	-2.13372410508011
H	5.31067711079793	-0.90898023567272	-2.78277565217433
H	0.87265228750278	1.65904194390771	-1.56420462660471
H	1.85512665466715	1.90533931974002	-2.98341666934266
N	1.59704702666293	1.32513439622478	-2.19458614813841
N	4.38201456960147	-1.55632470772009	-1.05861333841512
N	3.56300822596526	0.15209423463317	-2.52641473660130

Products for TS3A

C	-3.06001435025862	1.69795092856154	-0.11668669207785
N	-2.18163650735494	2.66075788301855	-0.54564543474553
C	-0.94536989764784	2.12292947875244	-0.57835405934785
N	-0.96925192146124	0.85764590440547	-0.19367796386472
C	-2.28307872201531	0.56023856759416	0.10716937961591
Pt	0.62700054495766	-0.36930260140069	-0.10488549929781
Cl	1.22149099298194	0.54683972673074	1.94986649916010
N	0.10853651443091	-1.14311973320849	-1.92175479103509
N	2.27808807914754	-1.56217244927372	-0.06195642103083
H	-2.41378391475225	3.61783776235095	-0.79691311694642
H	-0.03355266672267	2.65416689590004	-0.86075619341948
H	2.17531612723695	-2.45636200969698	-0.54496706836116
H	2.57513730394474	-1.76934849461626	0.89395614243346
H	3.04367483883862	-1.02847060886259	-0.51277395401385
H	-0.63990785365065	-0.60453507658654	-2.36432090905772
H	-0.22560333899021	-2.10791461784050	-1.85108611795219
H	0.89338172793990	-1.14639159232857	-2.57796245264544
O	2.15590704066490	2.61126758014578	-0.56639855577960
H	2.79145313562091	1.92778162235598	-0.86290427619916
H	1.92887579443984	2.29411618998496	0.32084772941590
O	4.41732011134146	0.69192802285560	1.67195630309611
H	3.47111290177003	0.67323799913734	1.91110408721647
H	4.75342582902264	-0.15028599617382	2.00742724811858
O	3.97347961558335	0.53238820464932	-0.95047265534750
H	4.29095602312483	0.62349878798364	-0.01424072596244

H	4.76593870176628	0.57982426367713	-1.49970375243334
C	-2.95478039429624	-0.59287535048618	0.58366164659228
C	-4.90329799035858	0.65422839406868	0.49389059579918
H	-5.98760234978969	0.65495085440934	0.65879576721081
H	-1.32277059252405	-1.81435301723136	0.77159792631334
H	-2.86462971910620	-2.52381742986323	1.21926547235196
N	-2.33299511122926	-1.74251989065893	0.85600575300922
N	-4.37664758882853	1.79509232186886	0.06020437785616
N	-4.28197736382629	-0.49618152022264	0.75883871132849

Reactants for TS4A

Pt	2.420662000	-0.053310000	-0.146565000
N	3.718084000	1.049501000	-1.233287000
N	3.473793000	-1.761668000	-0.507518000
H	3.394287000	-2.071329000	-1.480588000
H	3.118678000	-2.523629000	0.077282000
H	4.473057000	-1.673067000	-0.303368000
H	3.785082000	0.728138000	-2.204455000
H	4.666622000	1.032092000	-0.846929000
H	3.426622000	2.032333000	-1.262774000
Cl	1.235324000	1.905903000	0.264812000
O	-0.811461000	-1.769673000	-0.634365000
H	-1.283784000	-0.882451000	-0.555674000
H	-0.597966000	-1.873323000	-1.573785000
O	1.151277000	-1.246177000	0.938263000
H	0.436981000	-1.566841000	0.295871000
H	0.610019000	-0.712309000	1.602350000
N	-2.374702000	0.346491000	-0.223282000
C	-3.690694000	-0.033824000	-0.079658000
C	-2.394838000	1.665047000	-0.233169000
C	-4.509296000	1.098785000	0.005811000
H	-1.514658000	2.302746000	-0.330309000
H	-3.904660000	3.151393000	-0.067719000
N	-3.651252000	2.165743000	-0.095644000
O	-0.411225000	0.293394000	2.404996000
H	-1.307272000	0.139844000	1.903861000
H	-0.022650000	1.097357000	1.961081000
C	-4.337387000	-1.296639000	-0.023163000
C	-6.328751000	-0.133669000	0.200425000
H	-7.416619000	-0.213936000	0.319657000
H	-4.202877000	-3.323010000	-0.092413000
H	-2.668994000	-2.471552000	-0.256621000
N	-3.678975000	-2.457401000	-0.108557000
N	-5.674216000	-1.296780000	0.121492000

N	-5.836550000	1.103120000	0.150141000

TS4A

Pt	1.57114252476305	-0.10341881354097	-0.09440609783861
N	1.93533260368608	1.04916734799424	-1.70958895138387
N	2.58285826641025	-1.67801120487039	-0.90793218115381
H	2.35688121527674	-1.85071266353594	-1.89057660163676
H	2.36922499496980	-2.53972938262207	-0.39872054138776
H	3.59509582155027	-1.54467733553810	-0.85149177764748
H	1.17459044379762	0.99116800161175	-2.39137210417038
H	2.79928453640070	0.81939568224881	-2.20569921408783
H	2.00977485934523	2.02991250649030	-1.42711882935504
Cl	1.78417858238599	1.95371411195022	1.51414482919891
O	-0.36918799151823	0.18826219704368	2.93031365376214
H	0.14727387278901	0.96000587279186	2.61541234051769
H	-1.24731836035184	0.31790216147725	2.54703013903575
O	1.23694911071636	-1.29094426497982	1.55046461308643
H	0.49595589405834	-0.85366462735570	2.07492283514741
H	2.04390824424587	-1.11852207372580	2.12960567501064
N	-0.71746890888755	0.86363693042071	-0.20542236853252
C	-1.82523928079362	0.23132353892837	-0.72174886282121
C	-1.11873505323127	2.08109935979784	0.08456672201338
C	-2.91678989971798	1.10590896479422	-0.72751171976417
H	-0.48444405253644	2.84905009792996	0.52589225662626
H	-2.95324983494819	3.13537984053127	-0.06240534941526
N	-2.43098833076700	2.27670592499634	-0.20830343729732
O	3.28657631753636	-0.42840220302765	2.87350900033719
H	3.23890106847512	-0.49302903510508	3.83678587390562
H	2.99410025500003	0.48398067305977	2.66845608201419
C	-2.06039432222625	-1.07180181950143	-1.22426907887839
C	-4.24944242922404	-0.42316633681631	-1.59424876109982
H	-5.23738178321920	-0.72904806979448	-1.96055367371898
H	-1.30180050774377	-2.91136231913236	-1.67927809134738
H	-0.15565779161846	-1.76289510330687	-1.00537766159759
N	-1.10342430210287	-2.00336724185614	-1.27934354085906
N	-3.29977077546306	-1.36015734783558	-1.65267776335110
N	-4.15073498705703	0.82629663047810	-1.15305741331125

Products for TS4A

Pt	0.54946289843921	-1.00383334190846	0.22158168836574
N	-0.24871239463857	-2.50176502843266	-0.87478709951794
N	2.01623382518178	-2.24166995714594	0.92527407333884
H	1.82674267416196	-2.56026591370641	1.87871362207767

H	2.90488534112312	-1.72029698709616	0.94055360742778
H	2.16916362301284	-3.08004127971340	0.36044969060886
H	-1.09115783549964	-2.21049635006703	-1.37707224165992
H	-0.51328741097776	-3.30737724362485	-0.30123351344297
H	0.40683541874602	-2.84409436356683	-1.58272905399244
Cl	2.73423231345335	1.27358065230904	-1.96187194705027
O	1.26530297716684	2.77499445866108	0.23399634077346
H	1.72643882236948	2.44770419494855	-0.58005365698294
H	0.45308635297995	3.19478797178373	-0.07802405995692
O	1.35941699209844	0.48320783535442	1.37545619393231
H	1.16813809257290	1.39887140311281	0.98655008414715
H	2.35185734515177	0.39754173139391	1.25385933166074
N	-0.84535451647833	0.26129224132165	-0.49180353506799
C	-2.10603564317949	0.50336658373057	0.01820287391287
C	-0.70781725611561	1.04305872962551	-1.55140567657879
C	-2.72966374571237	1.46685167220304	-0.77485203508403
H	0.20495094309284	1.11249364274115	-2.14962506066583
H	-1.94484345675567	2.45739267033524	-2.49984507148858
N	-1.81667805204037	1.78230287134578	-1.75070010898279
O	3.79191607815566	0.02486733539603	0.60290389081723
H	4.55098615231911	0.48966407686382	0.97892050351530
H	3.60935937041963	0.45958472401394	-0.27223391763222
C	-2.84155754850480	0.00402368629409	1.12152930939728
C	-4.55358835239390	1.41593563148566	0.46183798526220
H	-5.56993561817639	1.76912004187598	0.67424935666330
H	-2.93315718795412	-1.22904704276150	2.73880750823441
H	-1.42281519003298	-1.28140331037568	1.84675333289314
N	-2.36286958184323	-0.91392495804175	1.96387197195192
N	-4.07710201193159	0.49624757979086	1.30260385397628
N	-3.95466341821003	1.95700604185382	-0.59440824085286

Reactants for TS5A

C	2.84674040578920	-0.23804111214708	-0.02222211721480
N	1.81189967879380	-1.01499271600664	0.45126837829432
C	2.32769755489534	-2.20729777658173	0.66353719986446
Pt	-1.89056497604862	-0.33080511148618	-0.13244748730109
N	-2.66978270483739	-2.00552409735502	0.67745984159270
N	-3.30405093486083	-0.33752576323299	-1.57044584652484
H	-3.25439826507953	-1.17435453287416	-2.15921809738530
H	-3.23566781595470	0.46305977057770	-2.20450085796521
H	-4.24868145126309	-0.30651602542793	-1.17532489577380
H	-3.42183187285801	-2.42977257854535	0.12809508341894
H	-3.06196989116168	-1.82710876961940	1.60707544940759
H	-1.95646030084204	-2.73216452628211	0.79019068697165

H	1.78174635410818	-3.06617917497714	1.05332582259070
H	4.24672959551268	-3.06837540753488	0.44156106244217
O	-0.43833254965060	-0.26124629036789	1.31514640872621
H	-0.63500890741549	-0.78983157380607	2.10389267565446
H	0.51361388797540	-0.60698330872413	0.93015151110417
O	-1.01611038299527	1.36013167752324	-0.88952469212393
H	-1.30704960242144	2.21220342237259	-0.33114185104078
H	-1.17486115088205	1.54399932522893	-1.82759865348429
O	0.41896232779059	2.37383829755716	2.10152601924934
H	0.13394626524152	1.44240150964071	2.05272604435838
H	1.13400796947254	2.40732623052884	1.43906142071668
O	-1.50690302139648	3.28710222083033	0.56560038870581
H	-0.82963674298789	3.04806208648792	1.26327005674105
H	-2.36777127162290	3.22597641152643	1.00219220794885
N	3.64278741760255	-2.25564655152930	0.35445574292145
C	4.01453061573339	-1.00922130539490	-0.07904486012125
C	2.96218888571746	1.10431071107904	-0.44767323005954
C	5.21276327935982	0.69690262629181	-0.79447120990706
H	6.17430024534206	1.12317905441424	-1.10419603940704
H	0.98280816961652	1.60024125002187	-0.62920332973802
H	2.10850254533131	2.85241537700703	-0.93965115485752
N	5.21780616032006	-0.58871929472568	-0.46059258809152
N	4.17483718964291	1.53726863433357	-0.81010113987395
N	1.92077329403267	1.97172731119717	-0.46914794983899

TS5A

C	1.98091088384400	-0.45888794445833	-0.37245661155179
N	0.77023302506333	-1.12870423749127	-0.35869113925562
C	1.08998538019358	-2.40527826334565	-0.36013070749279
Pt	-1.72861051159870	-0.54908055174573	-0.29427248760714
N	-2.21357542931811	-2.34776091880200	0.48265400284876
N	-3.36047851588971	-0.61951134610685	-1.46969071217702
H	-3.15084594030043	-1.05405680850529	-2.37304556927539
H	-3.72362963729166	0.31773875110446	-1.66487110180289
H	-4.13717856905638	-1.14517716187708	-1.06011623478877
H	-2.21430990607150	-3.08941358855405	-0.22412452748395
H	-3.14745645398121	-2.34456157780777	0.90342874407296
H	-1.57415413216421	-2.65163738304716	1.22227102959131
H	0.38515606279080	-3.23602620764219	-0.35802218122181
H	2.89376766925243	-3.51710270954307	-0.36345677394380
O	-0.64793171667522	-0.06000132000728	1.79770595564196
H	-1.20813470086237	-0.34301391432500	2.53748304926316
H	0.15824491968779	-0.59558735097843	1.87074861367192
O	-1.27579524030890	1.29533507651384	-1.06180913959793

H	-1.70993455869562	2.08945398328774	-0.49069023447477
H	-1.50060677762604	1.41824654974851	-1.99643707753182
O	-0.02395380082425	2.65864106018866	1.94598827832177
H	-0.22469617758720	1.71554266457215	2.10297485545000
H	0.67655016378322	2.59630537762023	1.26920203321897
O	-2.08635660932795	3.08862240551714	0.39363490862850
H	-1.34482219615215	3.03027790046049	1.06981961278203
H	-2.89445869950767	2.84531863414177	0.86641490240633
N	2.42896349061912	-2.61367548268248	-0.36423965274311
C	3.03416017863141	-1.38696098510723	-0.36584093504922
C	2.36540622915064	0.90200813613970	-0.43407665318817
C	4.56848674095036	0.19578879198478	-0.38252388716770
H	5.62076580474204	0.50439139112712	-0.37114826010084
H	0.53280083056356	1.71990524186238	-0.80994009996677
H	1.87023750663244	2.81822229634364	-0.78022378814275
N	4.33685822597700	-1.11164556906080	-0.36594545027974
N	3.67427052543176	1.18493593280594	-0.42409518247917
N	1.48225193592589	1.92583912766911	-0.48957757857469

Products for TS5A

C	1.85143284702354	-0.23832179930377	-0.12020542713446
N	0.76295042191687	-0.82568045178437	0.49971188927820
C	1.24191630409812	-1.53359243547617	1.50868922165901
Pt	-1.17481985055983	-0.58093323078060	0.00192703362498
N	-1.40978103310098	0.71764714669019	1.50554623019186
N	-3.11834729610869	-0.25269886456160	-0.51914708247335
H	-3.77428836908818	-0.68081695405406	0.13904534433628
H	-3.36134527202533	-0.60683078113033	-1.44674627535193
H	-3.28919634022800	0.76869147810394	-0.51428166545106
H	-1.81006547167975	0.26276040083499	2.32950078803017
H	-2.05052880011722	1.47335320296302	1.19769343351094
H	-0.52253788674192	1.17065732191692	1.78258451734185
H	0.64214254914027	-2.11814057111053	2.20406603683404
H	3.17113984206443	-1.89790024555039	2.27099473740948
O	0.87625144015192	2.40446156744542	1.87140494583597
H	0.87096615076443	2.97135904260329	2.65570430279387
H	1.80347633481343	2.15104842468288	1.76035967986872
O	-0.89697300436437	-1.96913012280728	-1.53127103012170
H	-1.28684059967711	-1.72580219231630	-2.38739615756006
H	-1.27978822079367	-2.83357546396446	-1.30681219735084
O	-0.33449225391286	3.30682457424909	-0.44993908072411
H	0.12014231044586	3.11728406475030	0.39741587092426
H	0.12561546027869	2.71816149693688	-1.07293878606898
O	-2.85372007220865	2.53341435464962	-0.07941362397135

H	-1.95451403367721	2.90553680570535	-0.28905242190931
H	-3.37440780680562	3.27132718878596	0.26010097538784
N	2.58321255121067	-1.44276855061913	1.57715982471561
C	3.00997293812112	-0.62688247132041	0.56148678131708
C	2.03183470870578	0.61681927887333	-1.23277020168956
C	4.30116614113795	0.54351094371578	-0.78789913323415
H	5.29603904720056	0.88878392051524	-1.09242197825734
H	0.10936242391628	0.65007950232306	-1.92925196183205
H	1.26852774627763	1.51885055974777	-2.87376799659042
N	4.25353939594962	-0.26289684851280	0.26639014495787
N	3.28252184301651	0.98445187263297	-1.52890247927631
N	1.01744785485571	1.10353583516616	-1.98183825902106

Reactants for TS6A

N	-1.78408854142992	-2.86176938144913	-1.39111456234079
C	-0.80383988283009	-2.01099495066983	-1.02250942845108
N	-1.29476650106406	-0.99144714539042	-0.33746433404041
C	-2.65891443853208	-1.18896729920648	-0.25904513804915
C	-2.97826566736650	-2.37250942670314	-0.92578838834824
Pt	-0.26377947911913	0.60784929265218	0.33328374537828
Cl	-0.90025455625003	1.75307074370768	-1.58577938543632
N	0.81835366971846	2.20412505774125	1.00050609225210
N	0.23368396562059	-0.44043059598541	2.01757099450421
N	2.43459041859560	-2.38756656971652	-0.83930269405188
C	2.59869502055132	-3.32161480081699	0.06813229127225
N	3.19333450035609	-2.86060405262151	1.20696688635861
C	3.42832703498648	-1.52701581539786	1.02666046792389
C	2.95097156429878	-1.25335894687225	-0.25968146028036
H	3.41381143204564	-3.40604260863032	2.03473869057913
H	2.29851384632349	-4.36480866091189	-0.03447684488784
H	1.09575096404832	-0.12414076034014	2.46828756092454
H	0.35409808427474	-1.43694361403771	1.81905846870485
H	-0.50612007731785	-0.37264827742637	2.72153364442541
H	1.81975654565888	2.00582052160738	0.87759002469846
H	0.67024247738554	2.41329376554096	1.98988155304245
H	0.64386535941177	3.07203727737002	0.46872538913828
H	0.25523498533191	-2.16345872692257	-1.24900234681891
H	-1.64867611070000	-3.71980826156742	-1.91862049373992
O	3.26012166967901	3.24517236806584	-1.62555072103285
H	3.51264202022454	2.74088534030672	-0.82780807986222
H	2.42726207208997	3.69070996455285	-1.37207693218697
O	0.77247021084467	4.28504277076673	-0.95127161727632
H	0.42091565441417	5.18478732866306	-0.93423912020469
H	0.10168204354533	3.74398450917062	-1.41018036641080

C	4.00549399418578	0.57527483896488	1.37208244544216
H	4.42036371133308	1.36149890338796	2.01374483562527
C	3.05686951315128	0.08778798440093	-0.70005449416183
N	3.96690520731300	-0.64738115349967	1.88041347002482
N	3.58332286025900	0.98295844005076	0.16773039920907
N	2.66514522034002	0.51042133451942	-1.90040306204827
H	2.74352239633431	1.51677176910801	-2.09811137056409
H	2.21579101747586	-0.12570415227843	-2.54427294465184
C	-3.73094076377534	-0.47788022256991	0.33481066405314
C	-5.12400218699231	-2.13948825415373	-0.47664375337916
H	-6.15714410636948	-2.50048047044614	-0.55090478406213
N	-3.56611173798087	0.65633399530550	1.01839319472319
N	-4.19759337343655	-2.89138915183642	-1.06266027666634
N	-4.96063015638479	-0.99826618116858	0.19433421691249
H	-2.64262715541630	1.07098038023979	1.11156040021874
H	-4.37270272483248	1.12713289449635	1.40903716354103

TS6A

N	-1.70928266387682	2.68575860853322	-0.79070776190160
C	-0.93250684822633	1.62882365323924	-1.09752371285966
N	-1.32173796947640	0.54234475554364	-0.45337548539791
C	-2.41342132975620	0.91073489348238	0.31203428227535
C	-2.66770003322824	2.26827727263130	0.09572151762085
Pt	-0.44198958861175	-1.26524229624577	-0.61908090024050
Cl	0.03610829769083	-1.44886109149999	1.96918855945601
N	0.45547220543447	-3.08628065713629	-0.82454072211705
N	-1.80414566373073	-1.72096820811929	-2.07353958429901
N	1.74921129457523	-0.15846281607526	-0.52090348638532
C	2.60932133603269	-0.46847287670061	-1.46420302023931
N	3.56327968113816	0.48276580750664	-1.63363068712692
C	3.30743782478599	1.48206849167073	-0.73503163823323
C	2.16817975037564	1.06304724679690	-0.03492033284959
H	4.32092305982798	0.45893909574798	-2.30941756466645
H	2.58057964618395	-1.37260326423082	-2.07130085591588
H	-1.61483604535153	-2.60454978736513	-2.55087825440863
H	-1.83584115572755	-0.99908671763476	-2.79735201668078
H	-2.74682631990568	-1.79399338268157	-1.68366480473265
H	0.72036337618428	-3.29542231971224	-1.78976023647122
H	-0.14912820961540	-3.85010851768265	-0.51454368322426
H	1.31618502339495	-3.12978038851935	-0.25026798411739
H	-0.09461956353353	1.67957875860807	-1.78969229996077
H	-1.60284945270370	3.62425190436977	-1.16581260697130
O	4.68255668271416	-0.88093139929165	1.05091164181405
H	5.00204899989216	-0.88115513024108	0.14181086507049

H	3.99021883263437	-1.57042786061878	1.03814523006289
O	2.64037035056083	-2.78367824295621	1.04609523534609
H	2.80180444628684	-3.54879525771415	1.61505063537262
H	1.92189583650714	-2.28894781601080	1.50134691173152
C	3.43523656227258	3.36836407722737	0.39615747441645
H	3.92350268393276	4.33088263109109	0.59374160381500
C	1.70823180750588	1.93813720980290	0.98249059470004
N	3.97956756457194	2.61906299116812	-0.55659518019015
N	2.37121090782767	3.09582649754478	1.15141553966383
N	0.64754462562531	1.68015008826661	1.75930308864852
H	0.46866275437897	2.32087280071839	2.52344574561038
H	0.31333569505487	0.71651090586648	1.84693745374161
C	-3.29359962136214	0.23323265072507	1.19431236027242
C	-4.43122022149381	2.24020930876230	1.41687599103781
H	-5.27956999801650	2.75452054658557	1.88447366950607
N	-3.18473421950157	-1.06600406387092	1.49988302541039
N	-3.65825148333131	2.98011527466322	0.62908377747241
N	-4.30376310201607	0.94735059303590	1.71617488179642
H	-2.27867035543439	-1.52015639538810	1.38589927404516
H	-3.79597540049010	-1.41919757389235	2.22740346010319

Products for TS6A

N	-3.23839515351304	0.74694475663533	-1.24240708707214
C	-1.93271340050945	0.76491041385950	-1.56880258603569
N	-1.22649738096281	-0.01754559604578	-0.77070783239786
C	-2.12237152229013	-0.57655430827869	0.12805869755018
C	-3.40187257157520	-0.09646536803617	-0.17461365020296
Pt	0.77647787418170	-0.28653557560734	-0.86496862780464
Cl	2.27015462893295	-1.42447656999444	2.17726723843333
N	2.79545642122900	-0.55096871908479	-0.85531645563507
N	0.47945618631194	-2.26260016633325	-1.30183884644924
N	1.03152790341228	1.70194402509956	-0.57208550355179
C	1.36076086098868	2.53311197811917	-1.54916454734170
N	1.21938570429992	3.81692297591215	-1.17107784801033
C	0.75689103138143	3.83401967666226	0.11931053622390
C	0.66014838447000	2.49252188187596	0.50310977850800
H	1.40660628411984	4.62914518673357	-1.75297747787279
H	1.68676427781042	2.23243606907636	-2.54345914757767
H	1.02700896524301	-2.90783610463485	-0.69810770185953
H	0.76102734559966	-2.45074105472726	-2.26707842585090
H	-0.50011732132839	-2.54579908496048	-1.23428075571993
H	3.13936965519404	-1.39188592009906	-1.36069117625820
H	3.02367957601289	-0.69298128127200	0.14252496103925
H	3.32027831258487	0.25800507090045	-1.19187221544555

H	-1.52723373690367	1.35321453488319	-2.38995389882838
H	-3.97319085860883	1.26687489695540	-1.71449004794858
O	3.70142324394555	-3.05546568553518	-1.83918881976613
H	3.23212957979860	-3.39979804575802	-2.60998509277198
H	3.27945073395977	-3.51158624956665	-1.08044323338558
O	2.21783234346455	-3.81358172239966	0.31319629851865
H	1.91884932572061	-4.62818933251716	0.73702692947435
H	2.28315552773266	-3.13713078106935	1.03370150212893
C	-0.01542957090368	4.54156987260532	2.05641214094617
H	-0.33270940878931	5.35932422597622	2.71474802523516
C	0.24353699363886	2.24681848348333	1.83717879857764
N	0.43891214124032	4.89404784301013	0.85824526594212
N	-0.12008321698919	3.31546306023555	2.56618432356180
N	0.18227900452626	1.03207676475557	2.39424605254898
H	-0.04288474131643	1.00817665290551	3.38294236834868
H	0.77565108012067	0.26679313185681	2.05683640956802
C	-2.03005580434521	-1.48807010123613	1.21474932280067
C	-4.33181947501232	-1.27185132215579	1.43734185117842
H	-5.22020914208816	-1.58395089905280	1.99969016303387
N	-0.90549515572489	-2.07209602155417	1.64198076175876
N	-4.53527987200639	-0.40811020500763	0.44834251397326
N	-3.17954449498130	-1.80624387313587	1.83566185156220
H	0.03447096988993	-1.73978423538224	1.41887382696029
H	-0.98469852796210	-2.64696127809654	2.47324935991401

Reactants for TS7A

N	-3.00683127609063	-2.90069037905020	-0.20036605620366
C	-1.88742503768431	-2.26095214504357	0.18474428160880
N	-2.01625157231259	-0.95001947347702	0.05605811012127
C	-3.28881344663023	-0.72466301191871	-0.44380186897111
C	-3.92412554566053	-1.96162852701967	-0.59644112223829
Pt	-0.61268093702628	0.37950380501584	0.60926864496295
O	0.46493402152338	-0.02602238545599	-1.05409606257997
N	0.86122932975842	1.69358320204274	1.15888726480978
N	-1.68515113077238	0.81942436748800	2.28309111164405
N	2.71166866298797	-1.05714918552428	-0.38837361756848
C	2.76093386499961	-2.28656893730589	0.09026260148935
N	4.02008541526553	-2.61278058351017	0.40717848379635
C	4.83530589353502	-1.54030893997331	0.12327046927376
C	3.99371655405576	-0.54723862692689	-0.38346969841704
H	4.31640031615495	-3.50470491933401	0.79681244538329
H	1.90103496297817	-2.94409682245730	0.21134860550190
H	-1.10563623188948	1.01152591074636	3.10388604012656
H	-2.31081788737866	0.05522984873725	2.54999241416100

H	-2.27715164085292	1.64307038036359	2.14327887023451
H	1.66766285871678	1.20447257524295	1.55620670737460
H	0.57043029243363	2.38697798447573	1.85150013604329
H	1.20555195266385	2.21849777568395	0.34764655470778
H	-0.00522666520036	-0.64641076209022	-1.62837317279542
H	1.76809124413321	-0.58771522918975	-0.71247419928479
H	-1.00062145420891	-2.76938387215018	0.55882631755084
H	-3.14567692580028	-3.90754635534261	-0.18866511842855
O	-1.33272971322575	3.36277297426373	-0.85502694333750
H	-0.44461333684659	3.07180568536242	-1.14680330925138
H	-1.16933767980790	3.76714592553194	0.00656572155043
O	1.13647808717265	2.37462789778453	-1.73441894207526
H	1.22203085414399	2.62912235781632	-2.66230151809658
H	0.78584592505463	1.44159568023760	-1.72298389670251
C	6.61465018891716	-0.26450075243475	-0.08830798131761
H	7.69565521175983	-0.11181617272278	0.01513869323979
C	4.59926462277950	0.68644056198531	-0.74586909449367
N	6.15035068654338	-1.45190682619886	0.29115105047233
N	5.93176466682317	0.76731552010604	-0.57750741343421
N	3.95173417718151	1.74162274956836	-1.23356178404685
H	2.93807248735041	1.79938987663688	-1.35199980826054
H	4.49843069228670	2.57123442245378	-1.43343209380629
C	-4.03885728879443	0.42503485067129	-0.81333173394522
C	-5.78489983442329	-1.02722697865649	-1.30824933275133
H	-6.82204310127896	-1.11064834997146	-1.65552366010701
N	-5.16578040590835	-2.16963750050170	-1.02736598064097
N	-5.30274530142804	0.20999419161616	-1.22741846378509
N	-3.59739643059149	1.68172764744817	-0.77189356343100
H	-2.62054879297705	1.97303689633856	-0.65212733649799
H	-4.23601133242978	2.39013364863829	-1.11557075158431

TS7A

N	3.34468137044727	0.06351866424412	1.45128935650196
C	2.40361503983581	0.95164080975693	1.08247664103256
N	1.50973062442924	0.39015306726544	0.28034038497039
C	1.90468040537737	-0.92494656590085	0.10218792163750
C	3.06297336460578	-1.13941737562026	0.85436314014452
Pt	-0.16333073544394	1.35130224986322	-0.30739489487238
O	-0.83173549206324	1.88058022975998	1.90928479010541
N	-1.80454994602554	2.46499363807724	-0.81606307149204
N	0.71559029789381	1.82593280014631	-2.05002843382458
N	-1.41777874699513	-0.65520564455541	0.76666740934718
C	-1.01540323826086	-1.24257608010024	1.87380515284083
N	-1.55033826371745	-2.47917776219658	2.03556631248101

C	-2.35489021701400	-2.72208523066021	0.95453293646181
C	-2.25698544148455	-1.56961113306243	0.16576323695428
H	-1.38082642390724	-3.10683916620697	2.81601240515761
H	-0.32314525355617	-0.81835295887867	2.60077213245258
H	0.34589236355199	1.27503513630781	-2.82980918532579
H	1.72371661912280	1.65414965854155	-2.01401865582490
H	0.59304512200146	2.81339278534643	-2.28935764936256
H	-2.68713028608196	2.07268749996577	-0.47895644280620
H	-1.92476599066881	2.64129766931449	-1.81617324456875
H	-1.68257976531897	3.38183251448446	-0.35160485009560
H	-0.17408779887866	1.73613502235166	2.60715783339654
H	-1.61847687911185	1.38669781890857	2.18926887612960
H	2.37836451884985	2.00952915438104	1.36850365265243
H	4.13688983584143	0.26045948580119	2.05694606972772
O	1.88053901011451	3.98322075587475	0.89238662746338
H	0.93573529988603	4.23796373692369	0.92549770612712
H	2.05134044082919	3.90643971462216	-0.05472976772390
O	-0.85662279658486	4.46398444188947	0.98459638963243
H	-1.23942715860324	5.24613682960687	1.40244383988720
H	-1.00019674609343	3.73075835411111	1.61231653134030
C	-3.70710117575061	-3.70767716246951	-0.48080998551676
H	-4.32983890851374	-4.56251606099595	-0.77181471850961
C	-2.94477416307576	-1.60548821014369	-1.07152970597877
N	-3.07133291401875	-3.81274157201632	0.68084032967856
N	-3.67567535136128	-2.69559440127008	-1.35011499173135
N	-2.88170542288556	-0.61724205212509	-1.97337497881282
H	-2.26382725408951	0.17008185237686	-1.83403661752840
H	-3.33087488782573	-0.73516943559325	-2.87276948912315
C	1.45208484595147	-2.01677127046933	-0.68254701455895
C	3.19470161362618	-3.24619861058135	0.22226490651315
H	3.68676273260675	-4.22528649631732	0.26633198626088
N	3.74103960585229	-2.28020154899315	0.95324881043659
N	2.12623785267524	-3.17252961953517	-0.57030427084604
N	0.42517569283390	-1.95049985566444	-1.53183449200537
H	-0.11533972581009	-1.10275013114312	-1.63874463306539
H	0.15696432680858	-2.77727554542170	-2.05101828576023

Products for TS7A

N	3.15691042628419	0.84714528455903	2.06156919570226
C	2.85300067355192	-0.16322429077971	1.22764896699126
N	1.94357461166842	0.20544640682718	0.33850953106310
C	1.64554561606302	1.53151651003063	0.60797377583217
C	2.40373659813002	1.93787519080120	1.70791168168086
Pt	1.09373435378704	-1.07325544953063	-0.97739586692651

O	-5.36095775162217	1.50316462264276	0.44445006912141
N	0.27257443095552	-2.42175446544723	-2.27596038237926
N	2.29954131103524	-0.37544725483026	-2.47086000648570
N	-0.05816332511087	-1.77689254443415	0.52972376134502
C	0.37292784340171	-2.60447320270375	1.46940209711528
N	-0.54237482928433	-2.75468402580331	2.44394768655182
C	-1.62934310640131	-1.97973857925070	2.13473568482941
C	-1.32570837250218	-1.37621904735674	0.91426668250527
H	-0.43351553082294	-3.33728794608015	3.27034885841204
H	1.34335138489460	-3.09780993588974	1.47538454863085
H	3.01153205286607	-1.06565167175275	-2.72603638755762
H	2.80146780775041	0.47626485330861	-2.20788300138437
H	1.78322866677164	-0.15598142904947	-3.32711504444320
H	-0.45680910202144	-2.98941864454341	-1.83745552126710
H	0.97615172953907	-3.07461847778362	-2.63187296069200
H	-0.15481448686136	-1.97669888380358	-3.09299814999741
H	-6.13493698816379	0.99580547316983	0.16576978395121
H	-4.69670790169608	0.80559088370515	0.69642090840388
H	3.30172825635422	-1.15282176258932	1.29326160688881
H	3.82658100831878	0.80082888177299	2.82518649019913
O	-2.03602519995491	3.96348528314936	-1.87622630276318
H	-2.77629686683895	3.31481756264793	-1.84737347222347
H	-1.92175836299052	4.14130838979138	-2.81858677326550
O	-3.97057901378436	2.06029710810478	-1.69692369755680
H	-4.50900860313085	1.85559220992150	-2.47272649857225
H	-4.58559042392585	1.96691675565828	-0.91204074190189
C	-3.57639058472487	-0.95969328051452	2.26325566391638
H	-4.50688840355436	-0.73342626067771	2.79677284375997
C	-2.32372288866496	-0.54373299001217	0.33942089481611
N	-2.74379838727637	-1.80785350364246	2.84609870060473
N	-3.42762222141685	-0.34227895851603	1.08712252297621
N	-2.22210897170566	0.00025170081523	-0.86706150337090
H	-1.39239420196662	-0.18708350731019	-1.41614098287928
H	-2.89969333318277	0.69335940681272	-1.22400189102406
C	0.78841446866550	2.50623796167901	0.02707747895138
C	1.50209354639404	3.94730245012633	1.69925882883934
H	1.40252285709156	4.94803940887710	2.13731147854260
N	2.37393636657861	3.13502499303789	2.28965559651926
N	0.73717096401779	3.70505363157094	0.63687697250186
N	0.06623559294292	2.30550884901886	-1.07117004645864
H	0.11048318951468	1.41037833732529	-1.53921490501500
H	-0.65294289897255	2.99189295694761	-1.36791417448746

Reactants for TS3G

C	-2.61940738056316	-1.10734041750120	-0.45593269076339
C	-2.46527393788441	0.29773619475926	-0.25195928721566
C	-3.52386262334563	1.00843948153931	0.32039093662953
N	-4.71724302253810	0.54314252555037	0.70943740127238
C	-4.86698334926602	-0.75317382970013	0.51319831742711
N	-3.88142194803301	-1.53566786290171	-0.03451370676524
N	-1.41485875720543	1.15220158279226	-0.50052552647635
C	-1.80989492721028	2.33585120894279	-0.09429659925782
N	-3.07747638033012	2.29582675307807	0.40557795561926
Pt	1.72995976553876	-0.73119292007496	-0.06561304948713
O	1.00273314585983	0.70556819437448	-1.32818622967834
N	-6.01442535294524	-1.35388224806448	0.84646221136221
O	-1.82768480559965	-1.92475410844070	-0.92207635291294
Cl	2.54595658846404	0.80200871190867	1.48598054289326
N	0.95934475391849	-2.05087608817615	-1.40822645005984
N	2.42027932725067	-2.20849726568763	1.12545054110432
O	2.19821633236533	2.98283403719172	-0.64852219570351
O	0.15769578990194	4.84298733923914	-0.24308368096651
H	-3.59795895997784	3.08412089301153	0.77838786025209
H	-1.21025504813313	3.25346714657909	-0.13431543843998
H	1.67030006496468	-2.81780884629483	1.46263746375028
H	2.88327669415264	-1.82336893945384	1.95323278244329
H	3.10318811631027	-2.80927772354607	0.65642096272506
H	-0.07401616870690	-2.03158958513653	-1.32212603941860
H	1.26796673374689	-3.01783821824636	-1.29403286658472
H	1.19700879740577	-1.77332618414706	-2.36294069103771
H	0.91506621695813	4.24153494872779	-0.38627686351996
H	-0.11165553990099	5.06898537911718	-1.14229134329283
H	2.48608492411963	2.55831714435979	0.18674713343723
H	3.01647099568557	3.22922718350050	-1.10330787411861
H	1.46512690878163	1.57449749267876	-1.20065196015786
H	-6.15154993635392	-2.35125056532276	0.75402130776807
H	-6.73948921261793	-0.79906584392520	1.28212473859169
H	-4.05368737394770	-2.53143037827073	-0.15614109476509
H	0.02327956913527	0.87731480753960	-1.04693021465368

TS3G

C	2.03114475571518	0.00094849064347	1.16708534631576
C	1.45618675807085	0.51889257032252	-0.02149263912399
C	2.27330189502237	1.23350672734381	-0.90283357963335
N	3.57871096643244	1.49186274280401	-0.77594492735578
C	4.11592371461785	0.99830191224464	0.32481897304173
N	3.38605288326791	0.29121637028322	1.25209723487788
N	0.16864564492423	0.47344649337307	-0.49969565108577

C	0.19538687467139	1.14197522513353	-1.62525911721177
N	1.44532933814785	1.60741041273765	-1.91766023367736
Pt	-1.68462380933303	-1.06778515076861	-0.00168120184326
O	-1.18605764979870	0.13886595747239	2.01013333181466
N	5.41668332707382	1.17718648522738	0.57351111476689
O	1.46979588231460	-0.63417722077901	2.07456522356560
Cl	-3.15293622656786	0.52780190272089	-0.86339078401609
N	-0.41402991945830	-2.47297240627763	0.75202449244755
N	-2.76718255492101	-2.50079568700395	-0.92507057750705
O	-1.71931970867710	2.59251074644401	0.99685887085993
O	0.81250495903099	3.65495866958154	0.54032136258767
H	1.70204714457298	2.16359066153584	-2.72755588963716
H	-0.66448099674274	1.31365777771308	-2.27151866791785
H	-3.57070888097499	-2.08086542076831	-1.40024813698644
H	-3.13624145744645	-3.20263858682217	-0.27912545509535
H	-2.22788948548354	-2.99832700107196	-1.63774617670832
H	0.33643082997275	-2.01323004586259	1.28989933390046
H	0.03410559086923	-3.03483909740652	0.02475738497450
H	-0.88366918143358	-3.12545360873958	1.38374342169824
H	-0.08665753236016	3.29376908218666	0.65528032091824
H	1.34203652016401	3.10215582793816	1.12608459297025
H	-2.22161720391501	2.15691510573627	0.27776603934582
H	-2.37766902593924	3.09897768936725	1.49210654007746
H	-1.26346053763986	1.08472303512065	1.75215472840001
H	5.86862647806116	0.82614980731167	1.40745876998571
H	5.96700979763328	1.69704670787808	-0.09754286526133
H	3.85190670484854	-0.05924080290892	2.08699433111248
H	-0.23186589472006	-0.02235537271081	2.16092448940000

Products for TS3G

C	-2.32014188592830	-0.53439301814604	-0.90550006913310
C	-1.63212678613601	-0.08657412150575	0.26772968360474
C	-2.38113424298179	0.48658162903565	1.29792211134783
N	-3.69652466139703	0.70829402591432	1.33679617029249
C	-4.33506232124085	0.31264737368899	0.24986453602012
N	-3.68824798005594	-0.27600996748866	-0.80677069458232
N	-0.30361610543848	-0.13718731111837	0.66113487862431
C	-0.25432866538905	0.38371170097910	1.87146236547231
N	-1.48088413033436	0.77230470714256	2.28565254850592
Pt	1.34491738790668	-0.75643370596860	-0.33653198836252
O	-0.31205953102285	2.70030945008139	-1.09270996071955
N	-5.65482119317081	0.48119870966115	0.14943902571103
O	-1.86939442749806	-1.07422300531906	-1.91252025340511
Cl	2.16492779037106	-1.77932786807196	1.59050899031169

N	0.66740131389886	0.14889100928308	-2.03251349529379
N	3.03473603358556	-1.35758927676895	-1.30080685540761
O	1.37062648417464	2.97330468488783	1.06743431128821
O	3.49833434447388	1.24807574956053	1.38414467100198
H	-1.69373361397964	1.19837950543815	3.18348786409823
H	0.64690790509308	0.49163579928210	2.46852372997976
H	3.63802342098959	-1.87611094286266	-0.65702684669569
H	2.84340233036940	-1.97386736596837	-2.09460366426760
H	3.58426401962234	-0.57180723424132	-1.65792057391263
H	-0.20216839608415	-0.32616192420178	-2.31987831928265
H	1.32171458761213	0.12778191533627	-2.81591106687088
H	0.42473629028441	1.13555467699531	-1.84720540053099
H	3.12876412406305	0.37431983929495	1.57907256133524
H	2.70486563298061	1.81028308830871	1.26425070318697
H	0.78956339073330	3.09547358476051	1.82799756109725
H	0.75743710849734	2.88493203510318	0.30749037674127
H	-1.23765991182527	2.56896761843222	-0.84079563639754
H	-6.18144442120127	0.17420643171732	-0.65757830343056
H	-6.14903450077402	0.90219378752381	0.92553523826456
H	-4.23224072227900	-0.56505757614837	-1.61745483463776
H	-0.32860105391903	3.47594750338268	-1.67081536395357

Reactants for TS4G

Pt	0.91168819176151	-0.50635503599512	0.09869168589730
N	-0.20743959115406	-1.71962475557126	-1.07315960395088
N	0.58851979870970	-1.65693800715174	1.74469663430627
H	-0.33051588794110	-1.40668104821185	2.14661485614628
H	1.29993602441871	-1.46133760171889	2.45278320249155
H	0.59949738146187	-2.66458804074759	1.57504866475309
H	-0.96638723204914	-2.19464846831202	-0.57829898344478
H	0.36595667837843	-2.45429346116264	-1.49635686359881
H	-0.63268033206190	-1.19938177874258	-1.84524932680762
Cl	1.22970424870631	0.88174482633973	-1.74600994295839
O	0.55833217848593	2.55192892401293	2.12708090594759
H	0.88690171144549	3.36113750313258	1.71215512490340
H	-0.31297494512754	2.38411389268689	1.66318961854204
O	2.06077449280145	0.65356704927068	1.32885319151802
H	1.48425744690947	1.42023523775227	1.68072821717043
H	2.79898444238805	1.08629161298052	0.80538628134273
N	-1.64503879339387	2.09458742064792	0.63561726483206
C	-2.36920308093466	0.93995160705452	0.45097833580235
C	-1.85467804247101	2.80906216086399	-0.44097028480562
C	-3.03386922669577	0.98032413427198	-0.77813905311835
H	-1.42660841568471	3.78949865607482	-0.64800138285950

H	-2.99149785496941	2.54365875884882	-2.22147346275449
N	-2.68920956383564	2.18300343830736	-1.32170208312656
O	3.80996723549363	1.77865433679435	-0.25170703168346
H	3.82967639697614	2.73101070502138	-0.08298154221664
H	3.18549520001772	1.68430553045287	-0.99687295310651
C	-3.96780668298034	-1.02817538941713	-0.56837710836902
N	-3.33127382311786	-1.17131297911951	0.64086084244271
N	-3.83063833797080	0.04882385913908	-1.31809918439157
C	-2.49616119463579	-0.23054243952062	1.25596866554688
O	-1.96761961308423	-0.48829464894451	2.33682800964486
H	-3.46401620044751	-2.03075289160243	1.16988696250296
N	-4.74081020329795	-2.03884221935523	-0.98009540325394
H	-5.22245657012894	-1.94727091025389	-1.86484563475755
H	-4.88811583597208	-2.87208997782569	-0.42659861858685

TS4G

Pt	1.90451971472761	-0.45214047578431	-0.08075983723846
N	1.84266597256395	-1.39771865184148	-1.86280032476947
N	2.61377411858646	-2.11282302936097	0.86969494995422
H	1.95516937482237	-2.89428201309603	0.83380894514009
H	2.76759737084066	-1.90238850376412	1.85896570283695
H	3.50392210402671	-2.44876367219401	0.49581219126766
H	0.91467572791291	-1.78619935987844	-2.05002872237328
H	2.50993260344332	-2.16635184663081	-1.95902212027209
H	2.04645947596118	-0.73439252058102	-2.61518325026495
Cl	2.56705758213397	1.83497885704210	-1.26174472424457
O	0.77563011124929	2.69982886259451	1.14576494847255
H	1.24199432893084	2.65072175441021	0.28309244448026
H	-0.14203096058068	2.49902166790747	0.91552113724628
O	2.02047983535860	0.50283130961286	1.73806892463685
H	1.43917721988375	1.32047029364985	1.66050030115263
H	2.94866791901307	0.89346282680422	1.73607187580260
N	-0.28609224918699	0.42707141152523	-0.69949624805265
C	-1.48531077856597	-0.06821451796999	-0.23701659654914
C	-0.58736882538680	1.17445815749080	-1.73416073839090
C	-2.53183922784598	0.41338036741179	-1.02744035916937
H	0.13707112353596	1.72654276657755	-2.33118195448969
H	-2.39924593393627	1.71157508151524	-2.70875004124284
N	-1.92981352675080	1.19844272562196	-1.96879439613352
O	4.34613592078155	1.54992702291580	1.27443480563685
H	4.54548213076649	2.37186768968065	1.74236490825823
H	3.99765717989881	1.83607424160093	0.40303332797890
C	-4.13866424193485	-0.63098649105616	0.10040002412891
N	-3.18236337928799	-1.15911601874241	0.92922436856379

N	-3.84462855826493	0.17169284793456	-0.90456495405670
C	-1.79680381373369	-0.94218466969648	0.85567338978777
O	-1.04974390688160	-1.47667109949555	1.66346610512636
H	-3.47377159847194	-1.77454739000484	1.68525432948890
N	-5.41214018592174	-0.97002013562559	0.33727289398075
H	-6.13501522355152	-0.54742376785345	-0.23009988503105
H	-5.68323740413572	-1.51812372072003	1.14261857833815

Products for TS5G

Pt	0.83871841994895	-1.05142931383616	0.22722255175607
N	0.22221278220759	-2.60462809514962	-0.90754941629055
N	2.38990736227138	-2.14981077241042	0.98200541339401
H	2.20425357473551	-2.47055693411532	1.93552436457335
H	3.23106893155103	-1.55562218381018	1.01166990736488
H	2.62725820196311	-2.98056977247037	0.43608250385562
H	-0.56416447790678	-2.35236868662954	-1.51135702796528
H	-0.08890068788655	-3.39672562245988	-0.33847397380154
H	0.95989028240849	-2.95479191887344	-1.52485068693476
Cl	2.93174633590069	1.46097797557726	-1.87190994271446
O	1.21450930956907	2.76828524774312	0.27338108184370
H	1.74824667182166	2.48756281153729	-0.51374723690466
H	0.37932525901925	3.09627127863808	-0.08402287740115
O	1.46347978845573	0.49133892149880	1.42633085182178
H	1.20479328170504	1.38648820584559	1.03046841637752
H	2.46242017443014	0.49929032448955	1.33742407122094
N	-0.63219584141696	0.08741666545582	-0.54137088661764
C	-1.91577103377176	0.25340236722346	-0.06198738795301
C	-0.51915616952194	0.85963847721806	-1.60651065192477
C	-2.58412052341708	1.16285087047600	-0.87761828908639
H	0.39823016418211	0.99411348791983	-2.18388357153124
H	-1.83719030020982	2.17601303015473	-2.59854819780951
N	-1.67601359381429	1.52294701008008	-1.83675085973254
O	3.95483346580832	0.26885462748743	0.73172929043970
H	4.66201927852526	0.78762489385643	1.13688107576023
H	3.77346727445570	0.69184594847381	-0.14964508529342
C	-4.48488761749726	1.08869776155765	0.27537516027298
N	-3.90602240310635	0.18882098196653	1.13478792995806
N	-3.84052253978779	1.60280618020099	-0.75781144350511
C	-2.59883463820517	-0.31208193943353	1.06305278511228
O	-2.18987988289116	-1.11309186095886	1.88911418403644
H	-4.45139466096169	-0.16514101131900	1.91780603807519
N	-5.75089871233291	1.44139156798161	0.51327820837476
H	-6.18797867566031	2.11449481754964	-0.10268926903000
H	-6.27799980057114	1.08321165853458	1.29883997025852

Reactants for TS5G

C	-2.62993042152240	-0.52866550456167	-0.23197680773427
N	-1.82592279272065	-1.62695962931970	-0.44076226314042
C	-2.62706663278864	-2.65678588889291	-0.34835426314445
Pt	2.00228290688937	-0.46001094300039	0.05511126749103
N	0.99533879838627	-2.13773558344554	-0.41657744209186
N	3.16253122609987	-1.42097238368453	1.38119775697614
H	2.62855019308244	-1.83736411791663	2.14933953899417
H	3.83160474633448	-0.76919356060339	1.80100143877225
H	3.70962749772508	-2.17382864195522	0.95427523195191
H	1.10727270413076	-2.89288392531138	0.26364088610823
H	1.30259195524020	-2.51491492282791	-1.31718107073036
H	-0.03241783414847	-1.94005401826761	-0.48500836090963
H	-2.33387946987170	-3.70130479010090	-0.45489481108038
H	-4.70978737678885	-2.92044910743277	0.02081070558762
O	0.85433013473265	0.60189302113227	-1.28730395578722
H	0.67623380772170	0.12302301358744	-2.11157775637781
H	-0.07013916363724	0.88457585347246	-0.89186147824461
O	3.04726492303544	1.24130818343290	0.55276069551071
H	3.12244603431158	1.82168337184804	-0.24760922111831
H	2.45455877008367	1.83203864410528	1.11816389935834
O	2.28885342081524	3.00077597611991	-1.22410802288727
H	1.68588441421557	2.35678476607254	-1.63686734167279
H	2.68112484218280	3.50498608298821	-1.94883725482932
O	1.39660054513239	3.00447317706243	1.43343666352596
H	1.48999872905782	3.38859922196621	0.54086705115616
H	1.75236602175394	3.68048034138403	2.02628964635677
N	-3.91879526909907	-2.29311767309164	-0.09068435260531
C	-3.95343361615060	-0.93421351811907	-0.00678469631271
C	-4.75160847147180	1.12297984581915	0.27114582348188
N	-5.02012400900504	-0.16890311665439	0.24189082028995
N	-3.48694232978111	1.61520849965107	0.05515112948535
C	-2.35453027332982	0.86321420616668	-0.20767150999042
O	-1.27880094114156	1.46367835155918	-0.38556678523247
H	-3.33876714376262	2.62244845516984	0.07438779974577
N	-5.71973649915051	2.00931021303986	0.51509136657274
H	-6.65776440110558	1.66706157566759	0.67786386876388
H	-5.54667502545557	3.00501452504053	0.55534180376072

TS5G

C	-1.60599465147453	0.09333199052491	0.03793197827284
N	-0.38134035625413	0.10014649287833	0.67240996794874
C	-0.65697407890287	0.05487482637070	1.95376320729108
Pt	1.81522182984291	-0.69800983737506	-0.31236050182514
N	0.70005294825083	-1.85832020443919	-1.53698366793409

N	3.16832202514048	-2.16336285139430	-0.09092882932639
H	2.78276726282711	-3.00639930877037	0.34218887104896
H	3.91722664571465	-1.82230695352880	0.51904424489125
H	3.60374844068084	-2.44298121531225	-0.97375583504566
H	0.00730633196155	-1.28918108089235	-2.04529707233498
H	0.18145478345276	-2.57474714817818	-1.02177764734646
H	1.25994880898318	-2.34689469876401	-2.23963411445139
H	0.08408738991421	0.08300405226660	2.75385135389284
H	-2.44701558933473	-0.01602801188872	3.10101640561649
O	1.04898180240267	1.34244735701742	-1.34109172463647
H	0.28229884464766	1.03752829439064	-1.88983722516584
H	0.61869927369298	1.78049734194556	-0.58991375496682
O	3.04684914230925	0.42220869888616	0.86889334744721
H	3.34759919319205	1.21426268169787	0.32192040285395
H	2.63792199607844	0.73326574017471	1.72811464761488
O	3.56494866420157	2.32375299413228	-0.79902792583827
H	2.73878638297512	2.16853441031112	-1.29635834179127
H	4.26472834231706	2.04566080509556	-1.40623677021652
O	2.05996066362351	1.03509277379798	3.20142837101418
H	1.91437803728543	1.97557007178327	3.37790006258186
H	2.68384633467589	0.75207886384125	3.88506602738822
N	-2.00157882000354	0.01684736877158	2.18841683604783
C	-2.63735405652510	0.04140814466941	0.98181584671664
C	-4.27373296085402	0.07383206140133	-0.52755094096850
N	-3.95301164108001	0.02406013485813	0.75323261558344
N	-3.32741648434570	0.14419545918718	-1.52355300350204
C	-1.95490536363788	0.16495255535302	-1.33601068334301
O	-1.19348678378452	0.23575998662850	-2.31821491181153
H	-3.63128095111674	0.19617516014479	-2.49450240646861
N	-5.55577579067728	0.05910727468904	-0.89845262637046
H	-6.26491881998015	0.00975693202326	-0.17833445096285
H	-5.84434879619901	0.08987883770257	-1.86717175190411

Products for TS5G

C	-1.57391995946135	-0.30615091954508	-0.23652018081796
N	-0.43863961546756	-1.03952216757640	-0.51904068257060
C	-0.82689268295470	-2.28685405709252	-0.69087840641629
Pt	1.42222620560860	-0.28497873535961	-0.47682001759815
N	1.31931848026835	0.10301622283431	-2.45849637157988
N	3.25576568705133	0.61007701241618	-0.36927874350741
H	3.80919052050484	0.58062561291279	-1.22802904098631
H	3.84648411169197	0.22599636383155	0.37177277384246
H	3.07345206229538	1.59878823804997	-0.14417783861172
H	2.14321783200614	-0.21766329443370	-2.97386207556592

H	1.22705215727001	1.10596178163571	-2.64240655300916
H	0.51092106432612	-0.34802511005017	-2.89413117674527
H	-0.17458032597661	-3.12678768811806	-0.91981782266175
H	-2.70033111406755	-3.25837129512062	-0.62459822230356
O	1.60090905460781	2.86246357260793	0.48302512444379
H	1.52384833051289	3.82428588462982	0.44553921568054
H	0.70735842799490	2.51702604993699	0.26164221240436
O	1.55171862612411	-0.70120388488021	1.52060894711869
H	1.71789417388036	0.14848003564739	2.05532223083446
H	0.70817377110546	-1.09987173608320	1.88502120976537
O	2.01359130617036	1.50226316727662	2.74486043181041
H	1.86054396156240	2.14414512117443	2.00677225217701
H	2.96241191373601	1.56083637963283	2.92143359187823
O	-0.67830479344819	-1.71693600520699	2.44656810487590
H	-1.25418598123361	-1.01330803057534	2.77715983316765
H	-0.56338718313173	-2.30777310176105	3.20353252518160
N	-2.16634965853565	-2.39807531328643	-0.53663023663441
C	-2.67275490855280	-1.16365491940210	-0.23847407620182
C	-4.14861384123528	0.42298039719085	0.26090920547937
N	-3.94984099732621	-0.85724482227889	-0.00185863684784
N	-3.12597406894429	1.33951124890215	0.27948728777463
C	-1.77607010712109	1.08028651132695	0.04201462801557
O	-0.96046143822279	1.99688479857522	0.08838247980210
H	-3.34057930764123	2.31470503628023	0.47961612918778
N	-5.37909918606562	0.86992487991639	0.51824506760975
H	-6.14287378537344	0.20612433447169	0.52547052432275
H	-5.56755373195731	1.83487543152035	0.75618530668559

Reactants for TS6G

N	2.39808164176286	-1.12802387786360	2.49345902774361
C	1.37112645937207	-0.37110928468493	2.04181762821848
N	1.53745794985570	-0.06382213398301	0.76960070751798
C	2.72229380581282	-0.65607330991435	0.37254757471915
C	3.27642812173612	-1.32426545612655	1.46438938718167
Pt	0.29017280224695	1.14619610473250	-0.26269601260351
Cl	0.47236003498060	2.77105404814963	1.40730549111271
N	-1.03575855266480	2.35773195766310	-1.23029321808032
N	0.16712897839263	-0.25788847364998	-1.72795537661673
N	-1.84375694562773	-2.12286893418052	-0.74259484828269
C	-2.13414324651313	-2.96386657627571	0.21716836457026
N	-3.37076812027362	-2.73758023844075	0.75628849330380
C	-3.90556746131655	-1.66141741992881	0.11586611857662
C	-2.94272471420074	-1.29539655188175	-0.83247434928932
H	-3.79500866746372	-3.25109542461629	1.52245363775087

H	-1.49108069422013	-3.76745438123706	0.57596769644895
H	-0.50404760756401	-0.00209448235564	-2.45978959062770
H	-0.20449527415848	-1.14509435628164	-1.35050300597742
H	1.10193746834332	-0.40150946167445	-2.13111204649659
H	-1.53548951704248	1.87852575076670	-1.99003844350626
H	-0.60474952314660	3.20236732543115	-1.60977157205126
H	-1.75375821669313	2.64426131786904	-0.54937476655312
H	0.50790045154671	-0.08285057898384	2.64013870758720
H	2.49373639459887	-1.49007353684873	3.43807833137617
O	-1.83963373509802	-0.13144029338827	2.06344120106857
H	-1.43238287908111	-0.39758186495549	1.22765661389541
H	-2.22777508109012	0.73568205830664	1.83256338479335
O	-2.60265248086321	2.42182019335872	1.23519553641039
H	-3.27706837406487	2.99060994345169	1.62858102829111
H	-1.74834290968816	2.76851254898189	1.56066316745788
C	-5.33192449189587	-0.03670944293112	-0.40660281288488
N	-5.08631034698180	-1.08112522850629	0.36159484142228
N	-4.45639166999439	0.38796436444966	-1.37428107148062
C	5.07124981739802	-2.04205113642900	0.36377108853438
N	4.41908174716964	-2.01398314413057	1.51243844854353
N	4.59655304185401	-1.42662816742983	-0.76763833962008
C	-3.22136317185491	-0.18433437795292	-1.68150092376909
O	-2.53586492675488	0.30013686637048	-2.58805107459952
H	-4.70349326742612	1.19354320841895	-1.94495281913430
N	-6.46943719458256	0.65202763851945	-0.26180736379465
H	-7.12193240142641	0.36005997289185	0.45370386049490
H	-6.68610159787267	1.46708354115819	-0.81999974551775
C	3.40300272235314	-0.70986317051351	-0.88478249864766
O	3.06489431604733	-0.24338958633115	-1.96855552577005
H	5.13336222769634	-1.49720369385740	-1.62991832815953
N	6.23804121952945	-2.68498023144838	0.27469201939718
H	6.58714503271439	-3.16382406284015	1.09487644591542
H	6.75004883614928	-2.76680796087789	-0.59365506886885

TS6G

N	-3.40835094746768	-0.81921921801150	1.89200421882026
C	-2.35042899623463	-1.52145919645870	1.42228934154782
N	-1.83525386882127	-0.94556535717796	0.35481410935633
C	-2.59649361613763	0.18733408161981	0.11633764173751
C	-3.59049812317655	0.27360757687003	1.09324974290027
Pt	-0.15540856619831	-1.52938383569774	-0.62562387428119
Cl	0.50463815128441	-3.05204385273772	1.43561737828837
N	1.55949546709504	-1.98086594301912	-1.62243147821272
N	-1.16597773148170	-1.40516052321061	-2.40046128092383

N	1.19235117014048	-0.01434153486700	0.68301693315880
C	1.09359528353300	0.18014829913182	1.97290527307135
N	1.64519112829211	1.36958341869929	2.35147451936603
C	2.14942705286553	1.96673045059262	1.23439222888818
C	1.85316989406732	1.08580477140023	0.18998374971436
H	1.70419689864135	1.72865041445324	3.29913419016312
H	0.63210289246933	-0.51717354689796	2.67047544841058
H	-1.42608869989008	-0.41424293739088	-2.52485168581078
H	-2.02045485761624	-1.96453508273076	-2.41053141888341
H	-0.61619604479063	-1.69221502227193	-3.21174801501163
H	1.88486135286570	-1.09679311221799	-2.04591121581131
H	1.47217098868702	-2.68721363095600	-2.35422919335971
H	2.29681484408091	-2.28611193789734	-0.96643142449547
H	-1.95503653725277	-2.42332698730994	1.88532257927166
H	-3.96369381724604	-1.06370859452936	2.70701251694754
O	4.49225421562966	-0.19489158645678	1.61051935984160
H	4.47992547734913	0.38741923324978	0.84283974023866
H	4.08575013805544	-1.01449379863775	1.26349430904837
O	3.35938256650295	-2.49503641052442	0.57408101702849
H	3.86411421731290	-3.31801713620759	0.61605970061124
H	2.50538722425766	-2.70041204728519	1.02482975791615
C	3.17833330178465	3.45998608040748	-0.05330156291001
N	2.79621568328574	3.13798579842759	1.16773691838023
N	2.93540091195482	2.65789474902282	-1.14037462088487
C	-4.46561915262549	2.16914788922603	0.32508626905730
N	-4.52565234299268	1.21609123813905	1.23736911403056
N	-3.52662801984306	2.16842490074181	-0.67540642644728
C	2.27897286156070	1.42274201084030	-1.13046864713629
O	2.15183376961962	0.79058239622425	-2.17880409649681
H	3.26265248694688	2.95940489107781	-2.05541109453928
N	3.82803733497593	4.60858701043634	-0.27447543513100
H	4.04362275380846	5.20136704360667	0.51610558946490
H	4.17541347971241	4.86485820102822	-1.18864164042902
C	-2.53929769418686	1.20483891399150	-0.88829418639855
O	-1.78341076446316	1.30740558099091	-1.85211253464392
H	-3.53567561532766	2.92027018571617	-1.36231213241517
N	-5.33851734699502	3.17884522416802	0.35320166247445
H	-6.03803645571928	3.19277005755911	1.08409193762640
H	-5.32892234831245	3.92595087487321	-0.32873328313827

Products for TS6G

N	-2.84954755731194	-0.79422044712426	2.20866629319513
C	-1.67271830556109	-1.17557481533360	1.65666548277761
N	-1.55480955269200	-0.69161032329173	0.43412177062995

C	-2.71032740929286	0.02035152970824	0.17619643610696
C	-3.53208338431606	-0.03301735448692	1.30014023468642
Pt	0.05123323156073	-0.89194320834398	-0.78315472211534
Cl	0.95029925210170	-3.37065797968567	2.05253553749898
N	1.68947068209370	-1.12002783707926	-1.96872343339241
N	-1.17785045925010	-1.83419091008439	-2.11195682797657
N	1.21667319212395	0.17211549757432	0.48534744801013
C	1.50118008026972	-0.10382337213630	1.74362513098766
N	2.19041867122233	0.91139988283816	2.31260959251475
C	2.39511695442732	1.88019147963004	1.37268142519200
C	1.77676738779709	1.40814026865349	0.21658146025663
H	2.54548041118001	0.91821961967534	3.26410032535316
H	1.26038011217473	-1.05322518369405	2.23560812589233
H	-0.71575219884542	-2.25761833803069	-2.91863938714909
H	-1.82369782839129	-1.11341137236829	-2.46320269554730
H	-1.74205673604093	-2.56841263206348	-1.67807111148450
H	2.03176073489603	-0.19746707802918	-2.25812253248094
H	1.54296828111369	-1.66916936656932	-2.81692206807381
H	2.40116128033364	-1.60778474582892	-1.39663960139760
H	-0.93832949659088	-1.83353003415873	2.13560057659162
H	-3.17075693628687	-1.04184237653022	3.14039692470766
O	4.52500213100521	-0.30826113065560	0.48590408459461
H	4.20575221727218	0.20768794052527	-0.26292593134499
H	4.05402780935018	-1.15914343460843	0.37963219271037
O	3.10681105277464	-2.61940371631158	0.00761283735227
H	3.56590214041831	-3.43744413786938	-0.22220084420939
H	2.41336990641077	-2.88401608213370	0.66815097299221
C	3.12582640650721	3.76401941887049	0.44230518150632
N	3.05873737482282	3.02774294660944	1.53735015855957
N	2.58186612732081	3.36112495656551	-0.75157643680647
C	-5.16121030853567	1.18507948683562	0.39892414257749
N	-4.73506788734178	0.52496121818097	1.46170497616254
N	-4.43496101959434	1.25951379464884	-0.76434090174683
C	1.89294942013450	2.16766014717237	-0.98850585462489
O	1.51109461894924	1.88364897697269	-2.11923362818164
H	2.70388123968385	3.95069633351525	-1.57221726673840
N	3.74719135975823	4.94583357271352	0.46831551800898
H	4.15499514224653	5.25712392451383	1.34023854355442
H	3.81136747026567	5.54473653008836	-0.34395051320208
C	-3.19021326292789	0.67259029585724	-1.00197186418641
O	-2.67038376911906	0.73569604947879	-2.11203007984942
H	-4.82875501747539	1.75688516540952	-1.56037750860423
N	-6.34169093872792	1.80802841385899	0.42859571131296
H	-6.89658323737623	1.74953042448009	1.27259413922324
H	-6.71590038253706	2.30196800204123	-0.37082601384367

Reactants for TS7G

N	-3.89527720122479	-1.88986822446153	-1.55081541273829
C	-2.75424400045785	-2.07482569530741	-0.84746487675696
N	-2.41989099086147	-0.97887980004132	-0.19481070990576
C	-3.38692863737799	-0.03585205705099	-0.48736648580463
C	-4.32588589081466	-0.60871497052347	-1.34631857734394
Pt	-0.77650073015708	-0.74222696193859	0.95077922680762
O	0.14165687863545	0.16053714588246	-0.63551334799273
N	0.91382725959712	-0.47946633483219	2.06216990961442
N	-1.71234043057054	-1.64786835686820	2.50069664443973
N	2.68028429429162	-0.59530808932274	-0.63685982712136
C	2.88581236978919	-1.80266010288880	-1.10665004724189
N	4.19745683547488	-2.02239397001803	-1.39780273245605
C	4.88540372037040	-0.88050755723663	-1.10309007175514
C	3.91884752833926	0.00729287401913	-0.61954574727390
H	4.59270409167693	-2.87967716186776	-1.77295790328227
H	2.11789259964151	-2.55988167771462	-1.26409598916471
H	-1.20847453395891	-2.47767412830095	2.82567330548801
H	-2.65818873732755	-1.95632367688437	2.26229983633545
H	-1.80450539496160	-1.02479598192507	3.30785992682476
H	1.71807776098906	-0.90613857591255	1.59273800148504
H	0.88061775359664	-0.86500052476200	3.00741217910138
H	1.12630937030084	0.53521731018619	2.13812017139664
H	0.00887490801313	1.16711866687394	-0.52524805106221
H	1.13516371587718	-0.03070985147037	-0.61523662955748
H	-2.20123136462032	-3.01178251823805	-0.83409175568022
H	-4.35053845518471	-2.58933260779758	-2.13112971653719
O	-0.29856553325035	2.61496471880832	-0.11243453605739
H	0.29361739266650	2.65291463489172	0.67418881509828
H	-1.20125803565841	2.49902053518177	0.24943985748470
O	1.53598669876353	2.26743734753831	1.81947195580926
H	1.74635262650385	2.86455291809768	2.54902841959376
H	2.34724699634360	2.24310125918128	1.24993973023488
C	6.58338791835239	0.54066899842180	-0.90393160908549
N	6.19631893243518	-0.67019650601119	-1.26006402228243
N	5.71067787230384	1.47574872598365	-0.40533239140093
C	-5.59018710415696	1.21431529493419	-1.49244045226569
N	-5.41577674449520	-0.04157109110813	-1.86570258355086
N	-4.71968383108337	1.85909656565274	-0.64892425467244
C	4.34005382113550	1.30764406020374	-0.21137918677060
O	3.67945483381133	2.23675483198774	0.25729020133407
H	6.06886819158986	2.38949643751943	-0.13512650321585
N	7.86696733045099	0.89421649553868	-1.01605872168269
H	8.51857695059328	0.22385761658445	-1.40277769777432
H	8.19641150675838	1.82417907564670	-0.79414217861674
C	-3.56519713187679	1.32181530751045	-0.08096755195152

O	-2.86414916321567	2.00998440708771	0.65869125494051
N	-6.64256017200532	1.90382910961242	-1.93467470228130
H	-7.28838135165460	1.44839221176236	-2.56681772551222
H	-6.79663335802288	2.87352178051050	-1.69233354129698
H	-4.90604136536444	2.82802809286518	-0.39697389589628

TS7G

N	2.71719657302432	-0.44782870324617	2.39787997942087
C	1.64122733785692	0.25814619430916	1.98073879286765
N	1.57902848567008	0.29887590661076	0.66387325938632
C	2.67420740873223	-0.41220118318531	0.20205129228473
C	3.39341367303348	-0.89390505515024	1.29775784281664
Pt	0.07346233060672	1.16518777009095	-0.39160495680206
O	-1.43117262106763	1.22737448408009	1.44478924342360
N	-1.43999894707115	2.07560364415064	-1.38610749850262
N	1.43927479168342	2.10118967347572	-1.54863116124449
N	-1.14414766008159	-0.98359200928681	-0.19496752491362
C	-0.50888703193014	-2.07605916065702	0.15186796626053
N	-1.36373051312066	-3.10214509762194	0.42701053407889
C	-2.63513401811240	-2.64130406947941	0.24769846010525
C	-2.48352324236939	-1.30788397017187	-0.14879150988362
H	-1.10207392046600	-4.03977285801787	0.71639013658794
H	0.57211731746385	-2.18769027160779	0.22722931954045
H	2.07638354070874	2.68292239420749	-1.00021950828950
H	1.03046046370257	2.70673535111212	-2.26287077882271
H	1.99638482307303	1.37147726216104	-2.02367844274218
H	-2.33142977545999	1.58054453782894	-1.18985122092013
H	-1.32468244945485	2.09983161501452	-2.40059945869941
H	-1.52610177677496	3.04845180887368	-1.05287587560702
H	-1.40719718383952	0.48508571759860	2.06102991372332
H	-1.12460186776337	2.03777501112205	1.93511426463552
H	0.93079797935026	0.72159874037991	2.66055898333121
H	2.97260481720544	-0.61772229956835	3.36694616409480
O	-0.55090425615691	3.53129545856315	2.34229210765677
H	-0.82667729314711	4.02592894575173	1.53775746212272
H	0.41300677476336	3.50216377499288	2.28005619956910
O	-1.41419282482164	4.65312908754204	-0.00565218085164
H	-0.82801249393752	5.31509106408411	-0.39945425266614
H	-2.26431574846253	5.10747458669869	0.08165711856774
C	-4.85748938488060	-2.63231343300840	0.16840522360232
N	-3.76812223216575	-3.33360127435985	0.41841245948215
N	-4.81206866391404	-1.32227602610441	-0.23701792409500
C	4.93847000963539	-1.93088255381195	0.07969538950202
N	4.50130583627961	-1.63747606280563	1.29193081635829

N	4.30671428374273	-1.48938285358116	-1.05608168780464
C	-3.66253405506005	-0.55532799754526	-0.44410327448521
O	-3.77641229140979	0.60330797860823	-0.84035504418055
H	-5.68457252340134	-0.83466534765606	-0.42931732809591
N	-6.06366234495540	-3.19308401104485	0.30653716864399
H	-6.11590305493959	-4.16021693507437	0.59806555689037
H	-6.92070469341485	-2.69265106336711	0.11240648214049
C	3.15889015723815	-0.69997609301218	-1.11326387000829
O	2.71232894710172	-0.33931651937155	-2.19966888935789
N	6.03293342447486	-2.67705343157910	-0.07558414813494
H	6.50876649526659	-3.01576816390374	0.75078333066876
H	6.39054984463760	-2.93302761758759	-0.98653548420318
H	4.70459755292773	-1.72306694545050	-1.96367344745176

Products for TS7G

N	2.95476010456649	-2.28879859239092	-0.79979552950524
C	1.90092328277179	-1.72863879413787	-1.43630431550634
N	1.45427541817556	-0.67617250918198	-0.77821242957802
C	2.26532383778381	-0.53652483891270	0.33318424664426
C	3.21521755544158	-1.56114778887921	0.32677584558423
Pt	-0.11858907353708	0.50079954477791	-1.27198673982979
O	-0.54364174197200	3.11088714299725	1.30050236754624
N	-1.61632047597048	1.78763995449086	-1.76671448134199
N	1.16295278484511	1.61415937448709	-2.39119739935715
N	-1.38093583016991	-0.74401255968630	-0.23694634507096
C	-1.26000753283658	-2.05666510740170	-0.31980606898398
N	-2.17443560495802	-2.69208790988577	0.44283530588303
C	-2.94581844639674	-1.75167765625375	1.06026616038896
C	-2.44766578021846	-0.51838270676228	0.62533060981437
H	-2.26991642612244	-3.70030006598695	0.53368509700537
H	-0.53201318504071	-2.59380962334821	-0.92132513982980
H	0.69422517114194	2.23513798358189	-3.05348070647552
H	1.78027163628543	1.01440481459824	-2.94275422940233
H	1.76501756519818	2.21644188148765	-1.78506188789131
H	-2.06214939905976	1.57775962584372	-2.66157165577601
H	-1.23077816254292	2.74706112041724	-1.79946465912353
H	-2.33819131001169	1.81294317825941	-1.03343742196969
H	0.13859091084476	2.41554308964879	1.35824412085927
H	-1.38626568922842	2.62746643223693	1.19311844417482
H	1.48745913817864	-2.11630716722724	-2.36593829184814
H	3.46717806532290	-3.10866337356162	-1.11476498850701
O	2.42732483068224	3.35826453548220	-0.61179129854307
H	1.59216512457965	3.85409402485393	-0.75746969684825
H	2.24329943050859	2.84368310549497	0.19283218270984

O	-0.14151290504239	4.16155200550999	-1.06828365517961
H	-0.44898991836552	5.07541196211607	-1.11814420608626
H	-0.34616936304800	3.85876355399617	-0.13677424126307
C	-4.55041661819599	-0.89513931611962	2.33984996442799
N	-3.95869845673891	-1.98821831231932	1.89202969249512
N	-4.14991017476996	0.35821674800412	1.95156358430167
C	4.22901532418407	-0.89772429525565	2.19145318259745
N	4.18431663840673	-1.78575282922941	1.21283711839265
N	3.36178564453433	0.16548718480451	2.25694750546566
C	-3.11544755063722	0.65679914088325	1.07608666124733
O	-2.89810066058161	1.83578919405970	0.77312888663194
H	-4.65199659082122	1.16945919723629	2.30822595228713
N	-5.57028952254622	-0.98179619279057	3.19224180870524
H	-5.88693429939972	-1.90017506251489	3.47587024787085
H	-6.06021155671426	-0.16666867868630	3.53697081245502
C	2.34789271569839	0.45881156200741	1.35102412094345
O	1.68827894064980	1.49142452287098	1.48715535098225
N	5.14172521826052	-1.01093523325235	3.15540128259730
H	5.78790315661405	-1.78907261382769	3.12008512890122
H	5.20495090224528	-0.35710493992001	3.92451229300340
H	3.46679887800635	0.84278628738570	3.01070541400093
